# Actinomycetes-derived imine reductases with a preference towards bulky amine substrates

**DOI:** 10.1038/s42004-022-00743-y

**Published:** 2022-10-08

**Authors:** Jun Zhang, Xin Li, Rongchang Chen, Xianwei Tan, Xiongduo Liu, Yaqing Ma, Fangfang Zhu, Chunyan An, Guangzheng Wei, Yongpeng Yao, Lujia Yang, Peng Zhang, Qiaqing Wu, Zhoutong Sun, Bin-Gui Wang, Shu-Shan Gao, Chengsen Cui

**Affiliations:** 1 Tianjin Institute of Industrial Biotechnology, Chinese Academy of Sciences, Tianjin, 300308 China; 2 School of Life Science, Hebei University, Baoding, 071002 China; 3 Key Laboratory of Experimental Marine Biology, Institute of Oceanology, Chinese Academy of Sciences, Qingdao, 266071 China; 4 ReadCrystal Bio-tech Co. LTD, Suzhou, 215505 China; 5 State Key Laboratory of Microbial Resources, Institute of Microbiology, Chinese Academy of Sciences, Beijing, 100101 China; 6 National Technology Innovation Center of Synthetic Biology, Tianjin, 300308 China; 7 Tobacco Research Institute of Chinese Academy of Agricultural Sciences, Qingdao, 266101 China

**Keywords:** Biocatalysis, Enzymes

## Abstract

Since imine reductases (IREDs) were reported to catalyze the reductive amination reactions, they became particularly attractive for producing chiral amines. Though diverse ketones and aldehydes have been proved to be excellent substrates of IREDs, bulky amines have been rarely transformed. Here we report the usage of an Increasing-Molecule-Volume-Screening to identify a group of IREDs (IR-G02, 21, and 35) competent for accepting bulky amine substrates. IR-G02 shows an excellent substrate scope, which is applied to synthesize over 135 amine molecules as well as a range of APIs’ substructures. The crystal structure of IR-G02 reveals the determinants for altering the substrate preference. Finally, we demonstrate a gram-scale synthesis of an analogue of the API sensipar *via* a kinetic resolution approach, which displays ee >99%, total turnover numbers of up to 2087, and space time yield up to 18.10 g L^−1^ d^−1^.

## Abstract (web summary)

Imine reductases can catalyze reductive amination reactions to produce chiral amines, however, transformation of bulky amines has been challenging. Here, by using an increasing-molecule-volume-screening method, the authors identify a group of imine reductases that can accept bulky amines and achieve an efficient gram-scale synthesis of an API sensipar analogue.

## Introduction

**Fig. 1:**
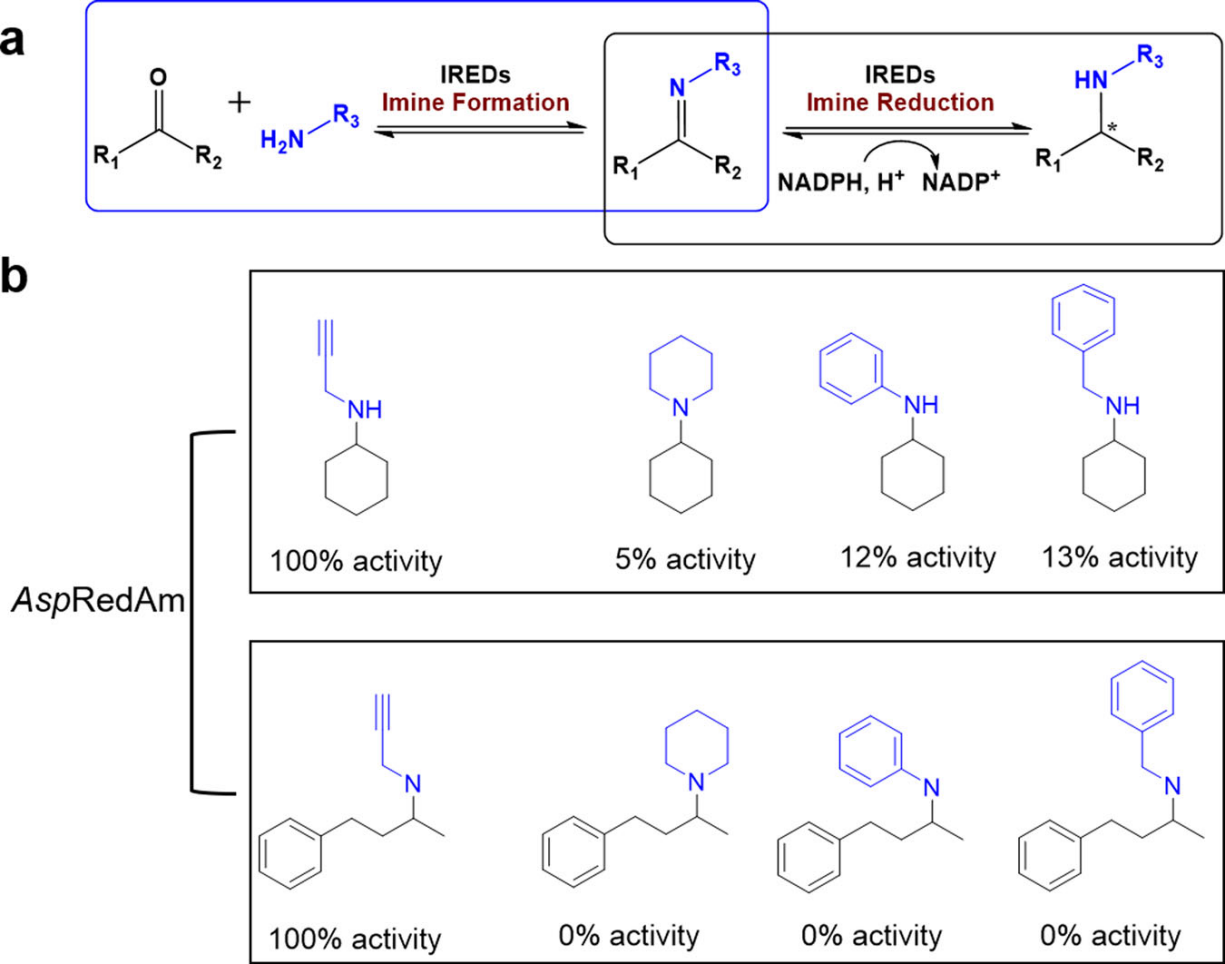
Synthesis of chiral amines by IREDs. **a** IREDs are capable of catalyzing imine formation and imine reduction to generate a wide variety of chiral amines. **b** The reported relative activities of *Asp*RedAms towards propargylamine and larger amines piperidine, benzylamine, and aniline.

Biocatalysis is an attractive approach for the synthesis of chiral amines, which constitute 40% of current pharmaceuticals approved by the FDA^1^. The employed biocatalysts include IREDs, lipases, transaminases, ammonia dehydrogenases, and monoamine oxidases^2,3^. Since IREDs were discovered in 2010^4^, they have attracted the catalytic research community for their potential application in the asymmetric synthesis of chiral amines^5,6^. Recently, reductive aminases are identified as a subfamily of IREDs by Turner and co-workers^7^, which could create diverse chiral amines through reductive aminations of carbonyl and amine donors provided in equal stoichiometric ratio (Fig. 1a)^7^. Based on the broad substrate scope, IREDs have been engineered for industrial production of LSD1 inhibitor GSK2879552 and JAK1 inhibitor abrocitinib^8,9^. Thus, IREDs become an efficient and economical way to rapidly construct chiral amine products^8,10–17^.

However, the industrial application of IREDs is mainly restricted by limited substrate scope, especially low activities towards bulky amines^18^. For example, the first characterized *Asp*RedAm showed excellent activities towards small amines, such as cyclopropylamine, propargylamine, and allylamine, but exhibited poor activities towards large and sterically hindered amines^7,18^. Specifically, for piperidine, benzylamine, and aniline, *Asp*RedAm retained only around 10% activity compared to propargylamine when they were reacting with the active carbonyl donor cyclohexanone (Fig. 1b)^7^. Furthermore, *Asp*RedAm displayed no activity towards those amines reacting with the more challenging carbonyl donor 4-phenyl-2-butanone^7^. However, only a few IREDs showed good transforming activities when using benzylamine as amine donor in the previous studies^13,15^. Due to the increasing desire for biocatalysts for the synthesis of APIs, designing a comprehensive screening to identify IREDs favoring bulky amines is of great practical significance.

As more and more IREDs are reported, screening of the promising enzyme for subsequent engineering and industrial application is a laborious job. In this study, a rapid protocol of Increasing-Molecule-Volume-Screening was constructed. Using Increasing-Molecule-Volume-Screening, we identified three IREDs with the preference towards bulky amine substrates. We further evaluated their abilities for the synthesis of APIs, together with their application to the gram-scale synthesis of a bulky *N*-alkylated benzylamine. The X-ray crystallography provides insight into the structural basis of the substrate preference. This study expands and deepens the research aimed at applying IREDs for asymmetric reductive amination reactions.

## Results and discussion

### Sequence diversity of IREDs

To investigate the preference of IREDs in reductive amination towards different amine substrates, *Asp*RedAm and 85 reported IREDs in our lab were selected (Supplementary Fig. 1)^19^. All 86 IREDs already showed excellent activities towards certain substrates^7,13,15,17,20^, which made them being a good starting point for this study. Previous studies of those enzymes mainly focused on small amines^8,10–17^, including methylamine, dimethylamine, cyclopropylamine, butylamine, isopropylamine, allylamine, pyrrolidine, piperidine, and propargylamine, etc. Screenings were also performed towards arylamines^7,15,18^. For instance, IR-G02, IR-G03, IR-G04, IR-G05, and IR-G08, showed excellent (>90%) conversions towards arylamines, including benzylamine, aniline, and 3-aminopyridine^14^. However, more bulky amines have rarely been examined.

### Identifying IREDs for bulky amines

**Fig. 2:**
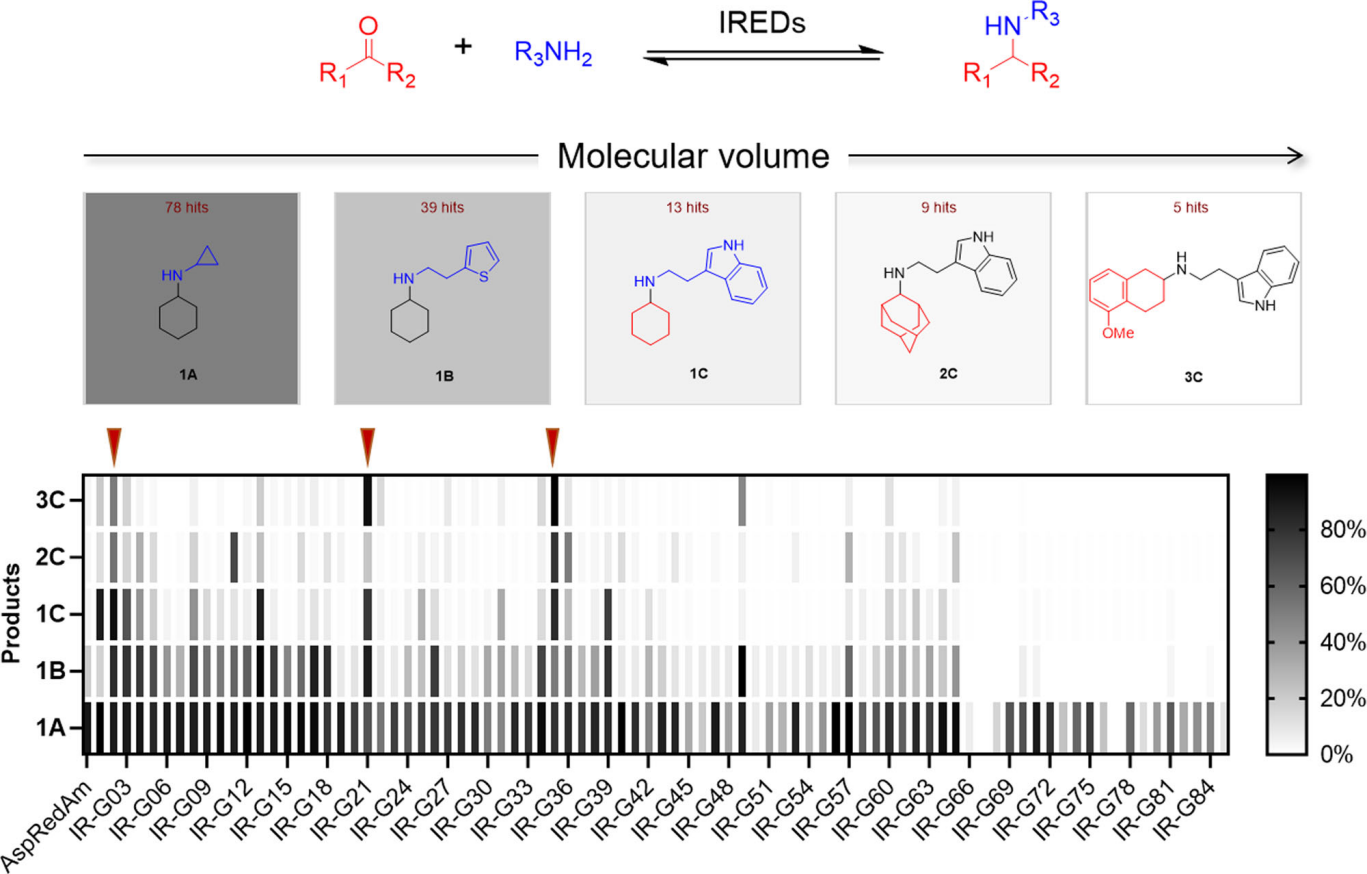
Screening for IREDs using the increasing-molecule-volume-screening approach. The number of enzyme hits is shown above the structure of the corresponding secondary amine. The black color scale in each tile is representative of the number of hits generated through the screening with the given substrate. The standard assay mixture (50 μL) contained 5.0 mM ketone, 5.0 mM amine, 1.0 mM NADP^+^, GDH (0.2 mg ml^−1^), D-glucose (30 mM), 100 mM potassium phosphate buffer (pH 7.0), and 1 mg mL^−1^ IREDs. The conversion of reductive amination products was determined by comparison with UV-Vis absorptions of synthesized standard reference materials. The enzymes (IR-G02, 21, and 35) with red triangle block are potent IREDs.

All 86 IREDs were successfully obtained by expressing their genes in *E. coli* BL21(DE3). Subsequently, their catalytic potential toward bulky amines was explored by using Increasing-Molecule-Volume-Screening. It is a rapid protocol to screen the preferable substrate size of IREDs by synthesizing a group of targeted products with increasing volumes. We targeted five structures (Fig. 2): i) **1** **A**, **1B**, and **1** **C** could be synthesized from the active carbonyl donor cyclohexanone **1** with increasingly-bulkier amines, cyclopropylamine **A**, thiophene-2-ethylamine **B**, and tryptamine **C**, respectively; ii) **2** **C** and **3** **C** could be synthesized from the amine **C** with the non-active and bulkier carbonyls, adamantanone **2** and 5-methoxy-2-tetralone **3**, respectively. An equimolar ration of carbonyl and amine were used for all reactions (Supplementary Method 1). Conversions have been determined by comparison of the peak areas of UV absorption or extract ions of amine products with standard amins using HPLC or LC-MS (Supplementary Method 2 and Supplementary Data 3).

In the reductive aminations of **1** with **A**, **B**, and **C**, the number of enzyme hits (conv. >20%) has fallen significantly with the addition of bulkier amines. As shown in Fig. 2, the number of enzyme hits for **1** **A**, **1B**, and **1** **C** were 78, 39, and 13, respectively (Fig. 2 and Supplementary Table 1). Similarly, *Asp*RedAm gave a >90% conversion for **1** with **A**, however, only 19% conversion and <5% conversion was observed for **1** with **B** and with **C**, respectively. These results confirmed the low tolerance of IREDs for bulky amines. Further analysis indicated only 9 and 5 enzyme hits were observed for **2** **C** and **3** **C**, respectively, while the rest of the enzymes exhibited weak or no measurable activities.

The activities of most enzymes are negatively correlated with the volumes of targeting molecules, such as IR-G08, IR-G13, and IR-G39, which were active for **1** **A**, **1B**, and **1** **C**, but lost activities for **2** **C** and **3** **C** (Fig. 2 and Supplementary Table 1). Only a few untypical examples, such as IR-G11 and IR-G49, showed better activities towards certain targets with larger volumes. Thus, the Increasing-Molecule-Volume-Screening could rapidly evaluate the tolerance of IREDs towards bulky substrates. Finally, only three IREDs were obtained: IR-G02, 21, and 35. They were constantly active for all reactions and all displayed >50% conversions for **1**, **2**, and **3** with **C**.

Phylogenetic and bioinformatic analysis suggested the IREDs IR-G02 (*Streptomyces albidoflavus*), IR-21 (*Saccharothrix espanaensis*), and IR-35 (*Allokutzneria albata*) were all derived from actinomycetes and belonged to the same evolutionary branch with sequence identity ranging from 40 to 44% (Supplementary Fig. 2). Overall, the IREDs from bacteria and fungi showed better activities towards bulky amines **B** and **C** (Fig. 2), compared to those from plants and humans. Based on the initial results, the IREDs (IR-G02, 21, and 35) were further evaluated for the synthesis of APIs against bulky carbonyls and amines.

### Evaluating enzymatic synthesis of APIs using IREDs

#### Reactions of 4-phenyl-2-butanone with bulky amines

**Fig. 3:**
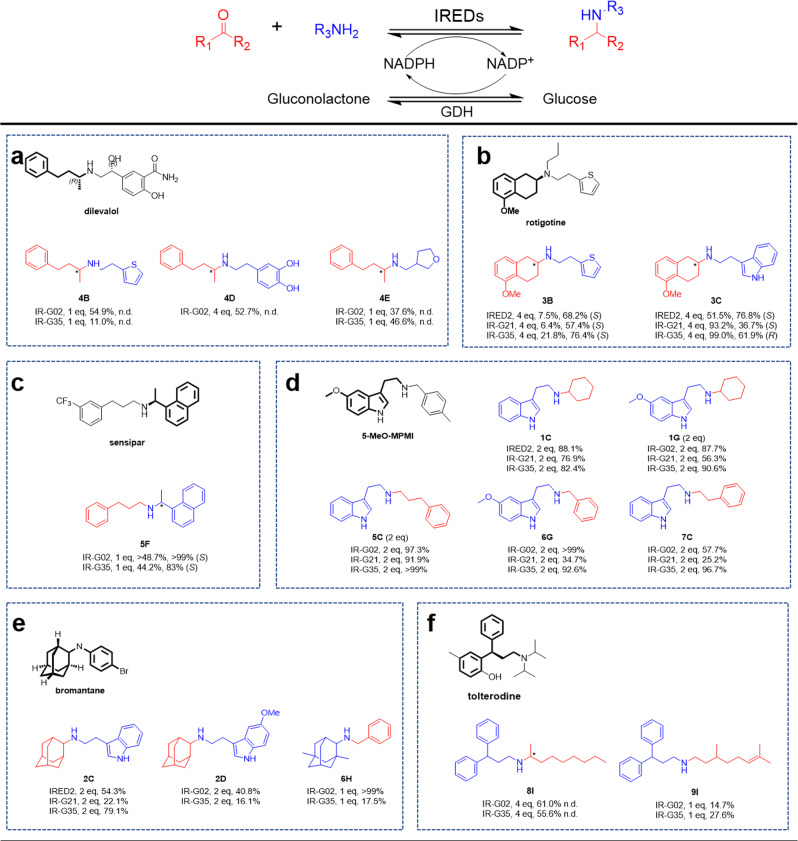
Applying IREDs for analytical-scale synthesis of APIs′ substructures. **a** Reductive aminations of **4** with **B**, **D**, and **E**. **b** Reductive aminations of **3** with **B** and **C**. **c** Reductive amination of **5** with **F**. **d** Reductive aminations of **1**, **5**, **6**, and **7** with **C** and **G**. **e** Reductive aminations of **2** and **6** with **C**, **D**, and **H**. **f** Reductive aminations of **8** and **9** with **I**.

4-phenyl-2-butanamines are often found in APIs, such as the antihypertension agent dilevalol^21^ in Fig. 3a. IR-G02 exhibited high specific activity for **4** with dopamine **D** (53% conversion), which harbors a hydroxyl group that reduces the compatibility of substrates with IREDs, while the activities of IR-G21 and IR-G35 were significantly lower with the conversions below 5%. Bulkier amines **B** and **E** were also accepted as reacting partners with **4**, with moderate to high conversions.

#### Reactions of tetralone with bulky amines

Tetralone-derived amines are frequently found in APIs, such rotigotine (Fig. 3b and Supplementary Figs. 3 and 4), an agent for the treatment of Parkinson’s disease and restless syndrome^22^. Turner and coworkers have studied synthesis of rotigotine by IREDs-catalyzed reductive aminations, using 5-methoxy-2-tetralone as ketone donor and *n*-propylamine or thiophene-2-ethylamine as amine donor with stereoselectivities up to 92% (*S*) and 99% (*S*) respectively. In this study, the specific activities of IR-G02, 21, and 35 were measured towards **3** with **B**, and low to moderate conversions (6–22%) were obtained. Interestingly, when **3** was incubated with the larger amine **C**, significantly higher conversions could be observed, and particularly, IR-G21 and IR-G35 all give >90% conversions, highlighting the contribution of the amine partner to the catalytic activities.

#### Reactions to access bulky N-alkylated benzylamines

*N*-alkylated benzylamines are also frequently found in APIs, such as sensipar (Fig. 3c and Supplementary Fig. 5), a treatment for secondary hyperparathyroidism^23^. Given aromatic conjugated ketones being challenged substrates for IREDs^20,24^, we applied kinetic resolution to synthesize bulky *N*-alkylated benzylamines. 3-phenylpropionaldehyde **5** and racemic 1-(1-Naphthyl)ethylamine **F** were used for reductive amination, and >44% conversions were observed for IR-G02 and IR-G35 with excellent kinetic resolution >99% (*S*) and >83% (*S*), respectively.

#### Reactions with bulky tryptamine and 5-methoxytryptamine

Tryptamine **C** and 5-methoxytryptamine **G** are building blocks for clinically important 5-HT agonists and therapeutics^25^, such as the psychedelic drug 5-MeO-MPMI (Fig. 3d). A series of reactions were performed towards **C** and **G** against a panel of carbonyls, including **1**, **5**, benzaldehyde **6**, and 2-phenylacetaldehyde **7**. In many cases, reactions afforded moderate to excellent conversions. Of significance, IRG-02 exerts high conversions (>55%) in all reactions.

#### Reactions with polycyclic adamantine derivatives

We used the adamantine derivatives to probe the preference of IR-G02, 21, and 35 towards polycyclic structures. 2-adamantanone **2** and memantine **H** have not been reported to be accepted by IREDs, which could be used to synthesize agents for viral infections and Alzheimer’s disease, such as bromantane^26^ in Fig. 3e. The synthesis of **2** **C**, **2D**, and **6H** using IR-G02 gave conversions of 54%, 41%, and >99%, respectively. IR-G35 also showed high conversion (79%) for the reductive amination of **2** and **D**. Both **2** and **H** could be well transformed, which represents an important extension of the substrate scope of IREDs.

#### Reactions with 3,3-diphenylpropylamine

We were further interested in the reductive aminations towards more challenging substrates, for instance, 3,3-diphenylpropylamine **I**. IR-G02 gave 61 and 15% conversions for the reductive aminations of **L** with two chain aldehydes (**8** and **9**), while IR-G35 showed 56 and 28% conversions for those two reductive aminations (Fig. 3f).

Overall, moderate (20–60%) or high conversions (>60%) were observed for most reactions in Fig. 3, and all bulky amines of **B**-**I** could be well transformed. IR-G02 showed unusually broad substrate tolerance with >50% conversions for 11 products in Fig. 3. Thus, we further expanded the test scope for IR-G02 with a variety of first-time-tested substrates (Supplementary Figs. 6 and 7). Supplementary Fig. 8 and Supplementary Tables 2 and 3 displays all reactions performed with an equimolar ratio of carbonyls and amines, using the mass-ion abundance of the corresponded products (determined by LC-MS), which indicates i) 14 first-time-tested bulky amines (**B**–**O**) could be efficiently transformed with certain carbonyl donors; ii) over 135 reductive-amination products could be synthesized with considerable conversions (each of their ion abundances is more than 5 × 10^6^); iii) aliphatic aldehydes, such as **5**, **6**, **9**, and **18**, are among the most active carbonyl donors. Overall, this work significantly expanded the substrate scope of IREDs.

### Structural studies of IR-G02

**Fig. 4:**
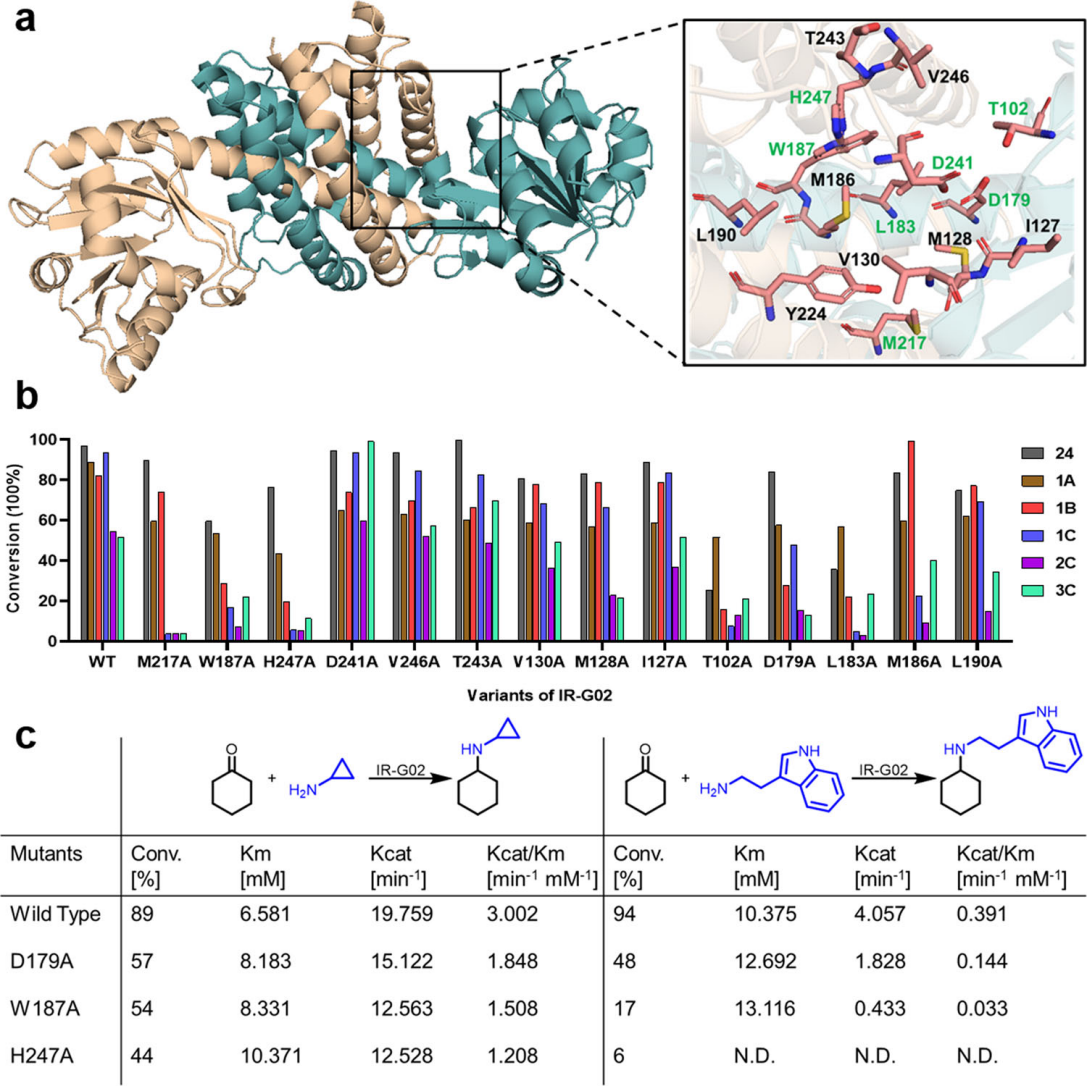
Structural and mutagenesis data of IR-G02. **a** *Apo*- structure of IR-G02 (PDB: 7XE8, Supplementary Data 1) and the residues inside the active pocket at the interface. **b** Analysis of the conversions of the imine reductions and reductive aminations with wild-type and mutants of IR-G02. **c** Kinetic data of IR-G02 wild-type and mutants W187A, H247A, and M217A for ketone **1**. N.D. was not determined because of the low activity.

**Fig. 5:**
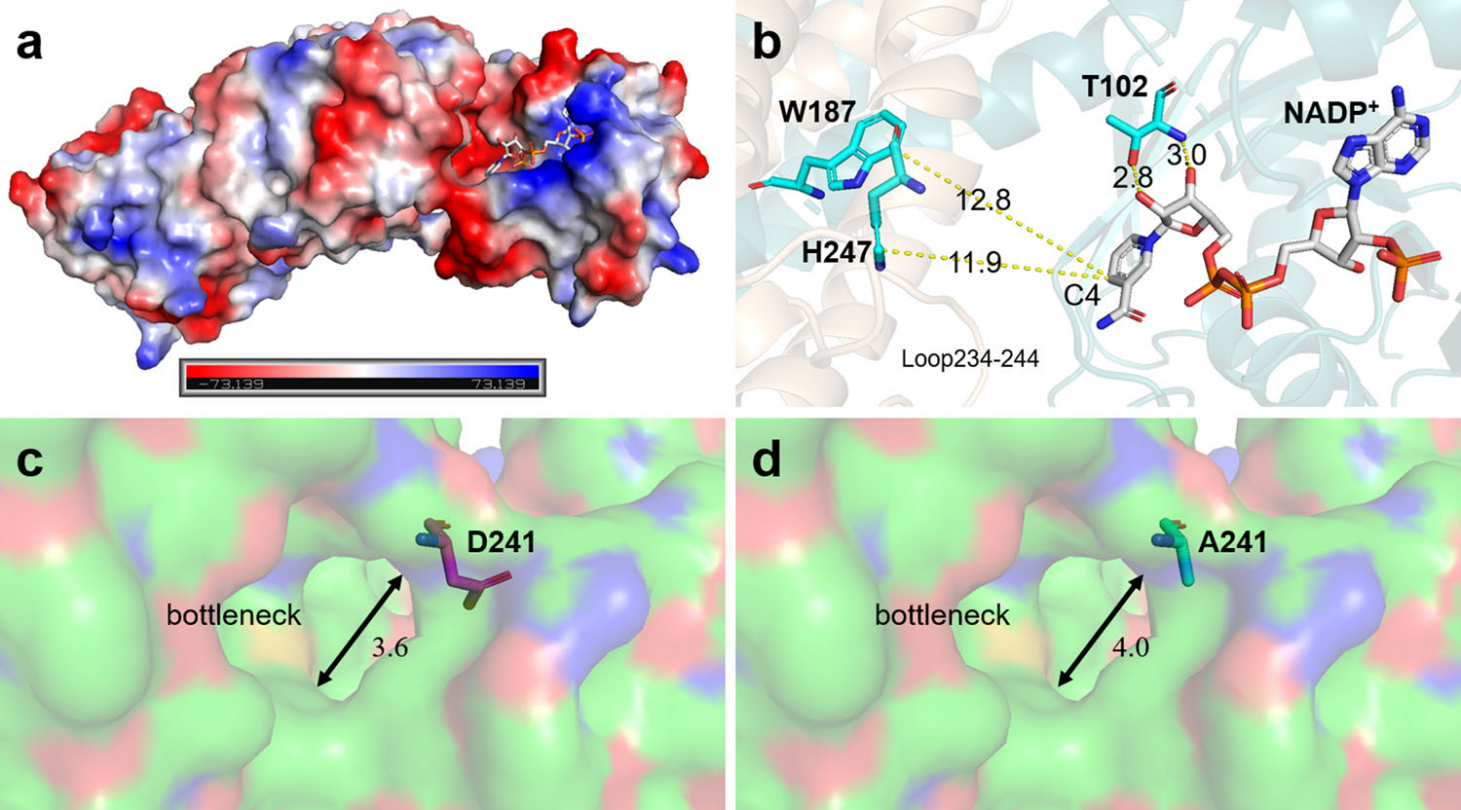
Structural analysis for the active-site pocket of IR-G02. **a** IR-G02 showed a strong negative electrostatic surface potential in the active-site pocket. **b** Active site of IR-G02 in complex with NADP^+^ (PDB: 7XR5, Supplementary Data 2). The distance between the residues (T102, W187 and H247) and C4 of NADP^+^ is given in Ångstroms. **c**, **d** Analysis of the side opening of WT and mutant D241A of IR-G02. The diameters of the bottleneck are given in ångströms.

To investigate the structural basis for observed prominent activities of IR-G02, the *apo-*structure (PDB ID: 7XE8) and NADP^+^ complex of IR-G02 (PDB ID: 7XR5) were obtained (Figs. 4a and 5a). However, several attempts to crystallize the ternary complex with NADP^+^ and (1-methyl-3, 4-dihydroisoquinoline **24**), **5**, or **F** were unsuccessful. This is consistent with the isothermal titration calorimetry (ITC) results showing no significant reaction heat or saturation curve detected for interactions between IR-G02 and the substrates above (Supplementary Fig. 9). The *apo*- structure of IR-G02 shared the similar dimeric structure of *Asp*RedAm^7^. It is a dimer with the active site forms at the interface (Fig. 4a). Each of the monomers was made up of an *N*-terminal Rossman domain and a *C*-terminal helical bundle connected by a long inter-domain α-helix. Except for the NADPH binding site, the pocket cavity of IR-G02 showed a strong negative electrostatic surface potential (Fig. 5a), which may favor the entry of positively charged amine donors and imine intermediate^27^, and contribute to its high substrate promiscuity.

Previous reports described three IREDs that *Ao*IRED, *Bc*IRED and *Asp*RedAm switching to a closed conformation upon cofactor binding, characterized by a relative angle-closure between the *N*-terminal Rossmann and the *C*-terminal helical domain^7,28,29^, while such movements were not found in IR-G02-NADP^+^ complex. Superimposition of *apo*-IR-G02 and IR-G02-NADP^+^ complex shows an overall rmsd of 2.665 Å. There are substantial changes in the Rossman fold: most secondary structures shift with the binding of NADP^+^, and the side chain of T102 flips with its hydroxy group facing the NADP^+^ and forming a hydrogen bond with the ribose moiety of NADP^+^. The homologous site of the residue S260 in IR-G36 is occupied by A242 in IR-G02 and no interaction is found between the loop 234–244 and NADP^+^, indicating IR-G02 is “wide” type IRED which possesses relative wide catalytic cavity for bulky-substrate entry and product release^19^.

IR-G02 showed a 37% sequence identity with *Asp*RedAm. Cavity-volume calculation using CASTp^30^ suggested the cavity volume of IR-G02-NADP^+^ (Supplementary Method 3) complex is 1.9-fold of that of *Asp*RedAm (Supplementary Fig. 10), which also rationalizes its better substrate tolerance^31–38^. This is also consistent with our hypothesis that IREDs obtained through the increasing-target-volume screening may possess large substrate-binding pockets.

### Site-directed mutagenesis of IR-G02

In order to investigate the key residues in the active site, and the residues associated with the bulky substrate tolerance, site-directed mutagenesis was performed with 14 selected residues in the active site (Fig. 4a). Three residues Y177, D169, and N93 have been proposed as the catalytic triad in *Asp*RedAm^39^. The homologous site of W187 was Y177 in *Asp*RedAm, which was proposed to be essential for protonation and anchoring of the carbonyls^39^. The weak protic W187 is incapable of being a proton shuttle, and a nearby protonic residue, most likely H247 could take the place of W187. Analysis of the IR-G02-NADP^+^ complex suggested the side chain of H247 is positioned approximately 11.9 Å from the NADPH C4 atom that delivers and accepts hydride (Fig. 4b and Supplementary Table 4), whereas the distance from W187 to NADPH C4 atom is 12.8 Å. The increased *K*_m_ values of mutants W187A and H247A for ketone **1** support the possible roles of two residues in ketone binding (Fig. 4c). Meanwhile, mutants W187A and H247A showed significant decreases in conversions for all reductive aminations (Fig. 4b and Supplementary Table 4).

The residue D179, which occupies the position equivalent to D169 in *Asp*RedAm, is proposed to be responsible for deprotonating the amine substrate^39^. The mutant D179A shows no apparent change in the imine reduction activity but a significant decrease in all reductive amination conversions (13–75%) and higher *K*m values for ketone 1, suggestive of a significant role in reductive amination.

The homologue of T102 has been reported to be the amine donor binding and cofactor sites^39,40^. Notably, the mutant T102A showed significantly decreased conversions toward all substrates (Fig. 4b and Supplementary Table 4). Protein structure analysis suggested that T102 was located at the Rossmann fold domain with its side chain approximately 2.8–3.0 Å from the 2′ and 3′-ribose hydroxyls of NADPH, which is responsible for recognizing and binding NADPH (Fig. 5b). Indeed, ITC results suggested that T102A showed a 43% decrease in the affinity to NADPH (Kd values: 2.69E4 M^−1^ (Wild Type); 1.88E5 M^−1^ (T102A); Supplementary Fig. 11).

Interestingly, 10 and 92% improvements were observed for D240A toward the synthesis of **2** **C** and **3** **C** (Fig. 4b and Supplementary Table 4), respectively. Tunnel analysis using Caver^41^ suggested D241 is located at the flexible loop A234-L244 on the top of the active site, which forms a bottleneck (about 3.6 Å) imposing steric constraints for the entrance of the bulky substrates (Fig. 5c and Supplementary Fig. 12). The structure of mutant D241A has been modeled, and indeed, the bottleneck was suggested to be improved to 4.0 Å (Fig. 5d), which is consistent with our recent results that the catalytic efficiency can be improved by expanding the side opening^19^.

M217 is located on the side opening of the substrate tunnel, which may affect the substrate entrance. Mutant M217A displayed high activities for the imine reduction of **24** or the synthesis of **1** **A** and **1B**, but almost lost the activities for the synthesis of bulky **1** **C**, **2** **C**, and **3** **C** with 93–96% decreases of conversions (Fig. 4b and Supplementary Table 4), suggesting a role of M217 in bulky substrates tolerance. The mutations of three hydrophobic residues L183, M186, and L190 on the long α-helix to alanine showed different reductions of activities towards substrates (Fig. 4b and Supplementary Table 4). Especially, the activities of mutant L183A showed considerable decreases for all reactions, suggesting that the residue L183 located on the inter-domain α-helix may provide the necessary hydrophobic force or steric hindrance to ensure the catalytic conformation of the substrate in the active pocket. Ala scanning mutagenesis on the rest of the residues in the active site gave no obvious differences in all activities.

Overall, our work revealed a similar reductive-amination mechanism of IR-G02 to *Asp*RedAm, and the intensely negative potential of the substrate-binding site and relative wide catalytic cavity may contribute to the good bulky amine compatibility in IR-G02.

### Preparative-scale synthesis of *N*-alkylated benzylamine *via* kinetic resolution

To demonstrate the practical utility of IR-G02, we set up to preparative scale synthesis of *N*-alkylated benzylamine (*S*)-**5F**, which is an analogue of sensipar. The pH optimum of IR-G02 was found to be 7.0, and the temperature optimum was 30 °C (Supplementary Table 5). The unfolding nature was studied by recording CD_222_ at different temperatures (20–80 °C), which indicated the melting temperature of IR-G 02 was found to be 52 °C with 8 degrees higher than that of *Asp*RedAm (Supplementary Fig. 13)^10^. The concentration of ketone, the number of amine equivalents, and the enzyme loading were also comprehensively studied. Kinetic parameters were determined with a *K*_m_ value of 6.464 mM, *K*_cat_ value of 0.606 s^−1^, and *K*_cat_/*K*_m_ value of 0.094 s^−1^ mM^−1^. Good conversion (>48%) could be achieved using an equimolar ratio of 20 mM **5** and racemic **F** with 0.15 mg mL^−1^ IR-G02. Therefore, a preparative scale reaction (250 mL reaction volume) was performed using **5** (670.85 mg, 5 mmol) and equal amine **F** (856.20 mg, 5 mmol) (Supplementary Method 4 and Fig. 14), which were converted into (*S*)-**5F** with high enantioselectivity (>99% ee) and 48% conversion. Eventually, (*S*)-**5F** was produced in 39% isolated yield, with total turnover numbers (TTNs) up to 2087, and space time yield (STY) up to 18.10 g L^−1^ d^−1^.

## Conclusion

In addition to the wider tunnel and negative potential of the enzyme cavity highlighted here, the characteristics of the substrates include polarity, the *pK*a of the amine donors, and steric hindrance around the reacting amine or carbonyl groups, etc., which could also affect the activities of IREDs. Thus, various IREDs with dedicated substrate preferences and complementary enantioselectivities towards diverse amine donors are desired, but not yet reported. The ones presented here represent an ideal starting point to further broaden the chemist’s biocatalyst toolbox for chiral amine synthesis. Also, IR-G02, 21, and 35 have been evaluated for the synthesis of various APIs. For example, IR-G35 showed moderate enantioselectivity (>77%) in the synthesis of a rotigotine substructure. Future protein engineering will be the method of choice to generate more efficient and stereospecific mutants of IR-G02, 21, and 35.

The IREDs identified here by the Increasing-Molecule-Volume-Screening showed good tolerance of 14 first-time-tested bulky amines. IR-G02 exhibits an excellent substrate scope that enables it to synthesize more than 135 secondary and tertiary amines with considerable conversions. Finally, a gram-scale and highly-enantioselective synthesis (>99%) of sensipar analogue (*S*)-5F was achieved with IR-G02 through the kinetic resolution approach, by using only a stoichiometric amount of bulky amine 1-(1-Naphthyl)ethylamine.

In summary, the broad tolerance to bulky amines of IREDs with low stoichiometry provides a biological route to access medicinal chiral amines. Our work will stimulate further research, as many questions are still open, including how to further engineer IR-G02, 21, and 35 for favoring polar and hindered substrates. Due to the hydrophobic nature of the active site in IREDs^7,18^, characterized IREDs tend to accept small and hydrophobic substrates^7,18^. Thus, one challenge regarding the industrial application of IREDs is the low preference toward polar substrates, including carboxylic-acid or hydroxyl-substituted ones. IR-G02 shows activities towards **D**, **O**, **M**, and **22**, which are only cases for the enzyme-catalyzed reductive aminations towards substrates harboring hydroxyl groups to the best of our knowledge. Hence, more efforts for identifying and engineering IREDs with substrate preference toward bulky substrates remains to be done.

## Methods

### General

LC-MS analysis was performed on the AGILENT-1100HPLC/G1946D MSD (Agilent Technologies Inc., California, USA) system using C18 analytical column (Ultimate XB-C18, 2.1 × 100 mm, 3 μm). Chiral HPLC analysis was performed on Shimadzu LC-2030C 3D plus (Shimadzu, Kyoto, Japan) or Waters 2695 (Waters, Milford, USA) using chiral analytical columns (CHIRALPAK IA/OD-H/AY-H, 4.6 × 250 mm, 5 μm). 1D and 2D NMR spectra were recorded on a Bruker Avance 500 MHz spectrometer. Size-exclusion chromatography was carried out with Sephadex LH-20 column (GE Healthcare, Chicago, IL, USA). Column chromatography was performed on silica gel (200–300 mesh, Qingdao Marine Ltd., Qingdao, China). Commercially available chemicals and reagents were purchased from Meryer (Shanghai, China), Macklin (Shanghai, China), 3AChem (Shanghai, China), Sigma-Aldrich (Poole, Dorset, UK), or Alfa Aesar (Karlsruhe, Germany) unless stated otherwise. HPLC solvents were obtained from Thermo Fisher Scientific (Waltham, Massachusetts, USA).

### Reductive amination procedure for the preparation of amines

A dry methanol (10 mL) solution of the ketone or aldehyde (2.0 mmol), the corresponding amine (3.0 mmol), and acetic acid (300 μL) under an N_2_ atm was stirred for 1.5 h at r.t. The reaction was placed in an ice-H_2_O bath and sodium cyanoborohydride (0.377 g, 6.0 mmol) was added. The reaction mixture gradually warmed to r.t. and stirred overnight. The reaction progress was monitored by TLC and following completion was quenched with sat. NaHCO_3_ solution (10 mL) and stirred for an additional 30 min. The mixture was extracted with EtOAc (3 × 10 mL) and the combined organic phase dried over Na_2_SO_4_ and concentrated under reduced pressure. Column chromatography on silica affords the corresponding racemic amine. The NMR data are provided in Supplementary Note 1 and Supplementary Data 4.

### Expression and purification of IREDs

The plasmids containing the genes for target enzymes were used to transform *E. coli* BL21(DE3) competent cells for gene expression. Pre-cultures were grown in LB-medium (10 mL) containing 30 µg mL^−1^ kanamycin for 12 h at 37 °C with shaking at 220 r.p.m. 1 L volume cultures were inoculated with the pre-culture (10 mL) and incubated at 37 °C, with shaking at 220 r.p.m. until an OD_600_ of 0.6~0.8 was reached. Gene expression was induced by the addition of IPTG (0.2 mM) and shaking was continued overnight at 16 °C with shaking at 160 r.p.m. The cells were then harvested by centrifugation at 5000 g for 30 min and resuspended in 50 mM Tris-HCl buffer pH 7.5, containing 300 mM NaCl. Cells were disrupted by ultrasonication for 10 min, 5 s on, 9 s off cycles, and the suspension was centrifuged at 50,000 g for 30 min to yield a clear lysate.

The N-terminal His-tagged proteins were purified using the Ni-NTA column. In each case, the lysate was loaded onto a pre-equilibrated Ni-NTA column, followed by washing with a load buffer (50 mM Tris-HCl, 300 mM NaCl, 40 mM imidazole pH 7.5). The bound protein was eluted with buffer containing 250 mM imidazole. Proteins were concentrated and used for biotransformation reactions.

### Protein purification and crystallisation

The amino acid and DNA sequences are available in Supplementary Note 2. The N-terminal His_6_-tagged proteins were purified using Ni-NTA column, followed by size exclusion chromatography (SEC) (Supplementary Fig. 15). In each case, the lysate was loaded onto a pre-equilibrated Ni-NTA column, followed by washing with a load buffer (50 mM Tris-HCl, 300 mM NaCl, 20 mM imidazole pH 7.5). The bound protein was eluted using a linear gradient with buffer containing 60~250 mM imidazole. IR-G02 fractions were pooled, concentrated, and loaded onto a GL 10/300 Superdex 200 gel filtration column pre-equilibrated with 50 mM Tris-HCl, 300 mM NaCl pH 7.5 buffer. The concentrated protein sample after gel filtration was used for crystallization screening.

IR-G02 was crystallized by the sitting-drop vapour diffusion method at 18 °C with 1 μL protein solution mixed with 1 μL reservoir buffer. High-quality crystals (Supplementary Fig. 15) of IR-G02 grew in the buffer containing 0.2 M ammonium acetate, 0.1 M Bis-Tris 5.5, 25 % w/v PEG 3350 with a protein concentration of 20 mg ml^−1^.

IR-G02-NADP^+^ complex was also crystallized by the sitting-drop vapour diffusion method at 18 °C with 1 μL protein solution mixed with 1 μL reservoir buffer. High-quality crystals (Supplementary Fig. 15) of IR-G02-NADP^+^ complex grew in the buffer containing 0.1 M Bis-Tris pH 7.5, 25% PEG 4000 and 0.25 M NaCl with a protein concentration of 20 mg ml^−1^ which was incubation with NADP^+^ (10 eq) for 2 h.

Diffraction data were collected with cryoprotected (in a reservoir solution containing 20% [v/v] glycerol) crystals at the Shanghai Synchrotron Radiation Facility beamline BL17U. All the datasets were processed with XDS and aimless. The structure of IR-G02 was determined by the molecular replacement method in Phenix. The atomic model was completed with Coot and refined with Phenix.refine in Phenix, and the stereochemical quality of the final model was assessed with Molprobity. Data collection, processing and refinement statistics are summarized in Supplementary Table 6.

### Michaelis-Menten kinetic analysis

The kinetics measurements of purified wild type and variants of IR-G02 for substrates were performed at 30 °C in potassium phosphate buffer (100 mM, pH 7.0) at various concentration ranges of ketones (0.1–50 mM). Kinetic constants were determined through non-linear regression based on Michaelis-Menten kinetics using GraphPad Prism7 software.

### Isothermal titration calorimetry

ITC experiments were performed in a Microcal ITC200 microcalorimeter at 25 °C in 100 mM PBS buffer (pH 7.4). Wild type, variant T102A of IR-G02 and the ligands (NADPH, NADP^+^, 1-(1-naphthyl)ethylamine, 3-phenylpropionaldehyde, and 1-methyl-3, 4-dihydroisoquinoline) were dialyzed into the PBS buffer containing 2% DMSO. All experiments were performed with proteins (wild type or variant T102A of IR-G02) in the microcalorimeter cell at 40 μM concentration, and ligands in the syringe at 600 μM concentration. The titrations (total 40 min) consisted of a total of 20 injections, spaced 120 s apart. Data were analyzed in ORIGIN using a one-site binding model with fixed *n* = 1 per B subunit (the fixed-parameter was required to achieve convergence of the fit).

### Thermal denaturation studies

CD thermal unfolding measurements were performed using a Jasco J810 CD Spectrophotometer fitted with a computer-controlled Peltier temperature control unit, using protein solutions at 0.25 mg mL^−1^ in 100 mM NaPi pH 7.0 containing 1 mM NADPH cofactor. The protein solution was heated through a 5 °C ramp with a 5 min relaxation time between the recording of CD spectra at different temperatures from 20 to 80 °C. The unfolding curves were built by using the CD signal at 220 nm to obtain melting temperature values.

### Reporting summary

Further information on research design is available in the Nature Research Reporting Summary linked to this article.

## Supplementary information

Supplementary information

Description of Additional Supplementary Files

Supplementary Data 1

Supplementary Data 2

Supplementary Data 3

Supplementary Data 4

Reporting Summary

## Data Availability

Supplementary Method, Notes, Tables, and Figures are available in the Supplementary Information. The crystal structure of *apo*-IR-G02 and NADP^+^ complex of IR-G02 have been deposited in the PDB with coordinate accession numbers 7XE8 and 7XR5, respectively, at http://www.rcsb.org, and both PDB files are provided in Supplementary Data 1 and 2. LC-MS chromatograms for IREDs-catalyzed reductive aminations are submitted as Supplementary Data 3. NMR spectra of all amine products are available in the Supplementary Data 4.
