# Supplementary material for: Actinomycetes-derived imine reductases with a preference towards bulky amine substrates
Source: Commun Chem. 2022 Oct 8;5:123. doi: 10.1038/s42004-022-00743-y (PMC9814587; doi:10.1038/s42004-022-00743-y)
Supplement: Supplementary file 1 — Supplementary information [file 42004_2022_743_MOESM1_ESM.pdf]

Actinomycetes-derived imine reductases with a preference towards bulky amine substrates

Jun Zhang[1,2,8], Xin Li[3,8], Rongchang Chen[4,8], Xianwei Tan[5], Xiongduo Liu[5], Yaqing Ma[1,5], Fangfang Zhu[5], Chunyan An[5], Guangzheng Wei[5], Yongpeng Yao[5], Lujia Yang[1,6], Peng Zhang[7], Qiaqing Wu[1,6], Zhoutong Sun[1,6], Bin-Gui Wang,[3*] Shu-Shan Gao,[1,6*] Chengsen Cui[1,6*]

[1]Tianjin Institute of Industrial Biotechnology, Chinese Academy of Sciences, Tianjin 300308, China

[2]School of Life Science, Hebei University, Baoding 071002, China

[3]Key Laboratory of Experimental Marine Biology, Institute of Oceanology, Chinese Academy of Sciences, Qingdao 266071, China

[4]ReadCrystal Bio-tech Co. LTD, Suzhou 215505, China

[5]State Key Laboratory of Microbial Resources, Institute of Microbiology, Chinese Academy of Sciences, Beijing 100101, China

[6]National Technology Innovation Center of Synthetic Biology, Tianjin 300308, China

[7]Tobacco Research Institute of Chinese Academy of Agricultural Sciences, Qingdao 266101, China

[8]These authors contributed equally: Jun Zhang, Xin Li, Rongchang Chen.

Email: wangbg@ms.qdio.ac.cn; gaoss@im.ac.cn; cuichs@tib.cas.cn

Table of contents

Supplementary Methods 3

Supplementary Method 1: Biotransformations 3

Supplementary Method 2: LC-MS and chiral HPLC analysis 3

Supplementary Method 3: Cavity and tunnel analysis..4

1. Tunnel analysis 4

2. Cavity volume site analysis 4

Supplementary Method 4: IR-G02-catalyzed preparative scale biotransformation 4

Supplementary Note 1: NMR data of amine products 5

Supplementary Note 2: Amino acid and DNA sequences of IR-G02 10

Supplementary Tables...12

Supplementary Figures 19

Supplementary Reference 26

Supplementary Methods

Supplementary Method 1: Biotransformations

Biotransformations for reductive amination of ketones (**1-3**) and amines (**A-C**) were performed with purified IREDs (85 IREDs and *Asp*RedAm). A typical 50 μL reaction mixture contained 1 mM $NADP^+$, 1.0 mg mL^{-1} IREDs, 5 mM ketone, 5mM amine, 30 mM *D*-glucose, 0.2 mg mL^{-1} GDH and 5% DMSO. The reaction volume was made up to 50 μL with sodium phosphate buffer (100 mM, pH 7.0).

Biotransformations for reductive amination of carbonyls/ketones (**1-23**) and amines (**A-R**) were performed with purified IR-G02, IR-G21, and IR-G35. A typical 50 μL reaction mixture contained 1 mM $NADP^+$, 1.0 mg mL^{-1} enzyme, 5 mM ketone, 1-4 eq amine, 30 mM D-glucose, 0.2 mg mL^{-1} GDH and 5% DMSO. The reaction volume was made up to 50 μL with sodium phosphate buffer (100 mM, pH 7.0).

Biotransformations for imine reduction of imine **24** were performed with purified wild type and variants of IR-G02. A typical 50 μL reaction mixture contained 1 mM $NADP^+$, 1.0 mg mL^{-1} enzyme, 5 mM imine **24**, 30 mM D-glucose, 0.2 mg mL^{-1} GDH and 5% DMSO. The reaction volume was made up to 50 μL with sodium phosphate buffer (100 mM, pH 7.0).

All the reactions were incubated at 30 °C with shaking at 250 rpm for 24 h, after which they were quenched by the addition of 100 uL MeOH. The mixture was centrifuged at 12000 rpm, 8 min. The supernatant was analyzed on LC-MS.

Reactions for enantiomeric excess tests were performed with a reaction mixture (500 μL) at the same conditions above. They were quenched by the addition of 200 μL of $NH_3 \cdot H_2O$ and extracted with 500 μL ethyl acetate. The organic fraction dried under reduced pressure and analyzed on chiral HPLC.

Supplementary Method 2: LC-MS and chiral HPLC analysis

All biotransformation products were analyzed by LC-MS with gradient method (0.3 mL min^{-1}, 18 min, H_2O/MeCN, 90/10→0/100, v/v) using C-18 column (Ultimate XB-C18, 2.1×100 mm, 3 μm). The products were confirmed by UV spectrum comparison with standards or extracted the positive

ion of products $[M+H]^+$. Standard curves plotted for varying concentrations of products at a UV detection wavelength of 210 nm or extractions. Conversions were calculated according to the standard curves. The chiral products were analyzed by chiral HPLC with isocratic methods with different solvent ratios of *n*-hexane and ethanol.

Supplementary Method 3: Cavity and tunnel analysis

1. Tunnel analysis

The tunnels of the wild type, D241A and D241G of IR-G02 were analyzed by CAVER Analyst 2.0[1]. The tunnel starting point was the geometric center of the active site (D241).

2. Cavity volume site analysis

The cavity volumes were calculated and identified by CASTp with 1.4 Angstroms probe radius[2]. The cavity volumes of IR-G02 and *Asp*RedAm were defined by using the *apo*- crystal structure.

Supplementary Method 4: IR-G02-catalyzed preparative scale biotransformation

Preparative-scale reactions of **5** and **F** were performed using IR-G02. The pH of PBS buffers, the concentration of ketone and amine, and process temperatures were investigated (Supplementary Table 8) to optimize the biotransformation parameters. Preparative scale reductive amination reactions (250 mL reaction volume) contained with **5** (670.85 mg, 20 mmol), equal amine **F** (856.20 mg, 20 mmol), 2 mM $NADP^+$, 30 mM *D*-glucose, 0.5 mg mL^{-1} GDH and 10% DMSO, and was stirred for 24 h at 30 °C.

Supplementary Note 1: NMR data of amine products

***N*-cyclopropylcyclohexanamine, 1A**

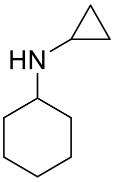

Colourless oil isolated. ^{1}H NMR δ_H (500 MHz, CD_3OD) 3.13 (m, 1H), 2.68 (m, 1H), 2.14 (m, 2H), 1.83 (m, 2H), 1.67 (m, 1H), 1.25-1.42 (4H), 1.21 (m, 1H), 0.85 (m, 2H), 0.75 (m, 2H). ^{13}C NMR δ_C (125 MHz, CD_3OD) 59.5, 30.8, 28.6, 26.1, 25.4, 4.3.

***N*-(2-(thiophen-2-yl)ethyl)cyclohexanamine, 1B**

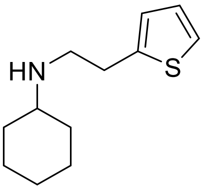

Colourless oil isolated. ^{1}H NMR δ_H (500 MHz, CD_3OD) 7.29 (m, 1H), 6.95-6.98 (2H), 3.20-3.30 (4H), 3.08 (m, 1H), 2.10 (m, 2H), 1.87 (m, 2H), 1.70 (d, J = 12.8Hz, 1H), 1.28-1.43 (4H), 1.24 (m, 1H). ^{13}C NMR δ_C (125 MHz, CD_3OD) 139.6, 128.3, 127.2, 125.6, 58.4, 46.8, 30.3, 27.6, 26.1, 25.5.

***N*-(2-(1H-indol-3-yl)ethyl)cyclohexanamine, 1C**

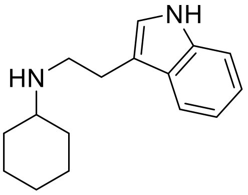

Colourless oil isolated. ^{1}H NMR δ_H (500 MHz, CD_3OD) 7.55 (d, J = 8.0 Hz, 1H), 7.35 (d, J = 8.1 Hz, 1H), 7.17 (s, 1H), 7.10 (t, J = 7.6 Hz, 1H), 7.03 (t, J = 7.5 Hz, 1H), 3.28 (m, 2H), 3.12 (m, 2H), 3.05 (m, 1H), 2.06 (m, 2H), 1.83 (m, 2H), 1.67 (d, J = 12.7 Hz, 1H), 1.26–1.37 (4H), 1.18 (m, 1H). ^{13}C NMR δ_C (125 MHz, CD_3OD) 138.3, 128.1, 124.2, 122.7, 120.1, 118.9, 112.5, 110.3, 58.3, 46.1, 30.3, 26.1, 25.5, 23.5.

***N*-(2-(5-methoxy-1H-indol-3-yl)ethyl)cyclohexanamine, 1G**

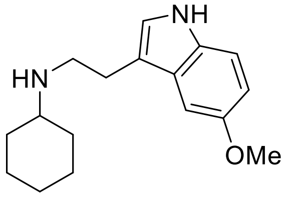

Colourless oil isolated. ^{1}H NMR δ_H (500 MHz, CD_3OD) 7.21 (d, J = 8.8 Hz, 1H), 7.09 (s, 1H), 7.01 (d, J = 2.4 Hz, 1H), 6.74 (dd, J = 8.8, 2.4 Hz, 1H), 3.77 (s, 3H), 3.19 (dd, J = 9.0, 6.6 Hz, 2H), 3.05 (dd, J = 9.0, 6.6 Hz, 2H), 2.97 (tt, J = 11.3, 4.1 Hz, 1H), 2.01 (m, 2H), 1.77 (m, 2H), 1.61 (m, 1H), 1.29 (m, 2H), 1.24 (m, 2H), 1.13 (m, 1H). ^{13}C NMR δ_C (125 MHz, CD_3OD) 155.2, 133.4, 128.5, 124.9, 113.3, 112.9, 110.1, 100.9, 58.3, 56.3, 46.0, 30.2, 26.0, 25.4, 23.4.

***N*-(2-(1H-indol-3-yl)ethyl)adamantan-2-amine, 2C**

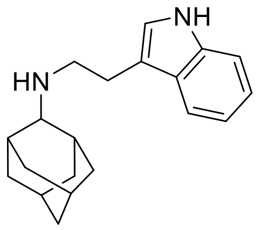

Colourless oil isolated. ^{1}H NMR δ_H (500 MHz, CD_3OD) 7.58 (dt, J = 8.0, 1.1 Hz, 1H), 7.37 (dt, J = 8.0, 1.1 Hz, 1H), 7.19 (s, 1H), 7.13 (ddd, J = 8.0, 7.0, 1.1 Hz, 1H), 7.05 (ddd, J = 8.0, 7.0, 1.1 Hz, 1H), 3.41 (s, 1H), 3.35 (s, 1H), 3.33 (m, 1H), 3.21 (m, 2H), 2.15 (m, 2H), 2.00–1.87 (m, 6H), 1.83–1.70 (m, 6H). ^{13}C NMR δ_C (125 MHz, CD_3OD) 138.3, 128.1, 124.1, 122.8, 120.1, 118.9, 112.6, 110.5, 63.9, 47.1, 38.0, 37.8, 31.1, 30.7, 28.4, 28.1, 23.3.

***N*-(2-(5-methoxy-1*H*-indol-3-yl)ethyl)adamantan-2-amine, 2D**

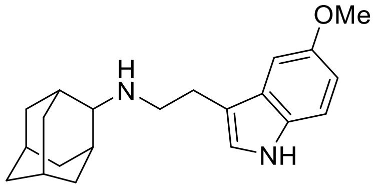

Colourless oil isolated. ^{1}H NMR δ_H (500 MHz, CD_3OD) 7.24 (d, J = 8.8 Hz, 1H), 7.15 (s, 1H), 7.05 (d, J = 2.4 Hz, 1H), 6.78 (dd, J = 8.8, 2.4 Hz, 1H), 3.40 (m, 1H), 3.29 (m, 2H), 3.16 (m, 2H), 2.13 (m, 2H), 1.84–1.97 (m, 6H), 1.67–1.81 (m, 6H). ^{13}C NMR δ_C (125 MHz, CD_3OD) 155.3, 133.4, 128.4, 124.8, 113.3, 113.0, 110.2, 100.9, 63.8, 56.3, 47.0, 38.0, 37.7, 31.1, 30.6, 28.3, 28.1, 23.1.

5-methoxy-*N*-(2-(thiophen-2-yl)ethyl)-1,2,3,4-tetrahydronaphthalen-2-amine, 3B

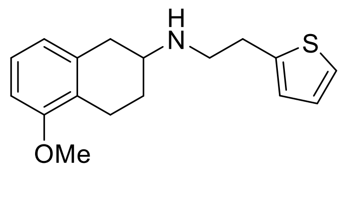

Colourless oil isolated. ^{1}H NMR δ_H (500 MHz, CD_3OD) 7.21 (dd, J = 5.2, 1.2 Hz, 1H), 7.05 (t, J = 8.0 Hz, 1H), 6.93 (dd, J = 5.2, 3.4 Hz, 1H), 6.88 (dd, J = 3.4, 1.2 Hz, 1H), 6.69 (d, J = 8.0 Hz, 1H),

6.66 (d, J = 8.0 Hz, 1H), 3.78 (s, 3H), 3.06 (m, 1H), 3.05 (d, J = 6.6 Hz, 1H), 2.99 (d, J = 6.6 Hz, 1H), 2.98 (m, 2H), 2.87 (m, 2H), 2.53 (m, 2H), 2.09 (m, 1H), 1.50 (dd, J = 12.6, 5.8 Hz, 1H). ^{13}C NMR δ_C (125 MHz, CD_3OD) 158.6, 143.2, 137.1, 127.9, 127.4, 126.2, 125.7, 124.6, 122.5, 108.2, 55.6, 54.4, 49.3, 36.9, 30.8, 29.7, 23.1.

***N*-(2-(1H-indol-3-yl)ethyl)-5-methoxy-1,2,3,4-tetrahydronaphthalen-2-amine, 3C**

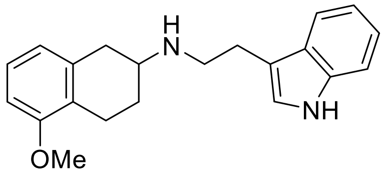

Colourless oil isolated. ^1H NMR δ_H (500 MHz, CD_3OD) 7.57 (dt, J = 8.1, 1.0 Hz, 1H), 7.34 (dt, J = 8.1, 1.0 Hz, 1H), 7.20 (t, J = 8.0 Hz, 1H), 7.01 (t, J = 8.0 Hz, 1H), 7.09 (ddd, J = 8.1, 7.0, 1.0 Hz, 1H), 7.07 (s, 1H), 6.67 (d, J = 8.1 Hz, 1H), 6.62 (d, J = 7.6 Hz, 1H), 3.76 (s, 3H), 2.98–3.05 (m, 4H), 2.95 (m, 1H), 2.83 (m, 2H), 2.51 (m, 2H), 2.05 (m, 1H), 1.49 (m 1H). ^{13}C NMR δ_C (125 MHz, CD_3OD) 158.5, 138.3, 137.1, 128.7, 127.4, 125.7, 123.5, 122.4, 122.4, 119.6, 119.2, 113.4, 112.3, 108.2, 55.6, 54.6, 48.1, 36.9, 29.7, 26.3, 23.1.

4-phenyl-*N*-(2-(thiophen-2-yl)ethyl)butan-2-amine, 4B

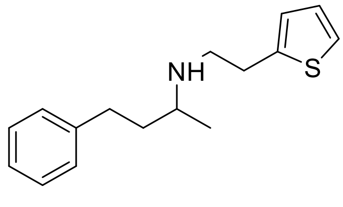

Colourless oil isolated. ^1H NMR δ_H (500 MHz, CD_3OD) 8.44 (s, 1H), 7.34–7.28 (m, 3H), 7.25 (m, 2H), 7.21 (m, 1H), 6.99 (m, 2H), 3.16–3.31 (m, 5H), 2.81 (ddd, J = 13.8, 9.9, 5.2 Hz, 1H), 2.67 (ddd, J = 13.8, 9.9, 6.8 Hz, 1H), 2.13 (dddd, J = 13.6, 10.3, 6.8, 3.7 Hz, 1H), 1.86 (dtd, J = 13.3, 9.7, 5.2 Hz, 1H), 1.40 (d, J = 6.5 Hz, 3H). ^{13}C NMR δ_C (125 MHz, CD_3OD) 141.7, 139.4, 129.7, 129.4, 128.3, 127.4, 127.3, 125.8, 55.4, 47.1, 35.9, 32.5, 27.6, 16.2.

4-(2-((4-phenylbutan-2-yl)amino)ethyl)benzene-1,2-diol, 4D

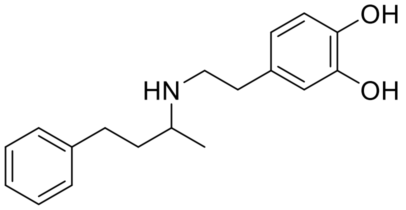

Colourless oil isolated. ^1H NMR δ_H (500 MHz, CD_3OD) 8.55 (s, 1H), 7.27–7.34 (m, 2H), 7.18–7.27

(m, 3H), 6.75 (d, J = 8.0 Hz, 1H), 6.71 (d, J = 2.0 Hz, 1H), 6.58 (dd, J = 8.0, 2.0 Hz, 1H), 3.23 (m, 1H), 3.17 (m, 2H), 2.75–2.87 (m, 3H), 2.66 (m, 1H), 2.11 (m, 1H), 1.84 (m, 1H), 1.39 (d, J = 6.4 Hz, 3H). ^{13}C NMR δ_C (125 MHz, CD_3OD) 146.8, 145.6, 141.7, 129.7, 129.4, 129.0, 127.4, 120.9, 116.8, 116.7, 55.2, 47.4, 35.9, 32.9, 32.5, 16.2.

4-phenyl-*N*-((tetrahydrofuran-3-yl)methyl)butan-2-amine, 4E

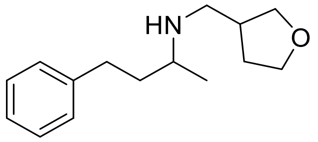

Colourless oil isolated. ^{1}H NMR δ_H (500 MHz, CD_3OD) 8.38 (m, 1H), 7.17–7.35 (m, 5H), 3.87 (m, 2H), 3.74 (q, J = 7.8 Hz, 1H), 3.51 (ddd, J = 8.8, 5.9, 2.0 Hz, 1H), 3.23 (m, 1H), 3.04 (m, 1H), 2.80 (ddd, J = 13.8, 10.1, 5.2 Hz, 1H), 2.65 (ddd, J = 13.8, 9.8, 6.8 Hz, 1H), 2.55 (m, 1H), 2.14 (m, 1H), 1.84 (m, 1H), 1.68 (ddt, J = 14.8, 7.9, 6.7 Hz, 1H), 1.40 (d, J = 6.6 Hz, 3H). ^{13}C NMR δ_C (125 MHz, CD_3OD) 141.7, 129.7, 129.4, 127.4, 71.9, 68.6, 56.0, 48.5, 38.0, 35.8, 32.6, 31.3, 16.1.

***N*-(2-(1*H*-indol-3-yl)ethyl)-3-phenylpropan-1-amine, 5C**

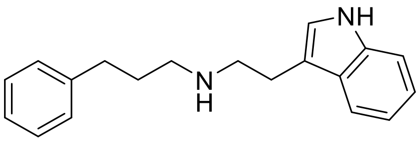

Colourless oil isolated. ^{1}H NMR δ_H (500 MHz, CD_3OD) 7.55 (d, J = 8.0 Hz, 1H), 7.37 (d, J = 8.0 Hz, 1H), 7.30–7.25 (m, 2H), 7.21–7.16 (m, 3H), 7.15 (s, 1H), 7.12 (ddd, J = 8.0, 6.9, 1.0 Hz, 1H), 7.04 (ddd, J = 8.0, 6.9, 1.0 Hz, 1H), 3.28 (t, J = 7.9 Hz, 2H), 3.13 (t, J = 7.6 Hz, 2H), 3.00 (m, 2H), 2.68 (t, J = 7.6 Hz, 2H), 1.97 (m, 2H). ^{13}C NMR δ_C (125 MHz, CD_3OD) 141.6, 138.3, 129.6, 129.3, 128.1, 127.4, 124.2, 122.8, 120.1, 118.9, 112.6, 110.0, 49.2, 48.4, 33.5, 28.9, 23.4.

***N*-(1-(naphthalen-1-yl)ethyl)-3-phenylpropan-1-amine, 5F**

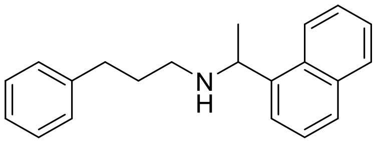

Colourless oil isolated. ^{1}H NMR δ_H (500 MHz, CD_3OD) 8.12 (d, J = 8.6 Hz, 1H), 7.91 (m, 2H), 7.65 (dd, J = 7.3, 1.1 Hz, 1H), 7.60 (ddd, J = 8.5, 6.8, 1.4 Hz, 1H) 7.54 (m, 2H), 7.14 (m, 2H), 7.08 (m, 1H), 7.03 (m, 2H), 5.30 (q, J = 6.8 Hz, 1H), 3.01 (ddd, J = 12.4, 10.2, 6.1 Hz, 1H), 2.81 (ddd, J = 12.4, 10.2, 6.1 Hz, 1H), 2.55 (td, J = 7.5, 3.5 Hz, 2H), 1.93 (m, 2H), 1.71 (d, J = 6.8 Hz, 3H). ^{13}C NMR δ_C (125 MHz, CD_3OD) 141.4, 135.4, 134.2, 132.0, 130.9, 130.3, 129.5, 129.3, 128.5, 127.5,

127.3, 126.6, 124.7, 123.0, 54.0, 46.8, 33.4, 29.0, 20.0.

***N*-benzyl-2-(5-methoxy-1*H*-indol-3-yl)ethan-1-amine, 6G**

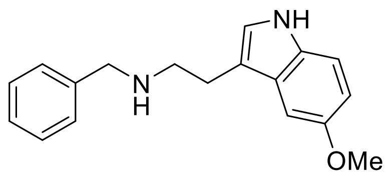

Colourless oil isolated. ^{1}H NMR δ_H (500 MHz, CD_3OD) 7.33–7.40 (m, 5H), 7.20 (d, J = 8.8 Hz, 1H), 7.07 (s, 1H), 6.96 (d, J = 2.4 Hz, 1H), 6.73 (dd, J = 8.8, 2.4 Hz, 1H), 3.74 (s, 3H), 3.73 (m, 2H), 3.22 (t, J = 7.7 Hz, 2H), 3.06 (t, J = 7.7 Hz, 2H). ^{13}C NMR δ_C (125 MHz, CD_3OD) 155.2, 133.4, 132.5, 130.9, 130.6, 130.2, 128.4, 124.9, 113.3, 113.0, 109.9, 100.9, 56.3, 52.1, 48.6, 23.2.

***N*-benzyl-3,5-dimethyladamantan-1-amine, 6H**

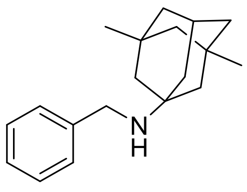

Colourless oil isolated. ^{1}H NMR δ_H (500 MHz, CD_3OD) 7.45–7.52 (m, 5H), 3.30–3.34 (m, 2H), 2.34 (m, 1H), 1.88 (m, 2H), 1.73–1.60 (m, 4H), 1.43–1.52 (m, 4H), 1.30 (m, 2H), 0.98 (s, 6H). ^{13}C NMR δ_C (125 MHz, CD_3OD) 133.5, 131.0, 130.5, 130.3,60.4, 50.9, 45.4, 45.0, 42.9, 38.1, 33.7, 31.4, 30.2.

***N*-(2-(1*H*-indol-3-yl)ethyl)-2-phenylethan-1-amine, 7C**

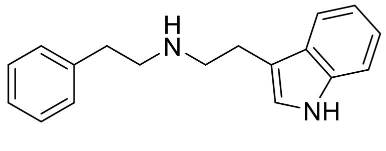

Colourless oil isolated. ^{1}H NMR δ_H (500 MHz, CD_3OD) 8.49 (s, 1H), 7.54 (d, J = 8.0 Hz, 1H), 7.34 (d, J = 8.1 Hz, 1H), 7.26–7.31 (m, 2H), 7.18–7.24 (m, 3H), 7.13 (s, 1H), 7.10 (t, J = 7.6 Hz, 1H), 7.02 (t, J = 7.6 Hz, 1H), 3.26–3.32 (m, 2H), 3.21 (m, 1H), 3.14 (m, 1H), 2.94 (m, 1H). ^{13}C NMR δ_C (125 MHz, CD_3OD) 138.3, 137.8, 130.0, 129.7, 128.2, 128.1, 124.2, 122.8, 120.1, 118.9, 112.6, 110.2, 49.9, 49.3, 33.3, 23.4.

***N*-(3,3-diphenylpropyl)heptan-1-amine, 8I**

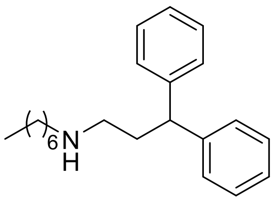

Colourless oil isolated. ^{1}H NMR δ_H (500 MHz, CD_3OD) 7.26–7.30 (m, 8H), 7.18 (m, 2H), 4.02 (t, J = 7.9 Hz, 1H), 2.97–2.77 (m, 4H), 2.42 (m, 2H), 1.60 (m, 2H), 1.41–1.20 (m, 8H), 0.87 (t, J = 6.5 Hz, 3H). ^{13}C NMR δ_C (125 MHz, CD_3OD) 144.8, 129.8, 128.7, 127.8, 49.8, 48.9, 47.9, 32.9, 32.7, 29.9, 27.5, 27.4, 23.6, 14.4.

***N*-(3,3-diphenylpropyl)-3,7-dimethyloct-6-en-1-amine, 9I**

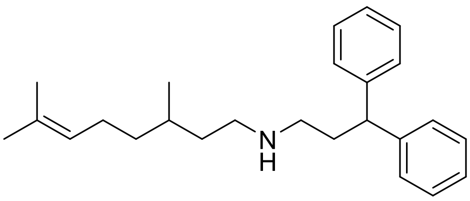

Colourless oil isolated. ^{1}H NMR δ_H (500 MHz, CD_3OD) 7.27–7.30 (m, 8H), 7.22–7.13 (m, 2H), 5.07 (m, 1H), 4.02 (t, J = 7.9 Hz, 1H), 2.85–3.01 (m, 4H), 2.38–2.36 (m, 2H), 1.90–2.06 (m, 2H), 1.65 (s, 3H), 1.62 (m, 1H), 1.58 (s, 1.58), 1.37–1.54 (m, 2H), 1.24–1.37 (m, 2H), 1.19 (m, 1H), 0.91 (d, J = 6.5 Hz, 3H). ^{13}C NMR δ_C (125 MHz, CD_3OD) 144.7, 132.5, 129.8, 128.7, 127.8, 125.3, 49.8, 47.9, 47.3, 37.9, 34.2, 32.9, 31.5, 26.3, 25.9, 19.4, 17.7.

Supplementary Note 2: Amino acid and DNA sequences of IR-G02

Amino acid sequence of IR-G02 (WP_010638508.1, *Streptomyces albidoflavus*)[3]

MSTPPHTTAGPAAVTVLGLGRMGSALAAAFLAAGHSTTVWNRTPGKADELAARGARRA

GSVAEAVAAAPLVVVCVADDEAVHQLLDPLDGALAGRTLVNLTTGTSAQARANAAWAKE

RGAAFLDGAIMAVPEDIATGDAVLLYSGPRDAFDAYEEALRVLAPAGTTHLGGDAGLAAL

HDLALLGIMWGVLNGFLHGAALLGTAGVRAGDFAPLAARMTTVVAGYVTAAAPEVDAG

SYPAGDATLTVHQEAMRHLAEESEALGVNAELPRFLQLLAGRAVAEGHAESGYSALVEQF

RKA*

DNA sequence of IR-G02

ATGAGCACCCCGCCGCACACCACCGCGGGTCCGGCGGCGGTTACCGTGCTGGGTCTG

GGTCGTATGGGTAGCGCGCTGGCGGCGGCGTTCCTGGCGGCGGGTCACAGCACCACC

GTTTGGAACCGTACCCCGGGTAAAGCGGATGAGCTGGCGGCGCGTGGTGCGCGTCGT

GCGGGCAGCGTTGCGGAAGCGGTGGCGGCGGCGCCGCTGGTTGTGGTGTGCGTTGCG

GACGATGAAGCGGTGCACCAGCTGCTGGACCCGCTGGATGGTGCGCTGGCGGGTCGT

ACCCTGGTGAACCTGACCACCGGTACCAGCGCGCAAGCGCGTGCGAACGCGGCGTGG

GCGAAAGAACGTGGTGCGGCGTTCCTGGATGGTGCGATCATGGCGGTTCCGGAAGAC

ATTGCGACCGGTGATGCGGTGCTGCTGTACAGCGGTCCGCGTGACGCGTTTGATGCGT

ATGAGGAAGCGCTGCGTGTTCTGGCGCCGGCGGGTACCACCCATCTGGGTGGCGACGC

GGGTCTGGCGGCGCTGCACGATCTGGCGCTGCTGGGTATCATGTGGGGCGTTCTGAAC

GGTTTCCTGCATGGTGCGGCGCTGCTGGGTACCGCGGGCGTGCGTGCGGGCGACTTTG

CGCCGCTGGCGGCGCGTATGACCACCGTTGTTGCGGGTTATGTGACCGCGGCGGCGCC

GGAAGTGGATGCGGGTAGCTACCCGGCGGGTGATGCGACCCTGACCGTTCACCAGGA

AGCGATGCGTCATCTGGCGGAGGAAAGCGAAGCGCTGGGCGTGAACGCGGAACTGCC

GCGTTTCCTGCAACTGCTGGCGGGTCGTGCGGTTGCGGAGGGTCATGCGGAAAGCGG

TTACAGCGCGCTGGTTGAGCAATTTCGTAAGGCGTAA

Supplementary Tables

Supplementary Table 1 Biotransformation data for screening of 85 IREDs for reductive of a panel of carbonyls (**1-3**) and amines (**A-C**) with different molecular volumes.

Products	1A	1B	1C	2C	3C
*Asp*RedAm	90.0%	19.3%	3.8%	2.5%	3.6%
IR-G01	99.5%	16.9%	88.1%	12.1%	20.4%
IR-G02	88.7%	82.0%	93.6%	54.3%	51.5%
IR-G03	88.8%	79.0%	66.6%	16.7%	16.9%
IR-G04	90.7%	76.9%	41.6%	31.0%	4.0%
IR-G05	83.6%	70.1%	19.7%	14.1%	2.1%
IR-G06	89.0%	40.7%	4.1%	0.0%	0.0%
IR-G07	88.7%	29.8%	0.0%	0.0%	0.0%
IR-G08	87.2%	70.5%	42.2%	17.1%	5.1%
IR-G09	87.5%	55.2%	15.1%	0.0%	0.0%
IR-G10	94.7%	51.8%	10.6%	2.2%	0.0%
IR-G11	86.9%	59.7%	10.5%	71.1%	0.0%
IR-G12	99.1%	61.8%	6.2%	5.8%	2.6%
IR-G13	87.9%	96.4%	85.9%	24.0%	18.8%
IR-G14	88.6%	76.0%	5.6%	0.0%	2.5%
IR-G15	94.2%	46.9%	0.0%	0.0%	3.0%
IR-G16	95.5%	57.7%	7.2%	13.7%	4.7%
IR-G17	95.4%	87.7%	10.7%	0.0%	6.0%
IR-G18	77.8%	79.0%	7.4%	11.0%	0.0%
IR-G19	88.3%	8.5%	0.0%	4.6%	3.3%
IR-G20	75.1%	10.3%	0.0%	0.0%	0.0%
IR-G21	66.4%	87.4%	76.9%	22.1%	93.2%
IR-G22	53.6%	9.2%	4.6%	0.0%	14.4%
IR-G23	74.4%	7.5%	0.0%	0.0%	0.0%
IR-G24	65.2%	25.8%	5.2%	2.0%	0.0%
IR-G25	73.4%	24.5%	30.0%	6.1%	0.0%
IR-G26	78.0%	76.7%	13.1%	3.7%	0.0%
IR-G27	75.5%	11.9%	2.1%	6.1%	8.1%
IR-G28	80.8%	19.6%	2.1%	2.2%	0.0%
IR-G29	82.5%	11.1%	0.0%	0.0%	2.9%
IR-G30	50.9%	33.2%	3.5%	0.0%	0.0%
IR-G31	47.6%	35.7%	32.7%	0.0%	5.8%
IR-G32	83.4%	27.3%	0.0%	0.0%	0.0%
IR-G33	77.8%	14.0%	0.0%	0.0%	0.0%
IR-G34	95.0%	72.4%	13.8%	5.0%	14.3%
IR-G35	79.8%	51.5%	82.4%	79.1%	99.0%
IR-G36	81.8%	52.5%	25.7%	51.1%	9.5%
IR-G37	75.9%	26.9%	0.0%	2.5%	0.0%

IR-G38	84.5%	36.8%	5.1%	3.0%	2.2%
IR-G39	82.2%	79.0%	74.8%	7.0%	2.3%
IR-G40	99.0%	8.2%	5.7%	13.4%	3.5%
IR-G41	81.7%	7.9%	3.0%	2.8%	0.0%
IR-G42	47.2%	29.1%	12.0%	0.0%	0.0%
IR-G43	86.3%	18.0%	3.7%	2.1%	0.0%
IR-G44	84.9%	10.5%	0.0%	8.8%	0.0%
IR-G45	33.0%	6.5%	0.0%	2.1%	0.0%
IR-G46	20.3%	8.1%	0.0%	0.0%	0.0%
IR-G47	89.3%	7.7%	0.0%	2.0%	0.0%
IR-G48	34.5%	8.2%	0.0%	0.0%	0.0%
IR-G49	85.6%	99.5%	4.2%	5.2%	46.3%
IR-G50	7.6%	6.0%	0.0%	0.0%	0.0%
IR-G51	35.9%	6.1%	0.0%	0.0%	0.0%
IR-G52	29.2%	6.3%	0.0%	0.0%	0.0%
IR-G53	85.4%	7.0%	0.0%	5.1%	0.0%
IR-G54	28.3%	12.1%	0.0%	0.0%	0.0%
IR-G55	41.5%	6.5%	0.0%	0.0%	0.0%
IR-G56	99.4%	6.9%	0.0%	0.0%	0.0%
IR-G57	99.5%	58.0%	8.1%	29.8%	5.5%
IR-G58	62.1%	6.9%	5.1%	0.0%	0.0%
IR-G59	65.6%	16.8%	0.0%	0.0%	0.0%
IR-G60	80.3%	29.1%	17.4%	12.0%	11.2%
IR-G61	62.4%	33.0%	11.8%	10.1%	0.0%
IR-G62	83.3%	25.1%	21.6%	4.1%	0.0%
IR-G63	74.2%	34.9%	5.1%	0.0%	0.0%
IR-G64	93.1%	21.2%	15.9%	0.0%	3.8%
IR-G65	94.3%	41.7%	3.9%	22.8%	4.7%
IR-G66	6.4%	0.0%	0.0%	0.0%	0.0%
IR-G67	0.0%	0.0%	0.0%	0.0%	0.0%
IR-G68	16.0%	0.0%	0.0%	0.0%	0.0%
IR-G69	66.0%	0.0%	0.0%	0.0%	0.0%
IR-G70	67.3%	6.2%	0.0%	0.0%	0.0%
IR-G71	90.1%	4.3%	0.0%	0.0%	0.0%
IR-G72	79.3%	0.0%	0.0%	0.0%	0.0%
IR-G73	23.2%	0.0%	0.0%	0.0%	0.0%
IR-G74	59.6%	0.0%	0.0%	0.0%	0.0%
IR-G75	76.7%	0.0%	0.0%	0.0%	0.0%
IR-G76	23.1%	0.0%	0.0%	0.0%	0.0%
IR-G77	0.0%	0.0%	0.0%	0.0%	0.0%
IR-G78	58.8%	0.0%	0.0%	0.0%	0.0%
IR-G79	13.2%	0.0%	0.0%	0.0%	0.0%
IR-G80	38.4%	0.0%	0.0%	0.0%	0.0%
IR-G81	64.3%	3.5%	0.0%	0.0%	0.0%

IR-G82	32.9%	0.0%	0.0%	0.0%	0.0%
IR-G83	43.5%	0.0%	0.0%	0.0%	0.0%
IR-G84	48.6%	0.0%	0.0%	0.0%	0.0%
IR-G85	10.5%	0.0%	0.0%	0.0%	0.0%

Supplementary Table 2 Relative biotransformation data (peak area of extracted ion of products) for screening of IR-G02 for reductive of a panel of carbonyls (**1-11**) and amines (**A-R**) with different molecular volumes.

	**1**	**2**	**3**	**4**	**5**	**6**	**7**	**8**	**9**	**10**	**11**
**A**	19158400	13501500	8045170	14688500	20938900	13969900	18081800	12566200	16821400	0	0
**B**	19700000	6481797	2501947	6833751	12743990	14727800	6572520	3758210	25088700	0	0
**C**	16704600	15423000	8704600	968761.3	11136400	13918500	10795000	350124.9	23875300	0	0
**D**	8409050	5069500	0	7280760	845998.1	630740.3	317254.2	0	15796100	0	0
**E**	20177500	13225400	0	4529580	11709800	21702200	5977080	1218780	41544400	0	0
**F**	99093.4	21032.7	0	0	10444543	7698940	626436.6	440098.5	25432300	0	0
**G**	14204200	8570280	0	0	13380800	11067300	2837370	0	23081400	0	0
**H**	0	0	0	0	22179174	17732066	22387000	0	31416600	0	0
**I**	433809.4	122521.5	0	208602.5	5573309	2805170	1188020	10105400	13403300	0	0
**J**	7950200	11838100	0	290563.1	3264390	8222250	1302680	0	23549600	0	0
**K**	0	0	0	0	25290082	4840461	16491600	0	32613500	0	0
**L**	17914400	13153997	0	4467183	9770049	12306700	5615990	3516000	27863100	0	0
**M**	3313510	3957686	0	915136.7	7619173	14773900	2307390	0	15206100	0	0
**N**	254392.8	191510.4	0	528373.9	15329300	3918610	1525300	5848830	31837500	0	0
**O**	1468480	0	553367.9	76681.1	0	0	0	0	0	0	0
**P**	12895800	5666870	2576270	9057880	17081000	11654800	11143800	4806060	23540900	2734210	418391.5
**Q**	16984400	12015500	1277380	6737810	18792100	17447900	3900270	4110620	49353000	120326.2	0
**R**	15247300	7303710	457396.3	1101740	16649900	10535400	22437100	796314.3	34046400	1158150	202891.2

Supplementary Table 3 Relative biotransformation data (peak area of extracted ion of products) for screening of IR-G02 for reductive of a panel of carbonyls (**12-23**) and amines (**A-R**) with different molecular volumes.

	**12**	**13**	**14**	**15**	**16**	**17**	**18**	**19**	**20**	**21**	**22**	**23**
**A**	0	0	11419600	0	0	13374700	7072690	11771200	9533720	14750600	4065990	0
**B**	192499.7	1159423	15432500	0	0	17207200	2124130	2088310	11334600	1566170	637732.3	920612.4
**C**	4127695	0	13659100	148384.9	553495.4	15437900	77009.9	454938	9582050	0	0	0
**D**	2501947	0	0	324996.1	668882.5	8401610	804161.9	0	0	0	3924700	0
**E**	0	0	11196800	0	360255	22173900	3722420	3491160	15476400	3916150	1435570	0
**F**	0	0	93851	0	0	13715500	0	0	3184770	0	0	158498.2
**G**	0	0	12576000	212640.5	1923720	16932200	0	0	5653340	0	0	0
**H**	0	5441635	0	0	0	0	0	0	0	0	0	0
**I**	0	0	565413.7	0	0	8447170	0	0	1303790	0	0	336023.7
**J**	0	0	6002090	0	0	8858290	0	0	1487140	0	0	0
**K**	0	0	0	0	0	19440032	0	0	0	0	0	0
**L**	543755.9	216701.8	14990600	0	761401.4	10925000	1191700	9071620	0	9504500	11521200	685353.3
**M**	280020.4	0	3202200	0	585432.3	9474450	0	0	1787410	0	0	204402.5
**N**	0	0	0	0	547147.3	26192000	0	0	4551240	0	1200570	0
**O**	0	0	0	0	0	144067.9	0	0	7441920	9051760	0	0
**P**	0	5533102	8924520	0	0	10283400	5390810	8093250	9076440	5453930	0	0
**Q**	0	0	19631600	0	0	22324800	3733120	5943880	10039700	8104850	0	0
**R**	0	0	8715900	6556630	0	21070300	871631.1	1246120	13444200	1052930	0	0

Supplementary Table 4. Biotransformation data for screening of 19 variants of IR-G02 for reductive aminations of a panel of carbonyls (**1-3**) and amines (**A-C**) with different molecular volumes, and imine reduction of **24**.

	**24**	**1A**	**1B**	**1C**	**2C**	**3C**
WT	96.7%	88.7%	82.0%	93.6%	54.3%	51.5%
M217A	89.4%	59.4%	73.8%	3.7%	3.8%	3.6%
W187A	59.4%	53.5%	28.7%	16.8%	7.1%	22.1%
H247A	76.5%	43.6%	19.7%	5.6%	5.4%	11.3%
D241A	94.6%	64.7%	73.8%	93.6%	59.7%	99.0%
V246A	93.4%	62.7%	69.7%	84.2%	52.1%	57.2%
T243A	99.4%	60.1%	66.4%	82.4%	48.8%	69.5%
V130A	80.3%	58.7%	77.9%	68.3%	36.3%	48.9%
M128A	83.0%	56.8%	78.7%	66.5%	22.8%	21.6%
I127A	88.7%	58.7%	78.7%	83.3%	36.9%	51.5%
T102A	25.4%	51.5%	15.6%	7.5%	13.0%	21.1%
D179A	83.9%	57.4%	27.9%	47.7%	15.2%	12.9%
L183A	35.6%	56.8%	22.1%	4.7%	2.7%	23.2%
M186A	83.4%	59.4%	99.0%	22.5%	9.2%	40.2%
L190A	74.7%	62.0%	77.1%	69.3%	14.6%	34.5%

Supplementary Table 5. Investigation of process parameters for IR-G02-catalysed reductive amination of **5** and **F**.

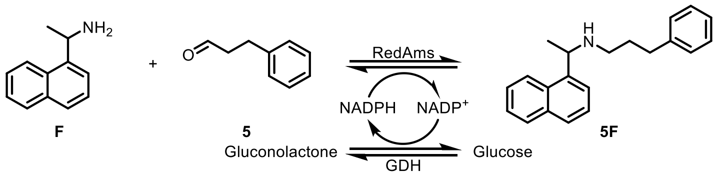

Entry	Ketone (mM)	Amine (mM)	pH (PBS Buffer)	Temperature	Time (h)	Conversion/%
1	5	5	6.0	30	3	38
2	5	5	7.0	30	3	>48
3	5	5	8.0	30	3	44
4	5	5	7.0	25	3	46
5	5	5	7.0	37	3	42.5
6	5	5	7.0	42	3	38
7	10	10	7.0	30	3	>48
8	15	15	7.0	30	3	>48
9	20	20	7.0	30	3	>48
10	25	25	7.0	30	3	38.5
11	30	30	7.0	30	3	32.5

Supplementary Table 6. Data collection and refinement statistics (molecular replacement).

	IR-G02-apo（7XE8）	IR-G02-NAPDH（7XR5）
Crystal conditions	0.2M ammonium acetate, 0.1M Bis Tris pH5.5, 25% PEG 3350	0.1M Bis-Tris pH7.5,25% PEG4000 0.25M NaCl
Wavelength	0.97918	0.97915
Resolution range	27.07-1.72 (1.781-1.72)	34.01- 1.58 (1.64-1.58)
Space group	C 1 2 1	P 1 21 1
Unit cell	110.5 65.8 126.9 90 103.772 90	76.3 120.03 76.5 90 117.2 90
Total reflections	584598 (32344)	848479 (86727)
Unique reflections	90868 (8782)	166817 (16450)
Multiplicity	6.4 (3.7)	5.1 (5.2)
Completeness (%)	96.62 (93.85)	98.64 (98.74)
Mean I/sigma(I)	28.13 (2.75)	14.62 (2.59)
Wilson B-factor	25.77	18.36
R-merge	0.04 (0.36)	0.08 (0.89)
CC1/2	1 (0.88)	0.998 (0.662)
Reflections used in refinement	90845 (8782)	164685 (16450)
Reflections used for R-free	1987 (195)	8045 (806)
R-work	0.1737 (0.2193)	0.1791 (0.2424)
R-free	0.2030 (0.2694)	0.2015 (0.2845)
R_{work}	0.967 (0.884)	0.966 (0.821)
R_{free}	0.958 (0.819)	0.959 (0.779)
Number of non-hydrogen atoms	7014	9603
macromolecules	6130	8179
ligands	0	302
solvent	884	1122
Protein residues	865	1156
RMS(bonds)	0.009	0.006
RMS(angles)	1.02	0.92
Ramachandran favored (%)	98.49	98.00
Ramachandran allowed (%)	1.51	2.00
Ramachandran outliers (%)	0.00	0.00
Rotamer outliers (%)	0.36	0.13
Average B-factor	28.91	21.52
macromolecules	27.66	20.31
ligands	-	21.95
solvent	37.54	30.25

Supplementary Figures

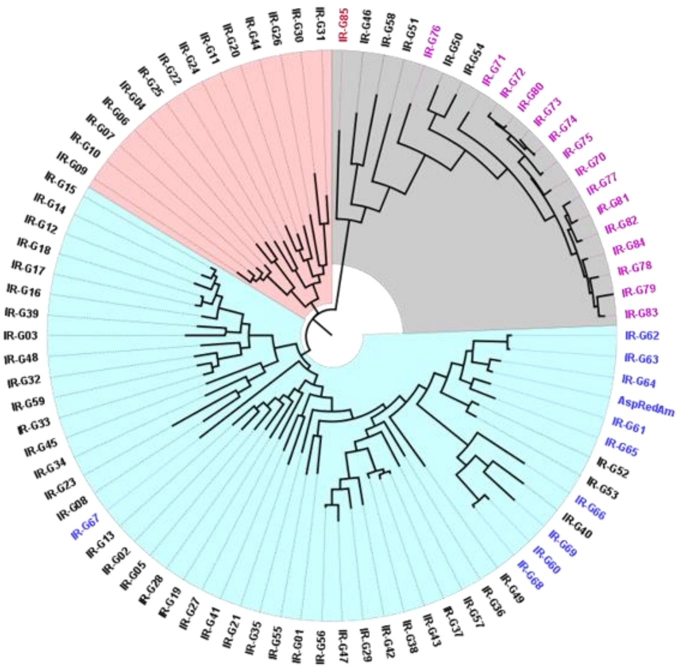

Supplementary Figure 1. The phylogenetic tree of 85 IREDs and *Asp*RedAm. All the 85 reported IREDs were selected from bacteria (black), fungi (blue), plants (purple), and human (red).

```
IR-G02          MSTPPHTTAGPAAVTVLGLGRMGSALAAAFLAAGHSTTVWNRTPGKADELAARGARRAGS
IR-G21          ----MVTEASPAPVSVIGLGLMGAALAGAYLKAGHQTTVWNRSAGKADALVAQGATNAAD
IR-G35          --------MSTKKVTVIGLGSLGSALAAALLRTGHDVTVWNRTPEKAEALVAQGATRADT
                             *.*.*** .*.*** * * .**  *****.  **. * *.**  *

IR-G02          VAEAVAAAPLVVVCVADDEAVHQLLDPLDGALAGRTLVNLTTGTSAQARANAAWAKERGA
IR-G21          IAEAVAASDVLVVCVVDYAAFHAILEPVKDALQGKVIVNLTSGLPDDARGAAEWASGTGA
IR-G35          VAEAAAASPVVIVCVFDTEAARELLAPIQ---AGKAVVNLTSGSPDEARELAAWAASRGV
                .*** **. ...*** *  *  . .* *.     *. .****.*    **  * **   *

IR-G02          AFLDGAIMAVPEDIATGDAVLLYSGPRDAFDAYEEALRVLAPAGTTHLGGDAGLAALHDL
IR-G21          EYLDGYIMSVPPGVGLPQTLLFYGGDADVFAKHEATLKVLG-GNSIHLGADAGVAALYDL
IR-G35          DYLDGAVMAVPAAIGTPDAFVMYSGSREVFDAHRAALDSFG--ASHFLGEDAGVAEFHDL
                 .*** .*.**  .    . . * *  . *    .*        .  ** ***.*   **

IR-G02          ALLGIMWGVLNGFLHGAALLGTAGVRAGDFAPLAAR-MTTVVAGYVTAAAPEVDAGSYPA
IR-G21          GLLAILWSSLAGALHAYALVASEKIPAAALAPFAEQWITHVVLPSVKGAAAAVDSGQYAT
IR-G35          GLLYAGYATLVGFLNSVAIVGTAGVTARELLPLVTTWLTGMVA-YLADVAREVDERDYTD
                 **     * * *.  *.. .  . *    *     .* .*   .    *  **   *

IR-G02          GDATLTVHQEAMRHLAEESEALGVNAELPRFLQLLAGRAVAEGHAESGYSALVEQFRKA-
IR-G21          SVSTTALNAVGLGKMVEASKAAGIRPDLMLPIKAYLEQRVADGHGEEALAGMFEVIRSPE
IR-G35          GASSVAINQVALDKIIAASRAAGVSPDLLLPFKELLDRRAADGHARDSASSVIELLRPGI
                  .. ...   . ..   * * *.  .*    .    .  *.**     . . *  *

IR-G02          ---
IR-G21          R--
IR-G35          HPE
```

Supplementary Figure 2. Sequence alignment three IREDs of IR-G02, IR-G21, and IR-G35.

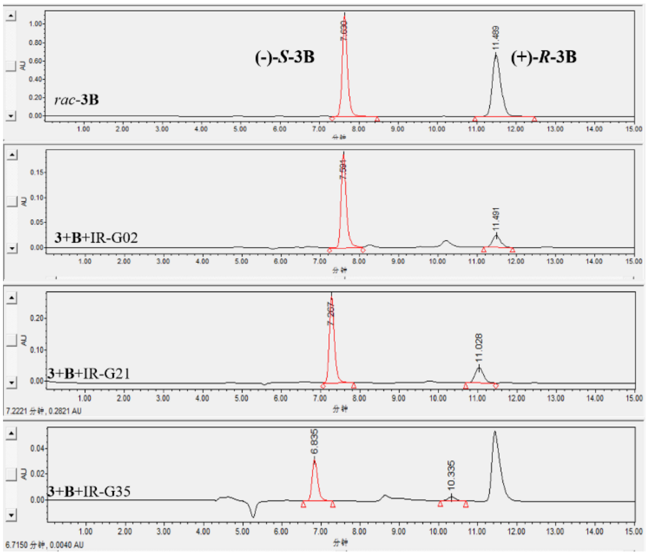

Supplementary Figure 3. HPLC analysis (CHIRALPAK AY-H; *n*-hexane/ethanol/diethylamine = 90/10/0.02, v/v) showing standard of *rac*-**3B** and IR-G/2/21/35-catalysed amine products.

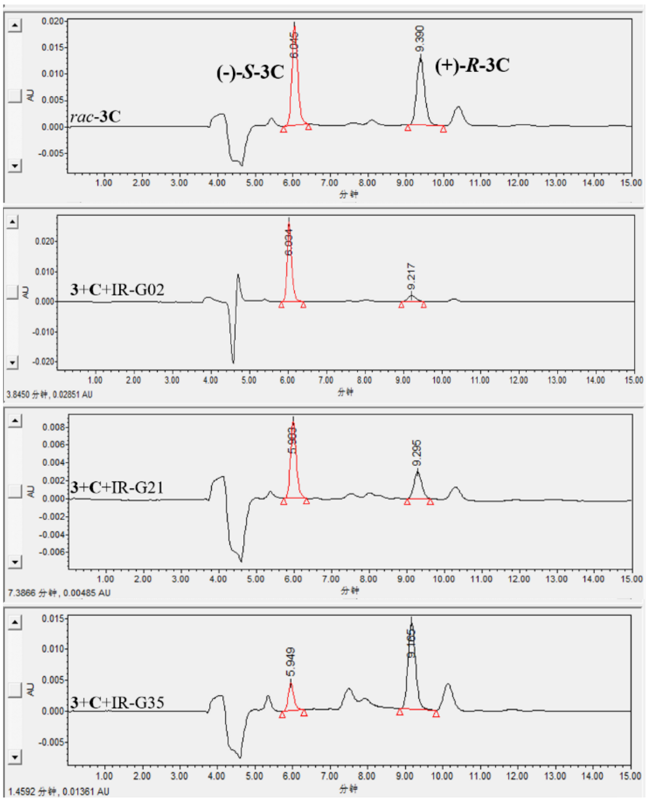

Supplementary Figure 4. Chiral HPLC analysis (CHIRALPAK IA; *n*-hexane/ethanol/diethylamine = 85/15/0.02, v/v) showing standard of *rac*-**3C** and IR-G02/21/35-catalysed amine prodcuts.

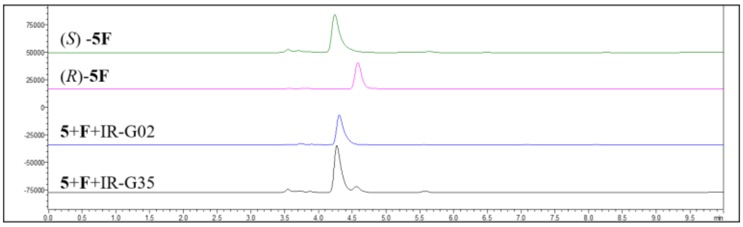

Supplementary Figure 5. Chiral HPLC analysis (CHIRALPAK AY-H; *n*-hexane/ethanol/diethylamine = 85/15/0.02, v/v) showing standard of *R*-**5F**, standard of *S*-**5F** and IR-G02/35-catalysed amine products.

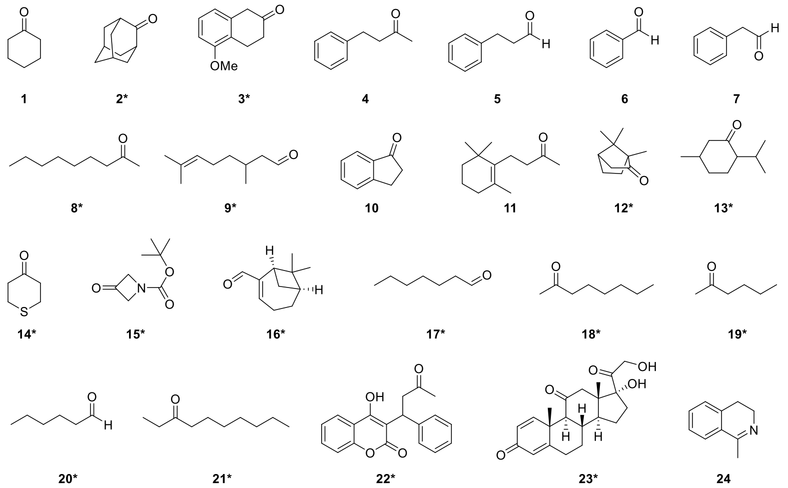

Supplementary Figure 6. Numbering of ketones (**1-23**) and imine (**24**) used in this study.

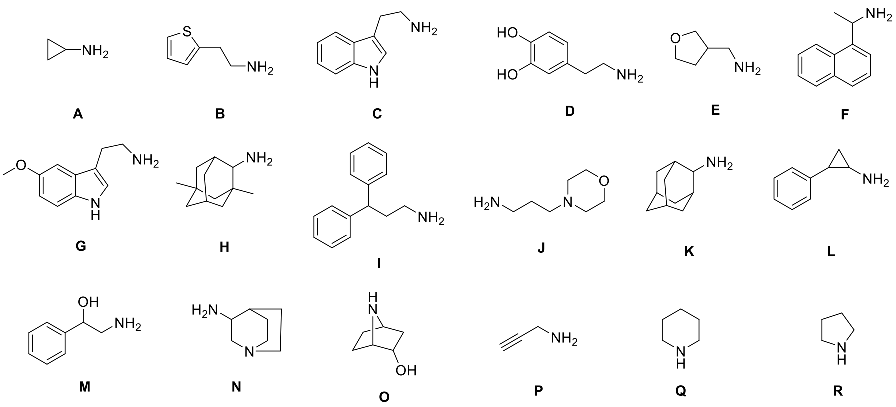

Supplementary Figure 7. Numbering of amines (**A-R**) used in this study.

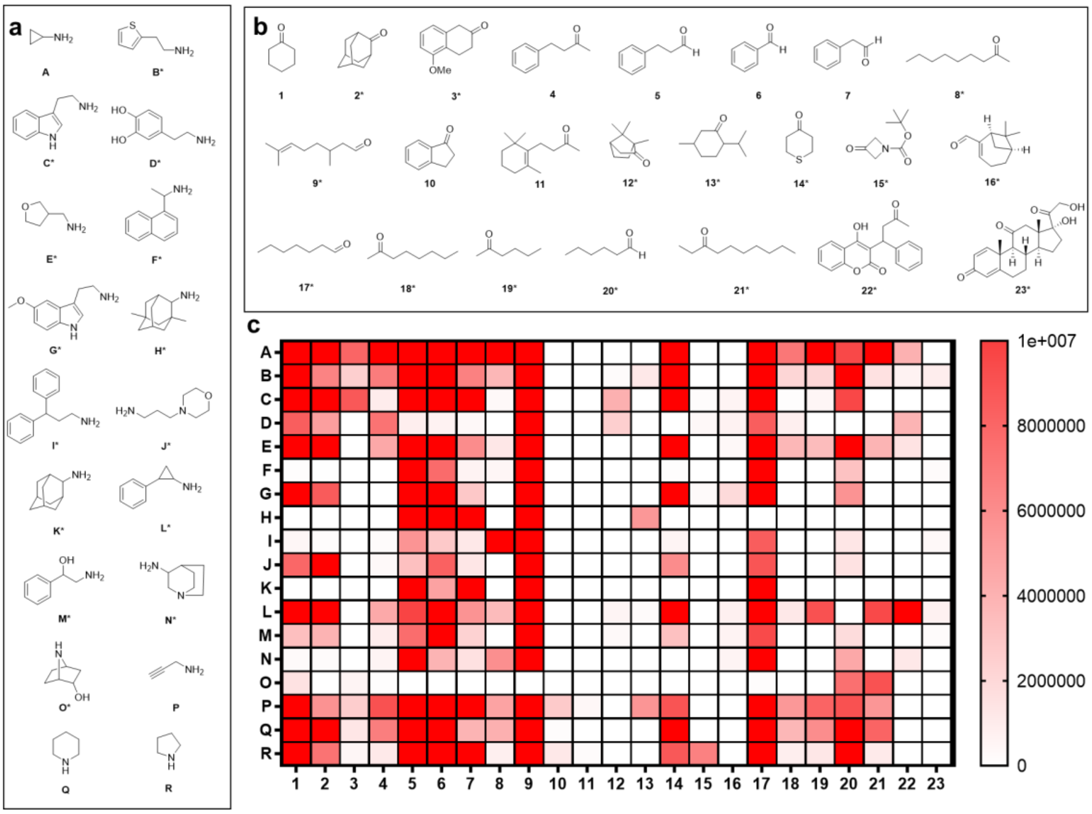

Supplementary Figure 8. LC-MS ion abundance for IR-G02-catalysed reactions based on specific activities of a panel of carbonyl compounds and amine reacting partners. **a** Carbonyls are used in reductive amination reactions. *The substrates used only in this study. **b** Amines used in the reductive amination reactions. *The substrates used only in this study. **c** Relative mass ion abundance for the reductive-amination products. The red color scale in each tile is representative of the mass ion abundance detected by the LC-MS. The numbered tiles are representatives for the reductive-amination products with ion abundance > 5 × 106. All reaction conditions: ketone/aldehyde (5 mM), amine (1 to 4 eq), IR-G02 (1 mg ml^{-1}), $NADP^{+}$ (1 mM), GDH (0.2 mg/ml), D-glucose (30 mM), PBS buffer (100 mM, pH 7.0), 30 °C, 250 r.p.m., 24 h.

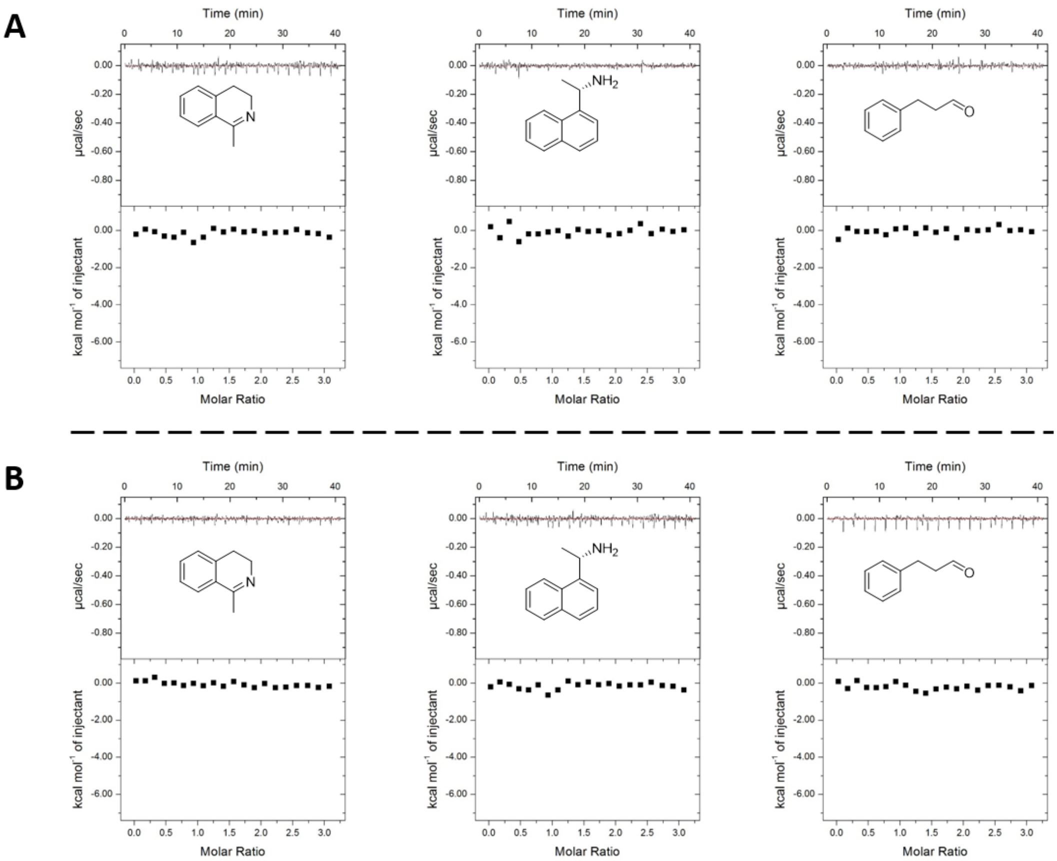

Supplementary Figure 9. Binding of ligands to purified IR-G02 by ITC in 100 mM phosphate buffer at 25 °C. (A) Three ligands (0.6 mM) were titrated into a microcalorimeter cell containing 40 μM of IR-G02. (B) Three ligands (0.6 mM) were titrated into a microcalorimeter cell containing the mixture of 40 μM of IR-G02 and 0.6 mM NADPH.

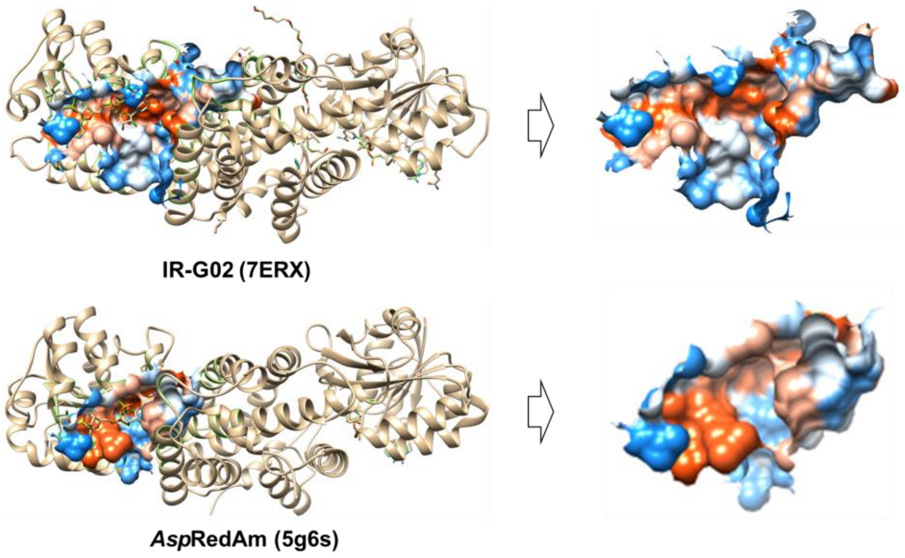

Supplementary Figure 10. The value of cavity volumes for IR-G02 and *Asp*RedAm.

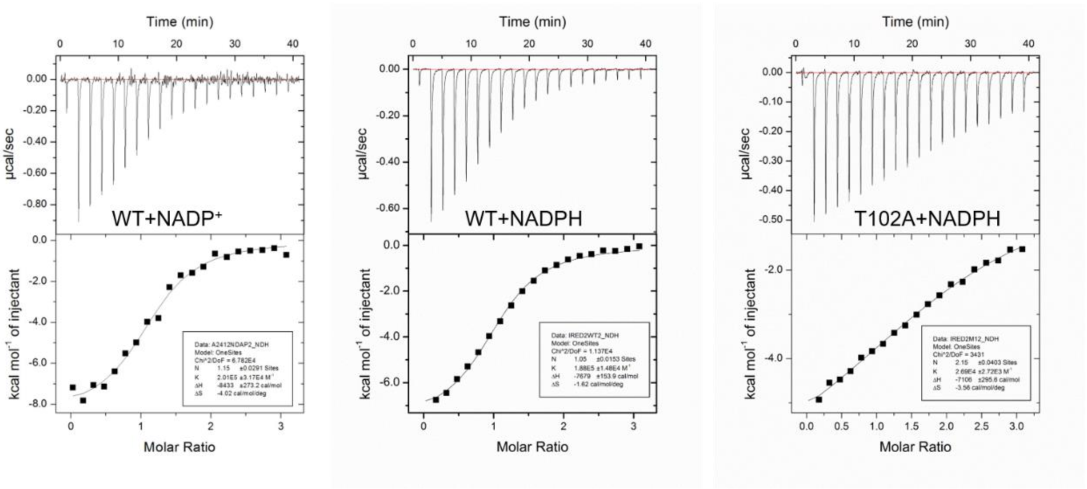

Supplementary Figure 11. Binding of NADP(H) to purified wild type (WT) or variant T102A of IR-G02 by ITC in 100 mM PBS buffer at 25 ℃. NADP(H) (0.4 mM) was titrated into a microcalorimeter cell containing 40 μM of WT or T102A.

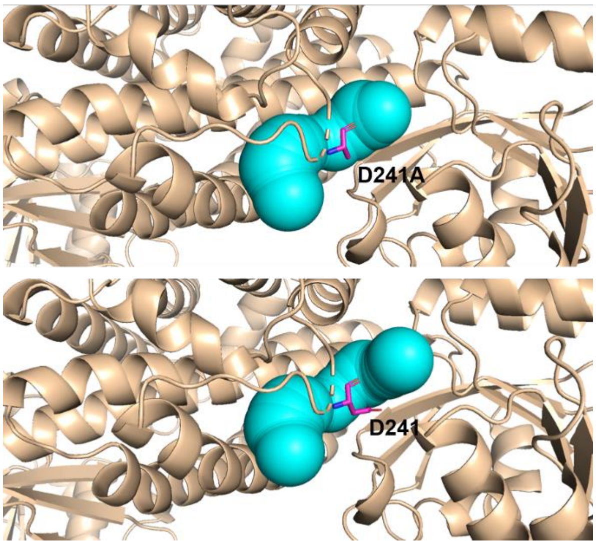

Supplementary Figure 12. The tunnels of the wild type and D241A of IR-G02 were analyzed by CAVER Analyst 2.0.

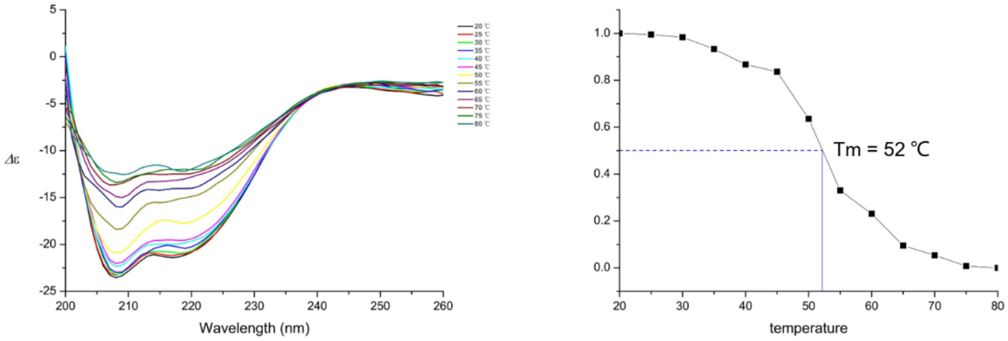

Supplementary Figure 13. Thermal denaturation studies as a measure of thermodynamic stability. (A) CD spectra of IR-G02 as the temperature is increased from 20 to 80 ℃. (B) Melting temperatures of IR-G02. CD220 plotted against temperature to calculate the melting temperatures of IR-G02.

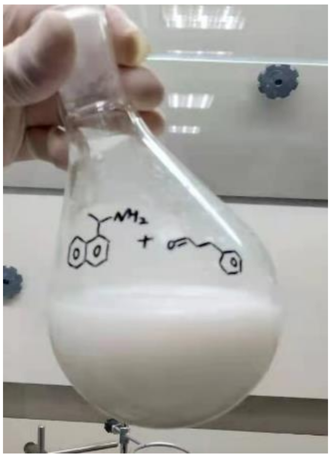

Supplementary Figure 14. Gram-scale biotransformation of 3-phenylpropionaldehyde **5** with *rac*-1-(1-naphthyl)ethylamine **F** by using IR-G02.

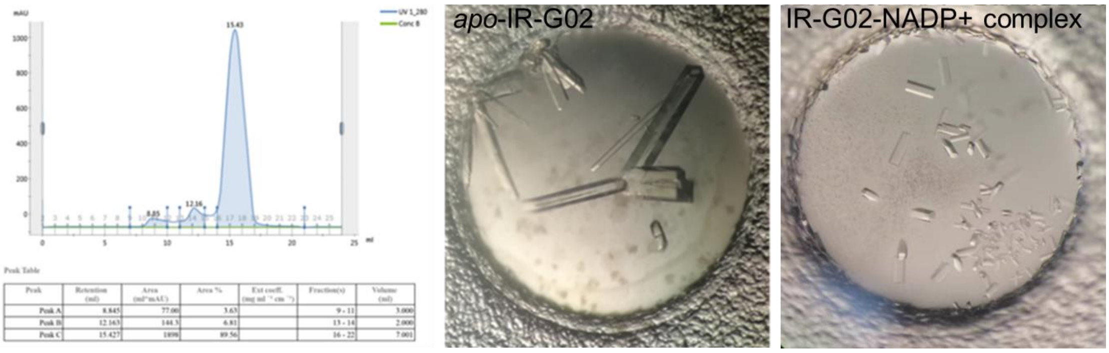

Supplementary Figure 15. The size exclusion chromatography and crystals of *apo*-IR-G02 and IR-G02-NADP$^+$ complex.

Supplementary Reference

1. Jurcik, A. et al. CAVER Analyst 2.0: analysis and visualization of channels and tunnels in protein structures and molecular dynamics trajectories. *Bioinformatics* **34**, 3586-3588 (2018).
2. Dundas, J. et al. CASTp: computed atlas of surface topography of proteins with structural and topographical mapping of functionally annotated residues. *Nucleic. Acids. Res.* **34**, W116-118 (2006).
3. Wetzl, D. et al. Expanding the Imine Reductase Toolbox by Exploring the Bacterial Protein-Sequence Space. *Chembiochem* **16**, 1749-1756 (2015).
