# Supplementary material for: Actinomycetes-derived imine reductases with a preference towards bulky amine substrates
Source: Commun Chem. 2022 Oct 8;5:123. doi: 10.1038/s42004-022-00743-y (PMC9814587; doi:10.1038/s42004-022-00743-y)
Supplement: Supplementary file 2 — Description of Additional Supplementary Files [file 42004_2022_743_MOESM2_ESM.docx]

Description of Additional Supplementary Files

**File name:** Supplementary data 1

**Description:** The crystal structure of apo-IR-G02

**File name:** Supplementary data 2

**Description:** The crystal structure of NADP+ complex of IR-G02

**File name:** Supplementary data 3

**Description:** LC-MS chromatograms for IREDs-catalyzed reductive aminations

**File name:** Supplementary data 4

**Description:** NMR spectra of all amine products
