# Supplementary material for: Actinomycetes-derived imine reductases with a preference towards bulky amine substrates
Source: Commun Chem. 2022 Oct 8;5:123. doi: 10.1038/s42004-022-00743-y (PMC9814587; doi:10.1038/s42004-022-00743-y)
Supplement: Supplementary file 5 — Supplementary Data 3 [file 42004_2022_743_MOESM5_ESM.pdf]

LC-MS Chromatograms

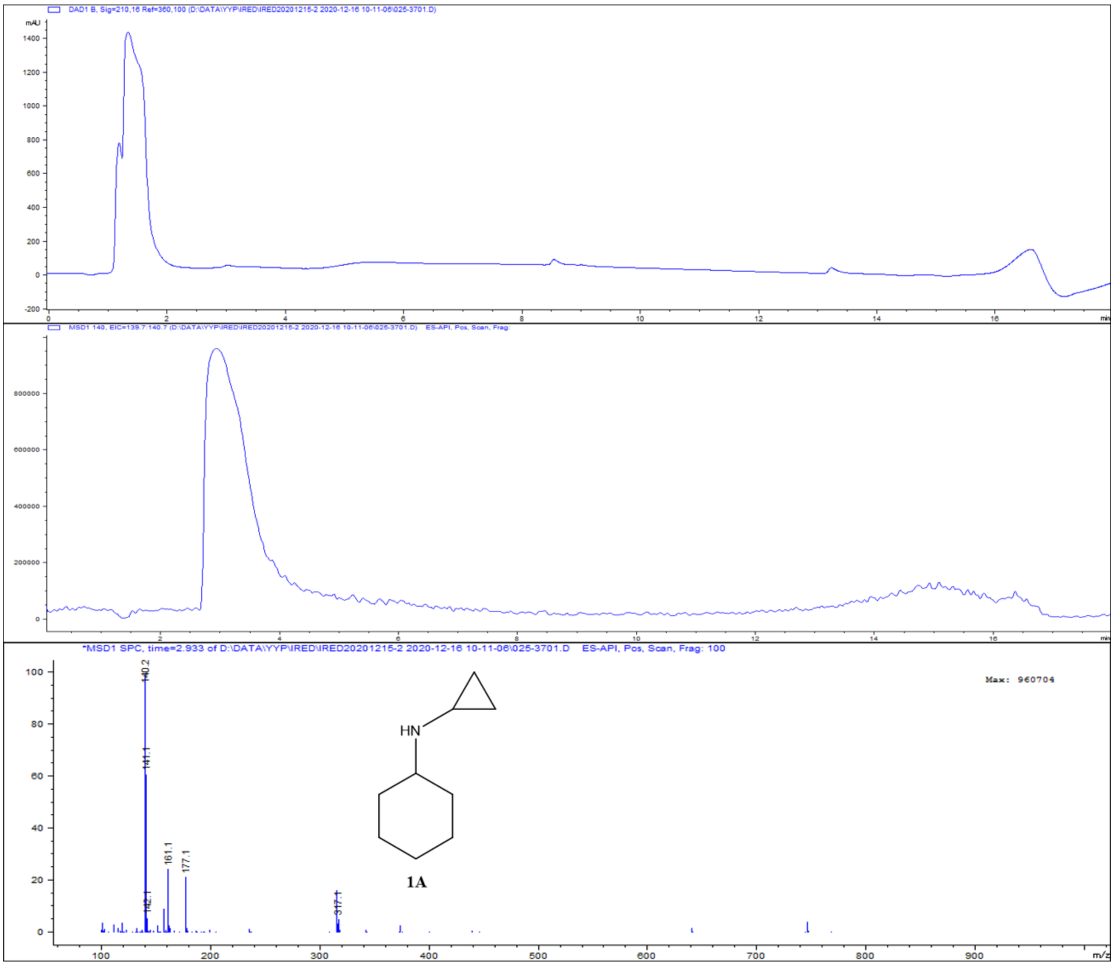

Figure 1. LCMS analysis: IR-G02-catalysed reductive amination of **1** with **A**, showing amine product **1A**.

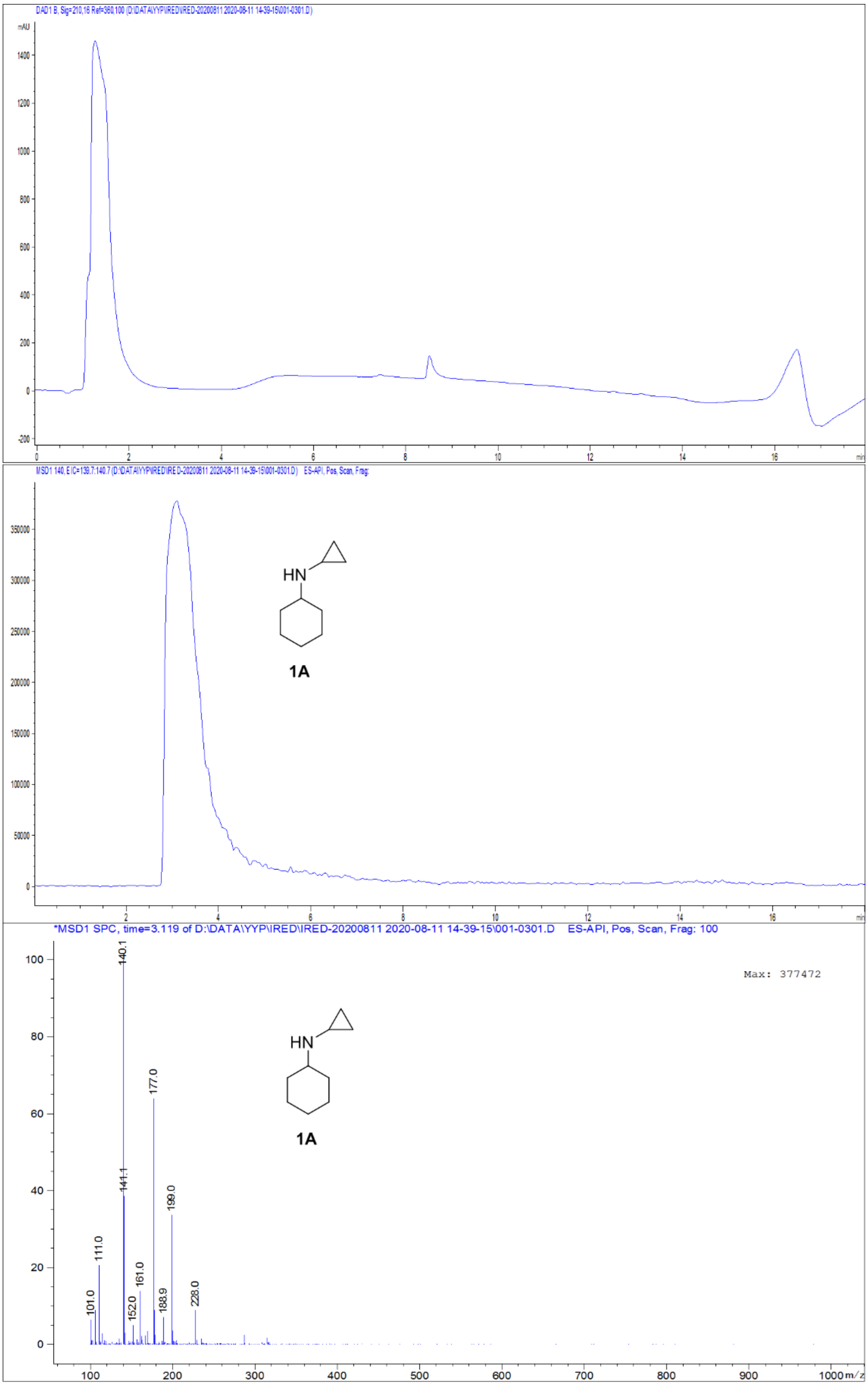

Figure 2. LCMS analysis: IR-G21-catalysed reductive amination of **1** with **A**, showing amine product **1A**.

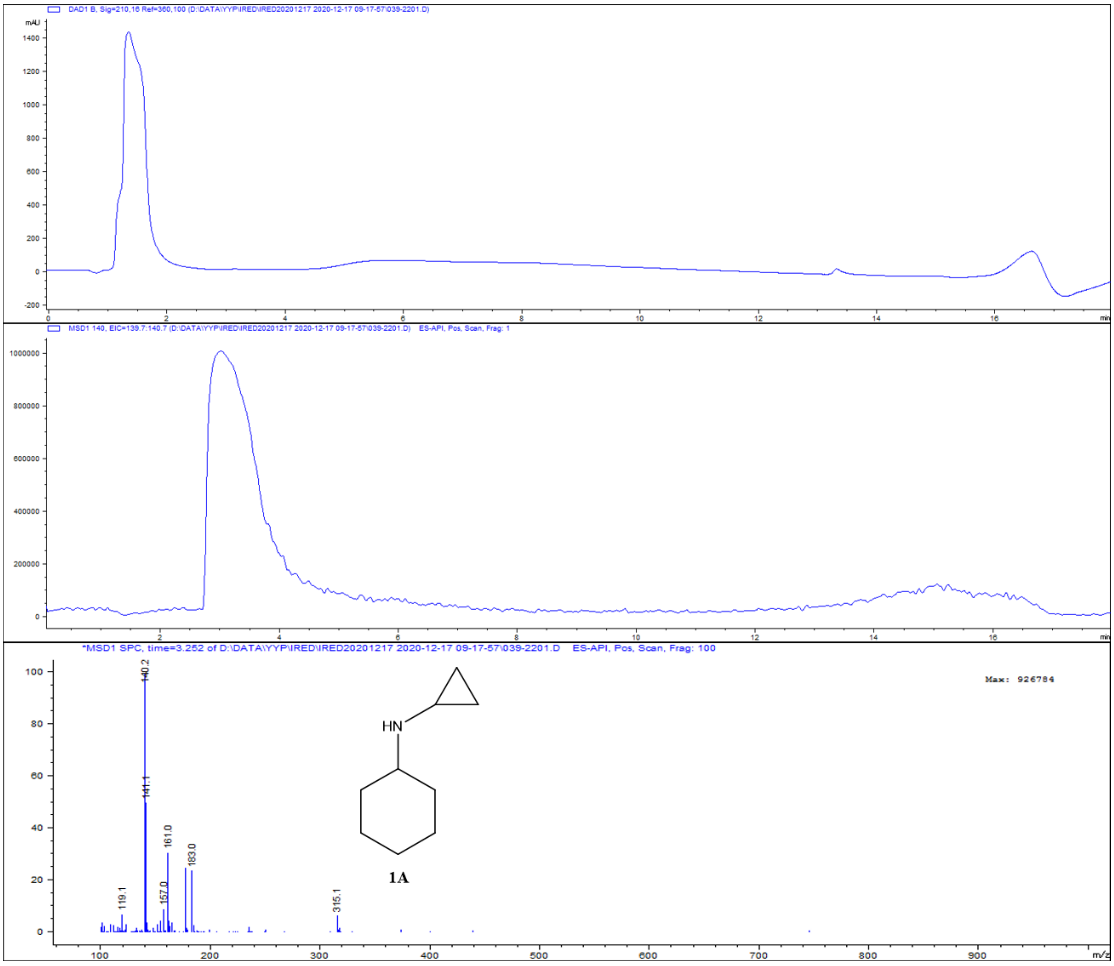

Figure 3. LCMS analysis: IR-G35-catalysed reductive amination of **1** with **A**, showing amine product **1A**.

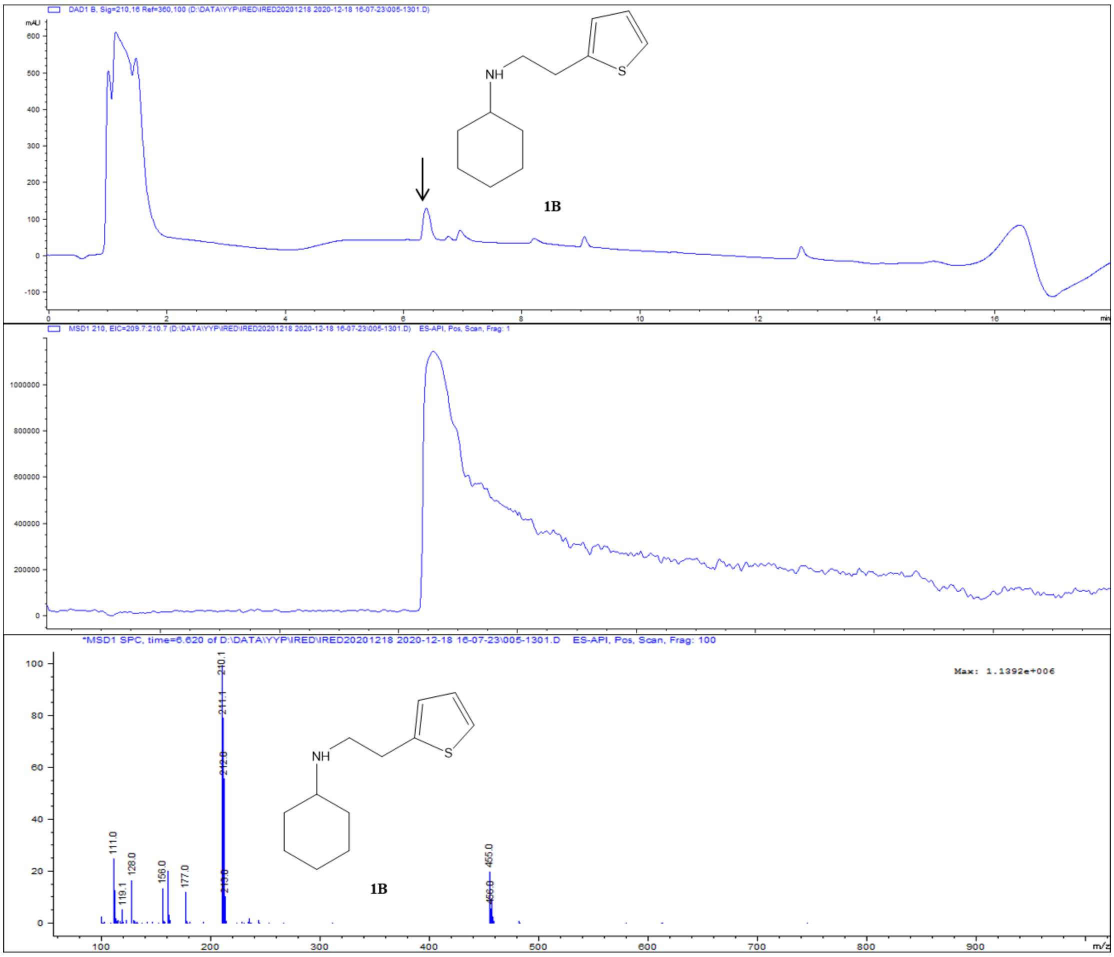

Figure 4. LCMS analysis: IR-G02-catalysed reductive amination of **1** with **B**, showing amine product **1B**.

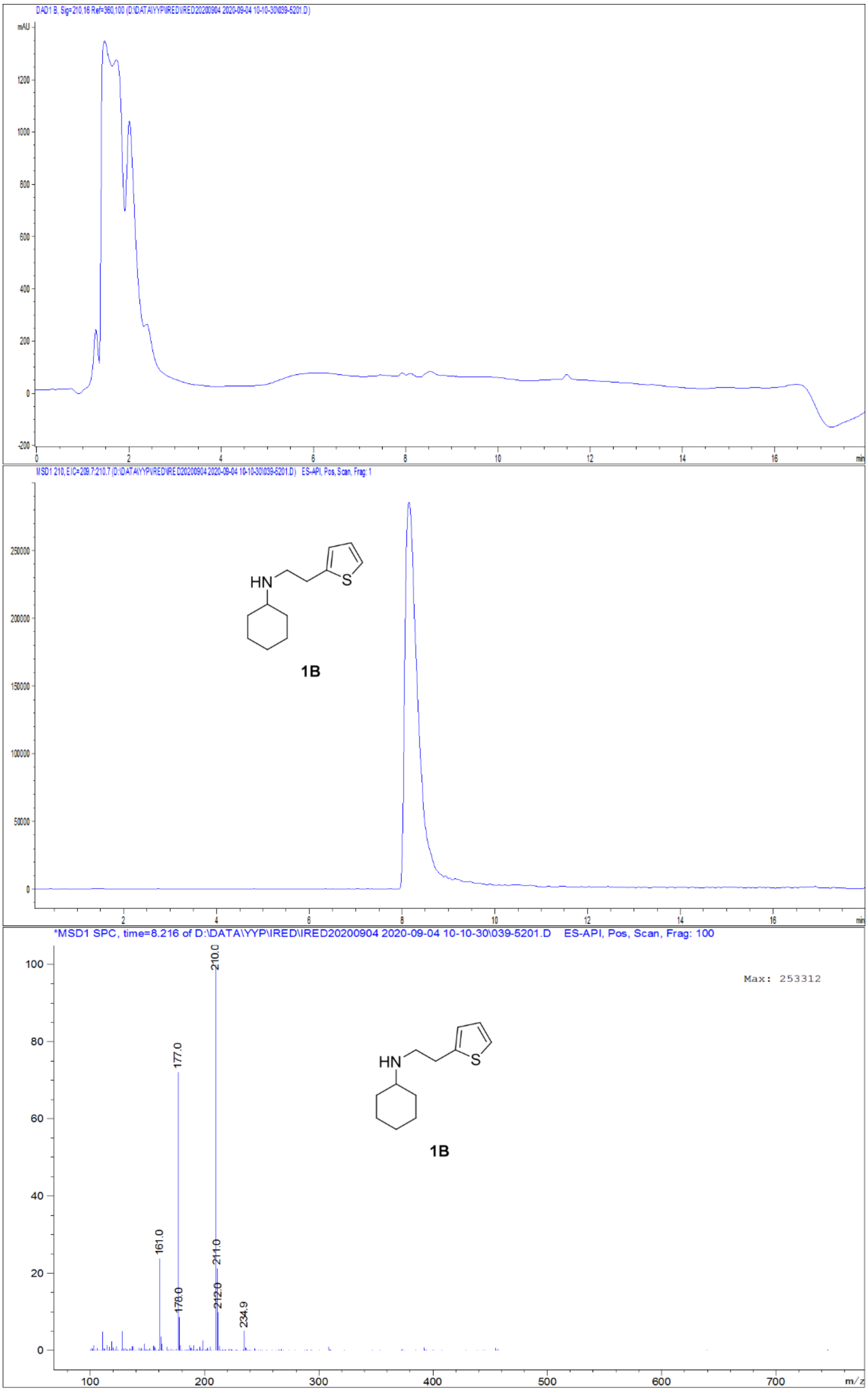

Figure 5. LCMS analysis: IR-G21-catalysed reductive amination of **1** with **B**, showing amine product **1B**.

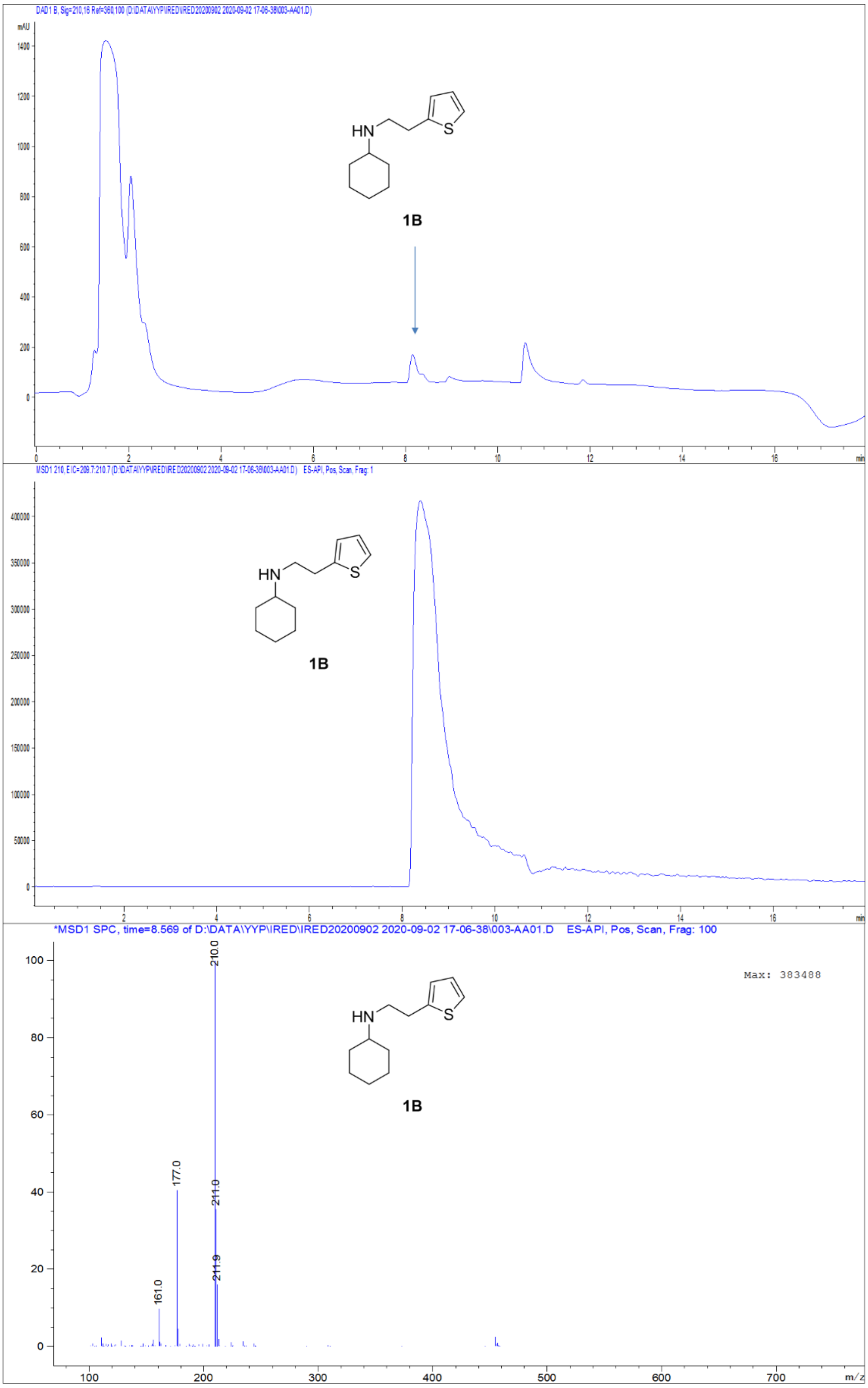

Figure 6. LCMS analysis: IR-G35-catalysed reductive amination of **1** with **B**, showing amine product **1B**.

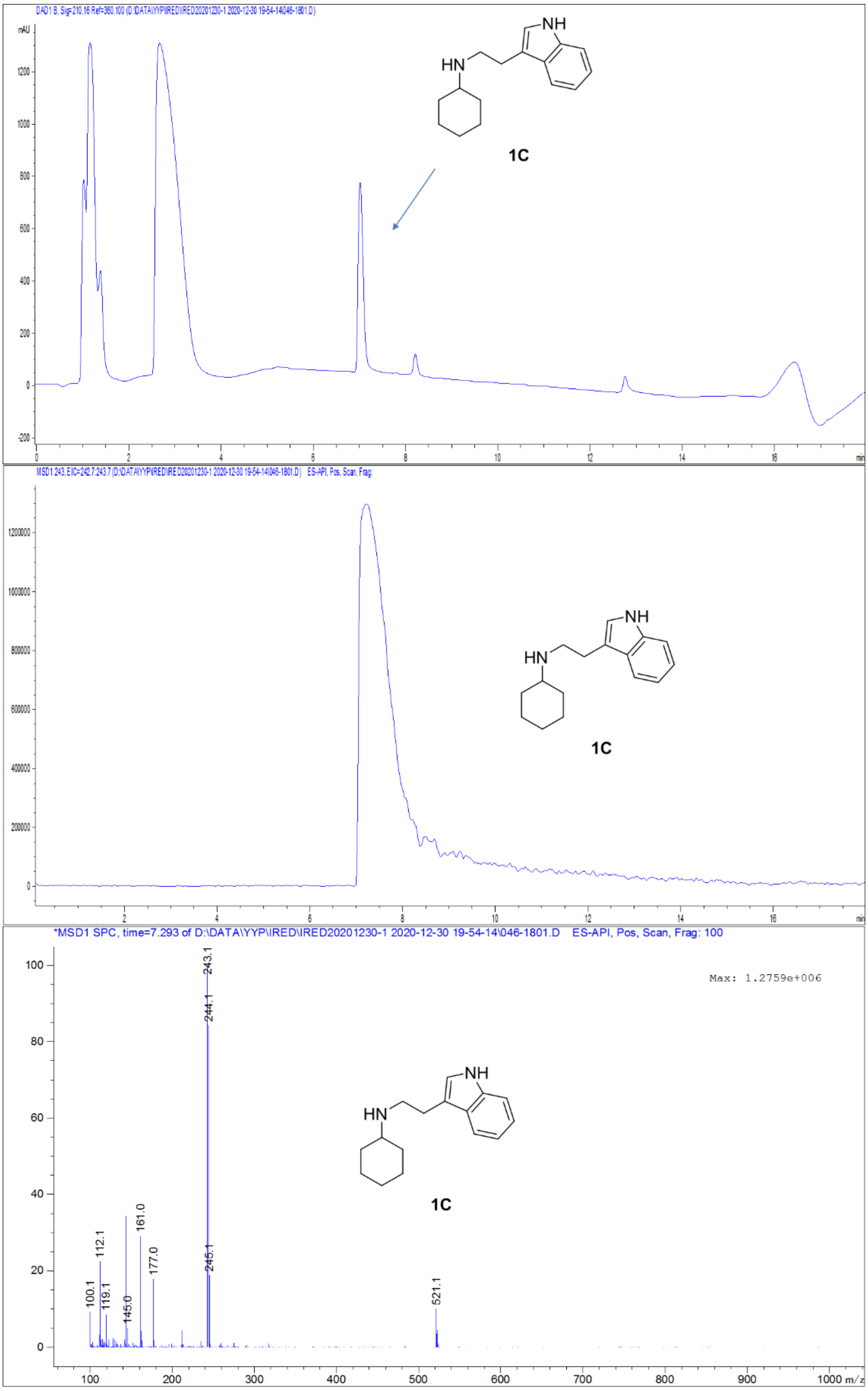

Figure 7. LCMS analysis: IR-G02-catalysed reductive amination of **1** with **C**, showing amine product **1C**.

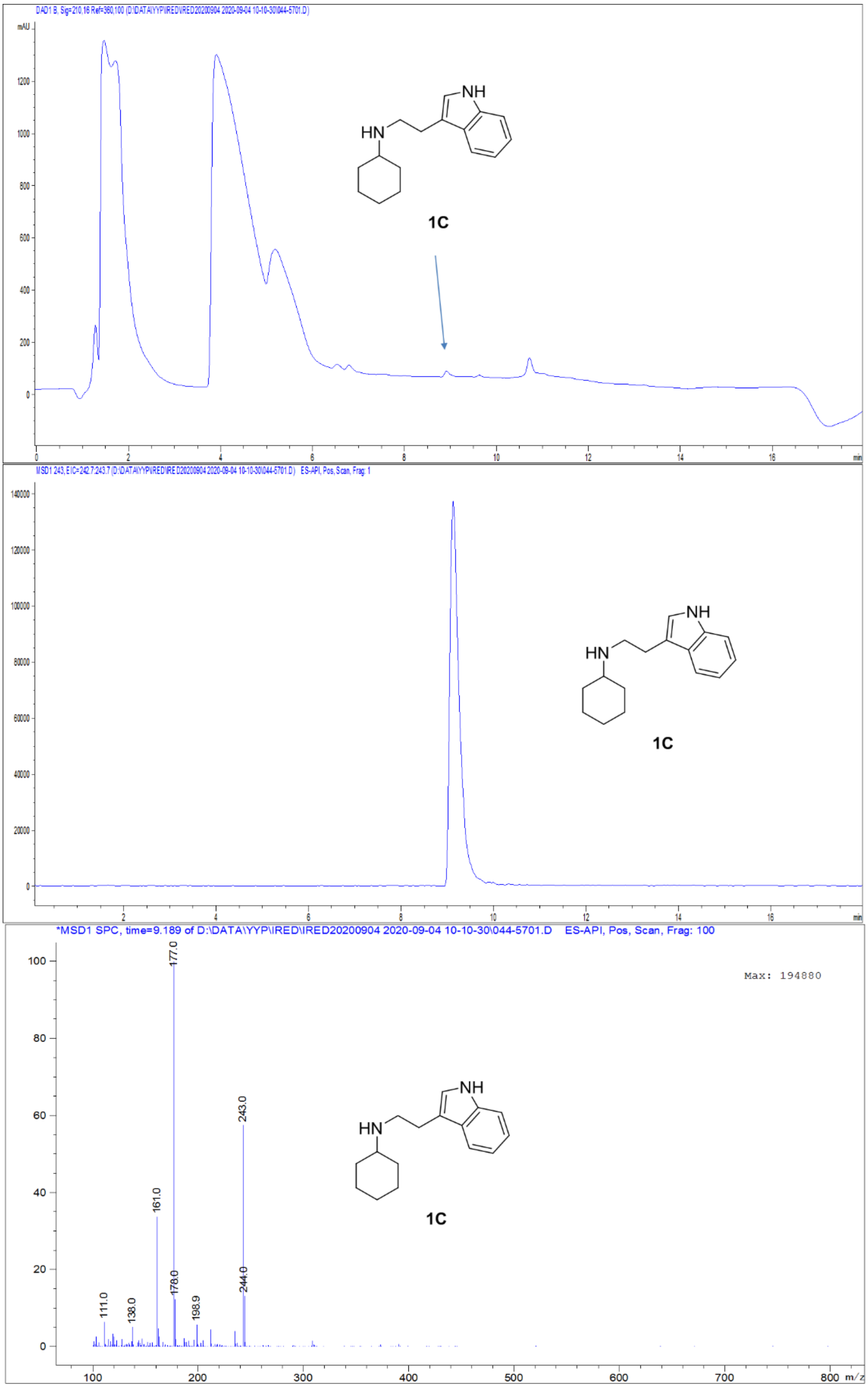

Figure 8. LCMS analysis: IR-G21-catalysed reductive amination of **1** with **C**, showing amine product **1C**.

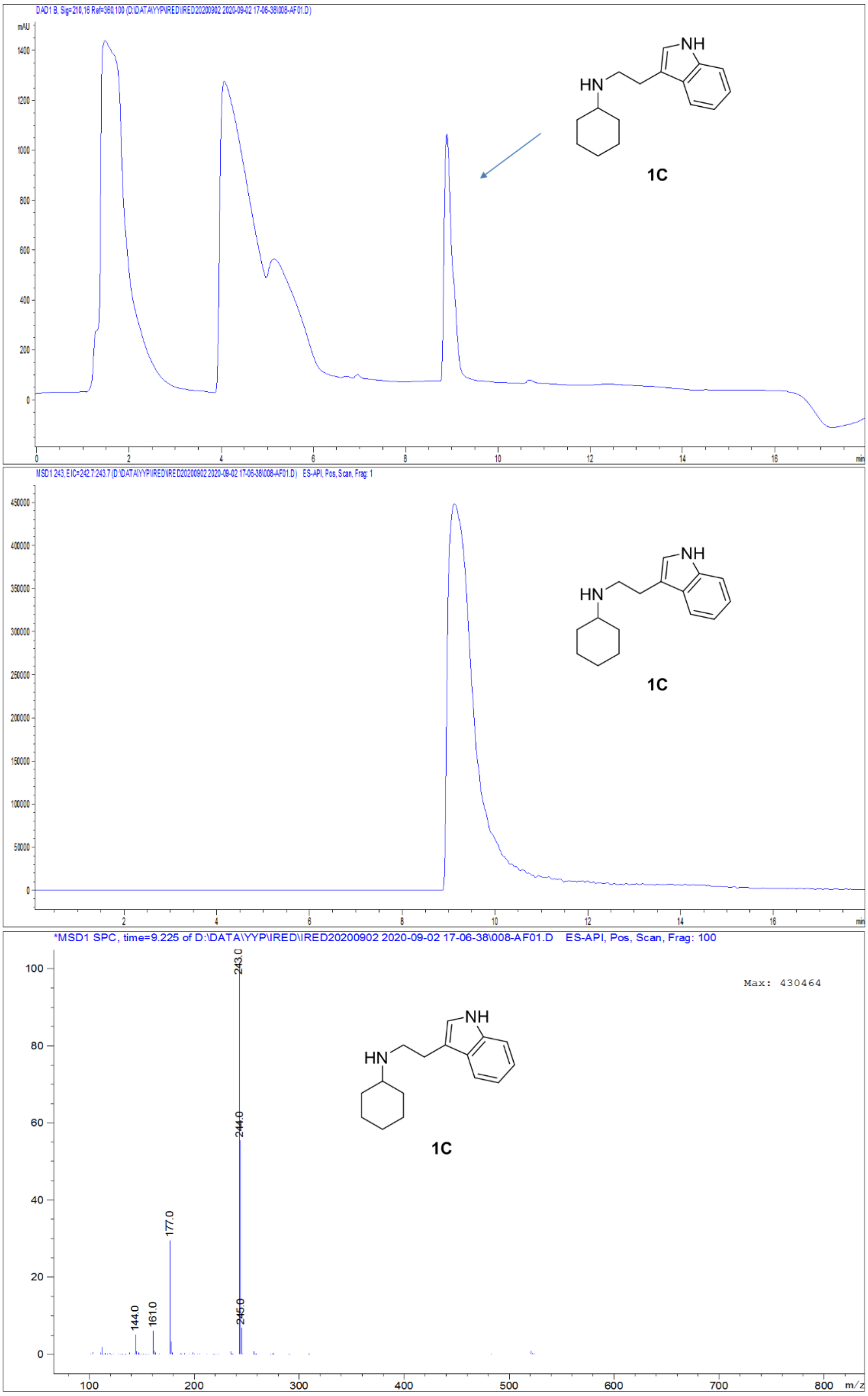

Figure 9. LCMS analysis: IR-G35-catalysed reductive amination of **1** with **C**, showing amine product **1C**.

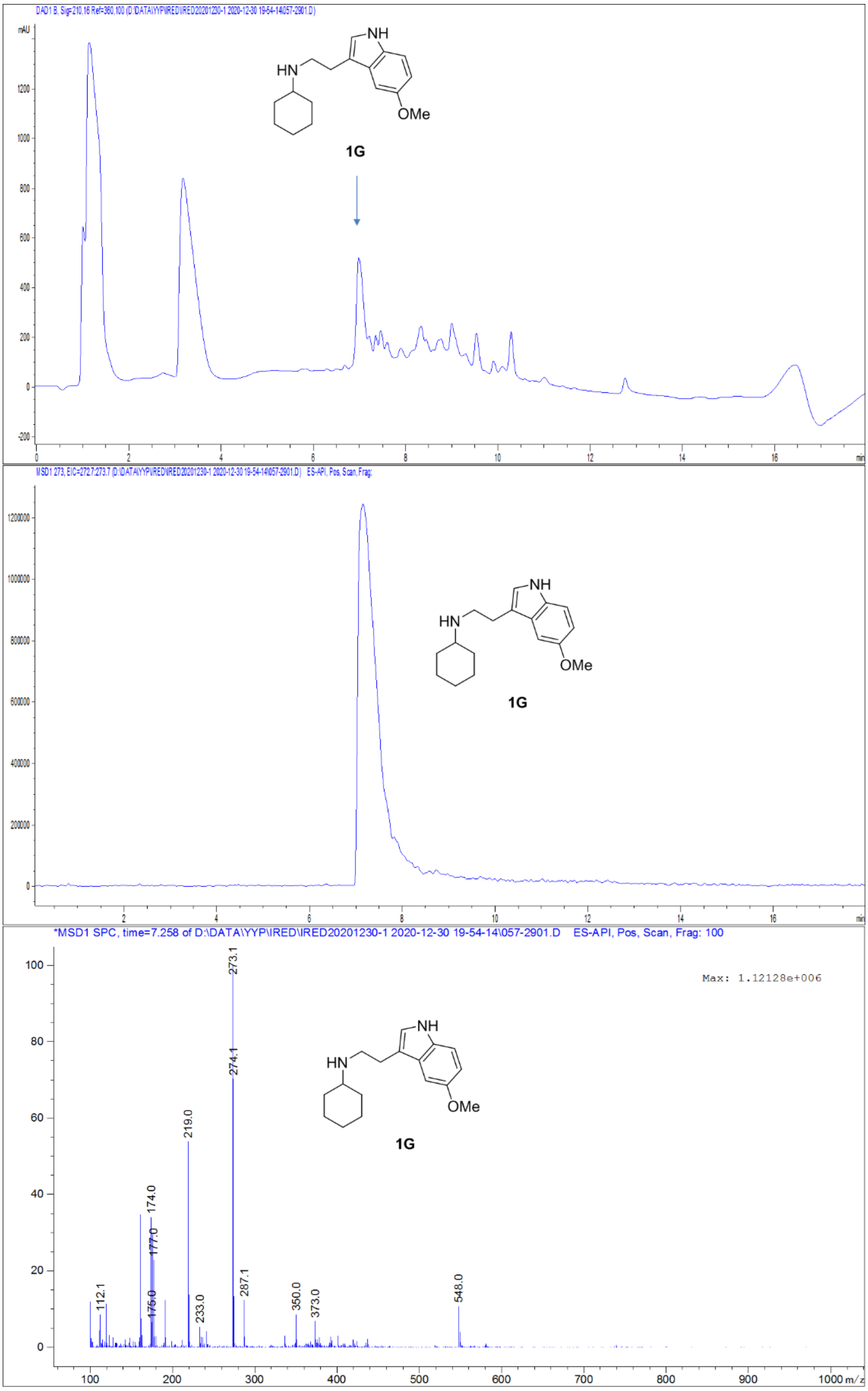

Figure 10. LCMS analysis: IR-G02-catalysed reductive amination of **1** with **G**, showing amine product **1G**.

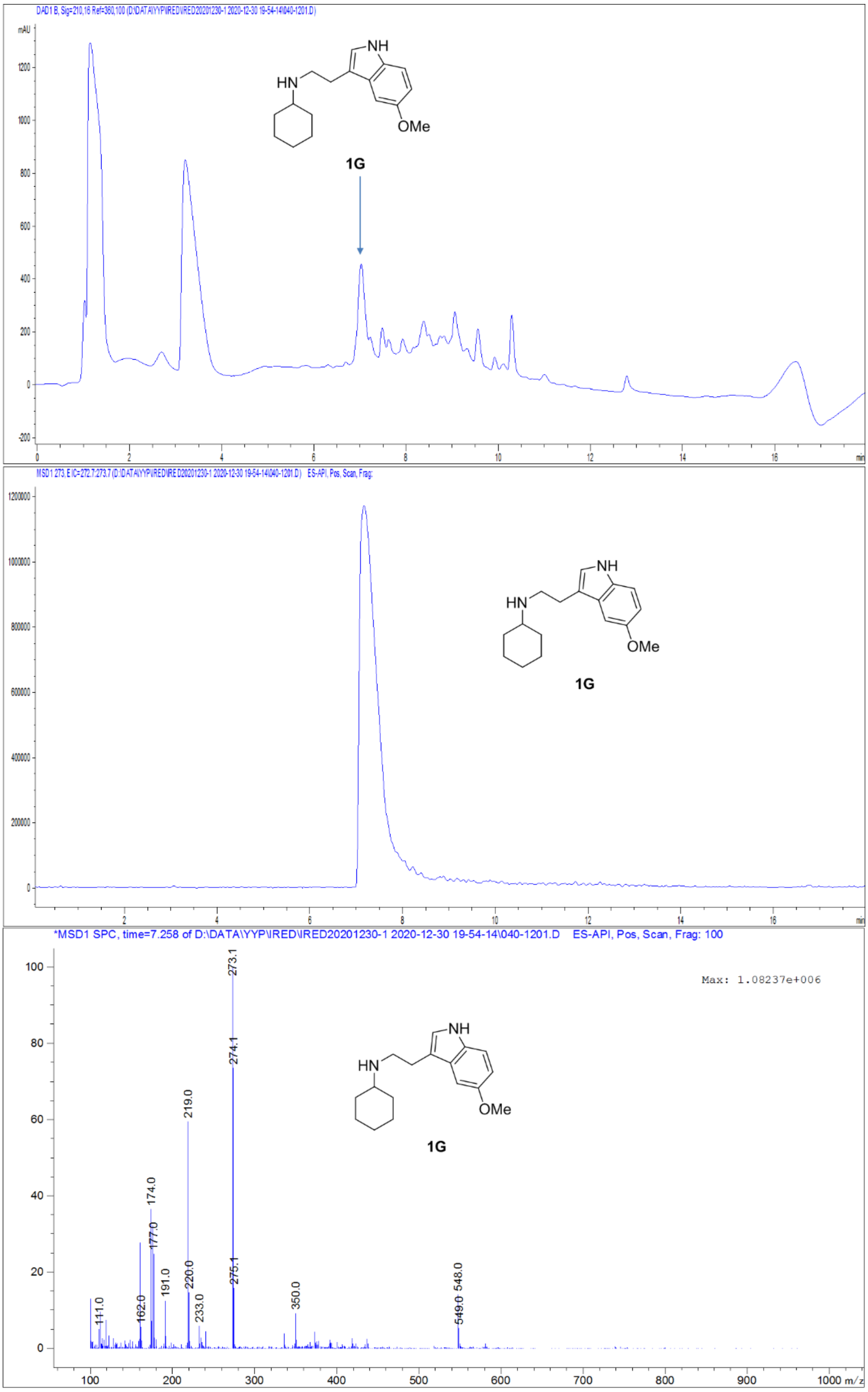

Figure 11. LCMS analysis: IR-G21-catalysed reductive amination of **1** with **G**, showing amine product **1G**.

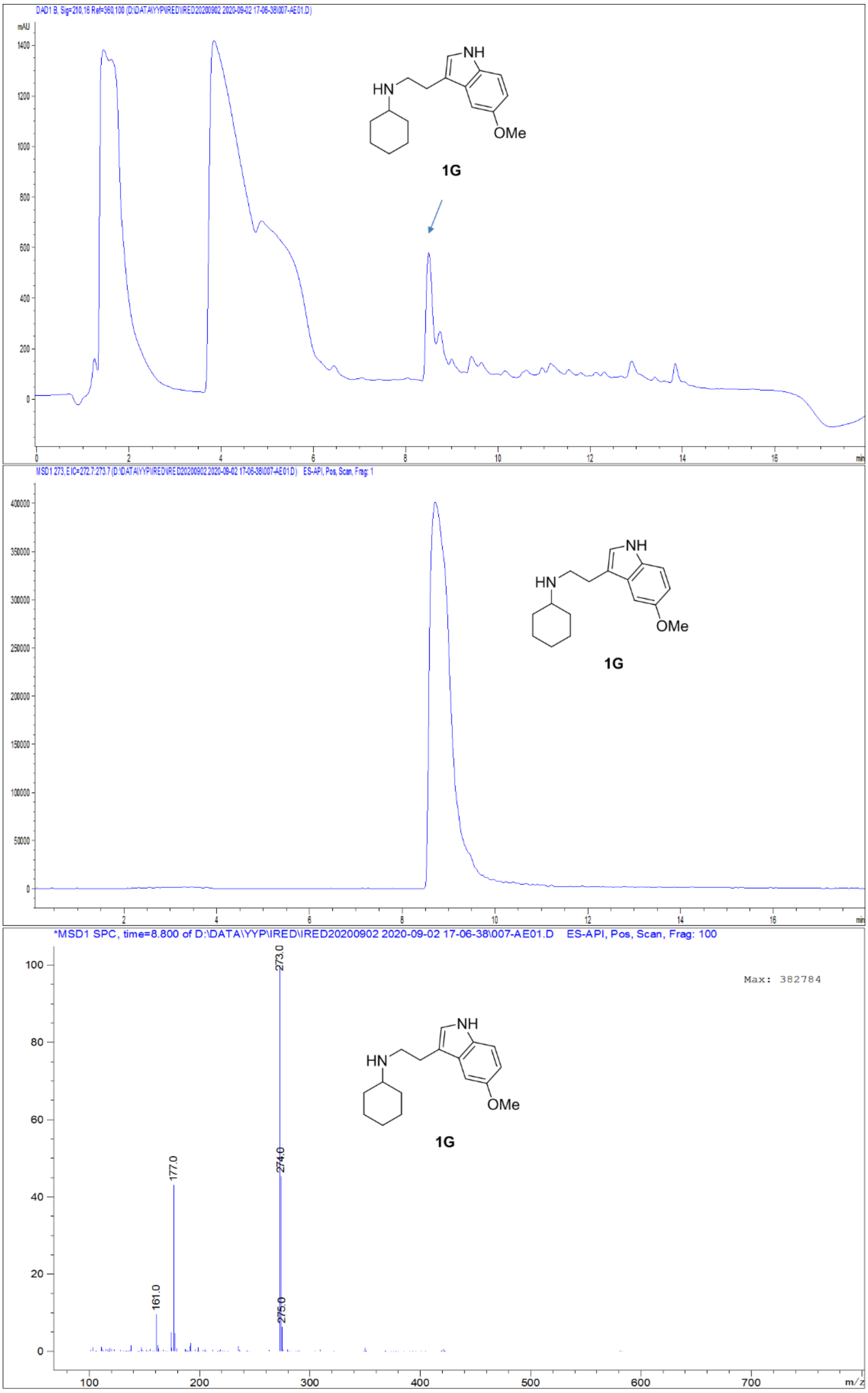

Figure 12. LCMS analysis: IR-G35-catalysed reductive amination of **1** with **G**, showing amine product **1G**.

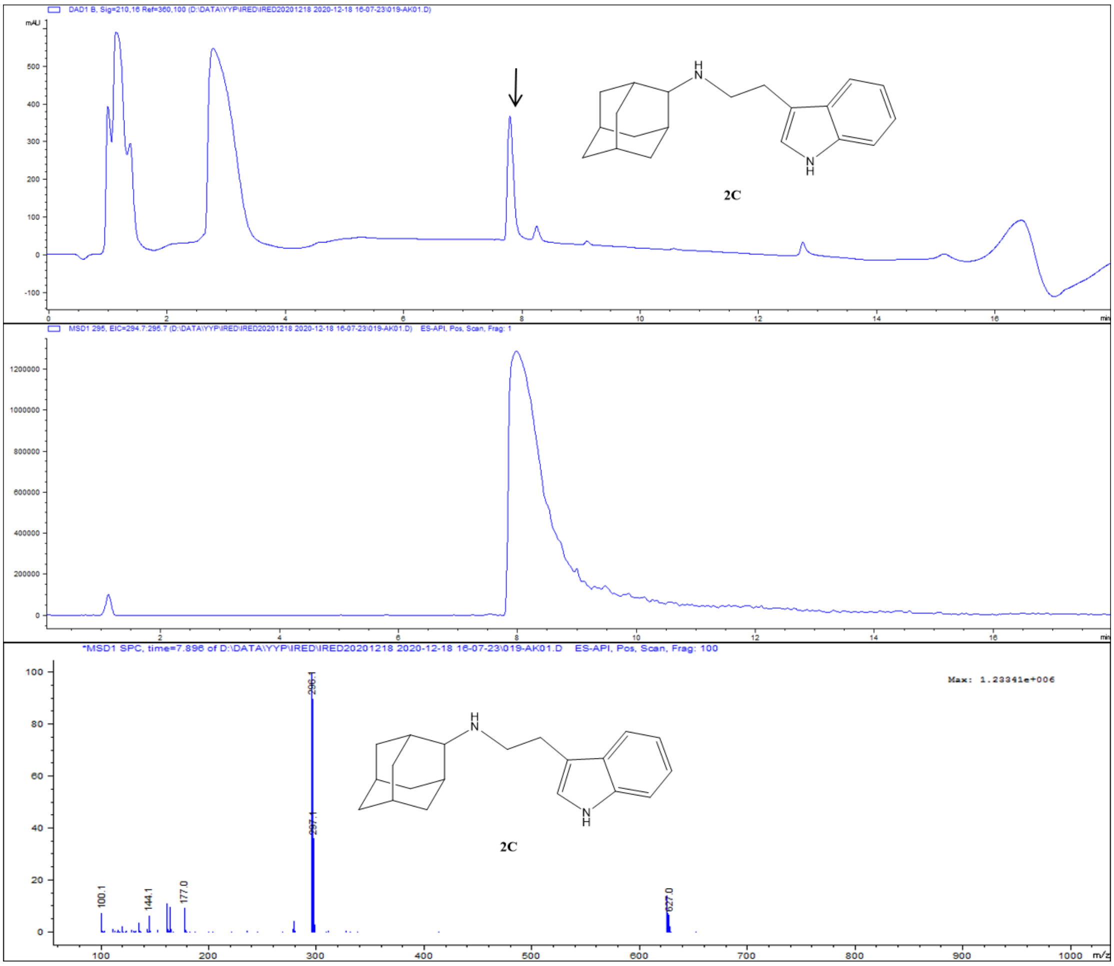

Figure 13. LCMS analysis: IR-G02-catalysed reductive amination of **2** with **C**, showing amine product **2C**.

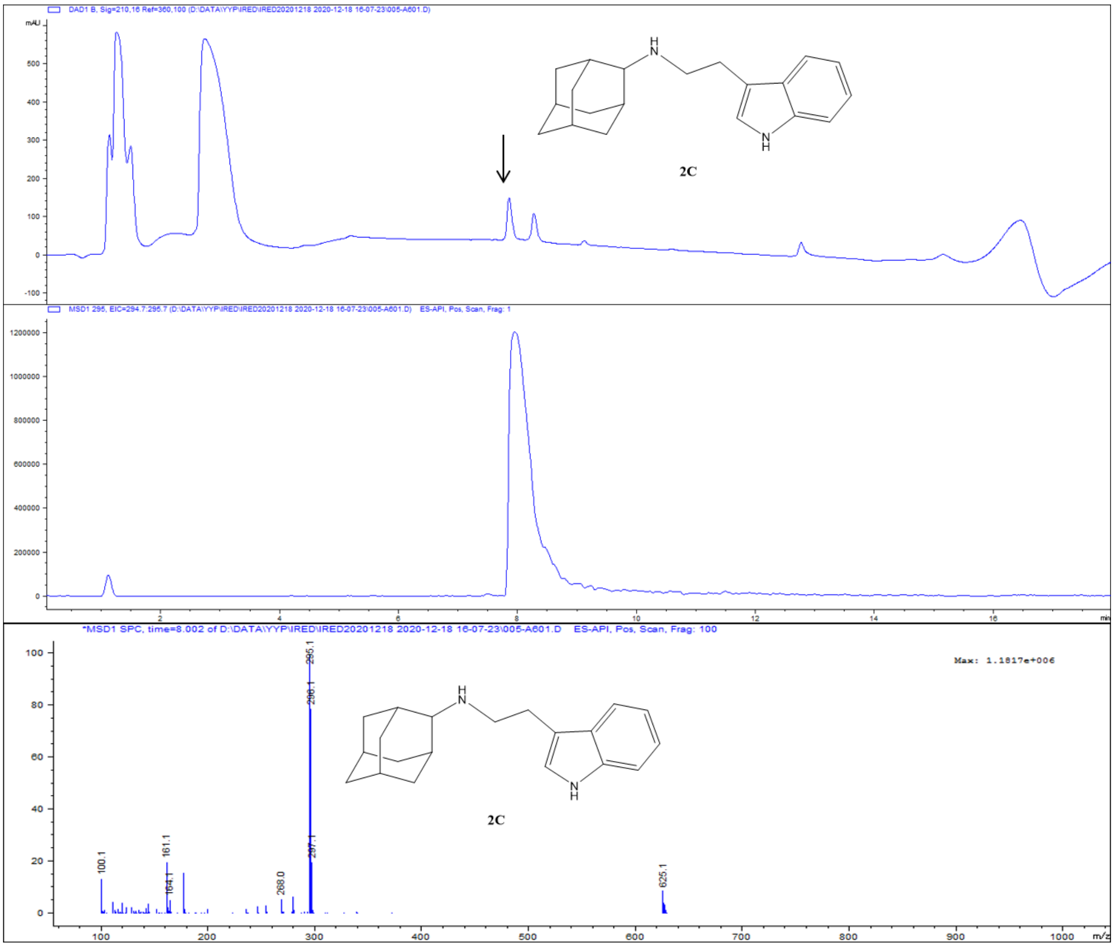

Figure 14. LCMS analysis: IR-G21-catalysed reductive amination of **2** with **C**, showing amine product **2C**.

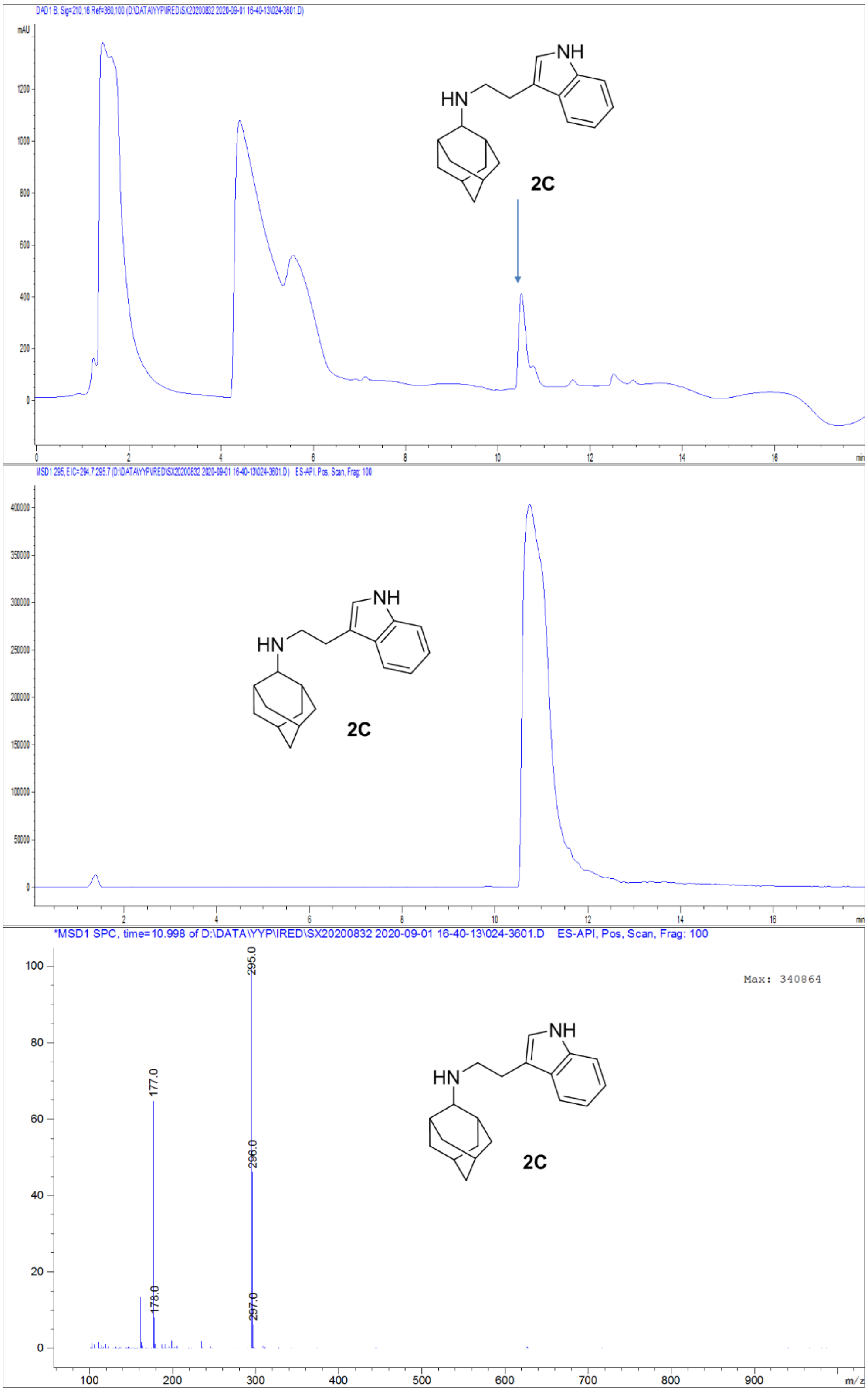

Figure 15. LCMS analysis: IR-G35-catalysed reductive amination of **2** with **C**, showing amine product **2C**.

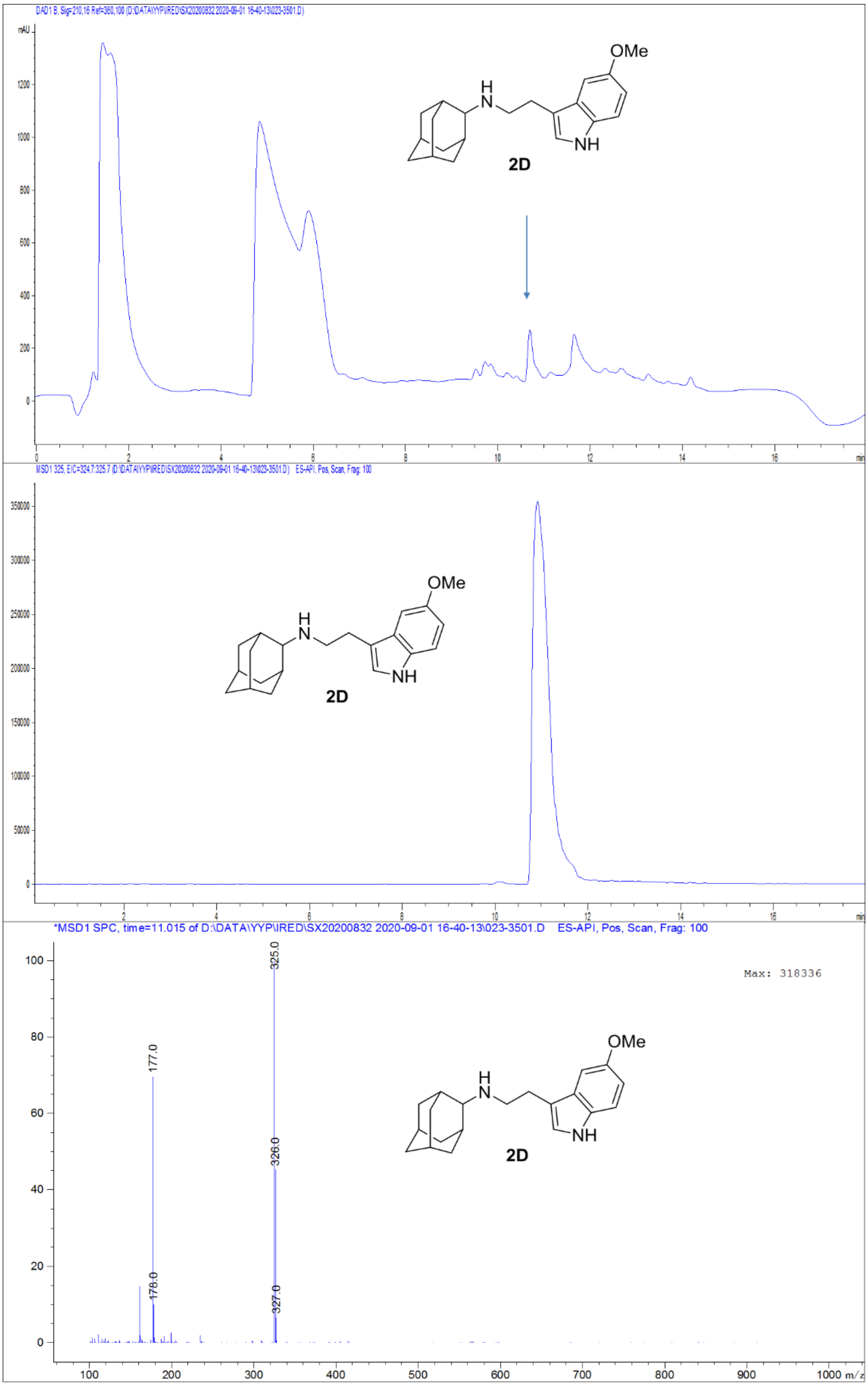

Figure 16. LCMS analysis: IR-G02-catalysed reductive amination of **2** with **D**, showing amine product **2D**.

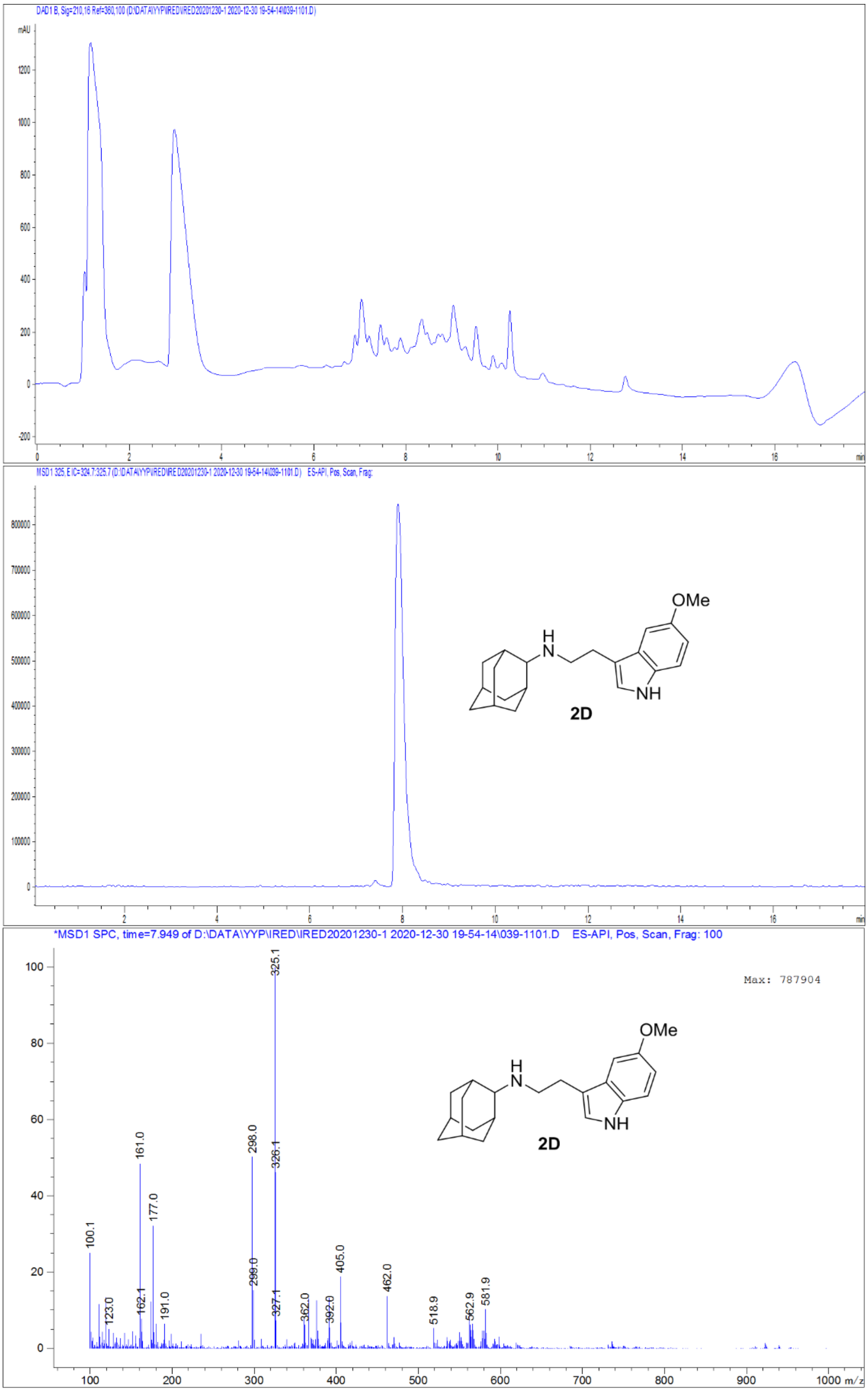

Figure 17. LCMS analysis: IR-G35atalysed reductive amination of **2** with **D**, showing amine product **2D**.

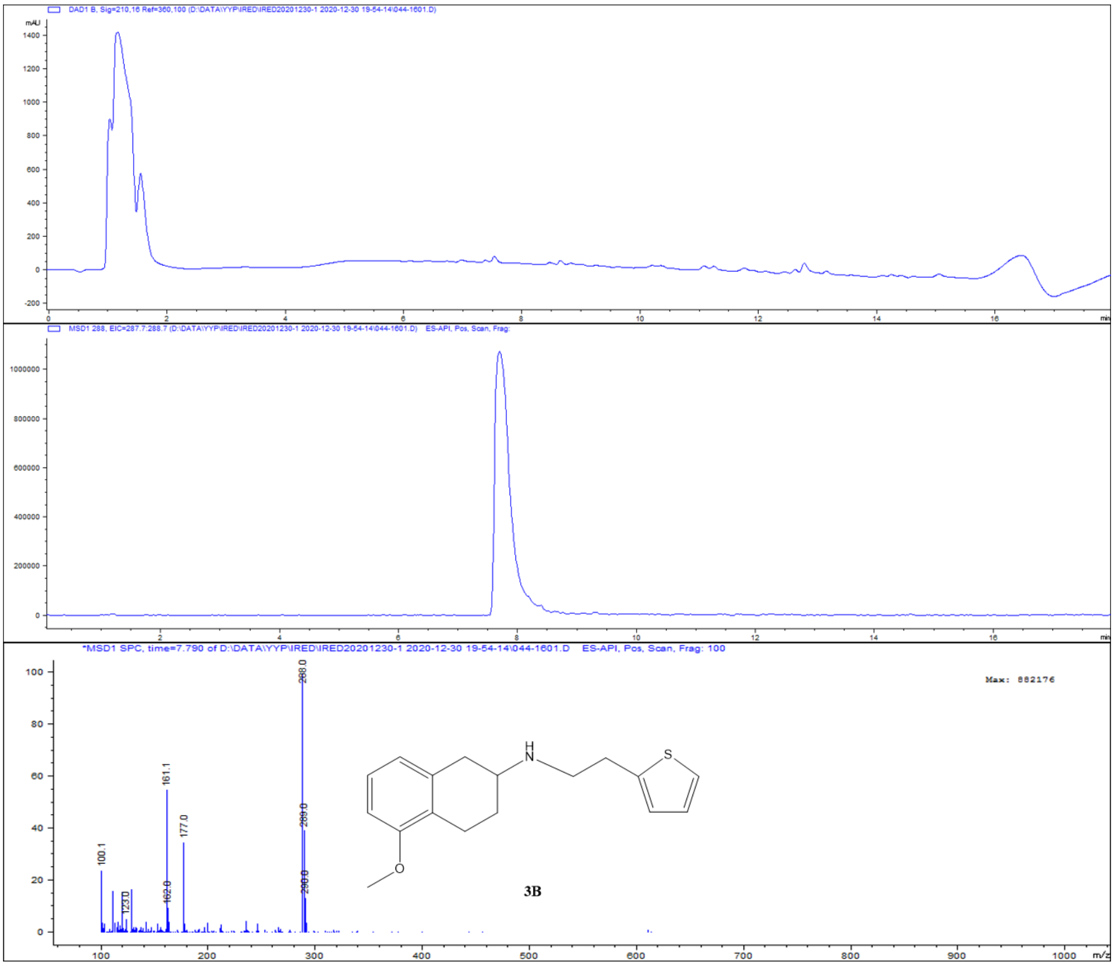

Figure 18. LCMS analysis: IR-G02-catalysed reductive amination of **3** with **B**, showing amine product **3B**.

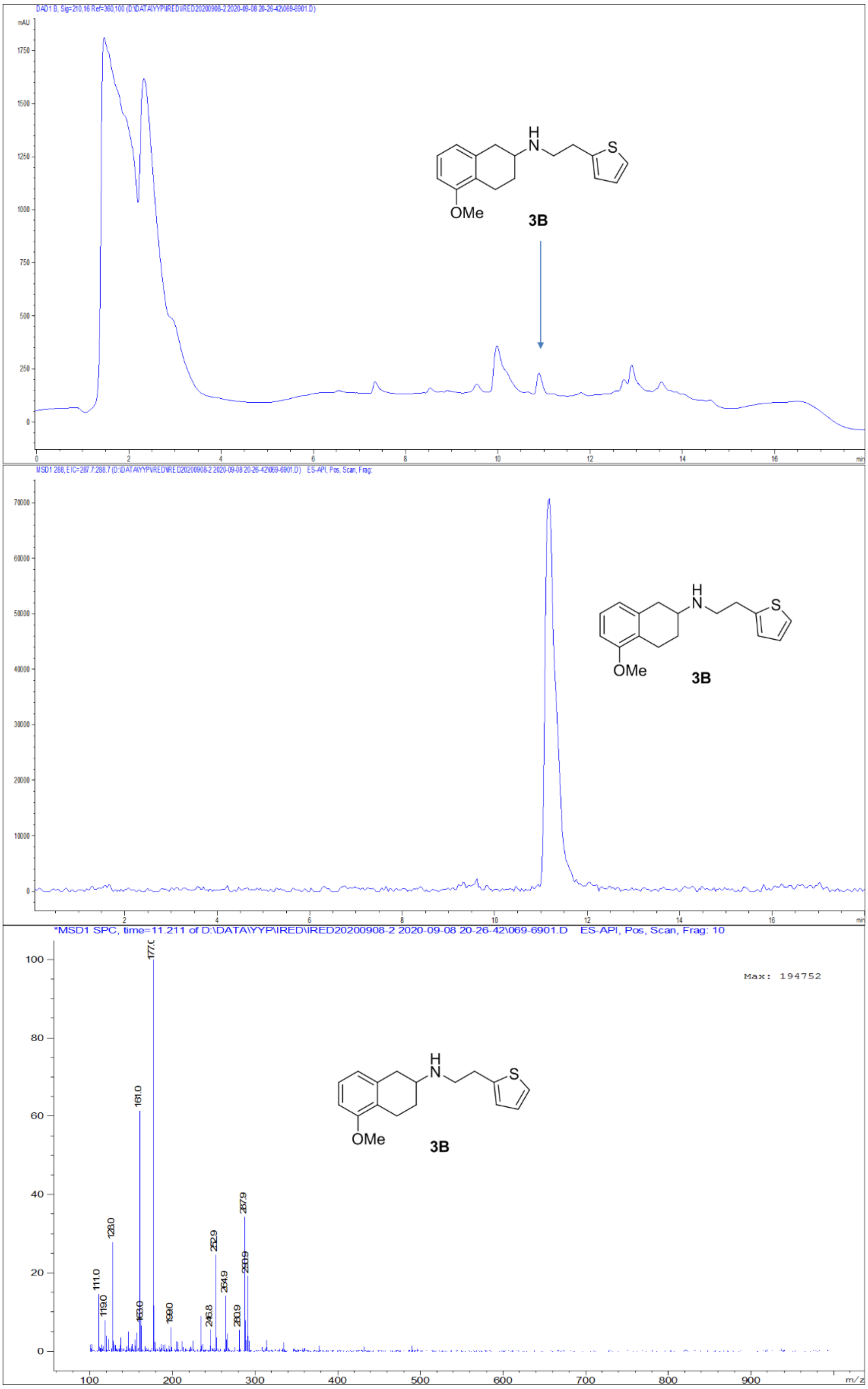

Figure 19. LCMS analysis: IR-G21-catalysed reductive amination of **3** with **B**, showing amine product **3B**.

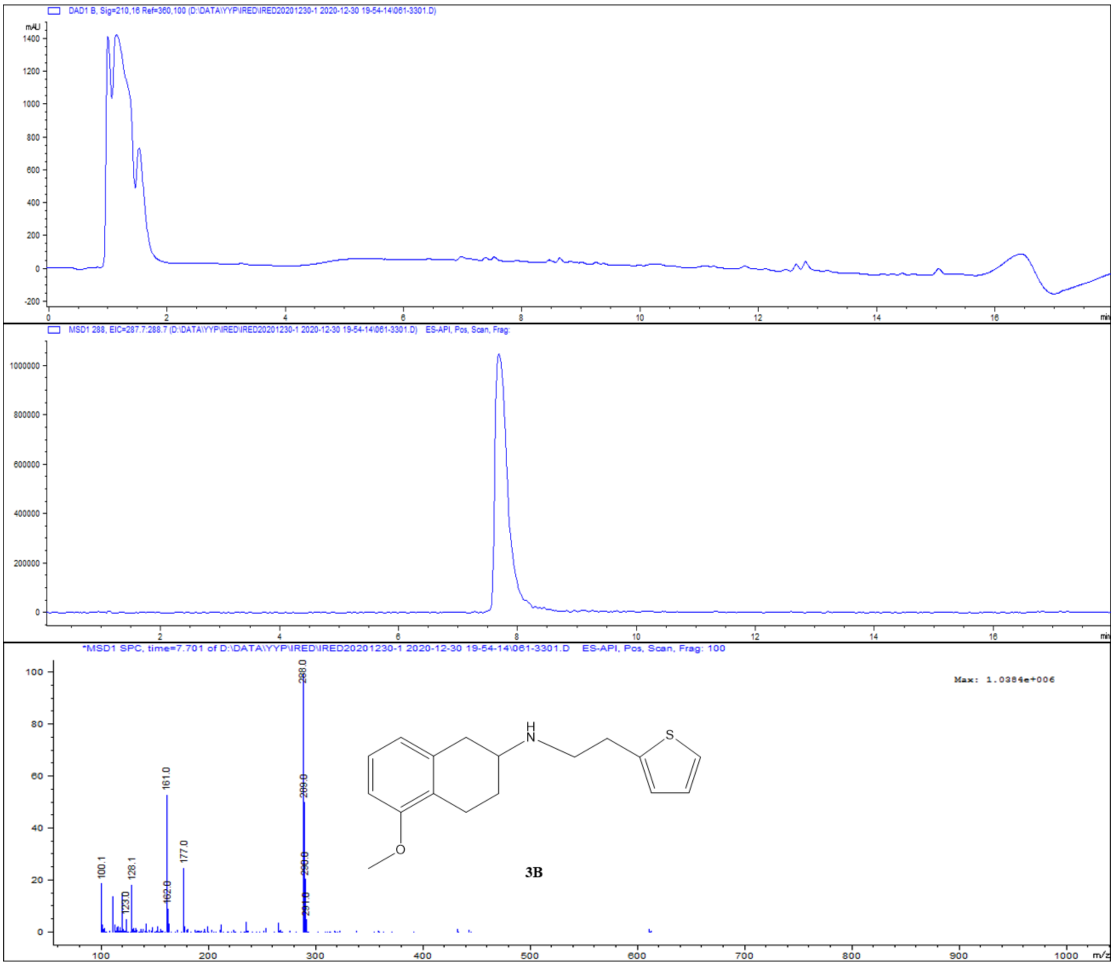

Figure 20. LCMS analysis: IR-G35-catalysed reductive amination of **3** with **B**, showing amine product **3B**.

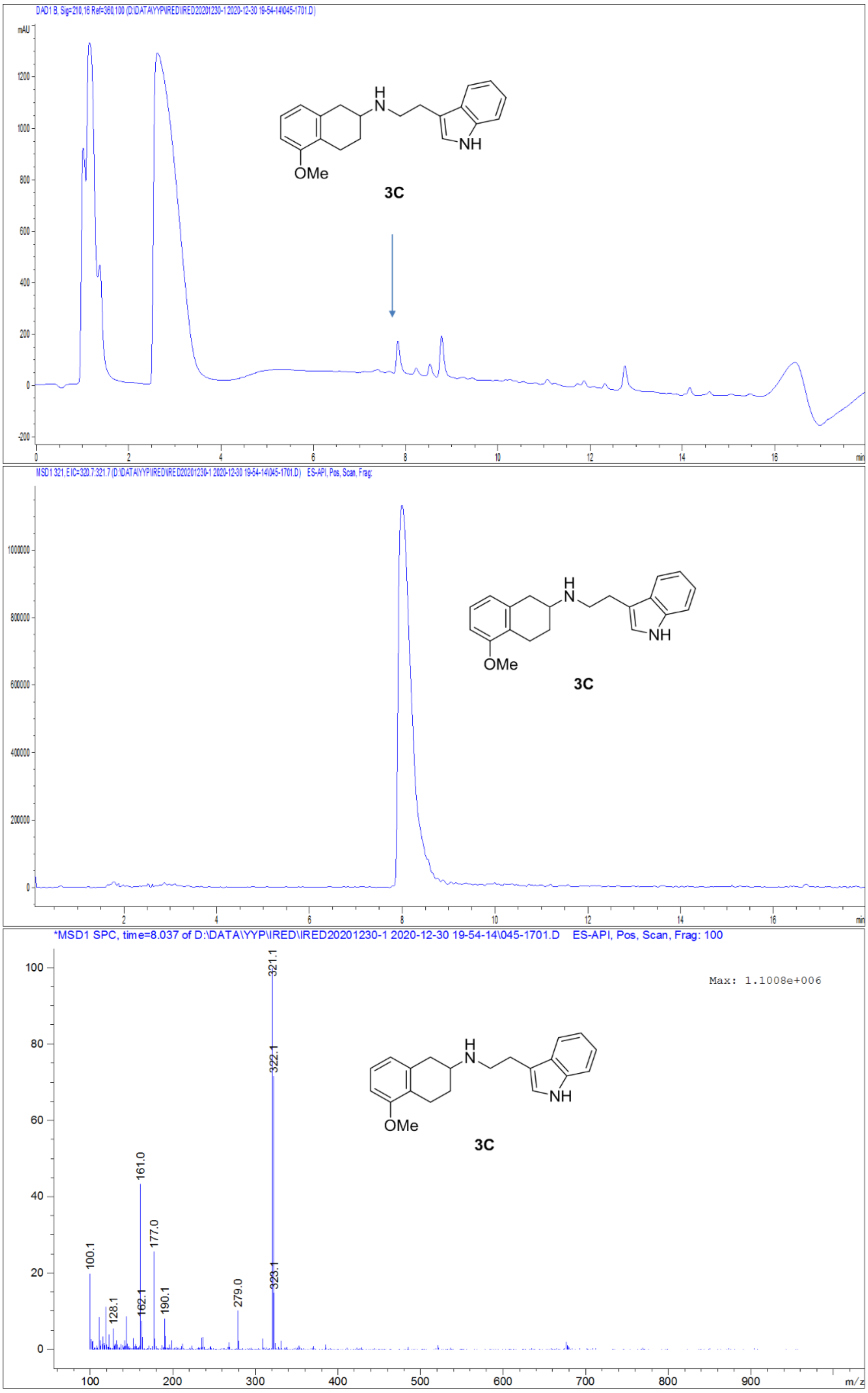

Figure 21. LCMS analysis: IR-G021-catalysed reductive amination of **3** with **C**, showing amine product **3C**.

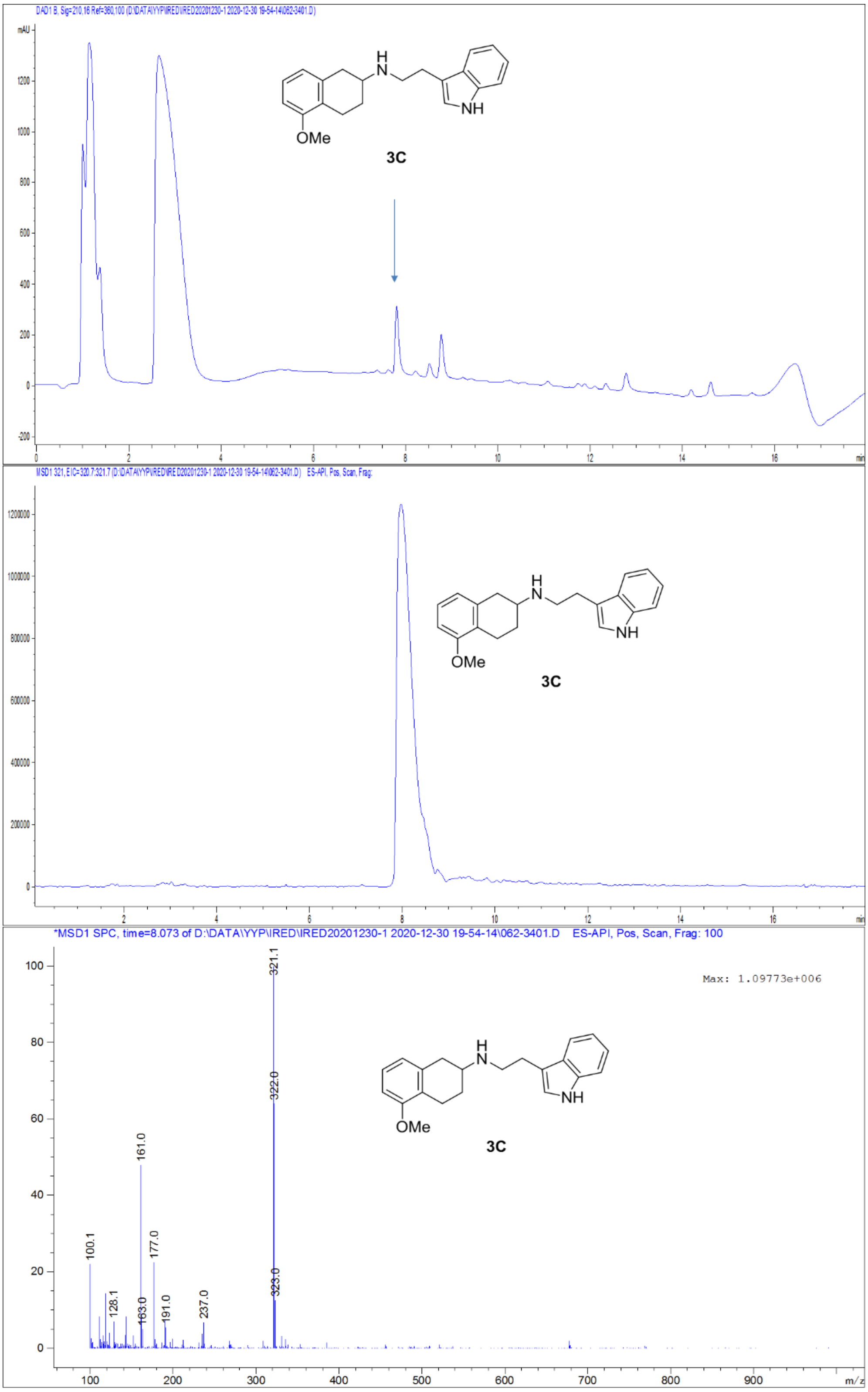

Figure 22. LCMS analysis: IR-G035-catalysed reductive amination of **3** with **C**, showing amine product **3C**.

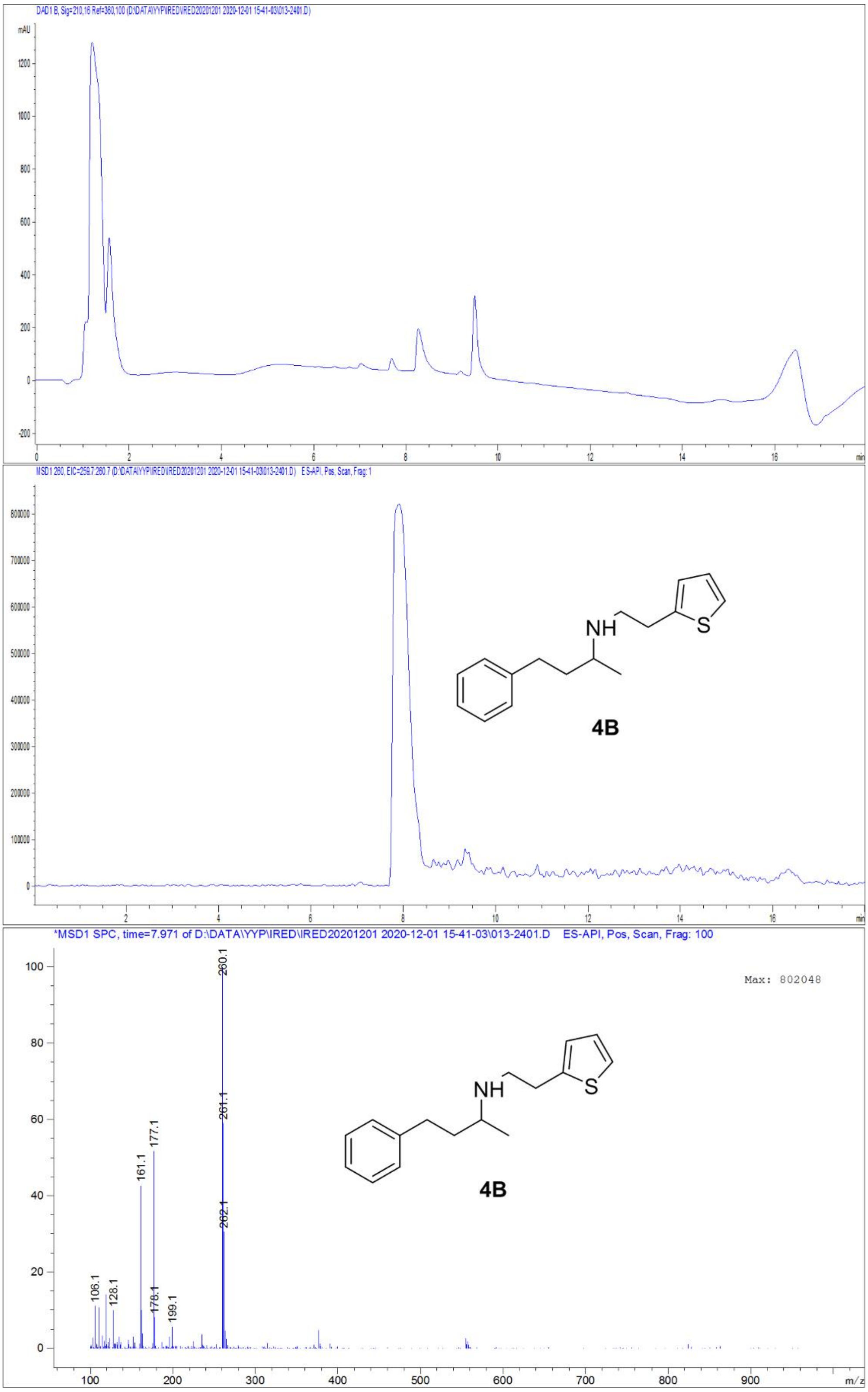

Figure 23. LCMS analysis: IR-G02-catalysed reductive amination of **4** with **B**, showing amine product **4B**.

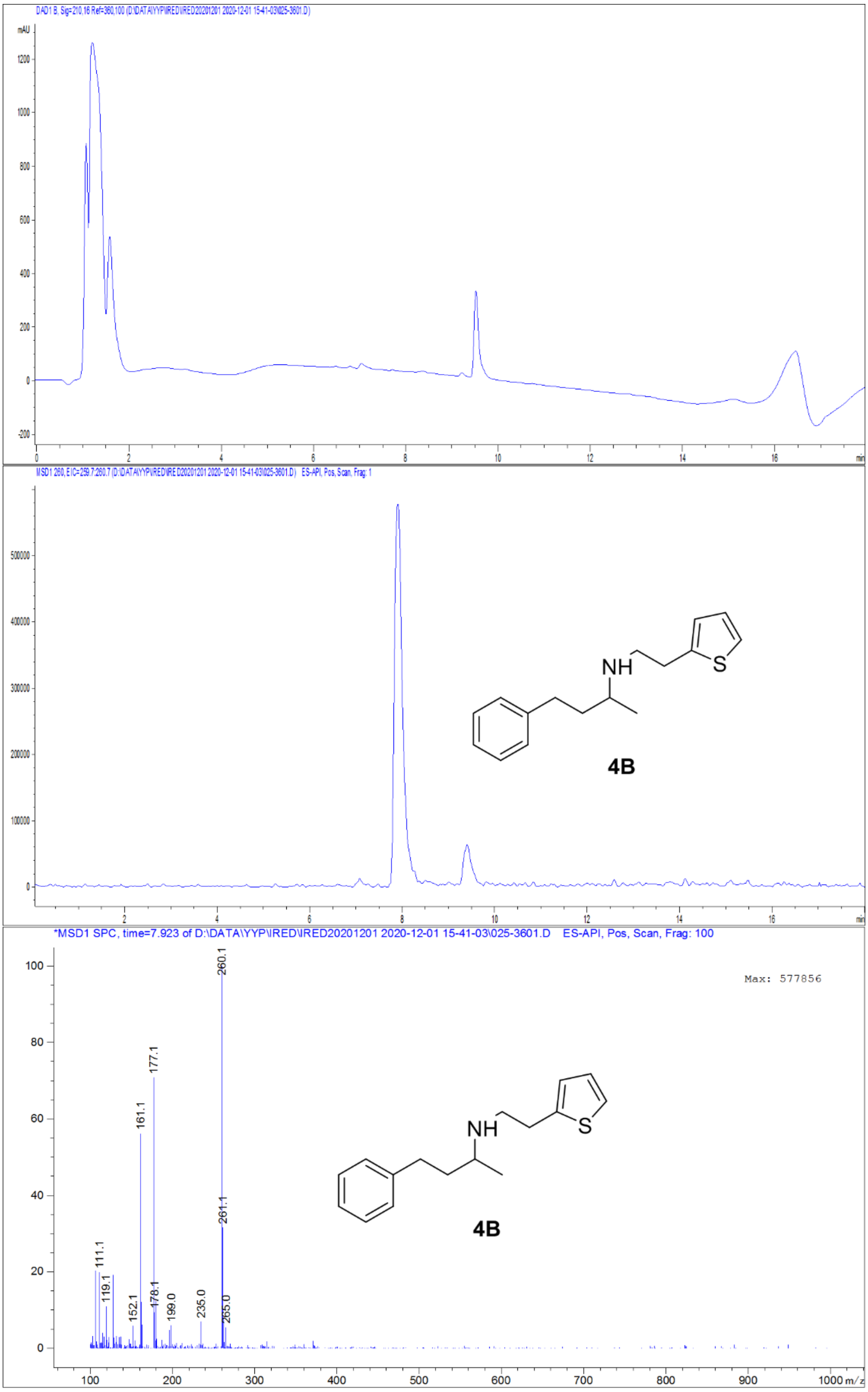

Figure 24. LCMS analysis: IR-G35-catalysed reductive amination of **4** with **B**, showing amine product **4B**.

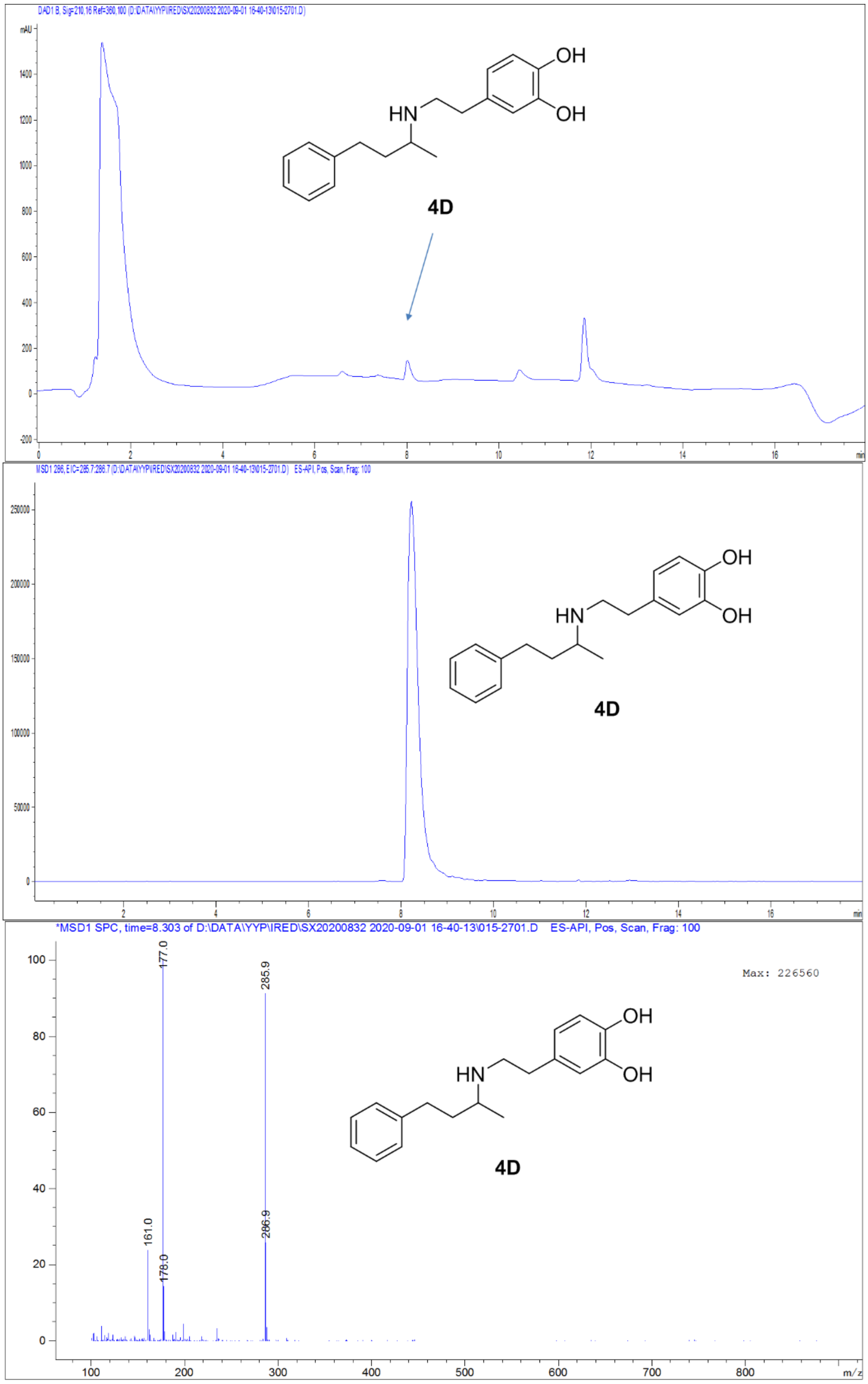

Figure 25. LCMS analysis: IR-G02-catalysed reductive amination of **4** with **D**, showing amine product **4D**.

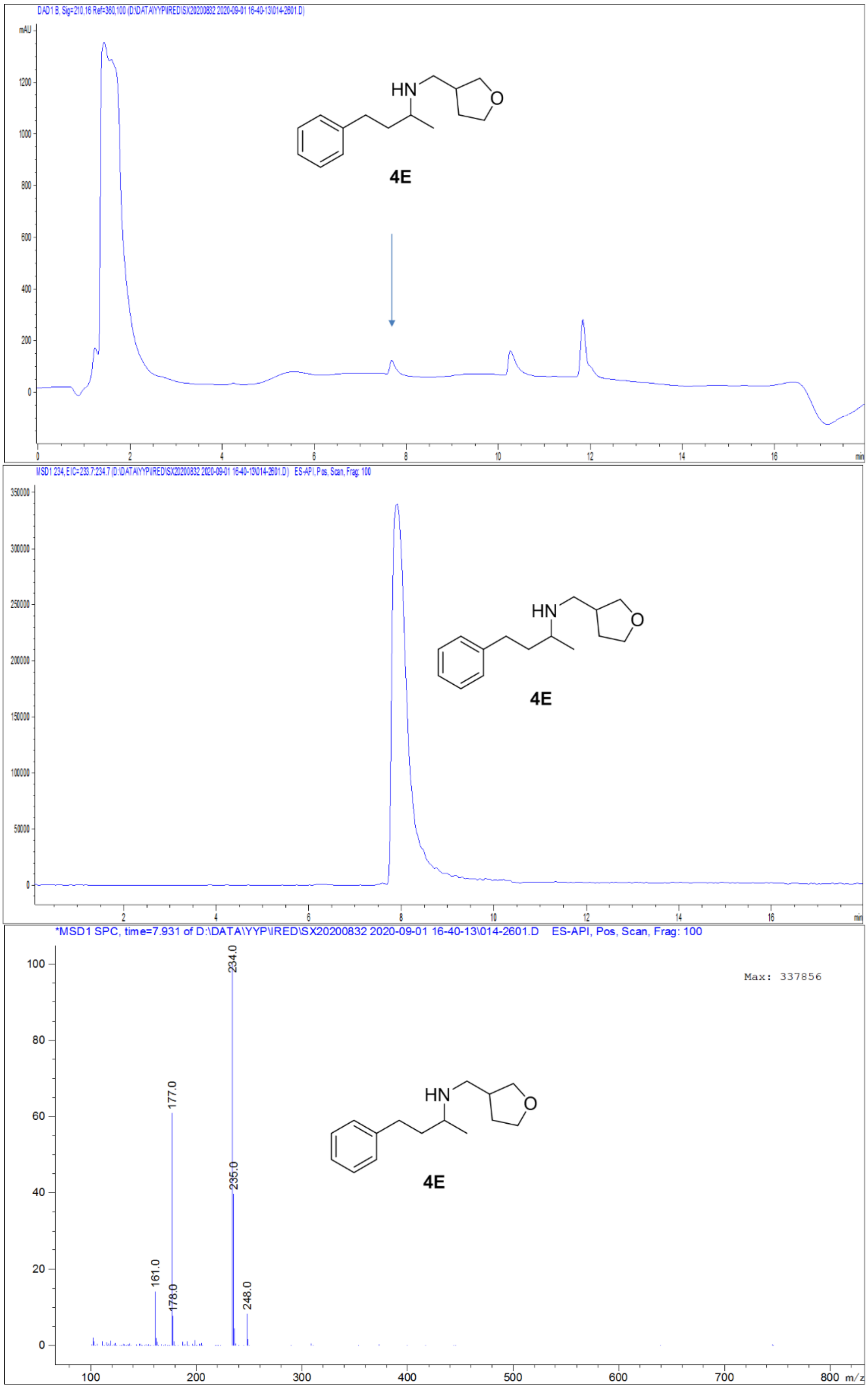

Figure 26. LCMS analysis: IR-G02-catalysed reductive amination of **4** with **E**, showing amine product **4E**.

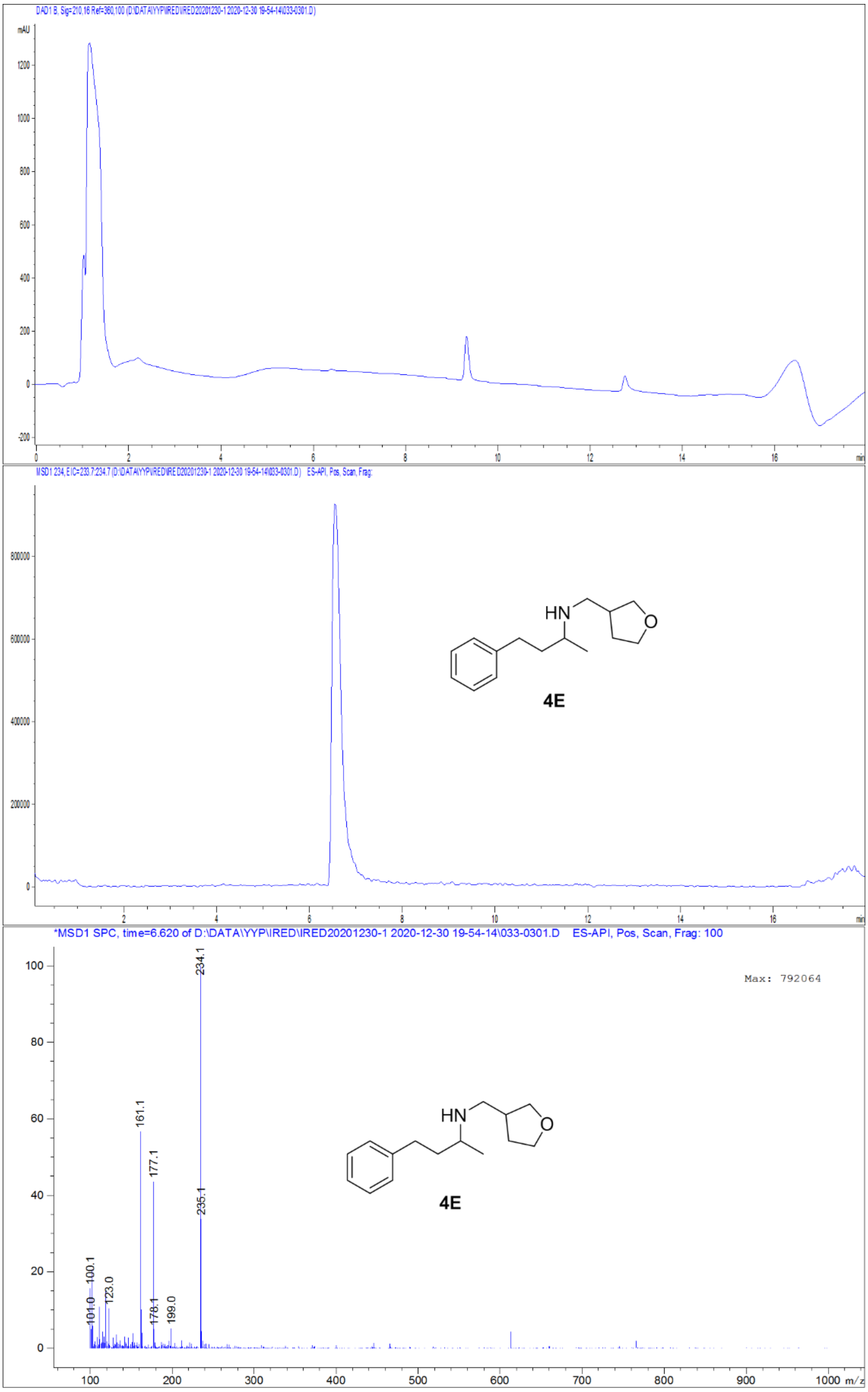

Figure 27. LCMS analysis: IR-G21-catalysed reductive amination of **4** with **E**, showing amine product **4E**.

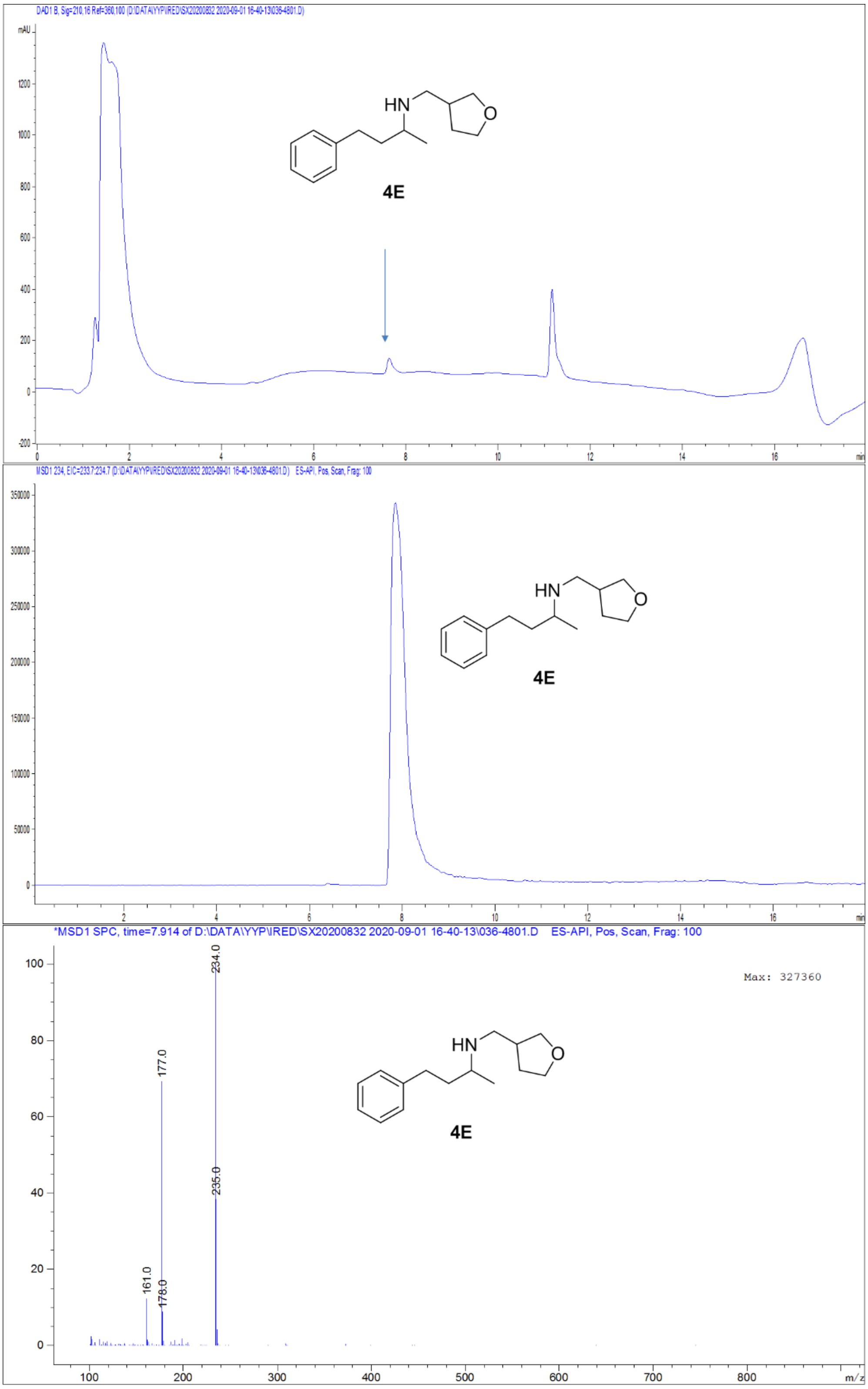

Figure 28. LCMS analysis: IR-G35-catalysed reductive amination of **4** with **E**, showing amine product **4E**.

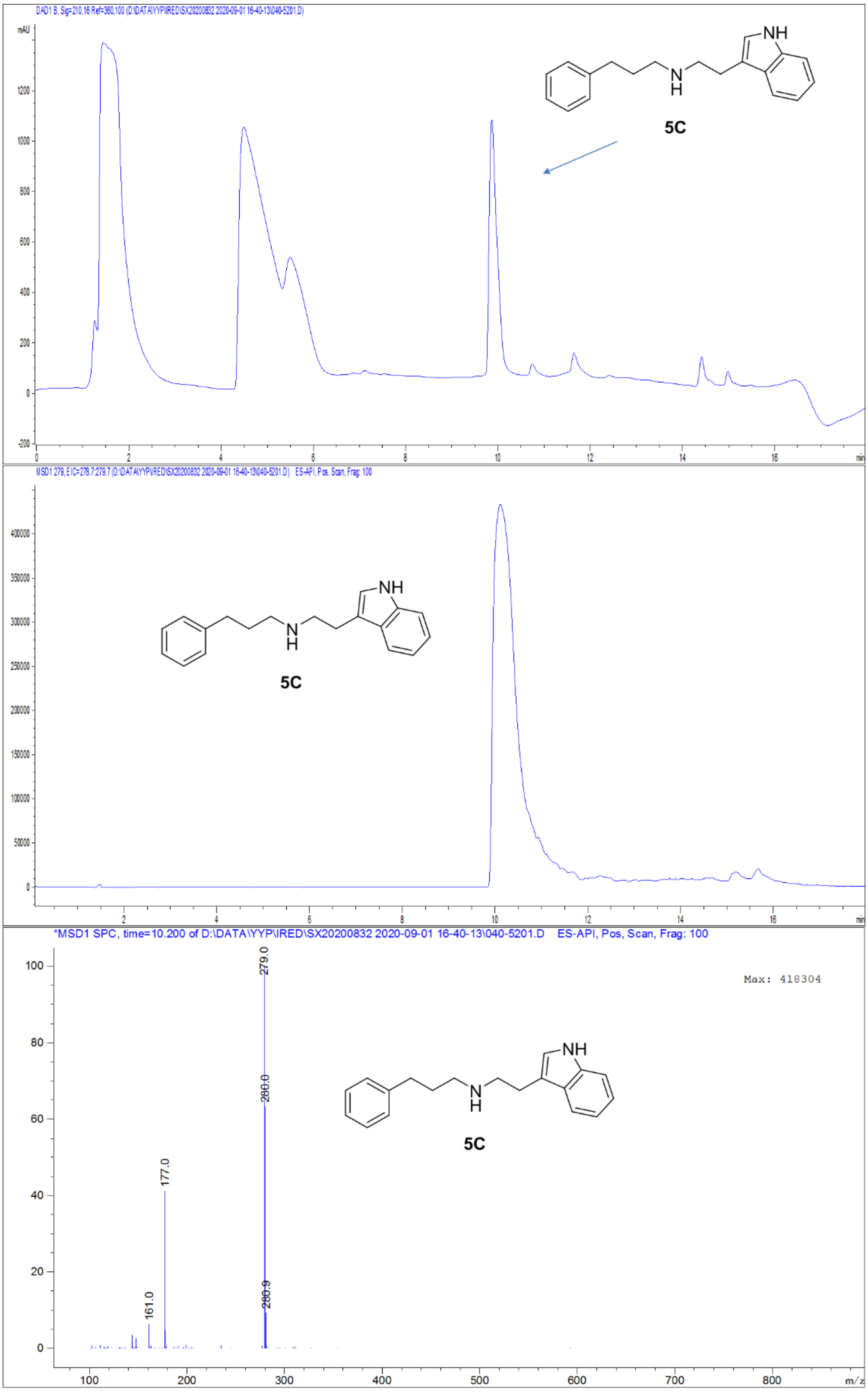

Figure 29. LCMS analysis: IR-G02-catalysed reductive amination of **5** with **C**, showing amine product **5C**.

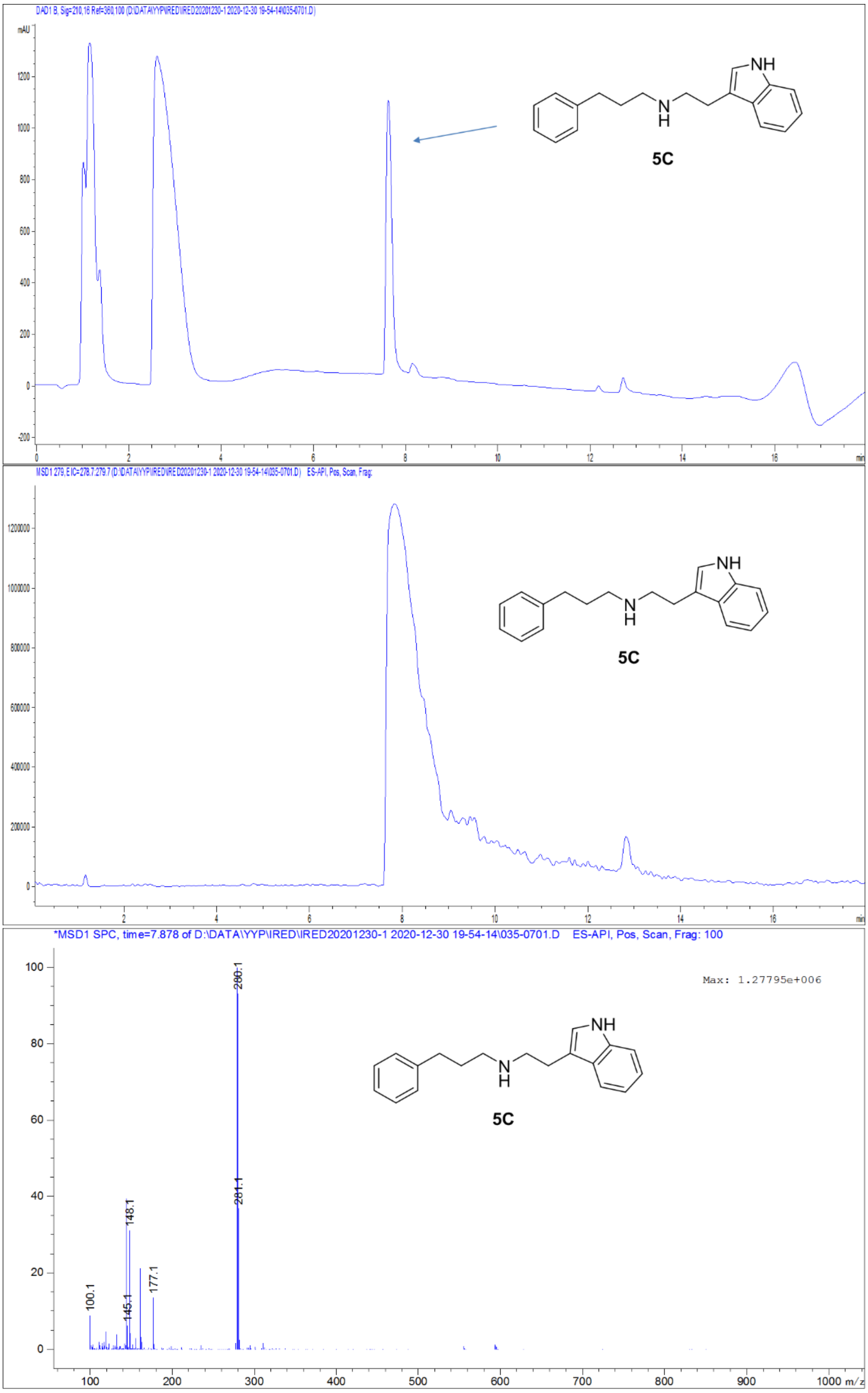

Figure 30. LCMS analysis: IR-G21-catalysed reductive amination of **5** with **C**, showing amine product **5C**.

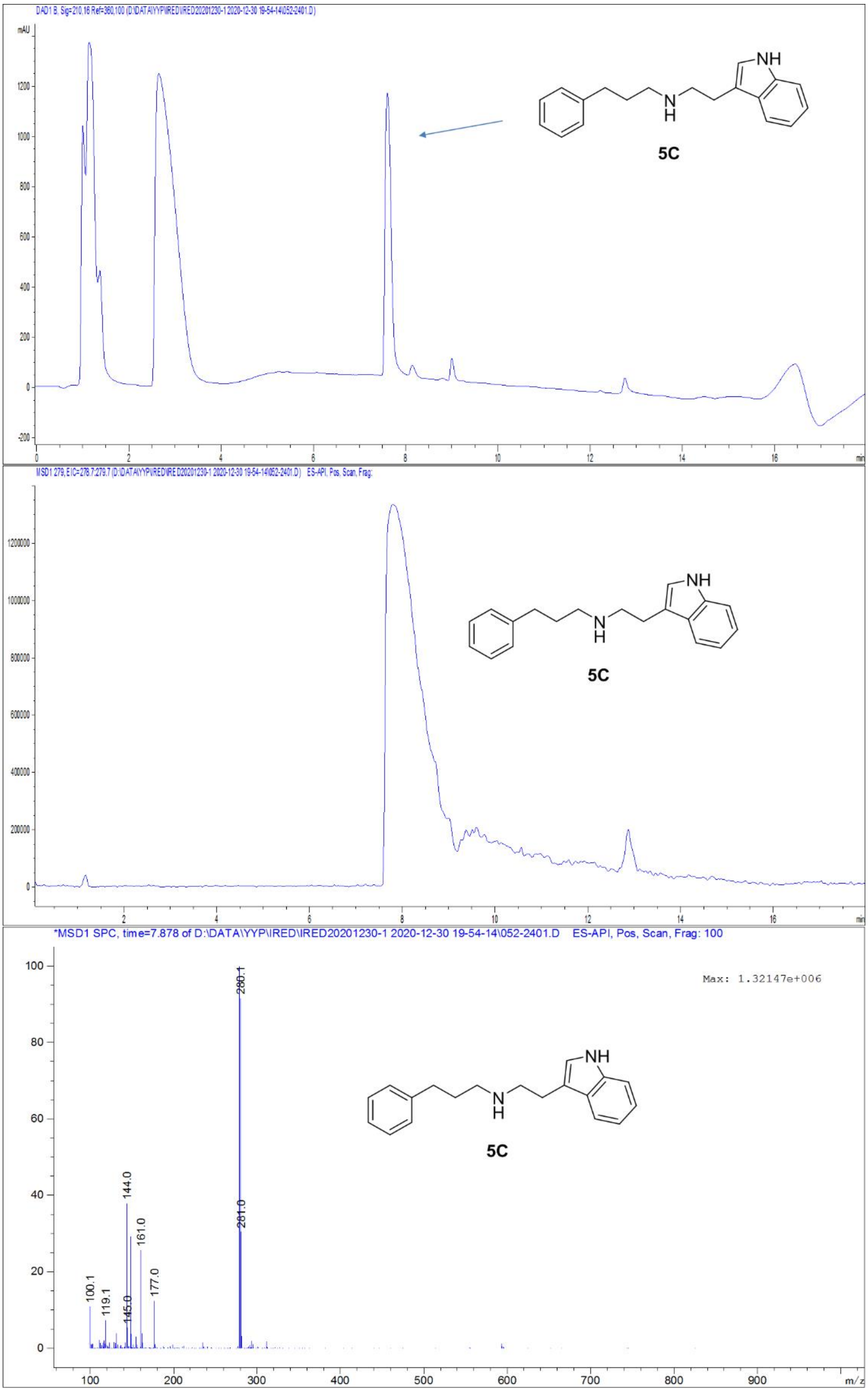

Figure 31. LCMS analysis: IR-G35-catalysed reductive amination of **5** with **C**, showing amine product **5C**.

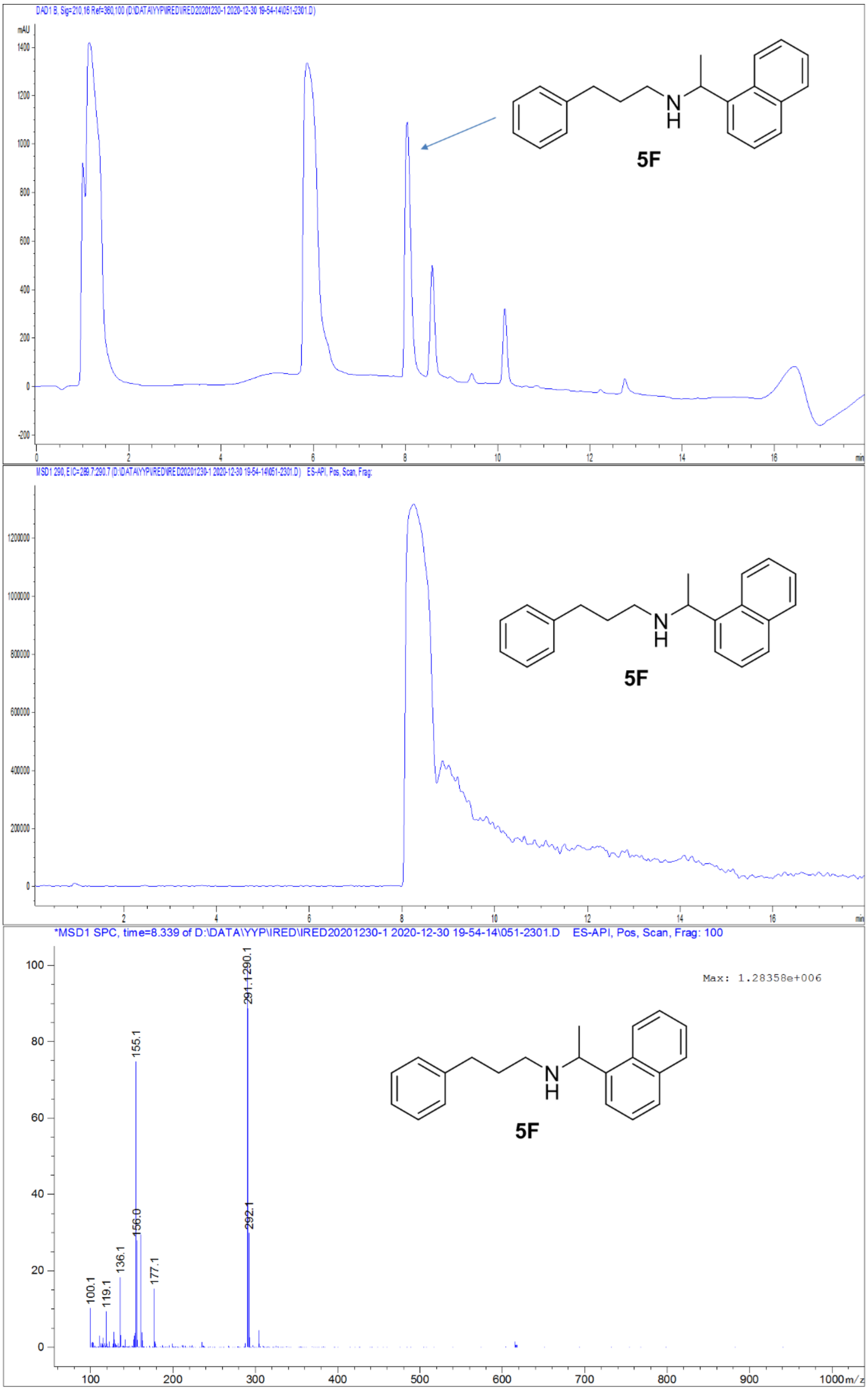

Figure 32. LCMS analysis: IR-G02-catalysed reductive amination of **5** with **F**, showing amine product **5F**.

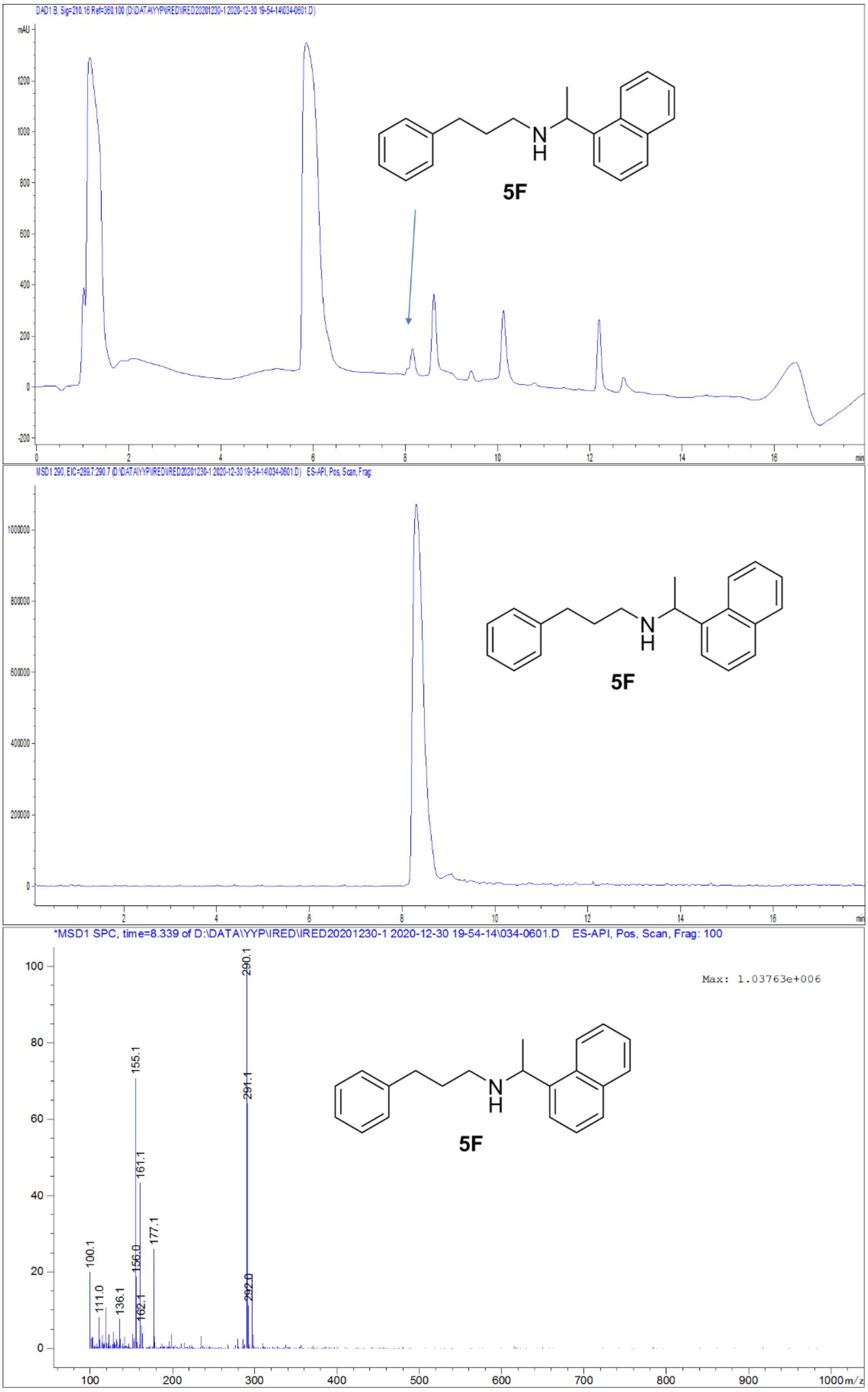

Figure 33. LCMS analysis: IR-G21-catalysed reductive amination of **5** with **F**, showing amine product **5F**.

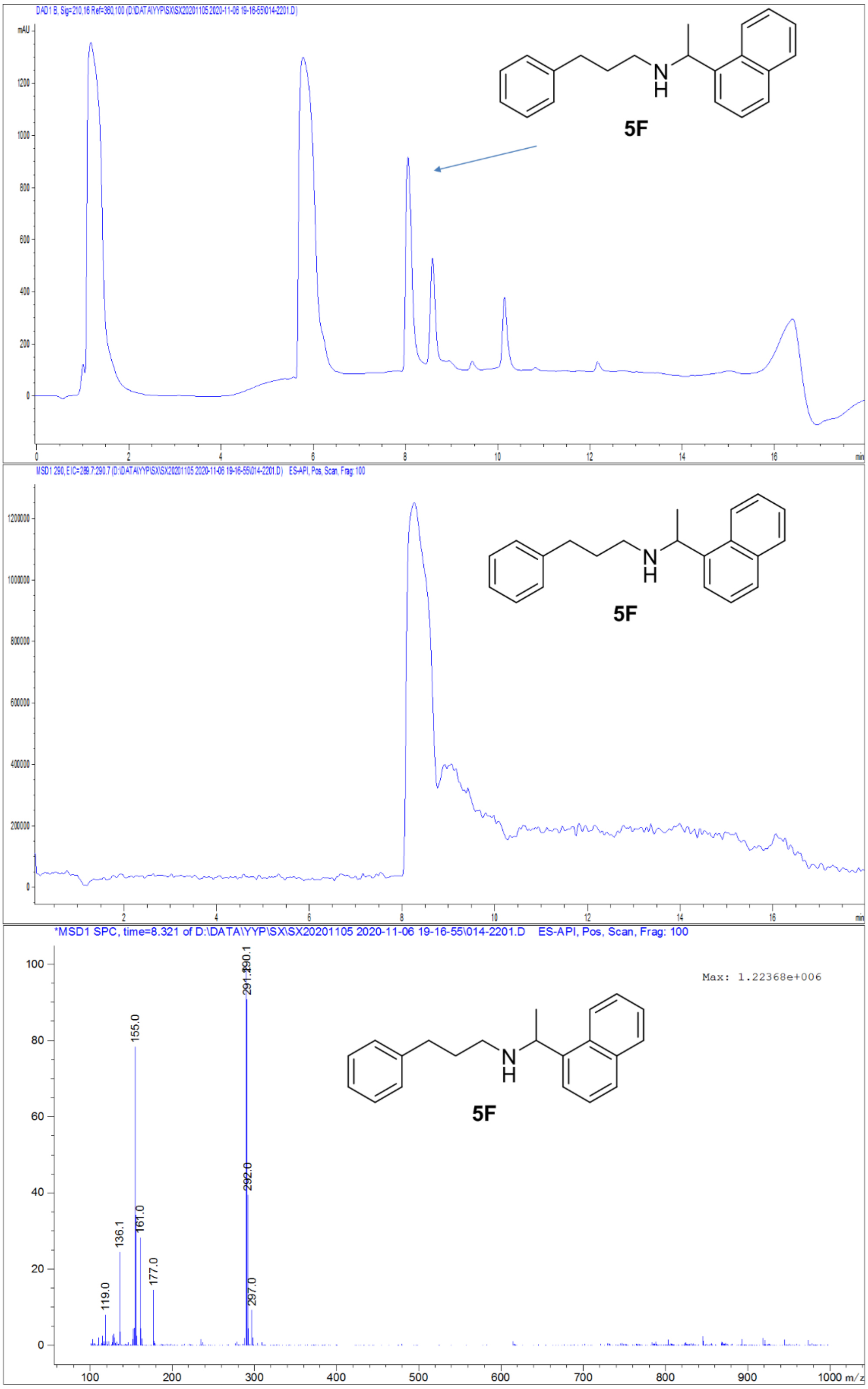

Figure 34. LCMS analysis: IR-G35-catalysed reductive amination of **5** with **F**, showing amine product **5F**.

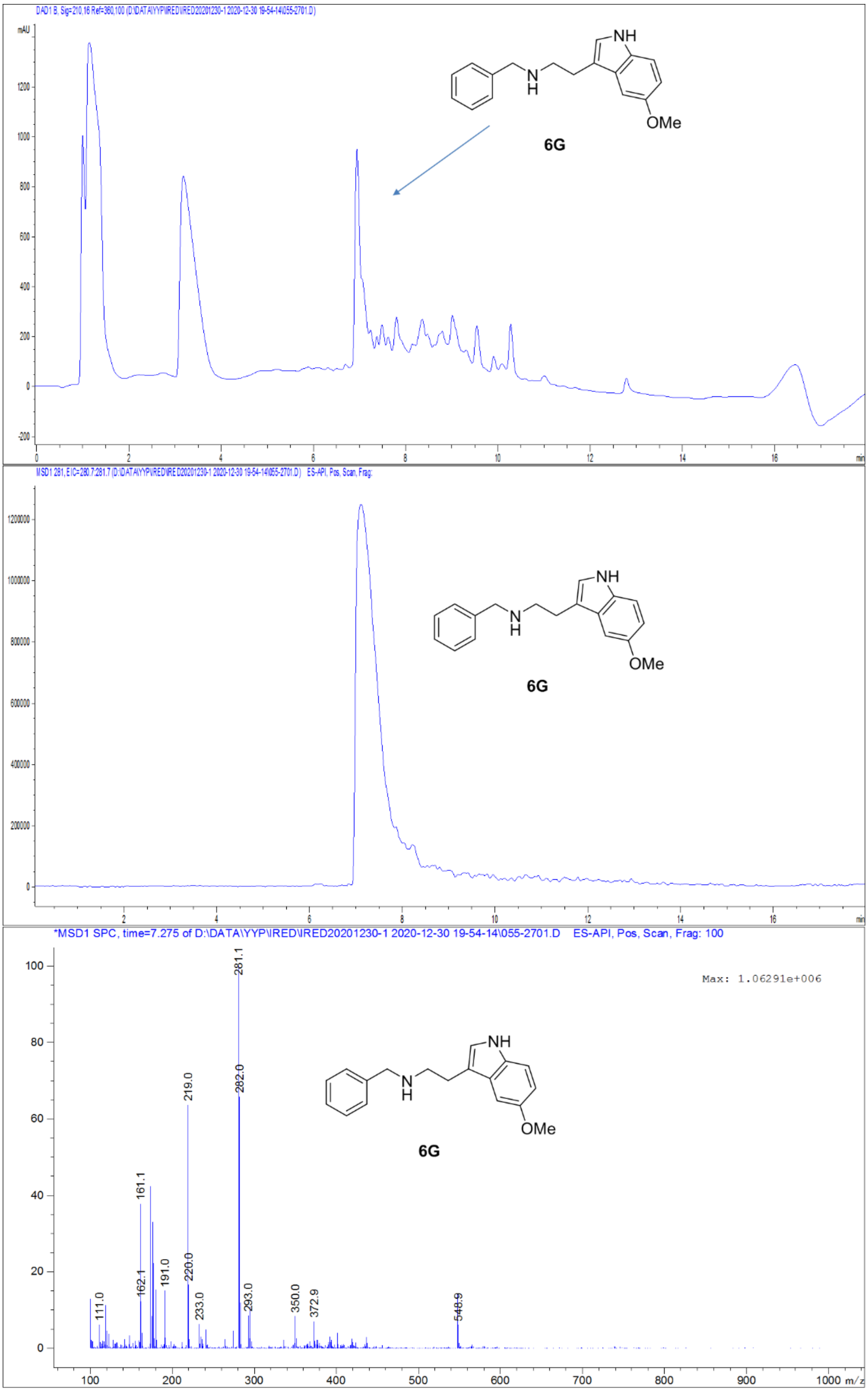

Figure 35. LCMS analysis: IR-G02-catalysed reductive amination of **6** with **G**, showing amine product **6G**.

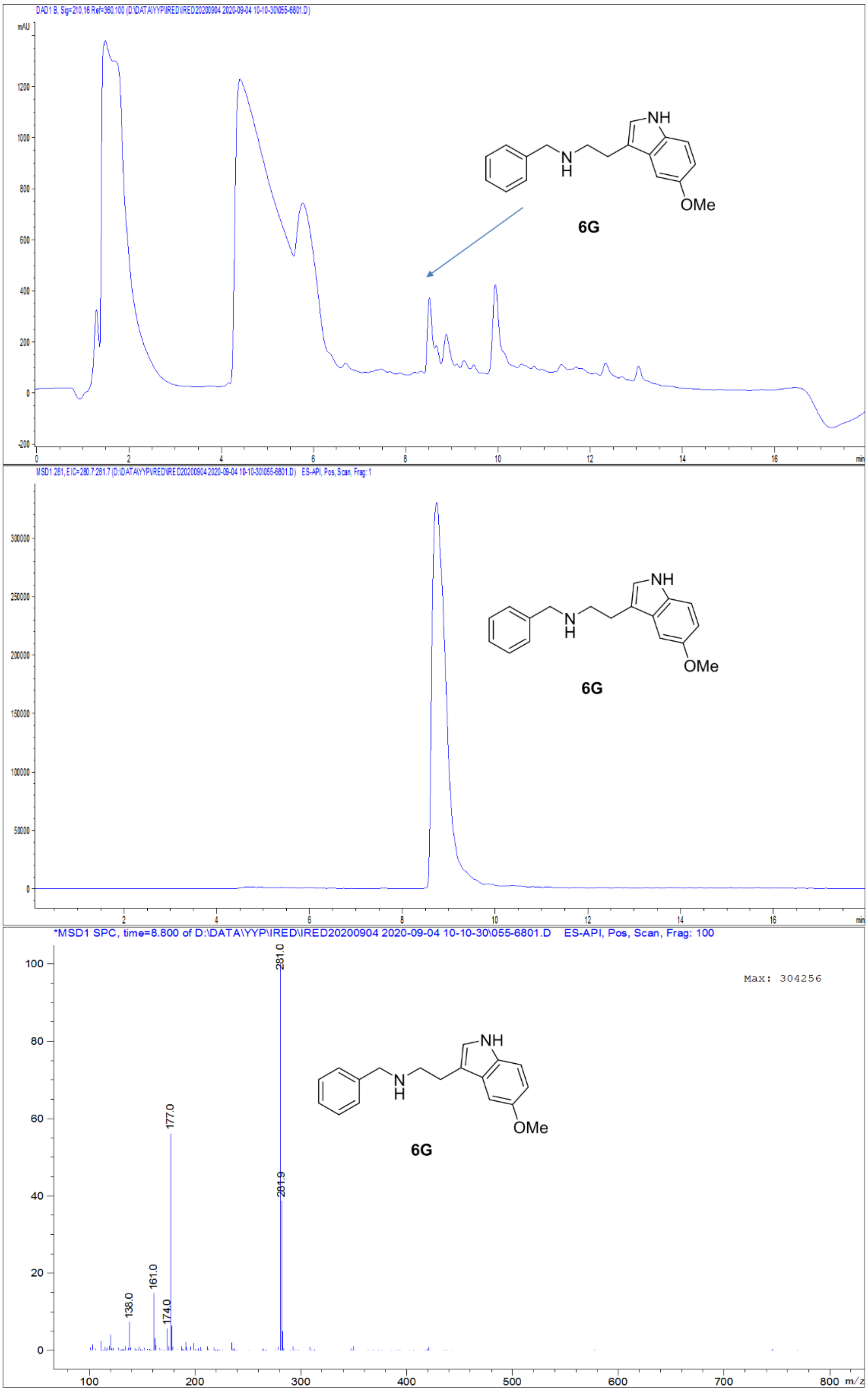

Figure 36. LCMS analysis: IR-G21-catalysed reductive amination of **6** with **G**, showing amine product **6G**.

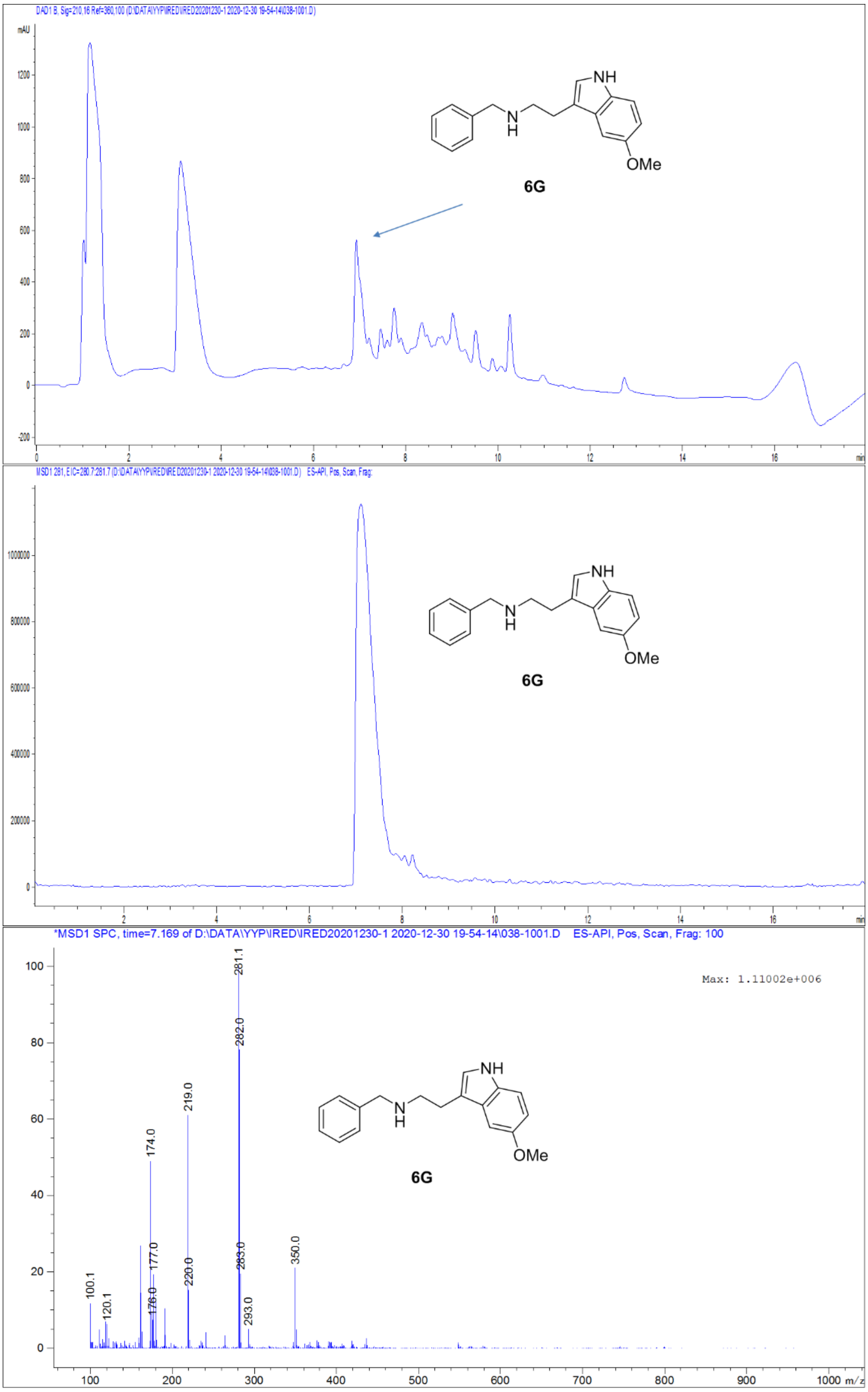

Figure 37. LCMS analysis: IR-G35-catalysed reductive amination of **6** with **G**, showing amine product **6G**.

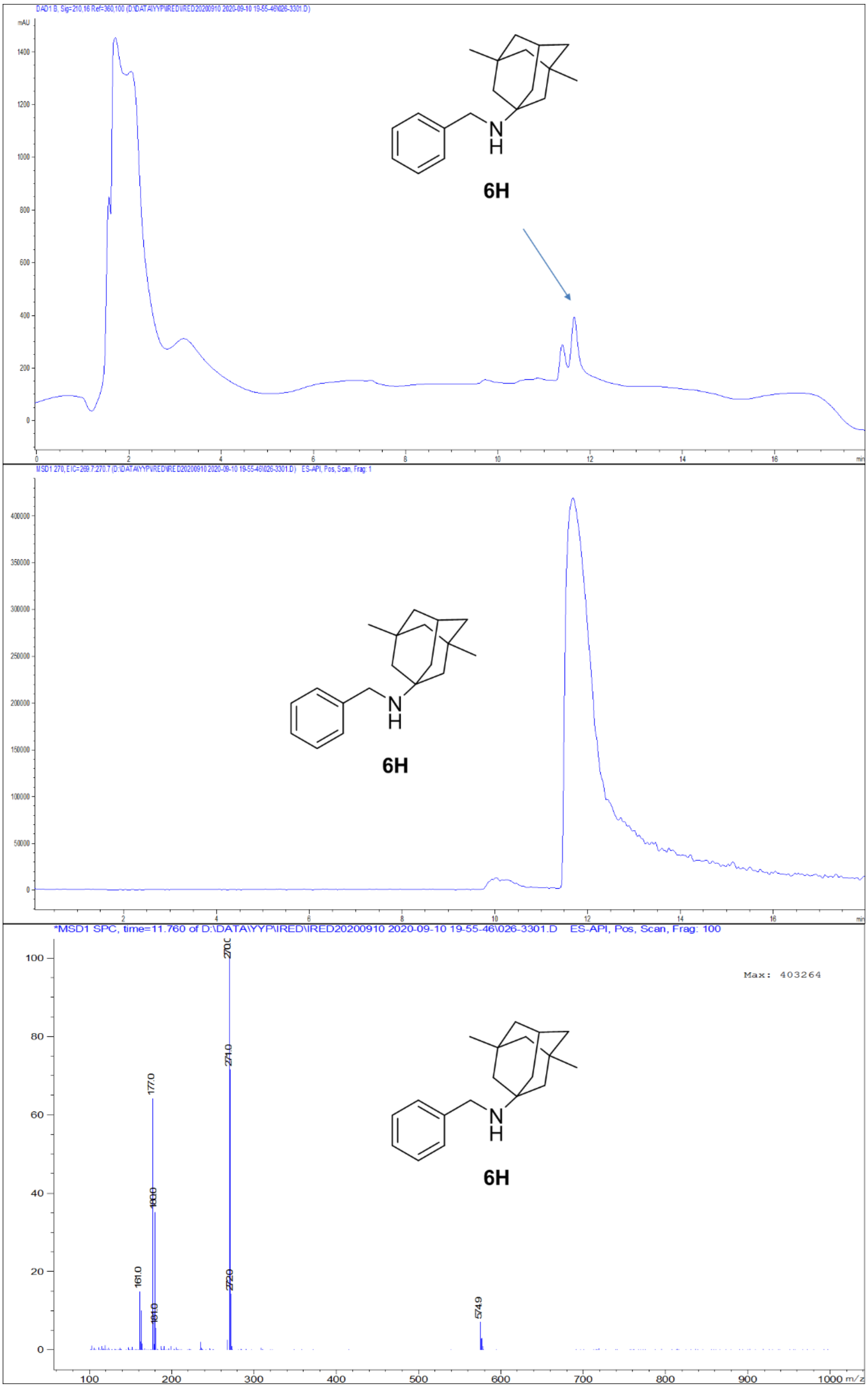

Figure 38. LCMS analysis: IR-G02-catalysed reductive amination of **6** with **H**, showing amine product **6H**.

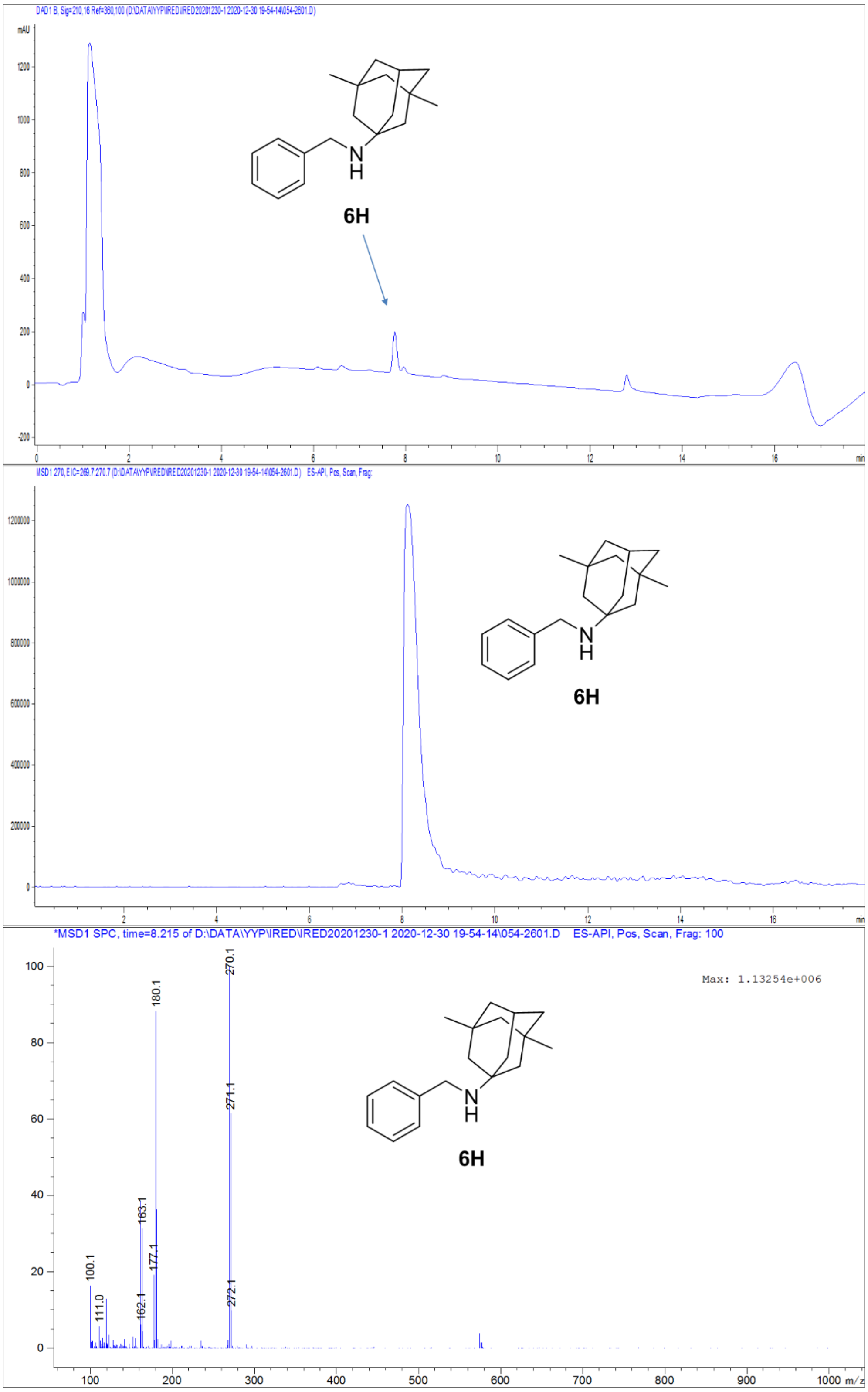

Figure 39. LCMS analysis: IR-G35-catalysed reductive amination of **6** with **H**, showing amine product **6H**.

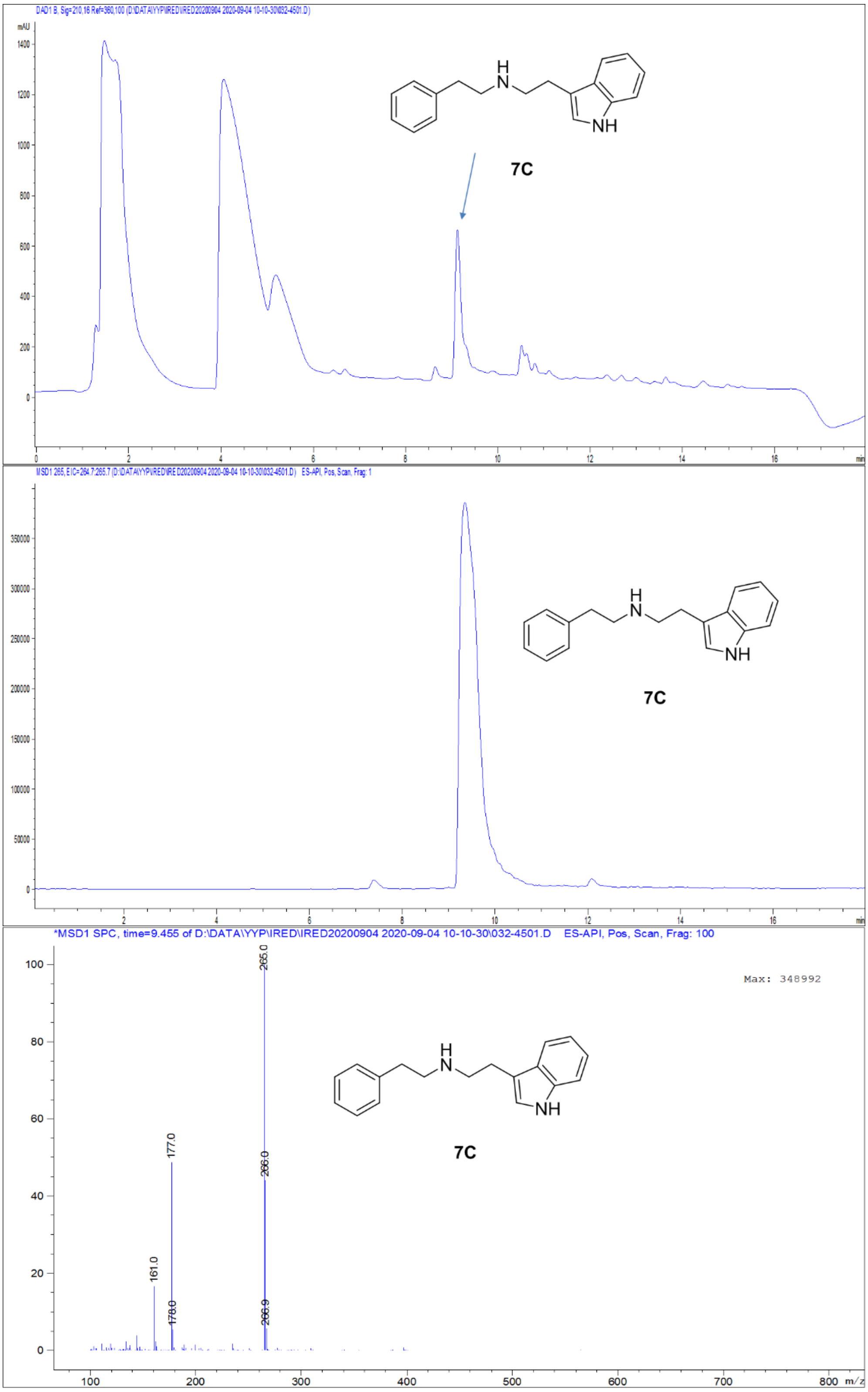

Figure 40. LCMS analysis: IR-G02-catalysed reductive amination of **7** with **C**, showing amine product **7C**.

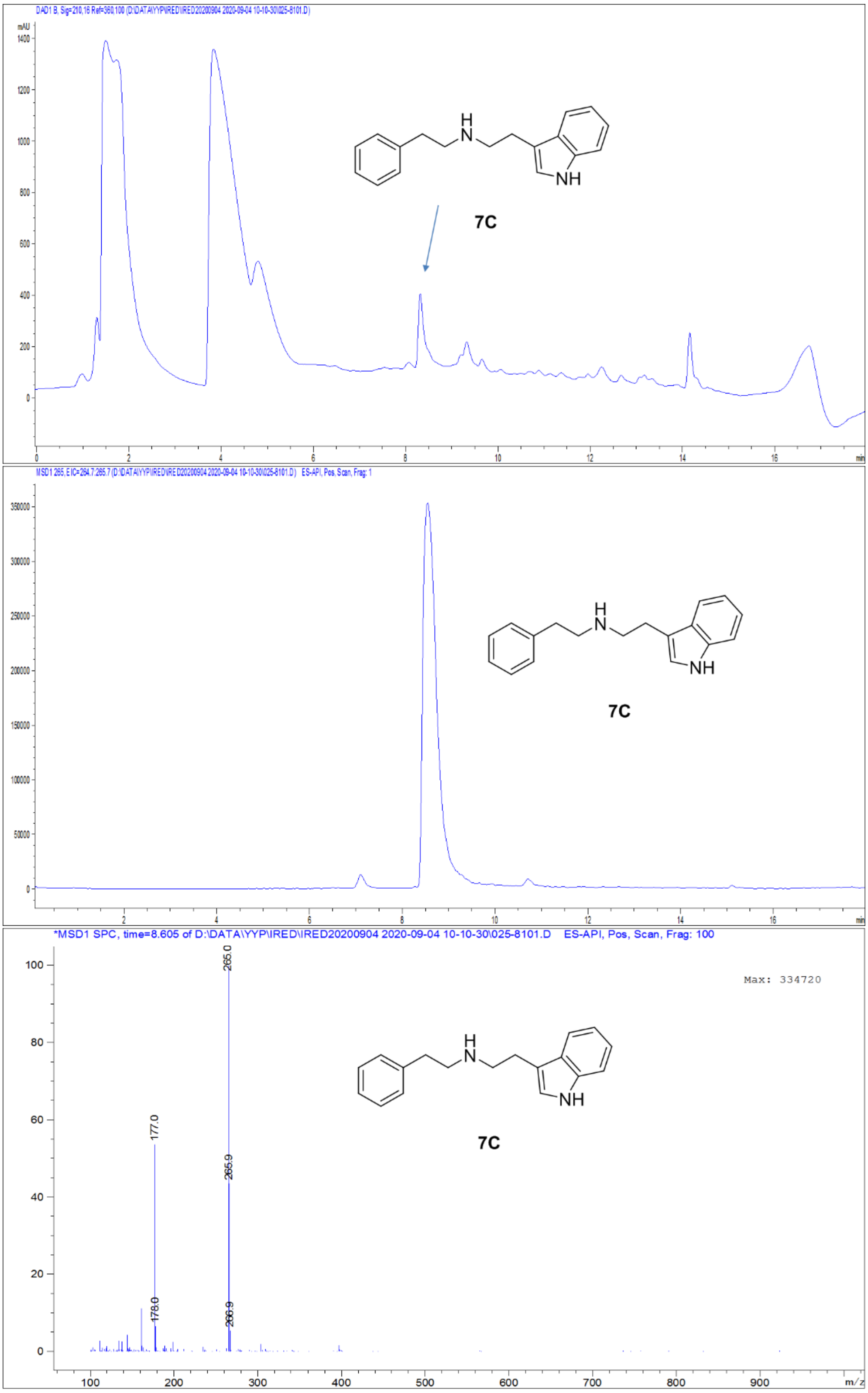

Figure 41. LCMS analysis: IR-G21-catalysed reductive amination of **7** with **C**, showing amine product **7C**.

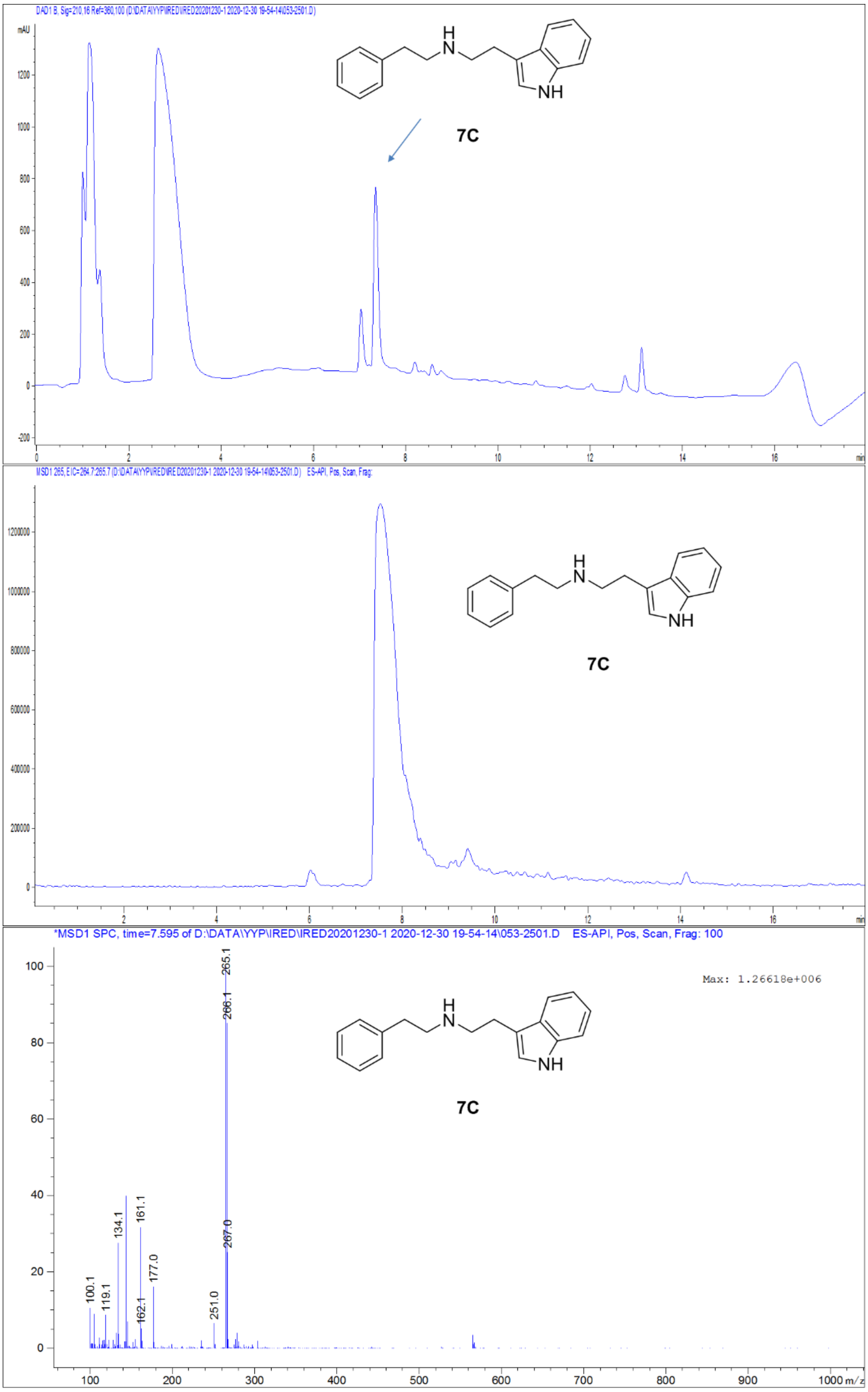

Figure 42. LCMS analysis: IR-G35-catalysed reductive amination of **7** with **C**, showing amine product **7C**.

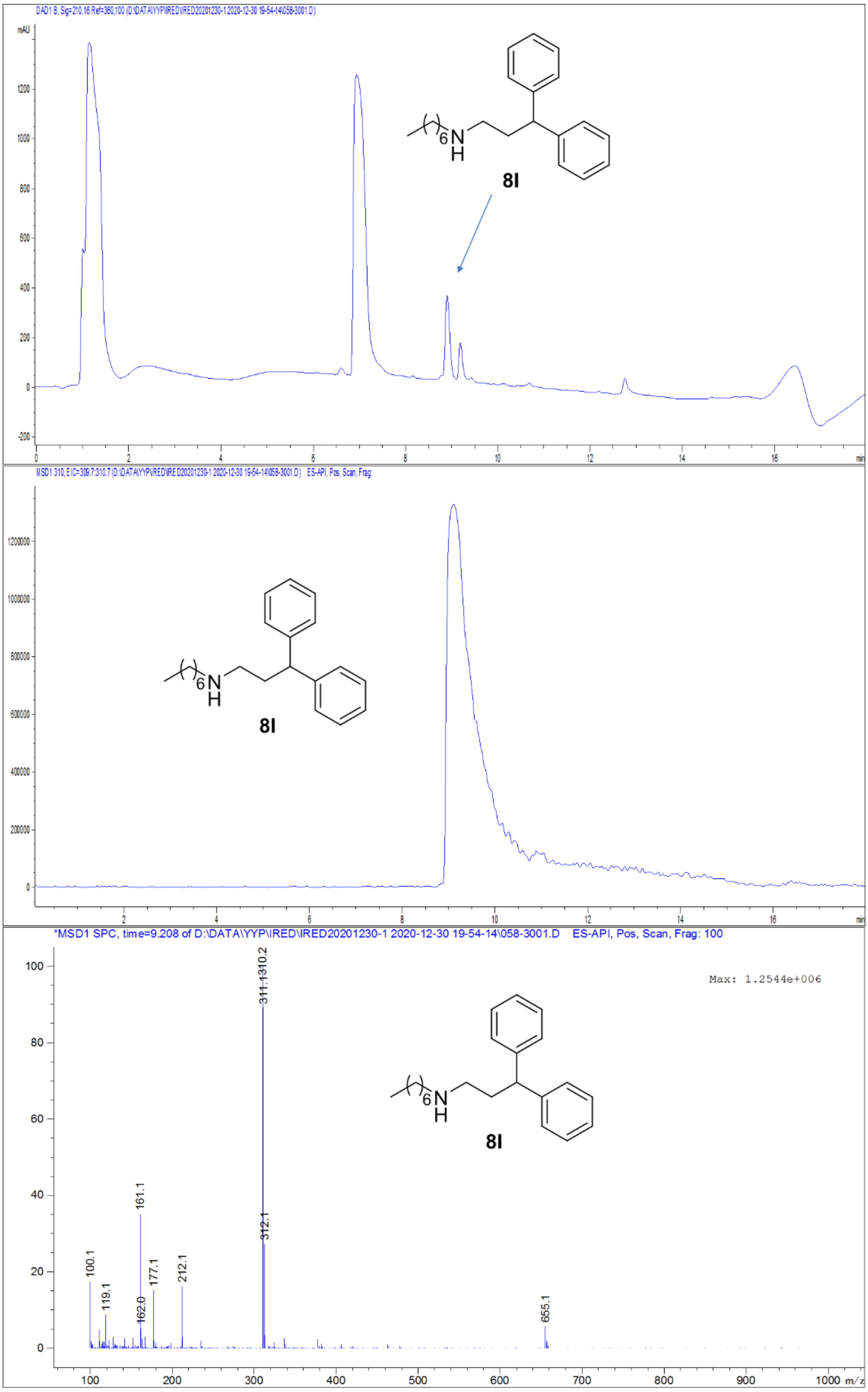

Figure 43. LCMS analysis: IR-G02-catalysed reductive amination of **8** with **I**, showing amine product **8I**.

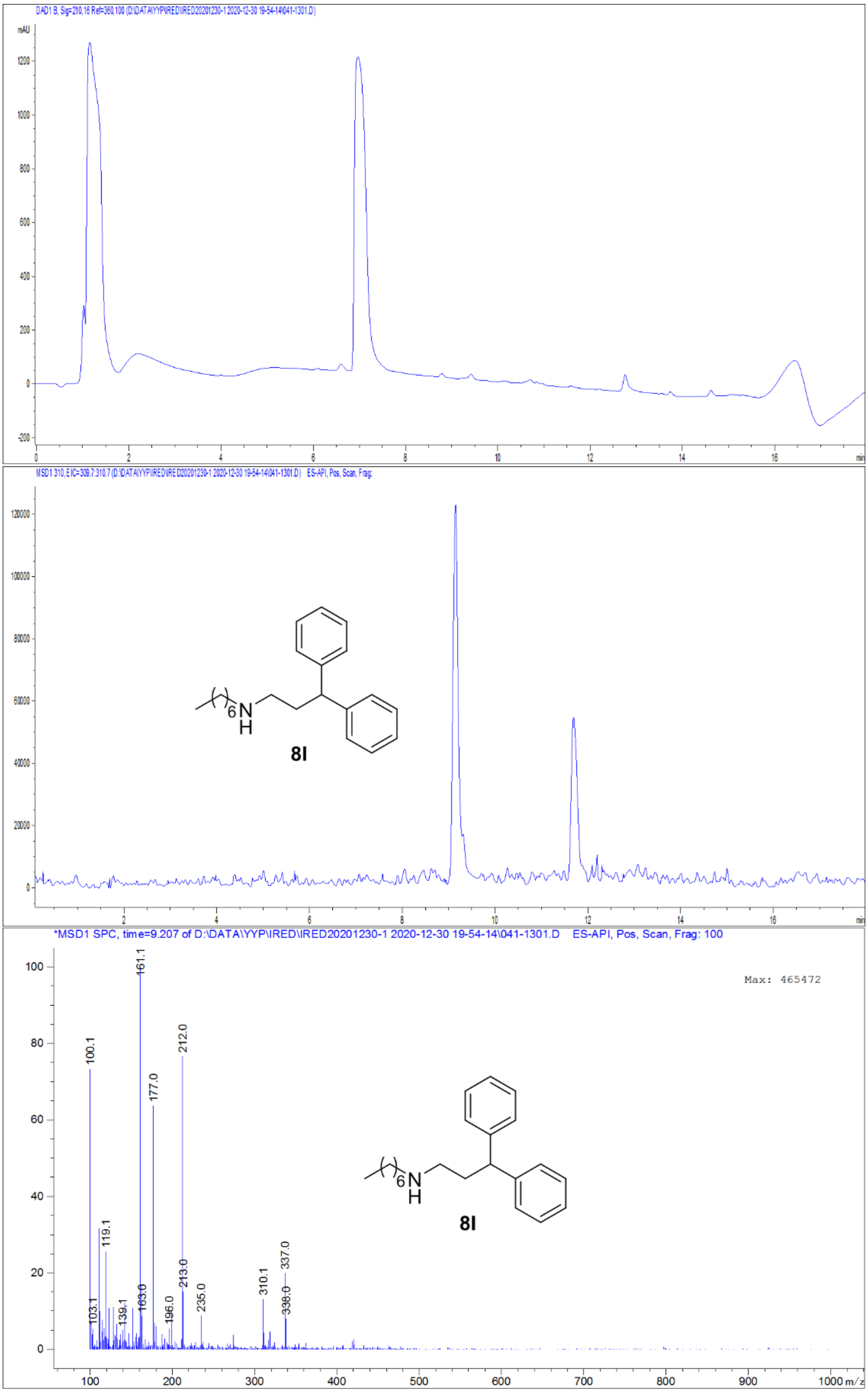

Figure 44. LCMS analysis: IR-G21-catalysed reductive amination of **8** with **I**, showing amine product **8I**.

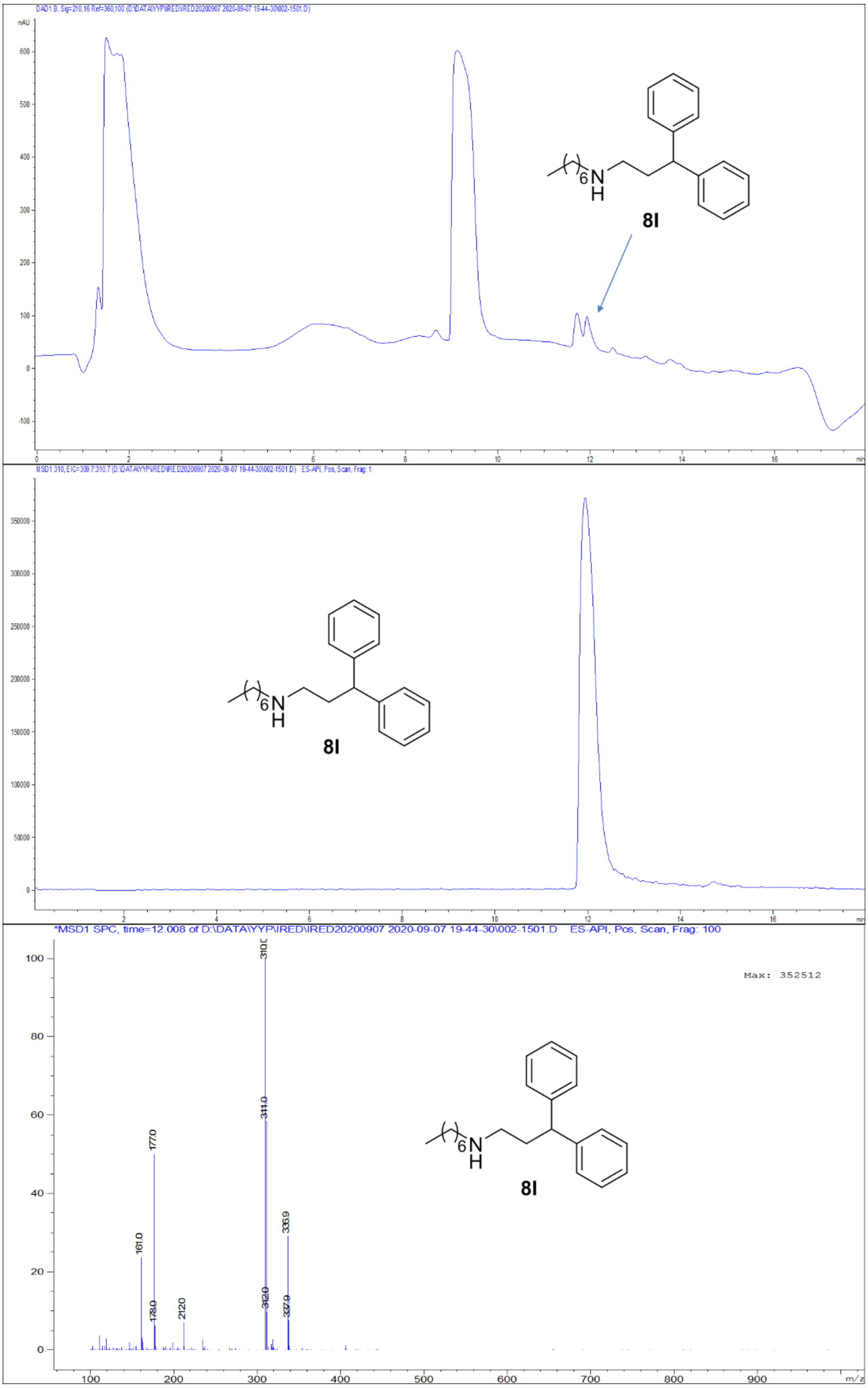

Figure 45. LCMS analysis: IR-G35-catalysed reductive amination of **8** with **I**, showing amine product **8I**.

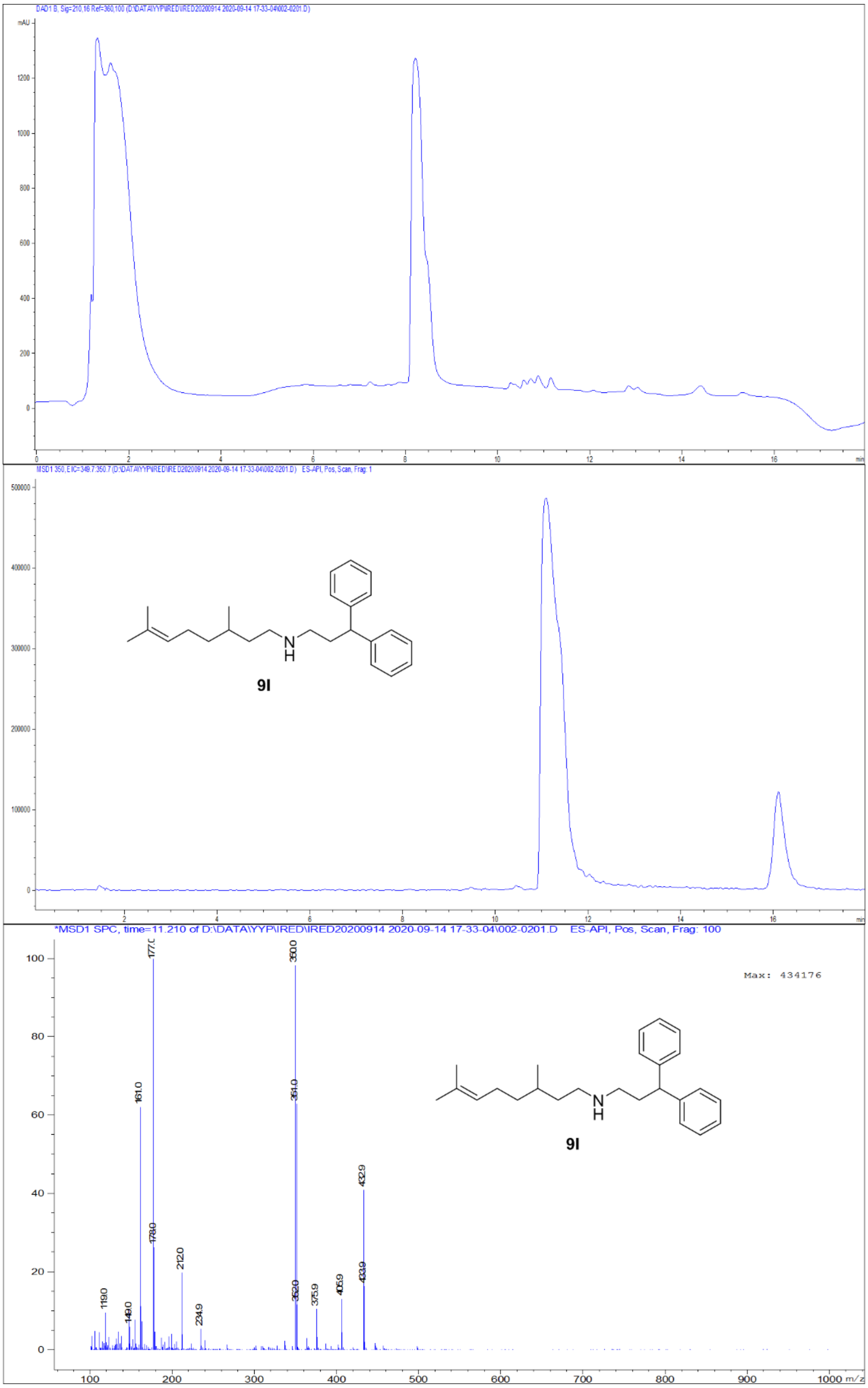

Figure 46. LCMS analysis: IR-G02-catalysed reductive amination of **9** with **I**, showing amine product **9I**.

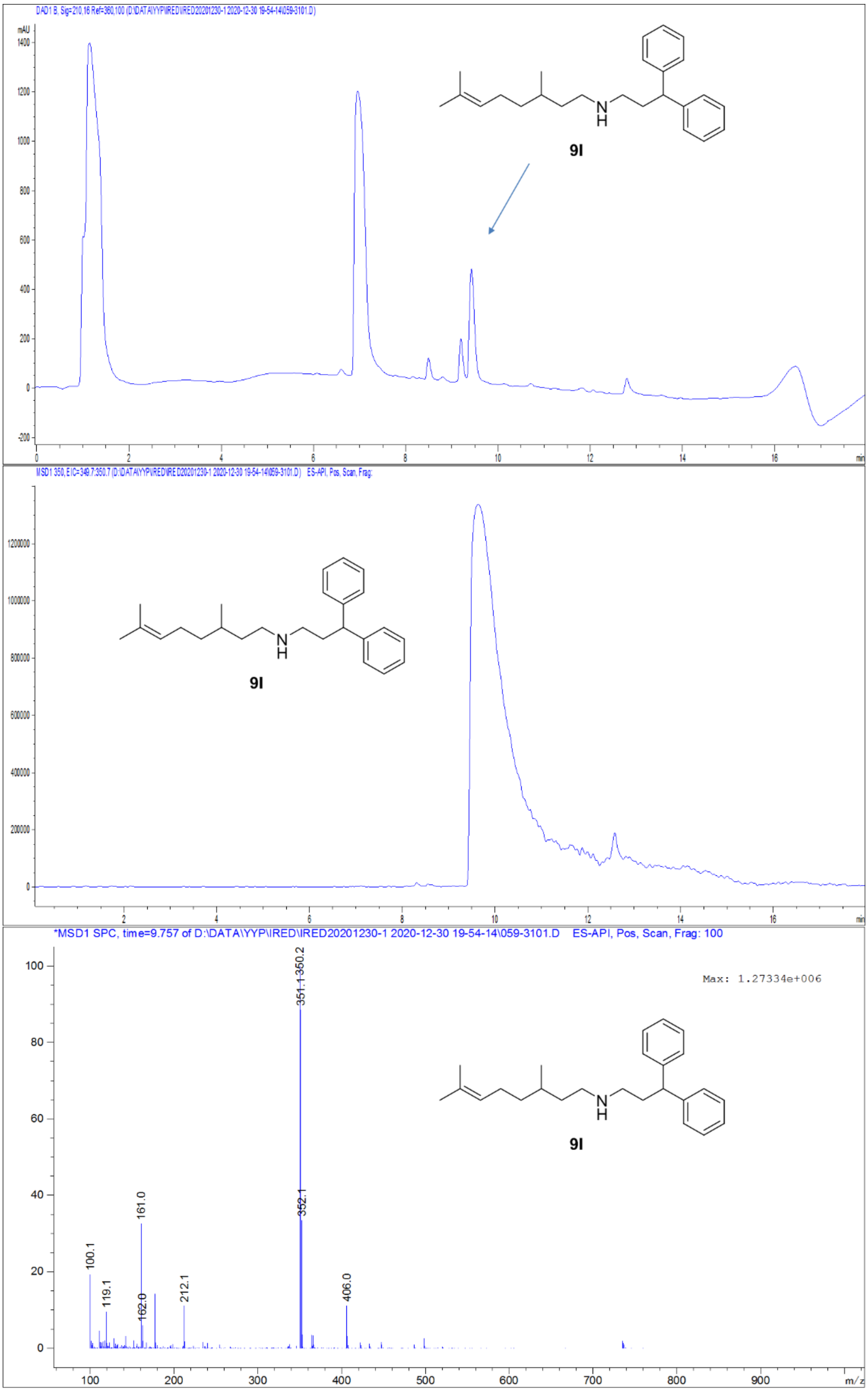

Figure 47. LCMS analysis: IR-G35-catalysed reductive amination of **9** with **I**, showing amine product **9I**.

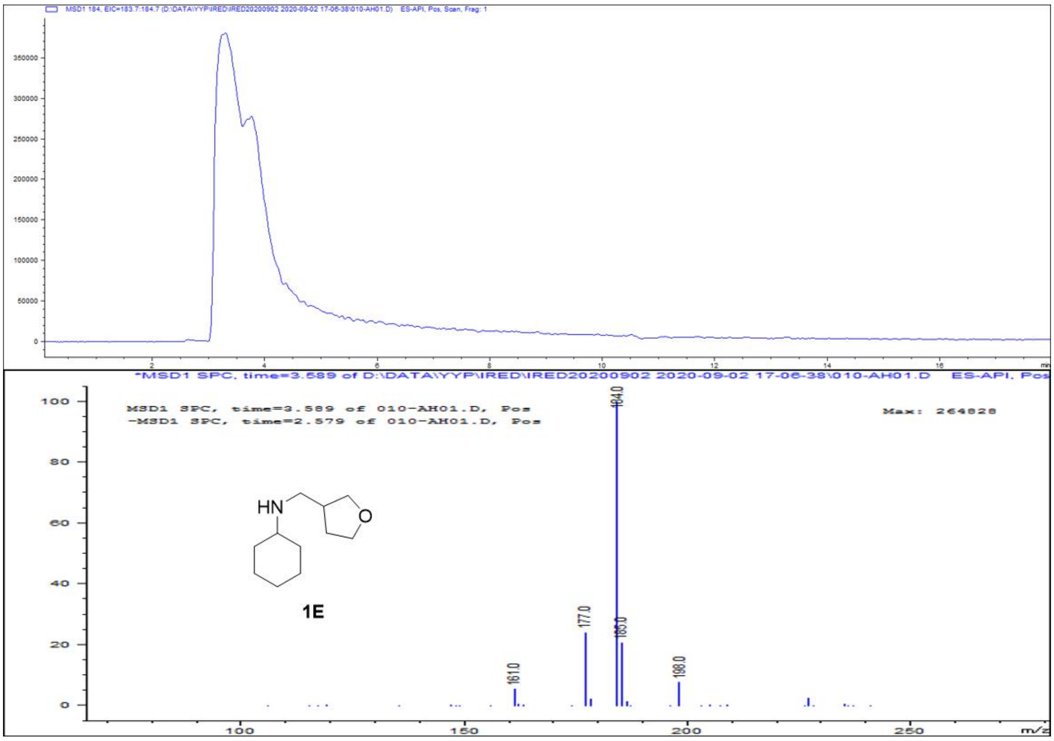

Figure 48. LCMS analysis: IR-G02-catalysed reductive amination of **1** with **E**, showing amine product **1E**.

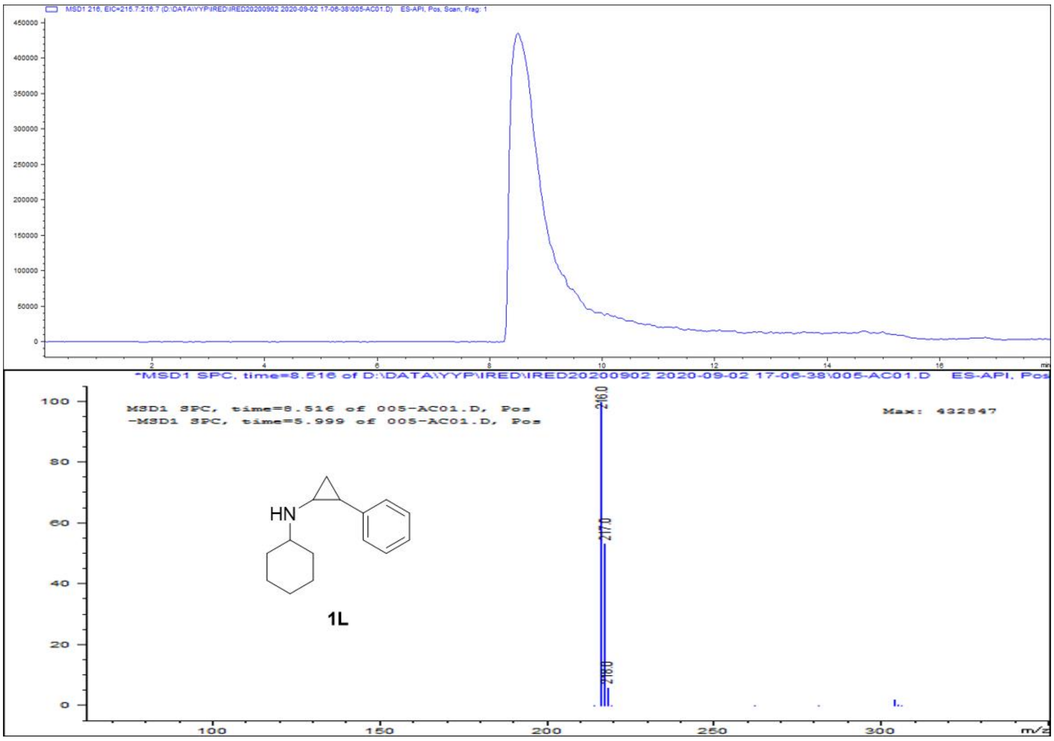

Figure 49. LCMS analysis: IR-G02-catalysed reductive amination of **1** with **L**, showing amine product **1L**.

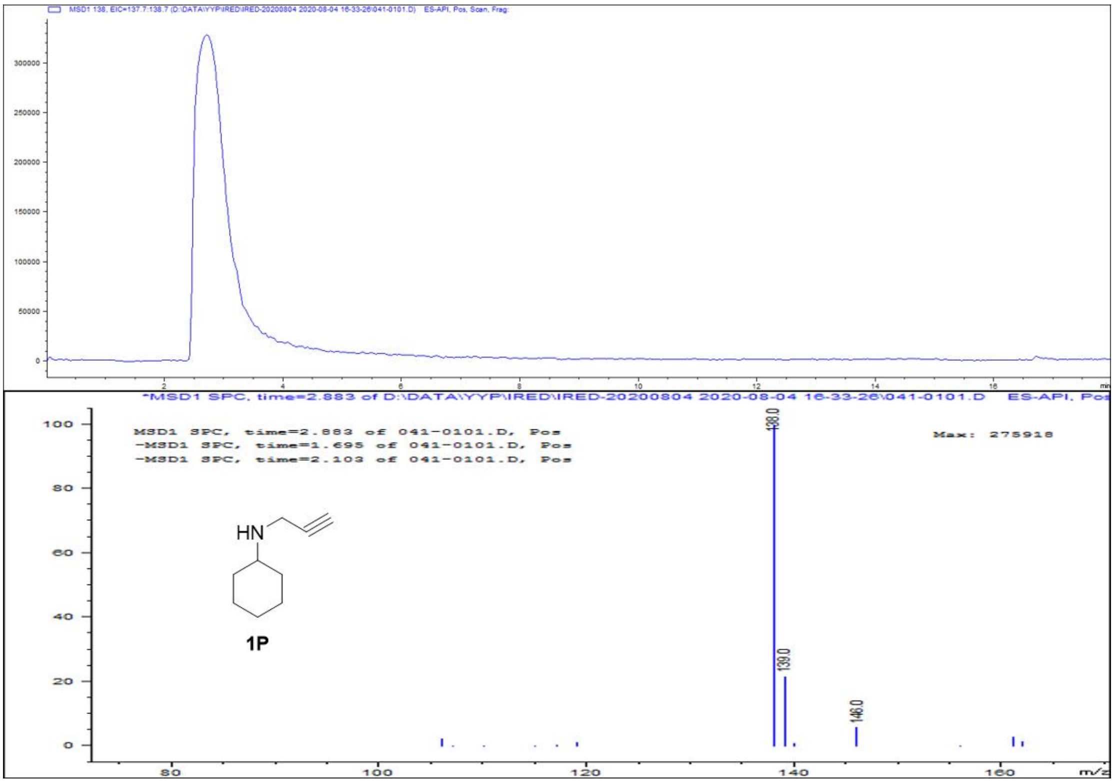

Figure 50. LCMS analysis: IR-G02-catalysed reductive amination of **1** with **P**, showing amine product **1P**.

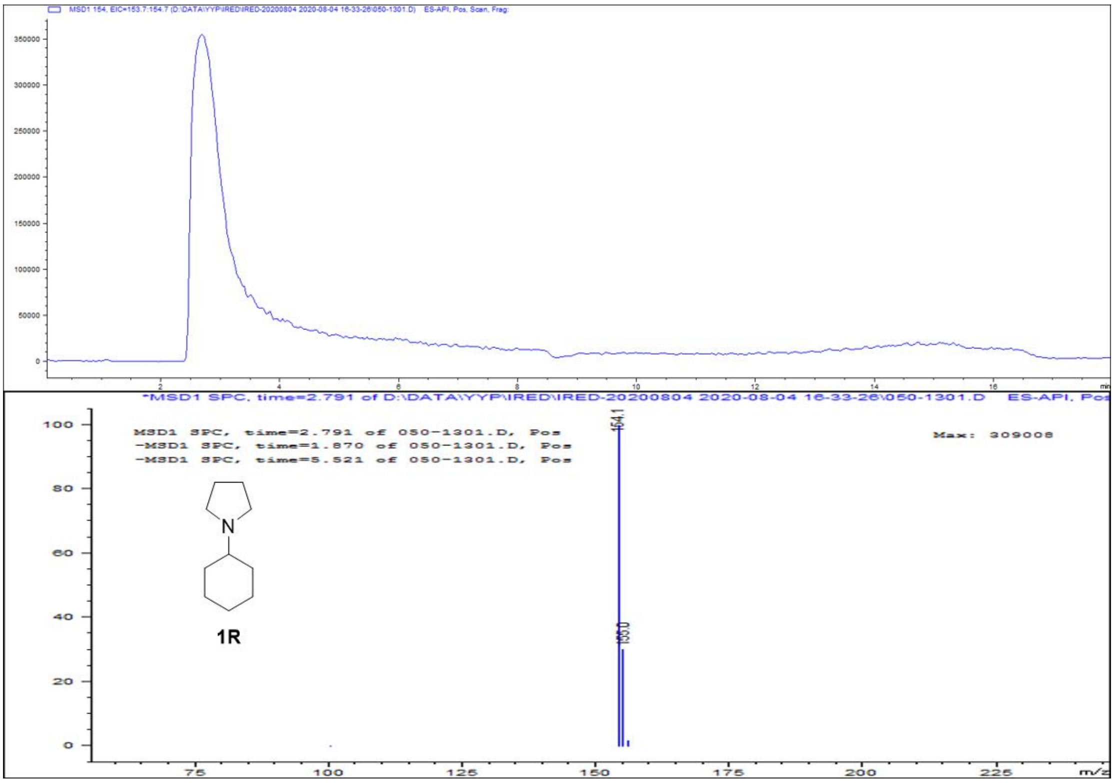

Figure 51. LCMS analysis: IR-G02-catalysed reductive amination of **1** with **R**, showing amine product **1R**.

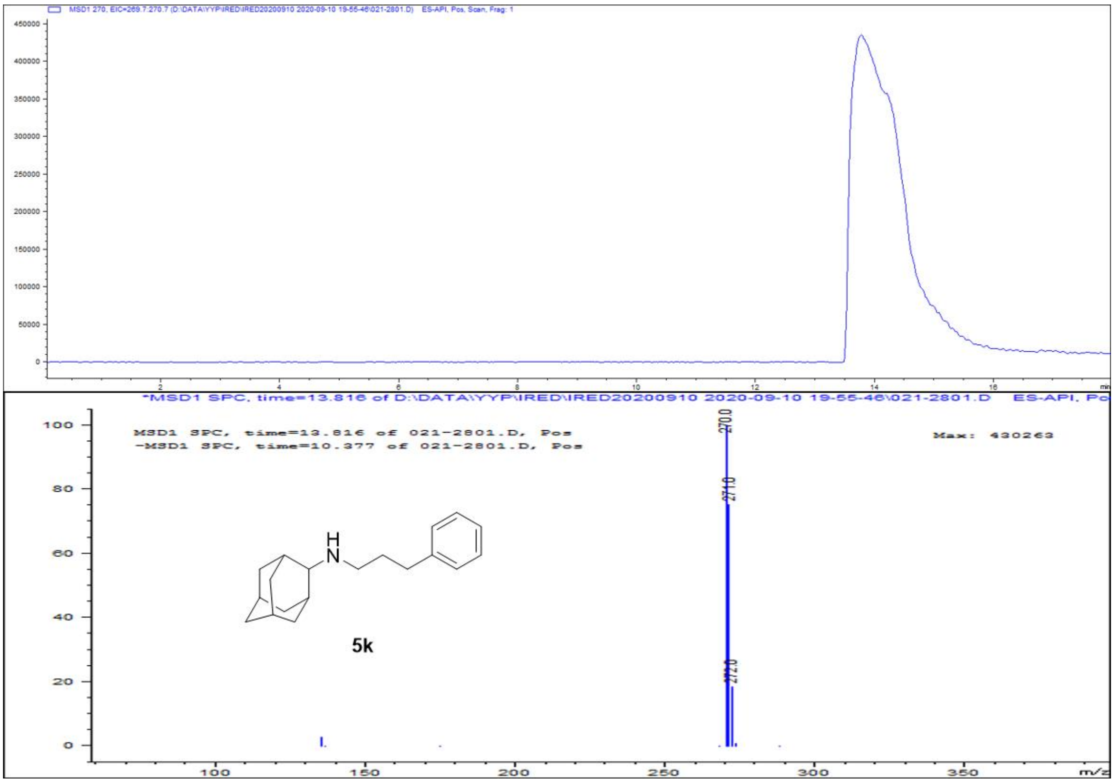

Figure 52. LCMS analysis: IR-G02-catalysed reductive amination of **5** with **K**, showing amine product **5K**.

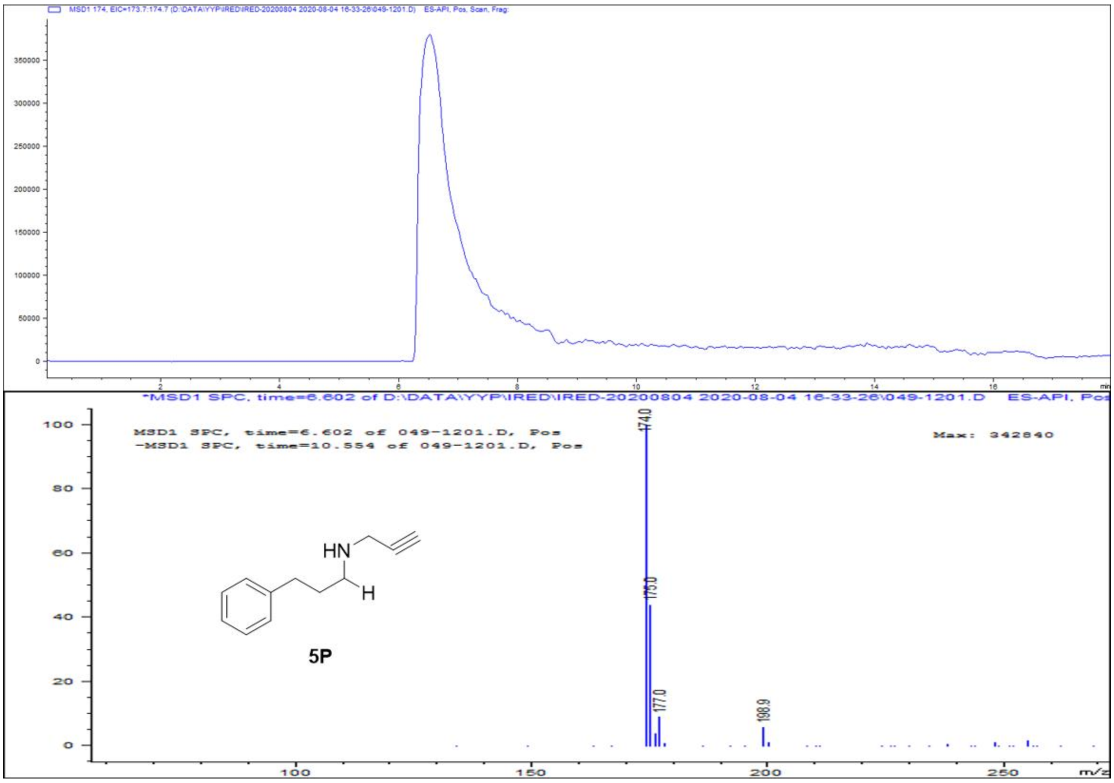

Figure 53. LCMS analysis: IR-G02-catalysed reductive amination of **5** with **P**, showing amine product **5P**.

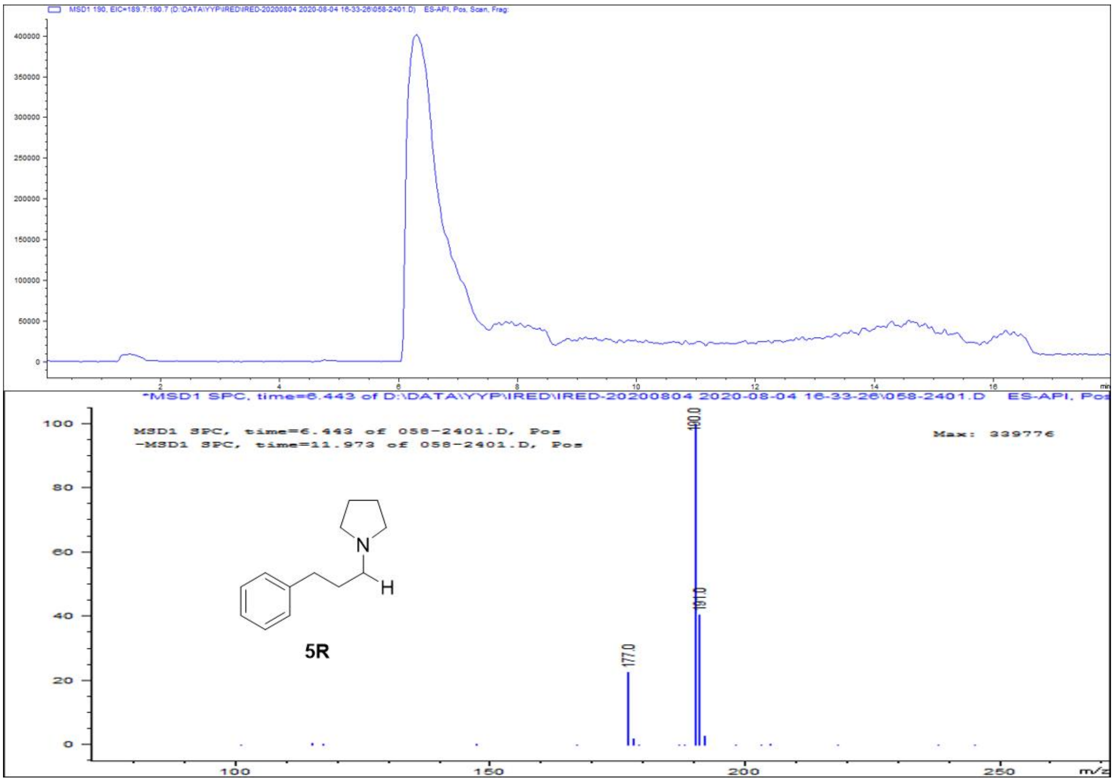

Figure 54. LCMS analysis: IR-G02-catalysed reductive amination of **5** with **R**, showing amine product **5R**.

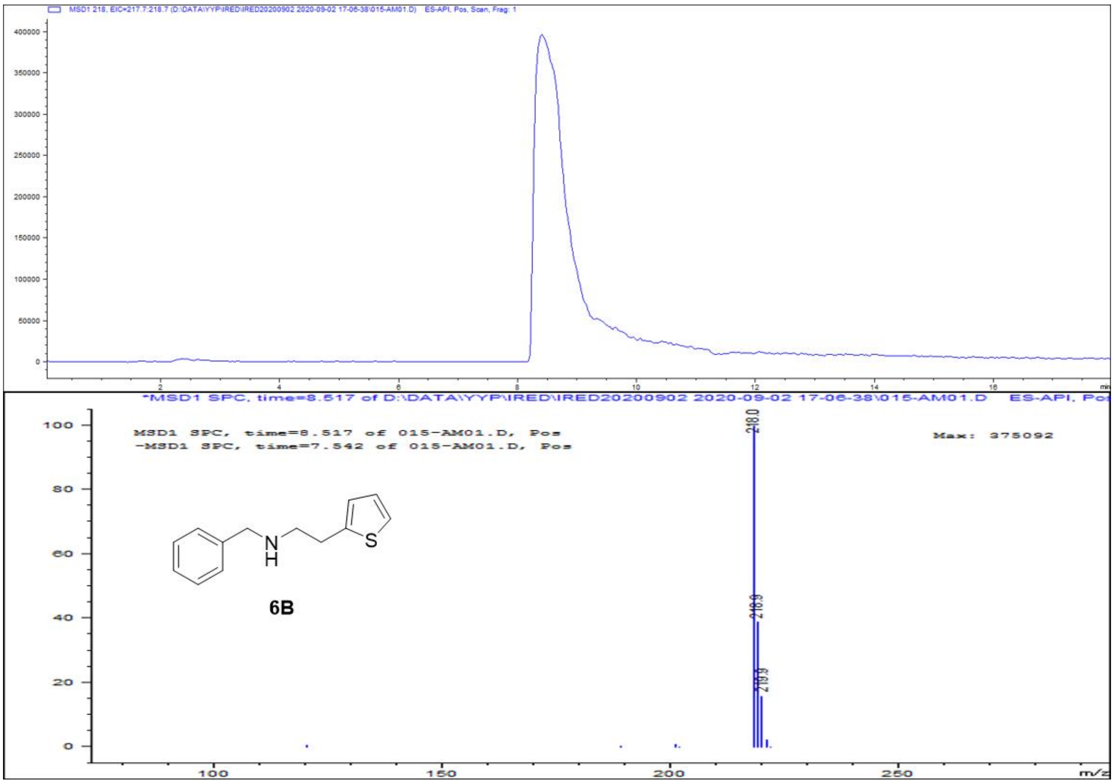

Figure 55. LCMS analysis: IR-G02-catalysed reductive amination of **6** with **B**, showing amine product **6B**.

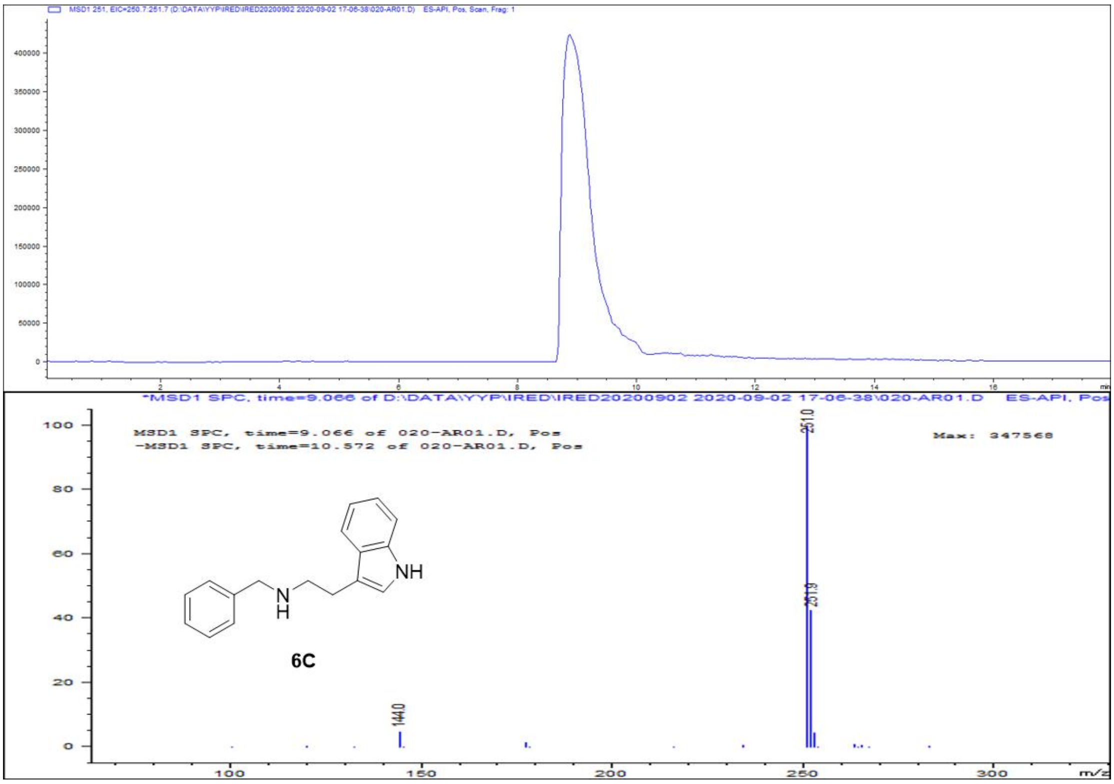

Figure 56. LCMS analysis: IR-G02-catalysed reductive amination of **6** with **C**, showing amine product **6C**.

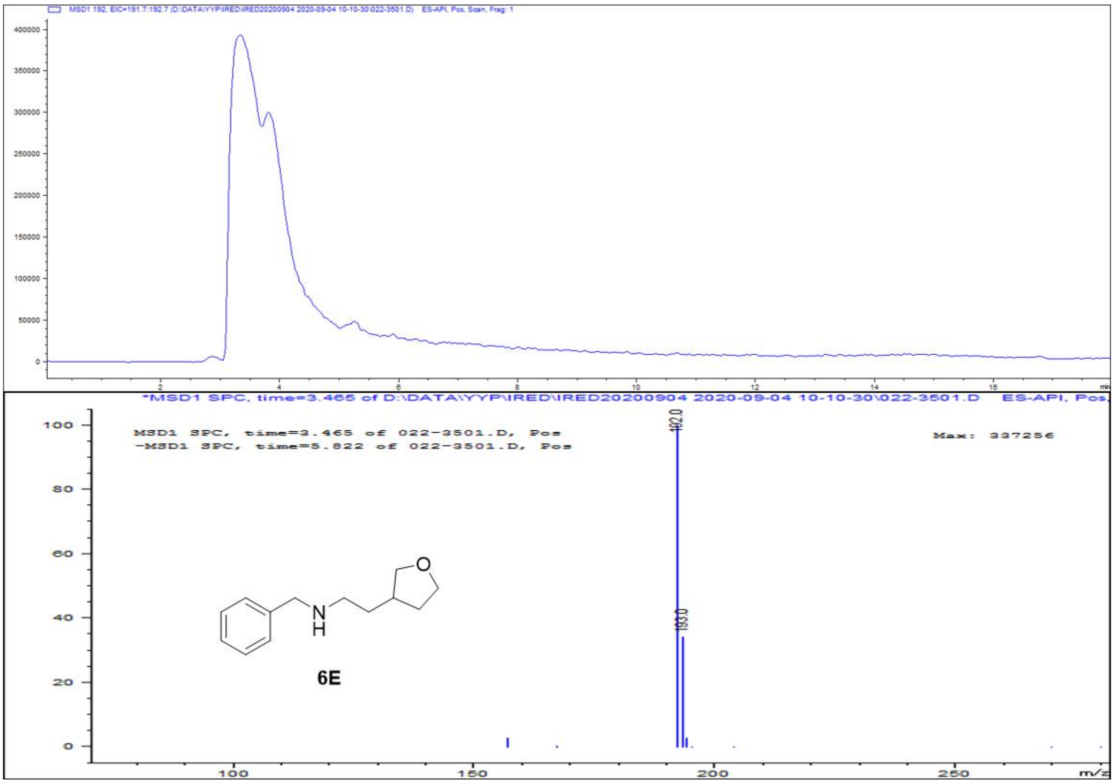

Figure 57. LCMS analysis: IR-G02-catalysed reductive amination of **6** with **E**, showing amine product **6E**.

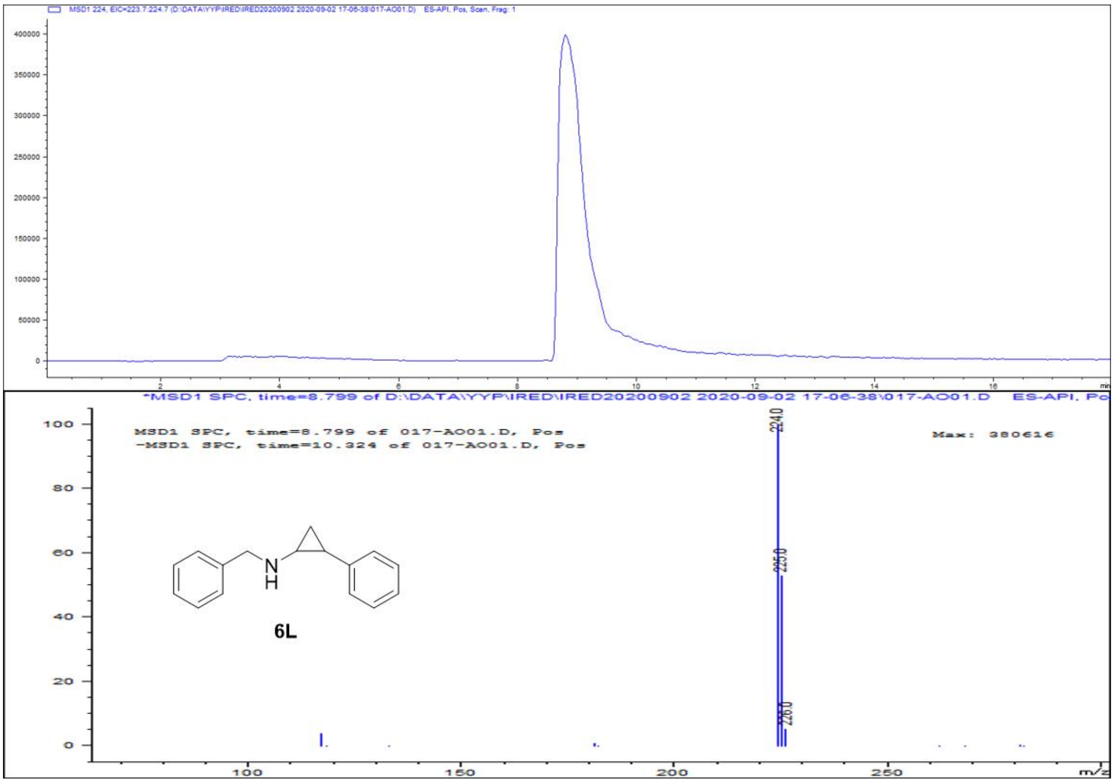

Figure 58. LCMS analysis: IR-G02-catalysed reductive amination of **6** with **L**, showing amine product **6L**.

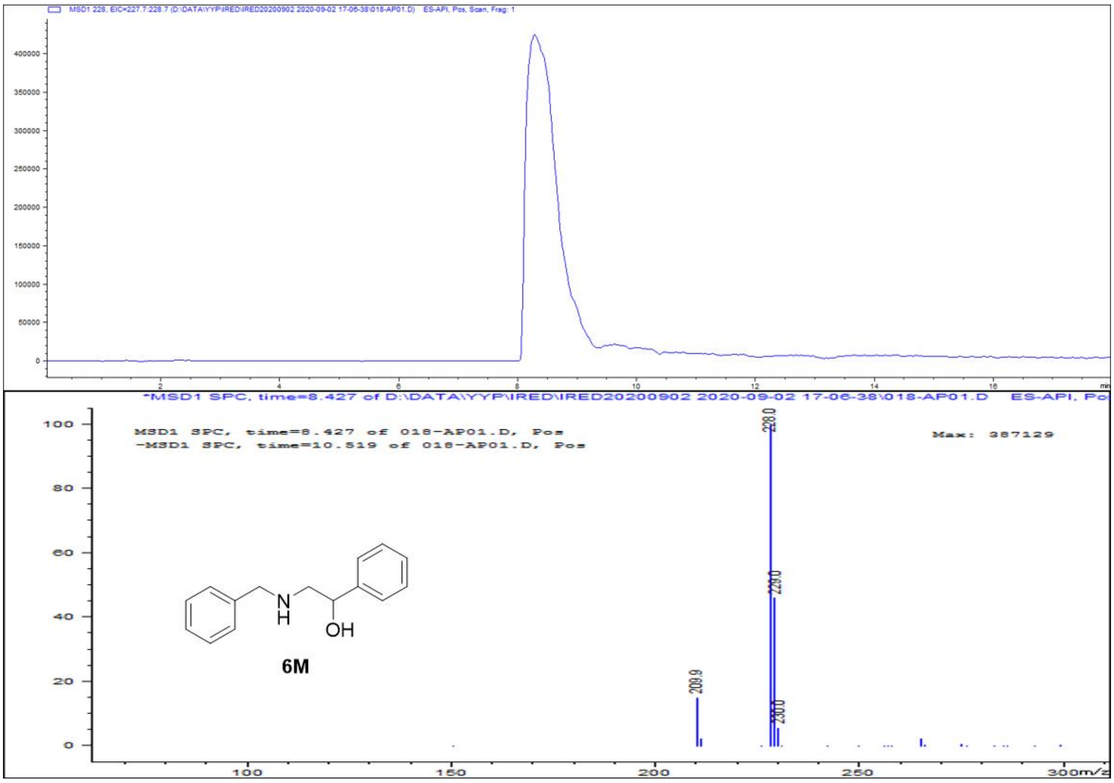

Figure 59. LCMS analysis: IR-G02-catalysed reductive amination of **6** with **M**, showing amine product **6M**.

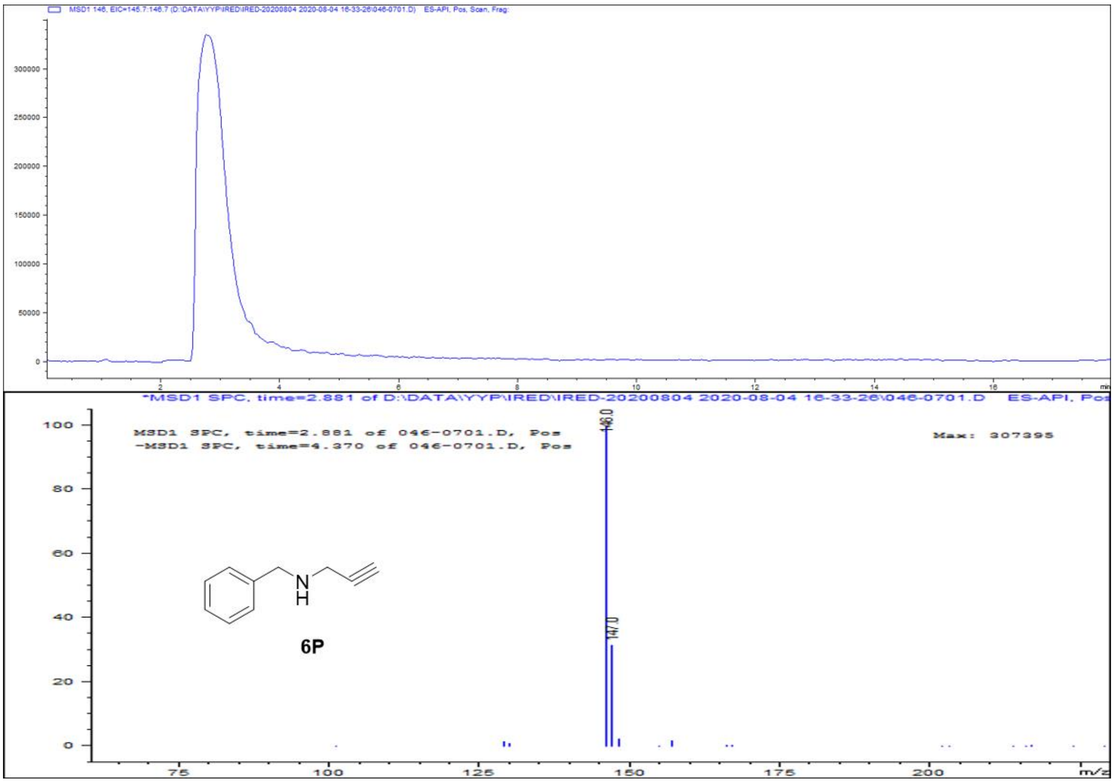

Figure 60. LCMS analysis: IR-G02-catalysed reductive amination of **6** with**P**, showing amine product **6P**.

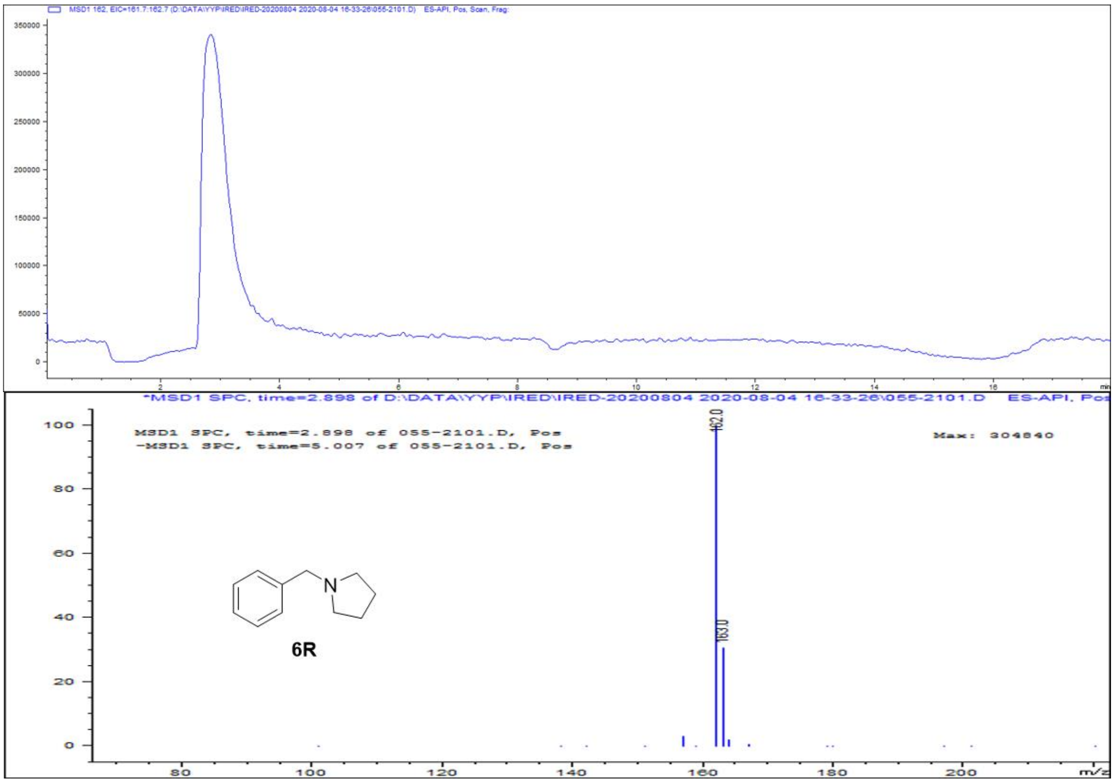

Figure 61. LCMS analysis: IR-G02-catalysed reductive amination of **6** with **R**, showing amine product **6R**.

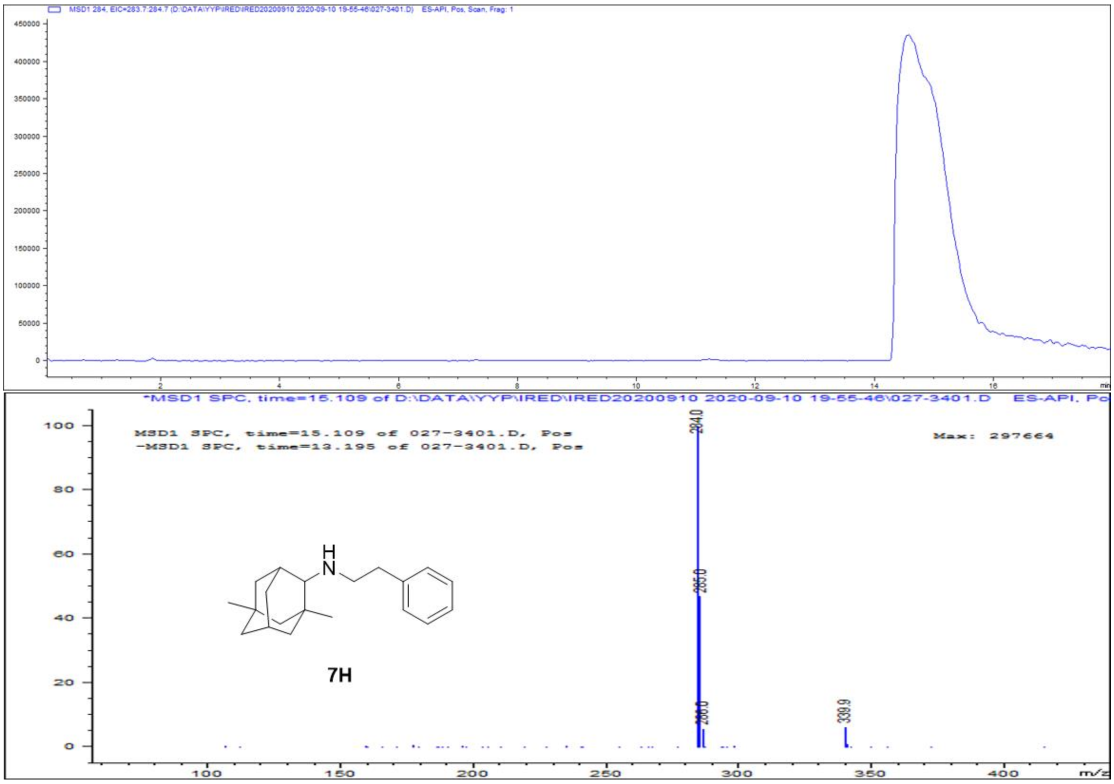

Figure 62. LCMS analysis: IR-G02-catalysed reductive amination of **7** with **H**, showing amine product **7H**.

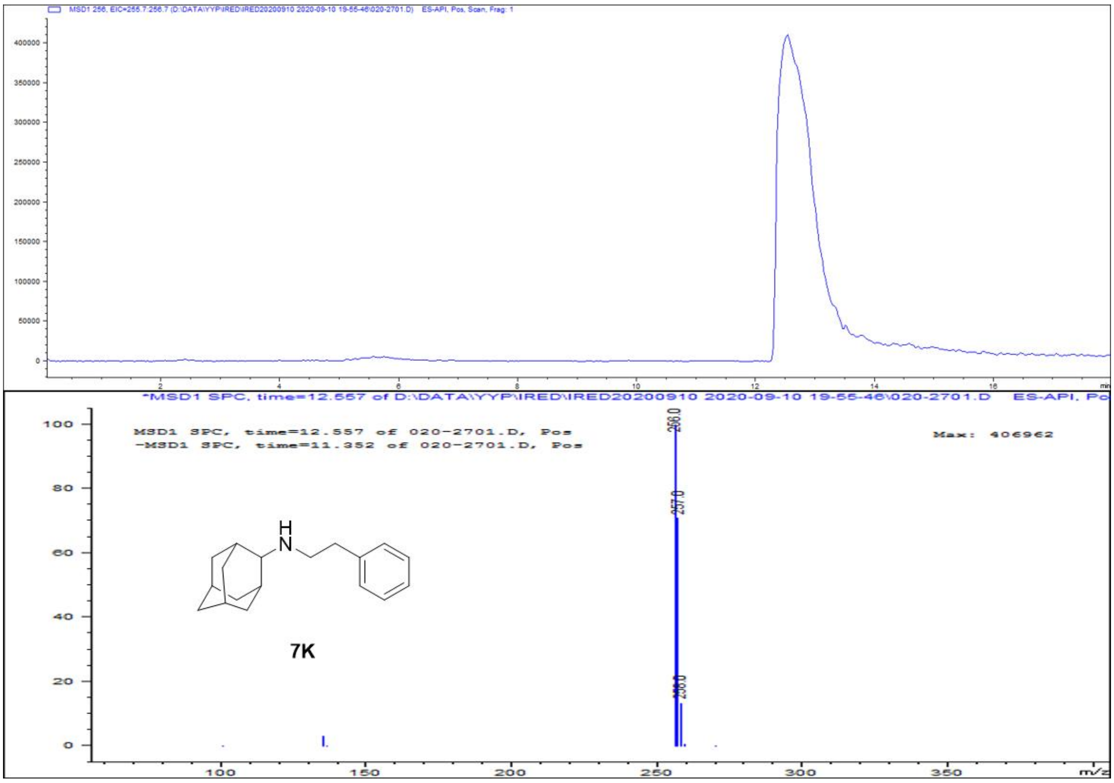

Figure 63. LCMS analysis: IR-G02-catalysed reductive amination of **7** with **K**, showing amine product **7K**.

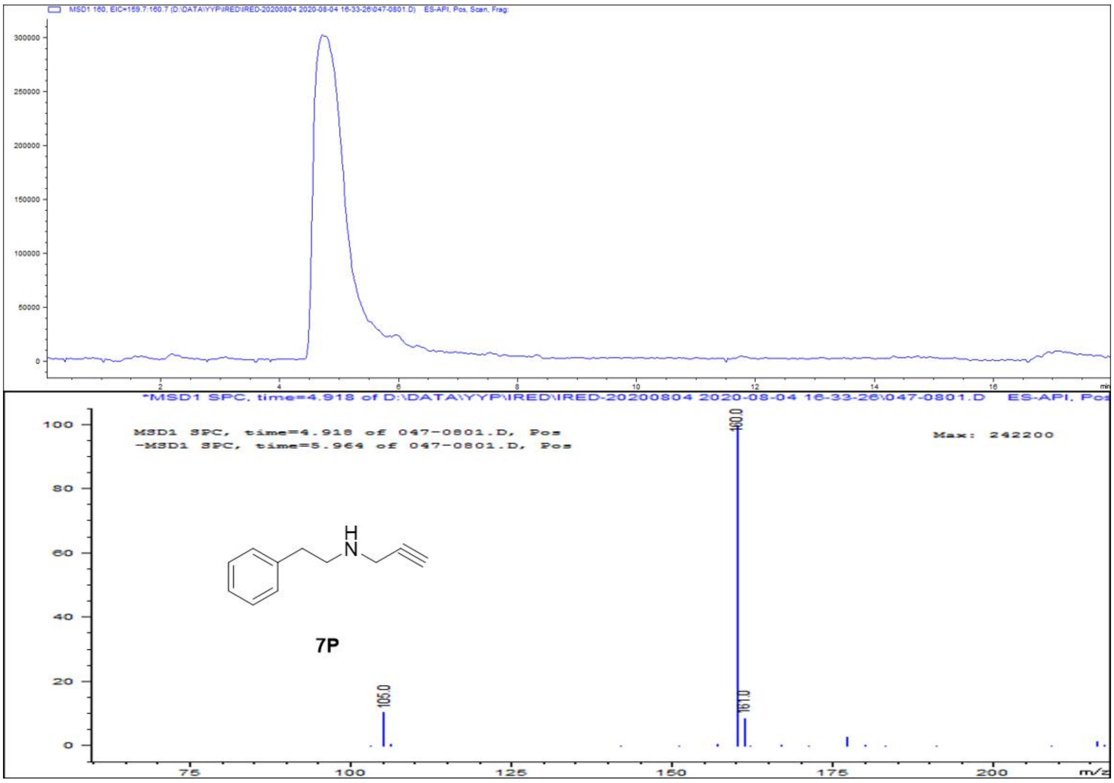

Figure 64. LCMS analysis: IR-G02-catalysed reductive amination of **7** with **P**, showing amine product **7P**.

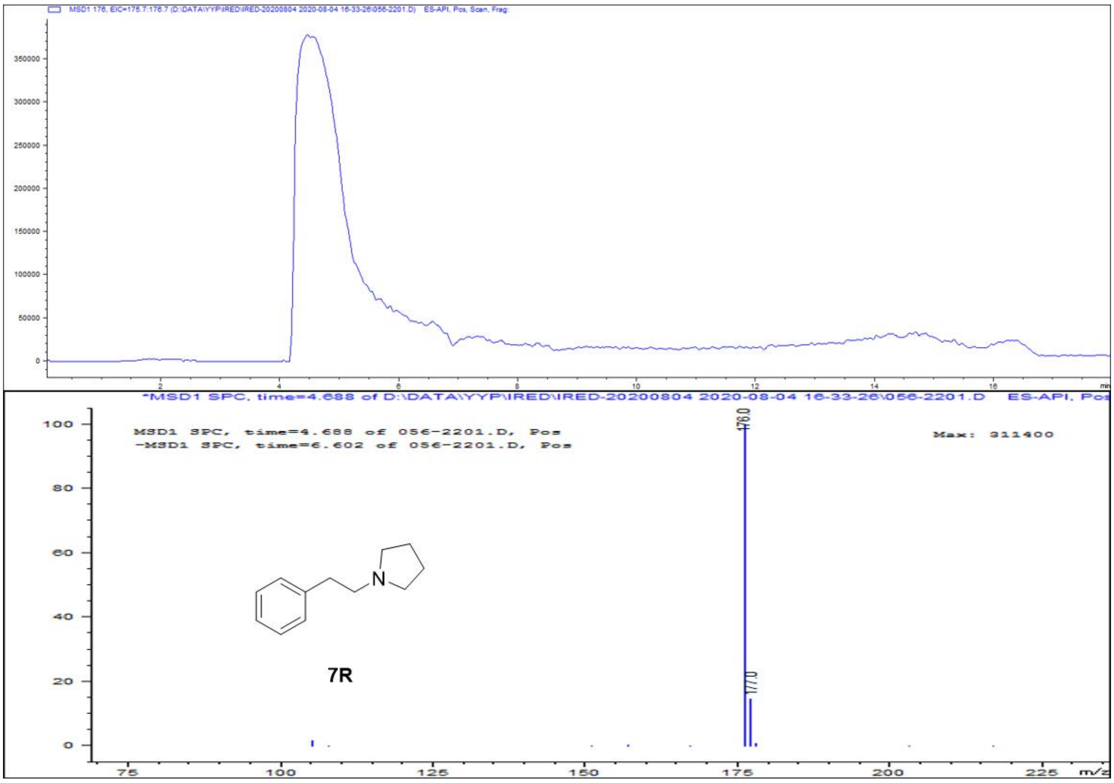

Figure 65. LCMS analysis: IR-G02-catalysed reductive amination of **7** with **R**, showing amine product **7R**.

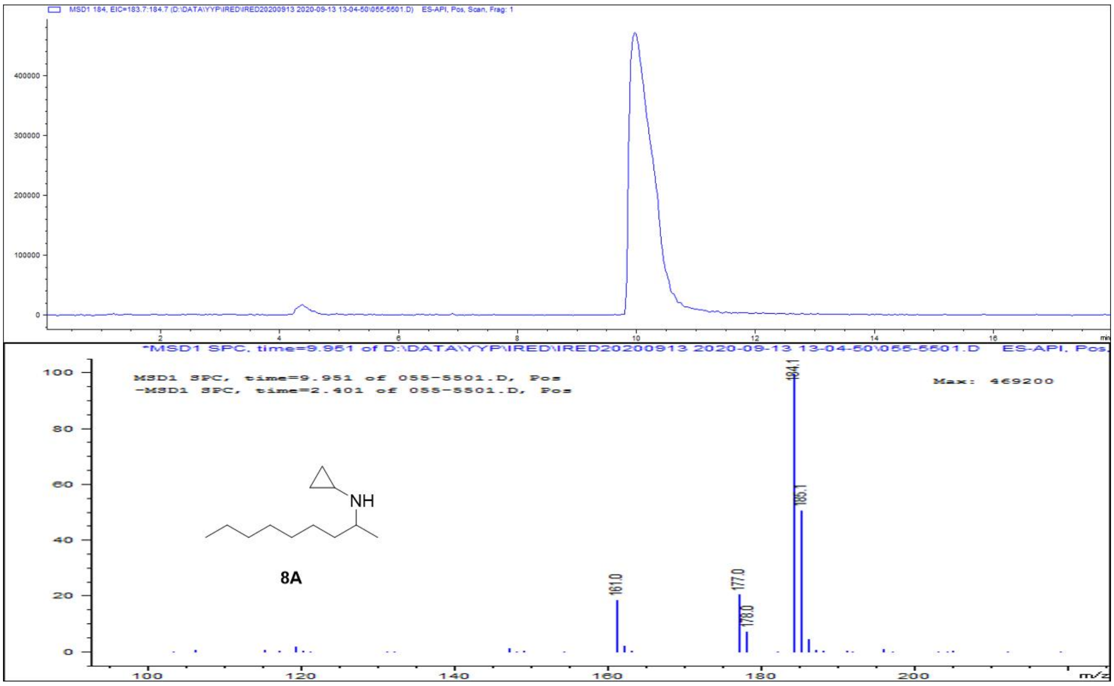

Figure 66. LCMS analysis: IR-G02-catalysed reductive amination of **8** with **A**, showing amine product **8A**.

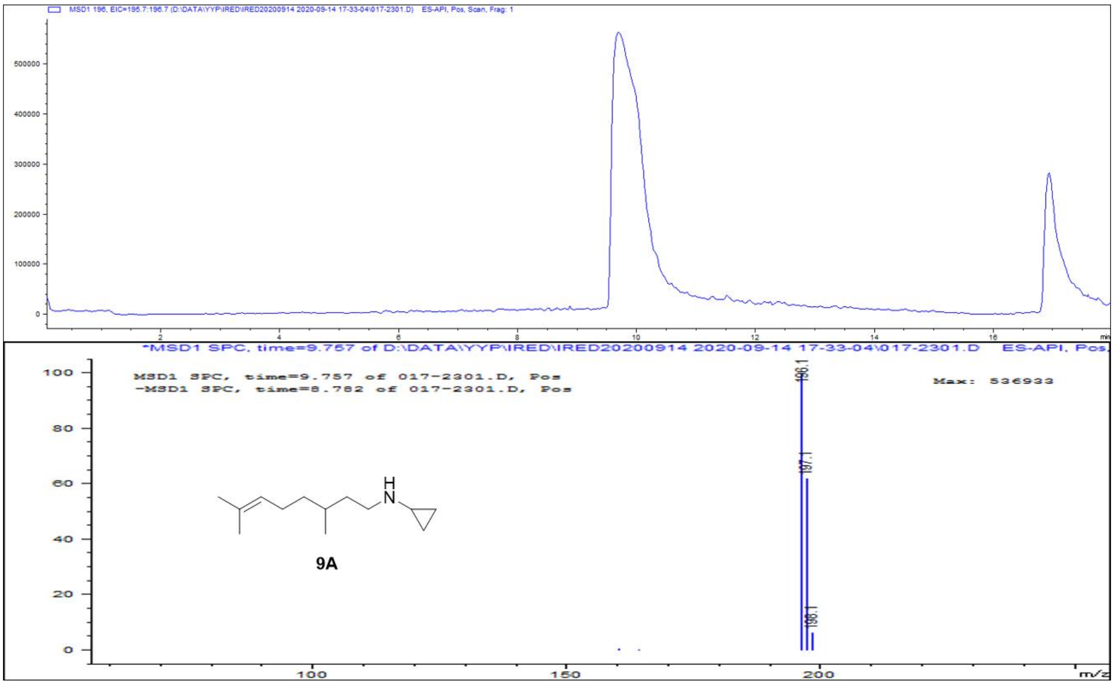

Figure 67. LCMS analysis: IR-G02-catalysed reductive amination of **9** with **A**, showing amine product **9A**.

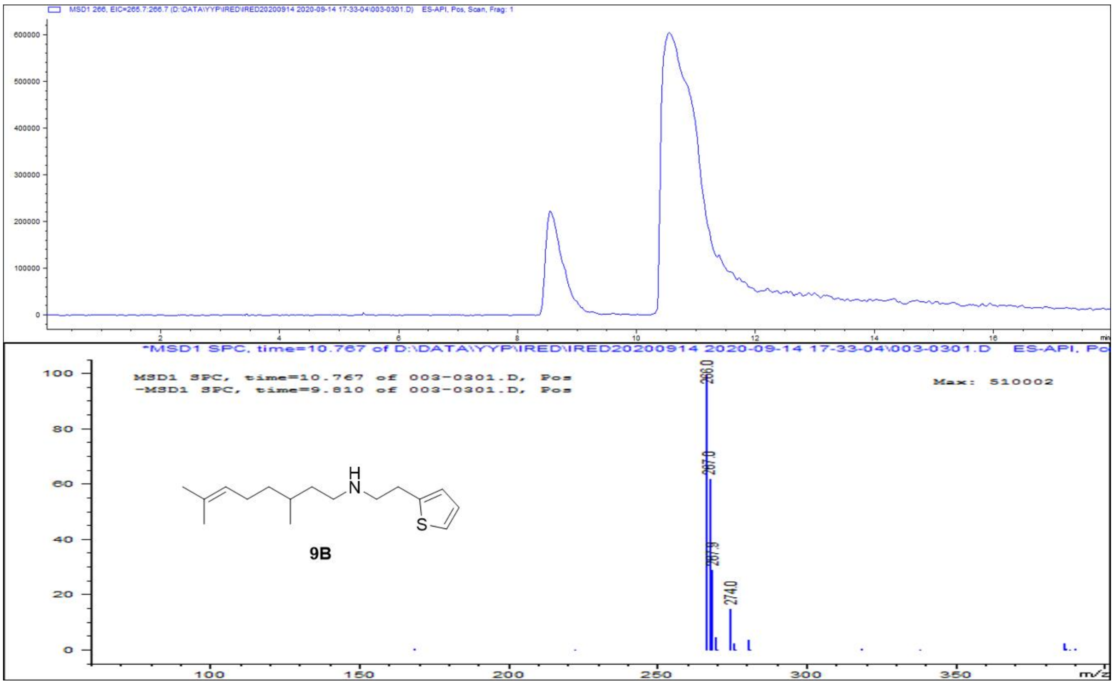

Figure 68. LCMS analysis: IR-G02-catalysed reductive amination of **9** with **B**, showing amine product **9B**.

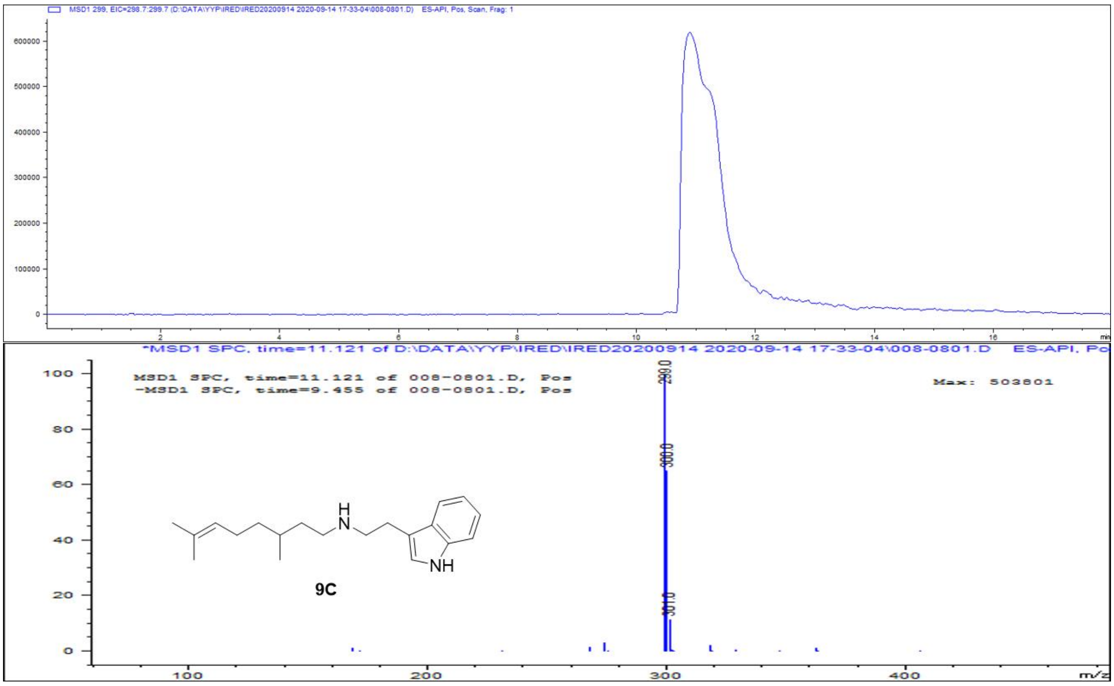

Figure 69. LCMS analysis: IR-G02-catalysed reductive amination of **9** with **C**, showing amine product **9C**.

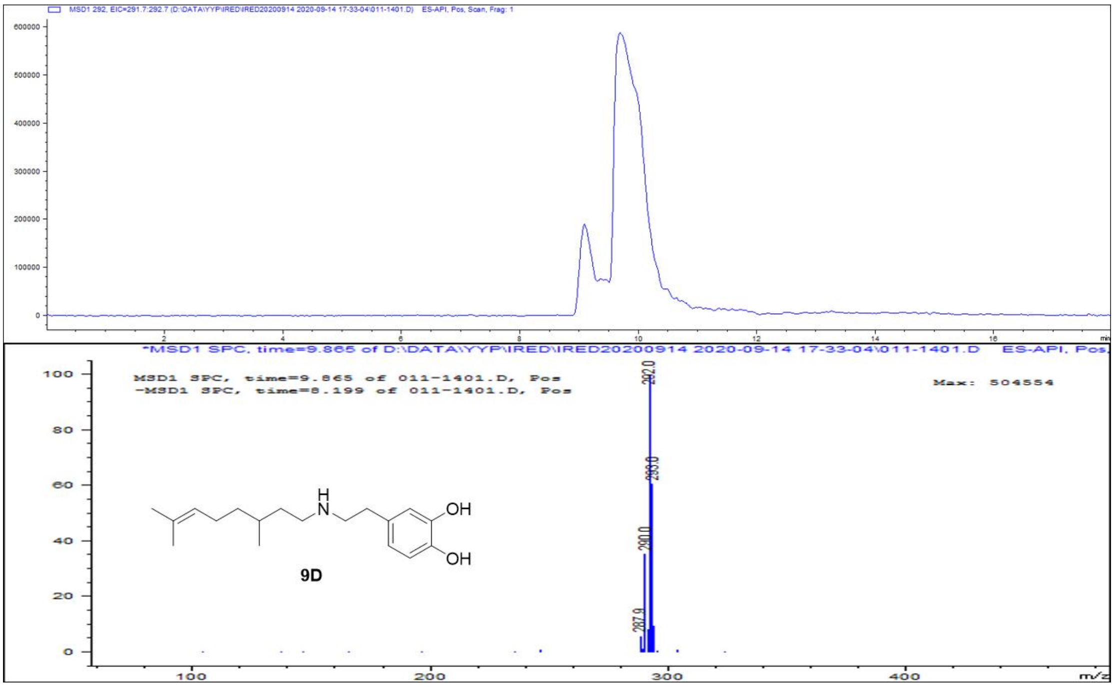

Figure 70. LCMS analysis: IR-G02-catalysed reductive amination of **9** with **D**, showing amine product **9D**.

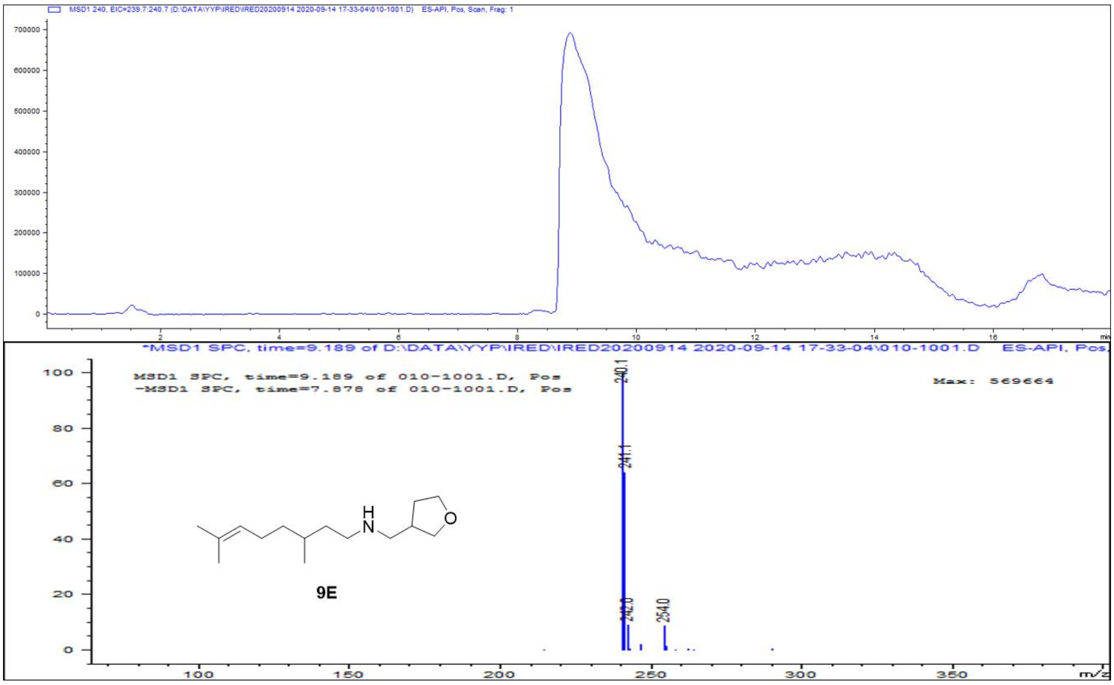

Figure 71. LCMS analysis: IR-G02-catalysed reductive amination of **9** with **E**, showing amine product **9E**.

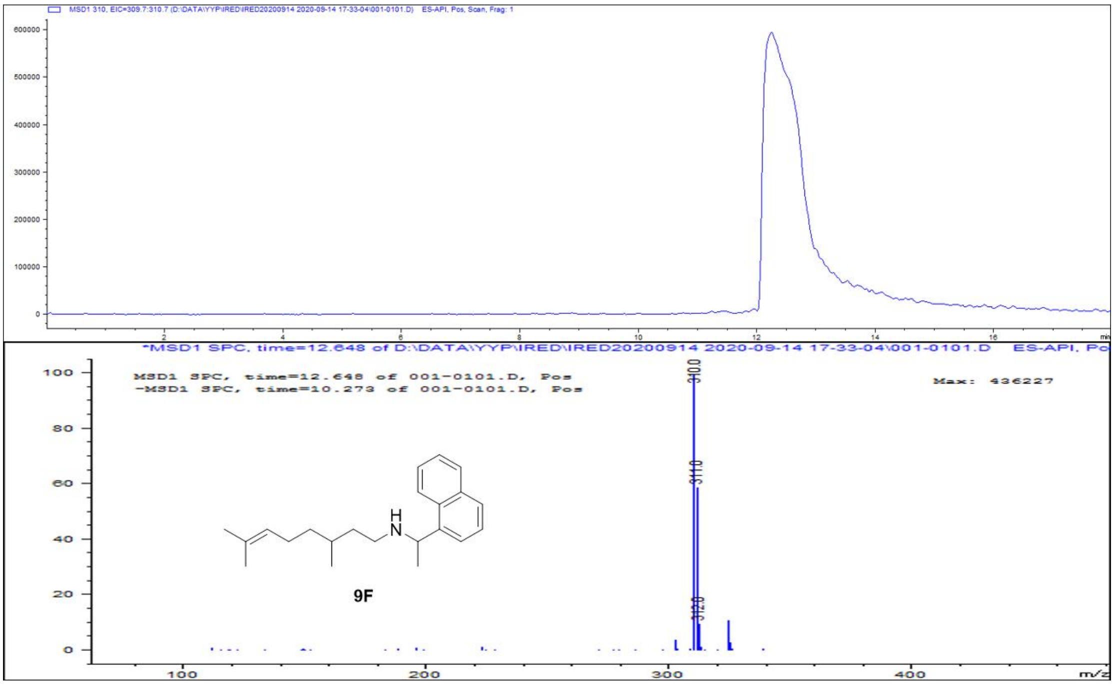

Figure 72. LCMS analysis: IR-G02-catalysed reductive amination of **9** with **F**, showing amine product **9F**.

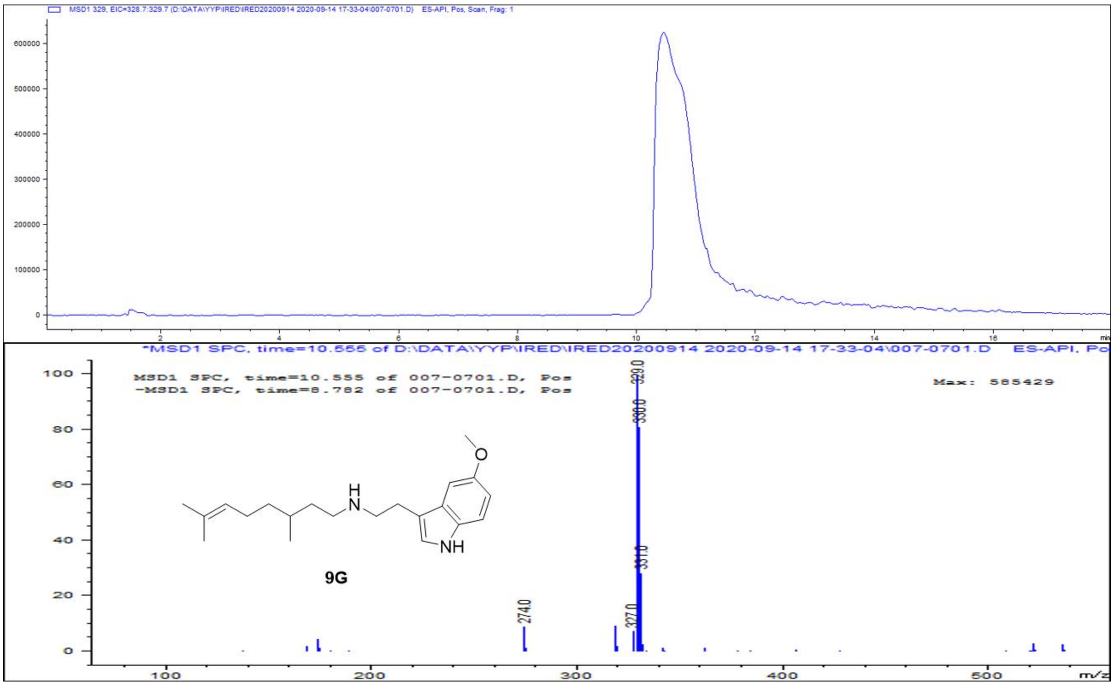

Figure 73. LCMS analysis: IR-G02-catalysed reductive amination of **9** with **G**, showing amine product **9G**.

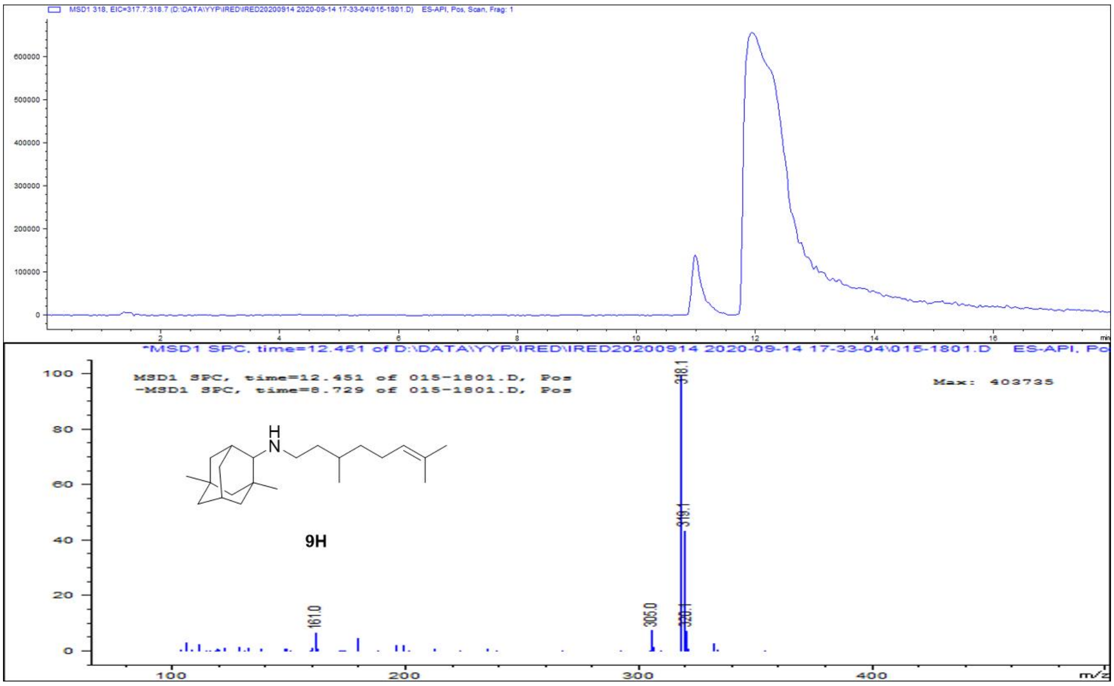

Figure 74. LCMS analysis: IR-G02-catalysed reductive amination of **9** with **H**, showing amine product **9H**.

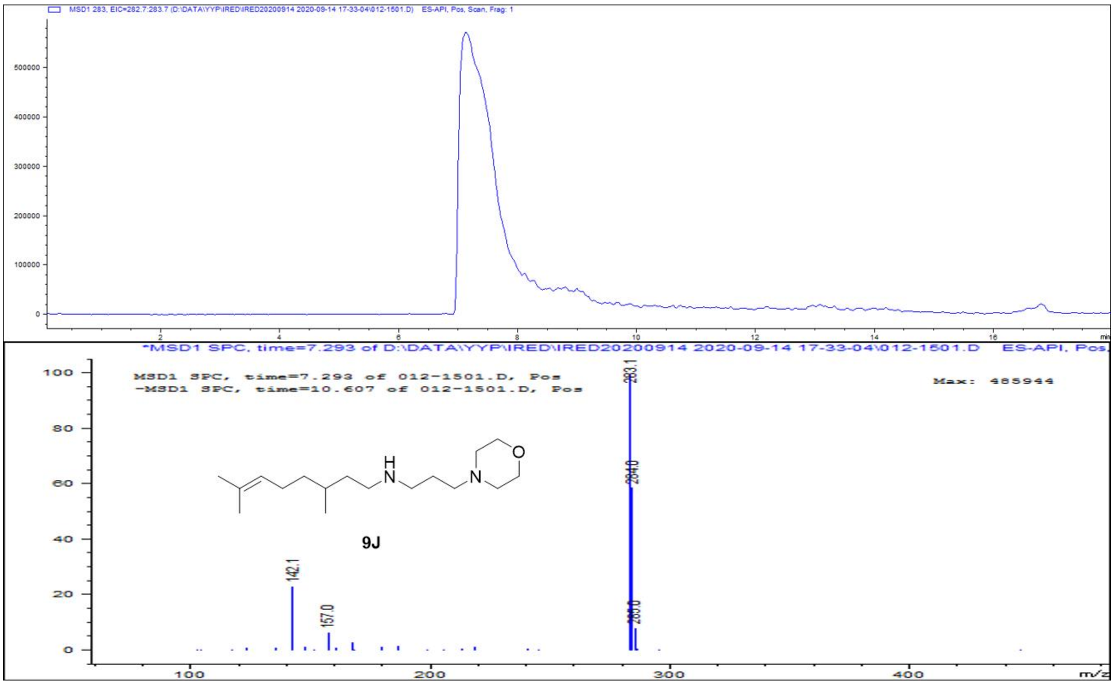

Figure 75. LCMS analysis: IR-G02-catalysed reductive amination of **9** with **J**, showing amine product **9J**.

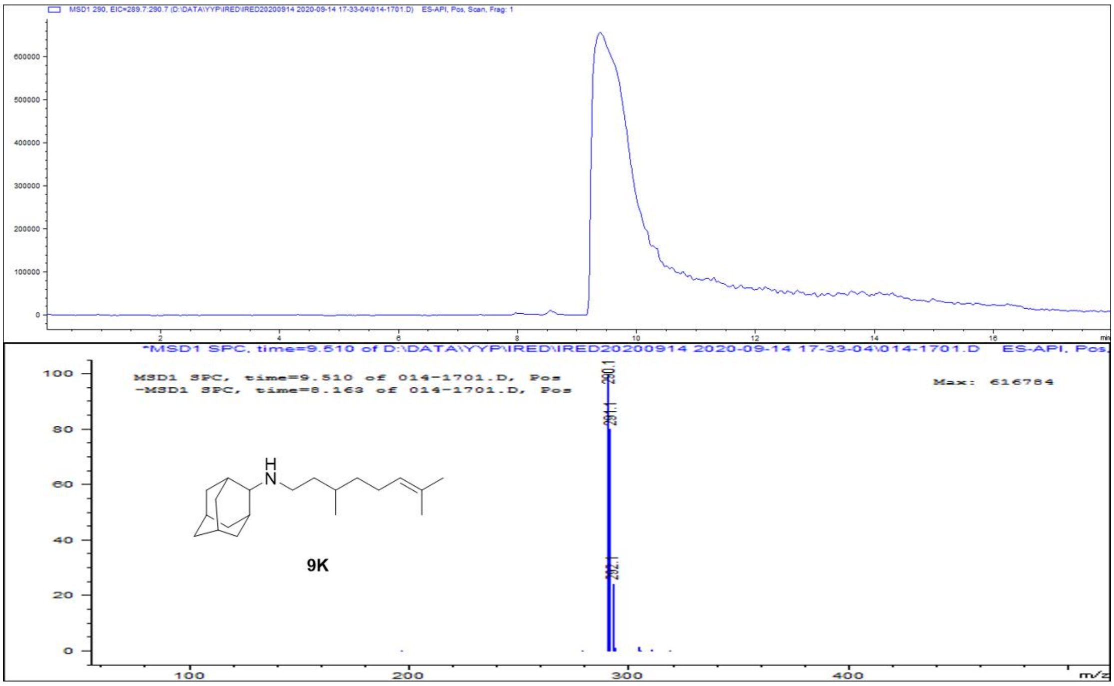

Figure 76. LCMS analysis: IR-G02-catalysed reductive amination of **9** with **K**, showing amine product **9K**.

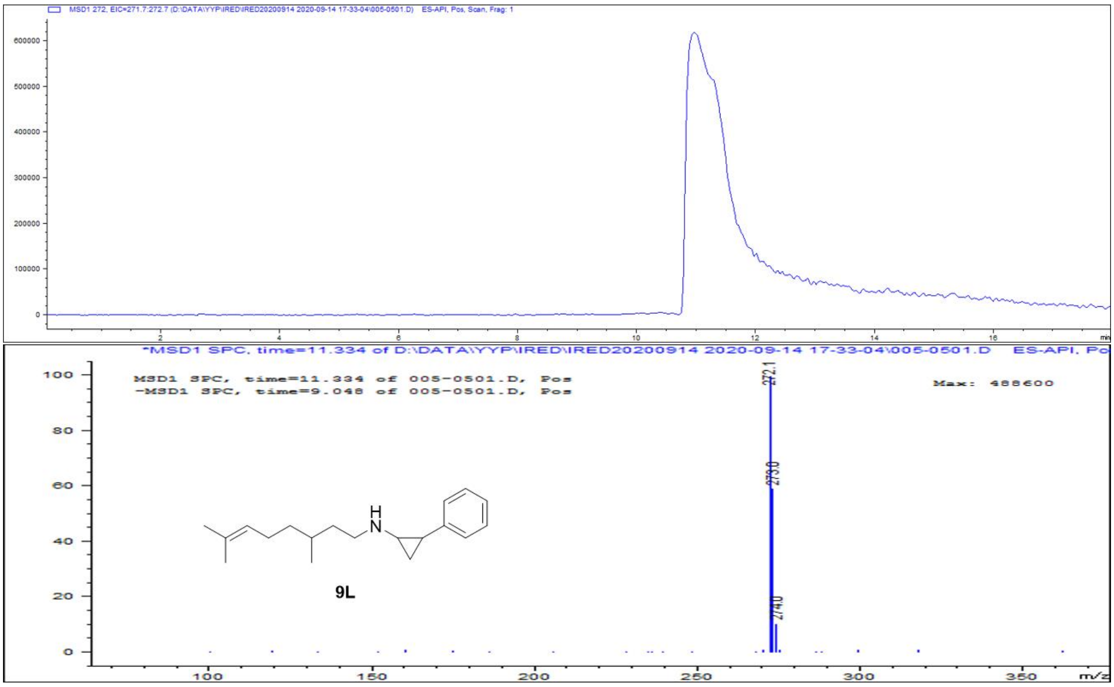

Figure 77. LCMS analysis: IR-G02-catalysed reductive amination of **9** with **L**, showing amine product **9L**.

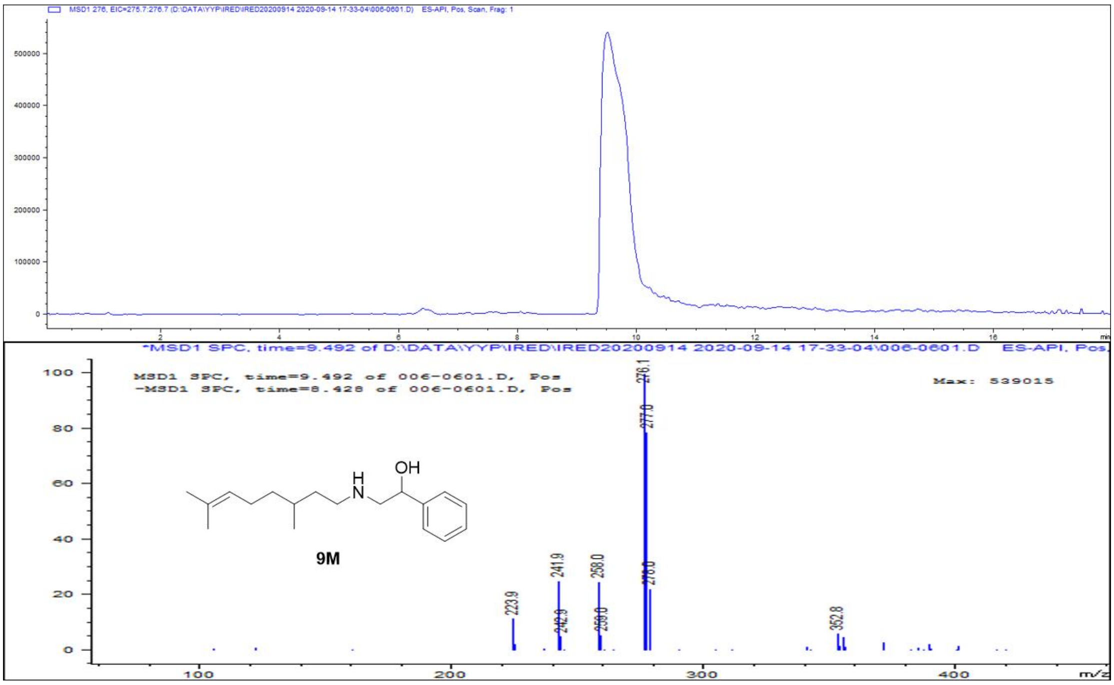

Figure 78. LCMS analysis: IR-G02-catalysed reductive amination of **9** with **M**, showing amine product **9M**.

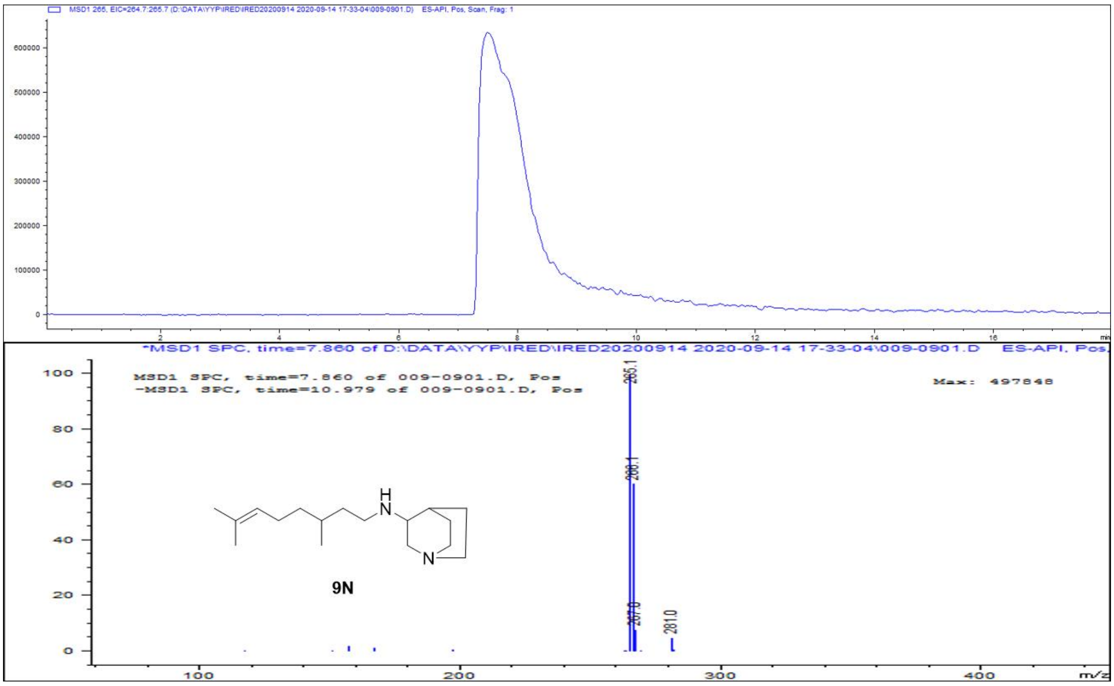

Figure 79. LCMS analysis: IR-G02-catalysed reductive amination of **9** with **N**, showing amine product **9N**.

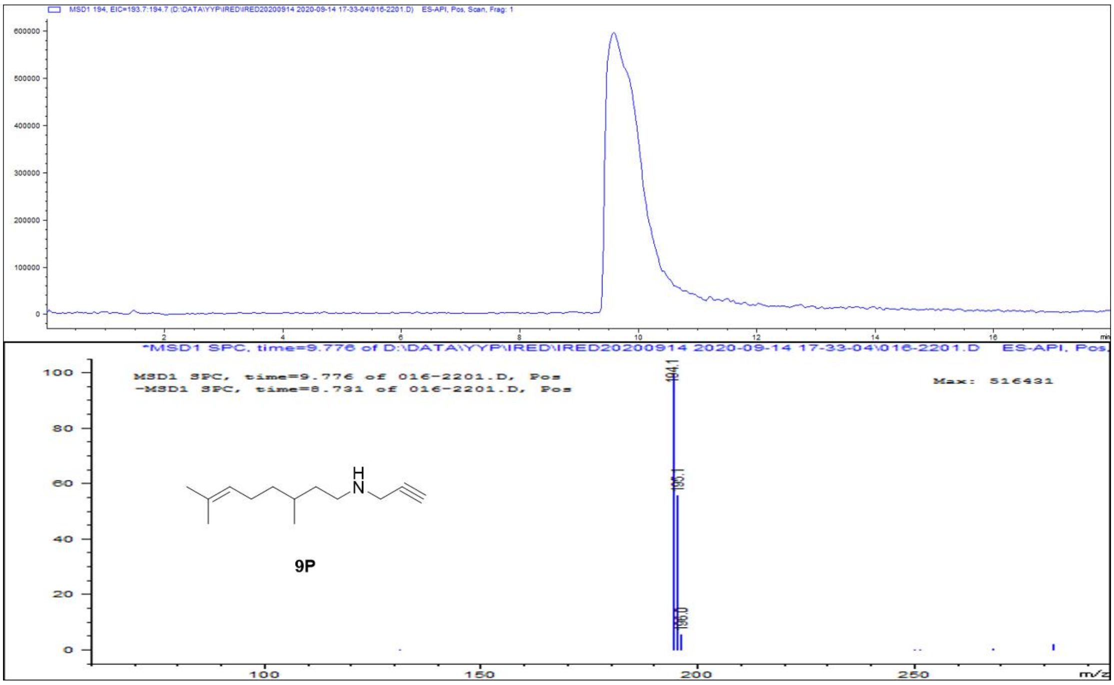

Figure 80. LCMS analysis: IR-G02-catalysed reductive amination of **9** with **P**, showing amine product **9P**.

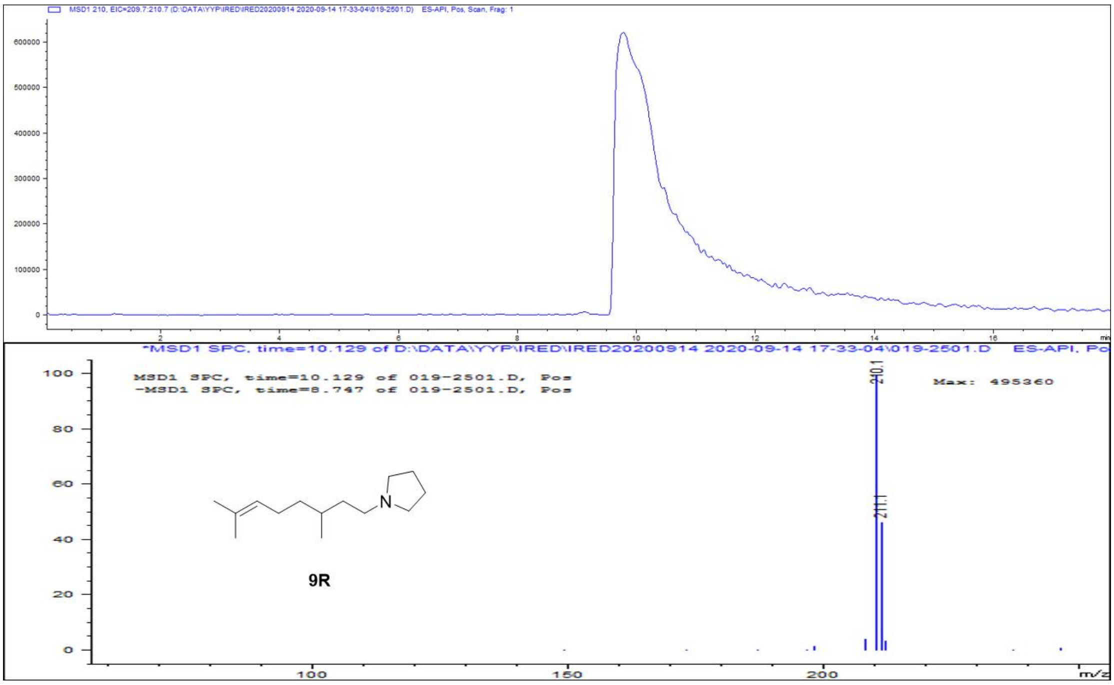

Figure 81. LCMS analysis: IR-G02-catalysed reductive amination of **9** with **R**, showing amine product **9R**.

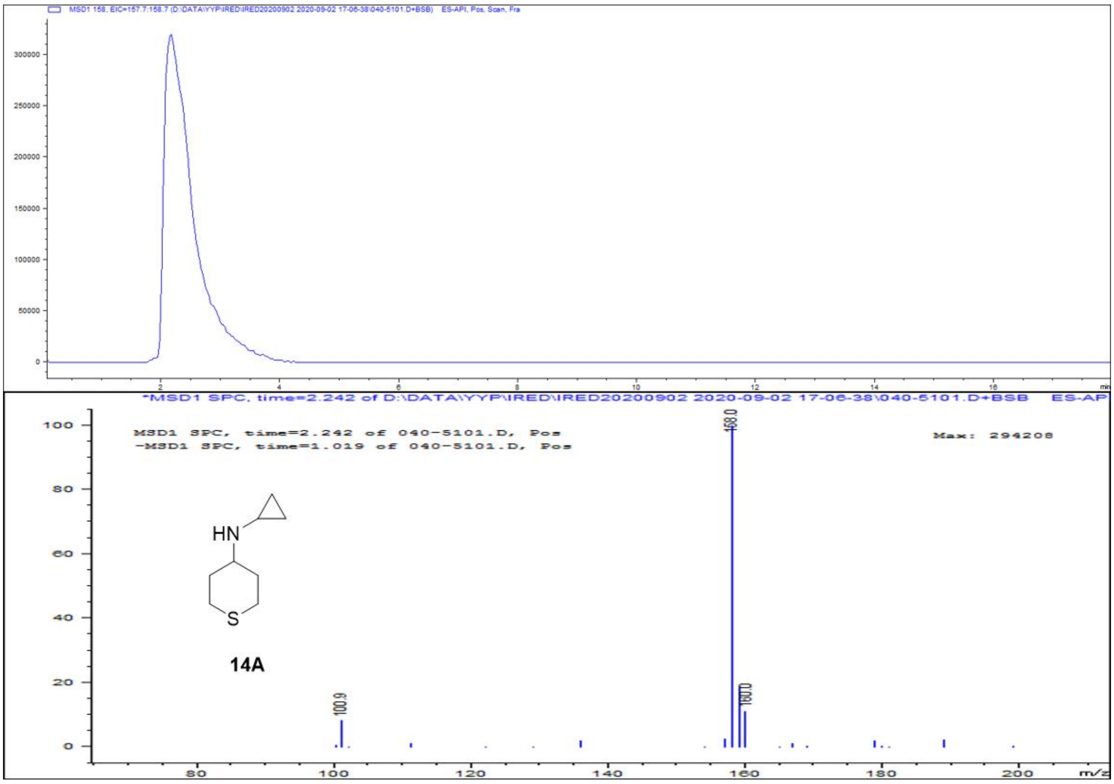

Figure 82. LCMS analysis: IR-G02-catalysed reductive amination of **14** with **A**, showing amine product **14A**.

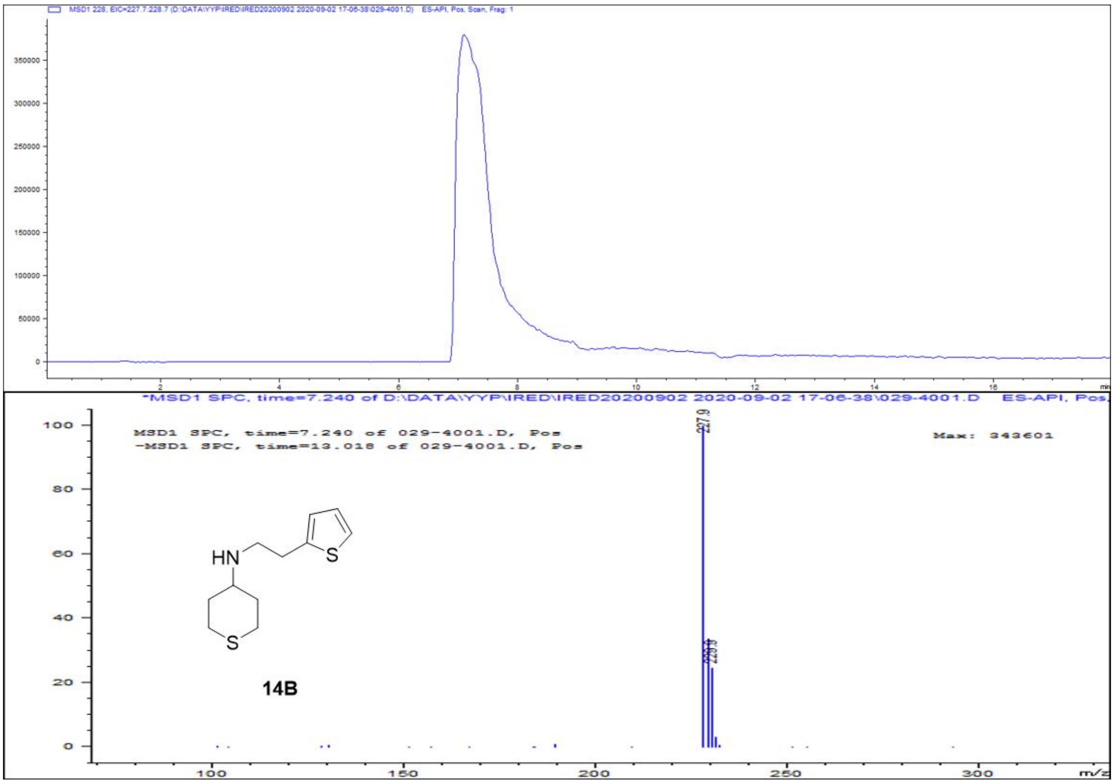

Figure 83. LCMS analysis: IR-G02-catalysed reductive amination of **14** with **B**, showing amine product **14B**.

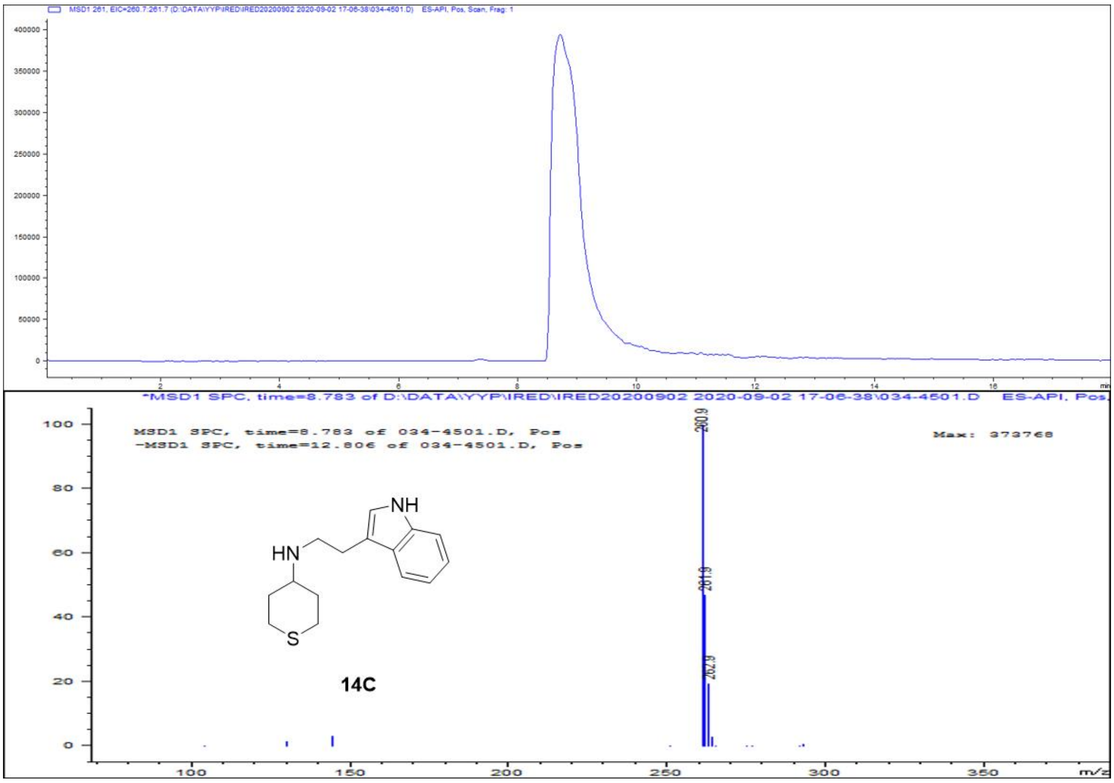

Figure 84. LCMS analysis: IR-G02-catalysed reductive amination of **14** with **C**, showing amine product **14C**.

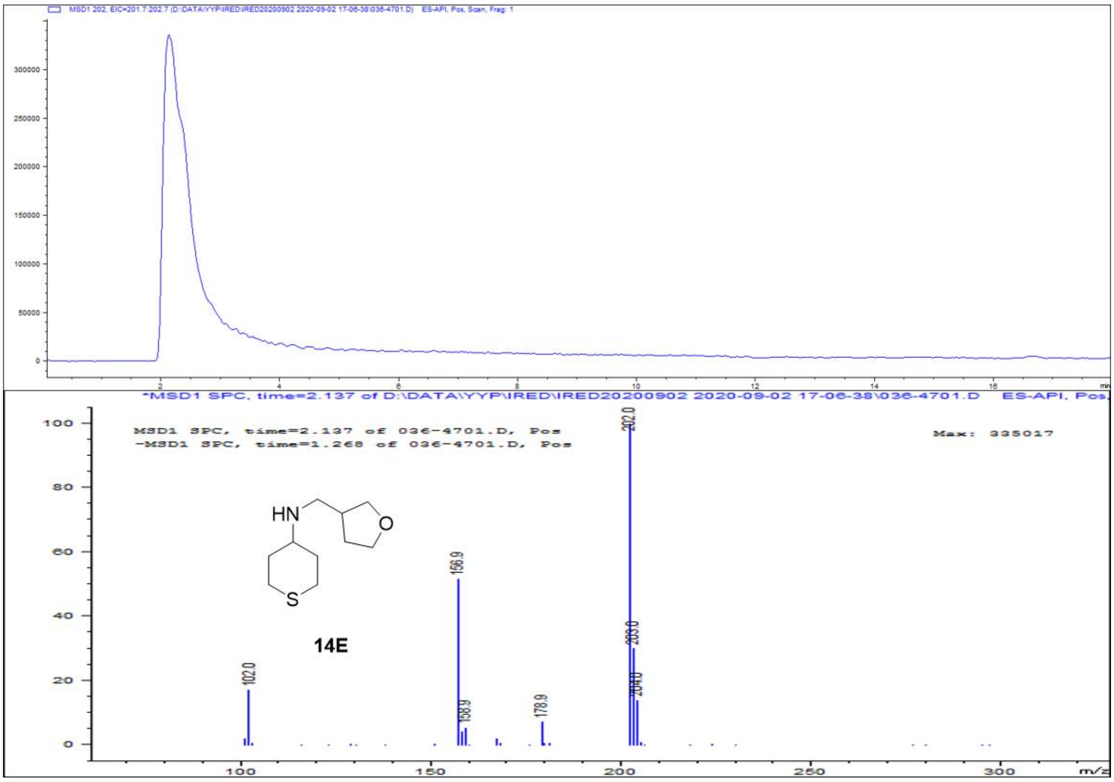

Figure 85. LCMS analysis: IR-G02-catalysed reductive amination of **14** with **E**, showing amine product **14E**.

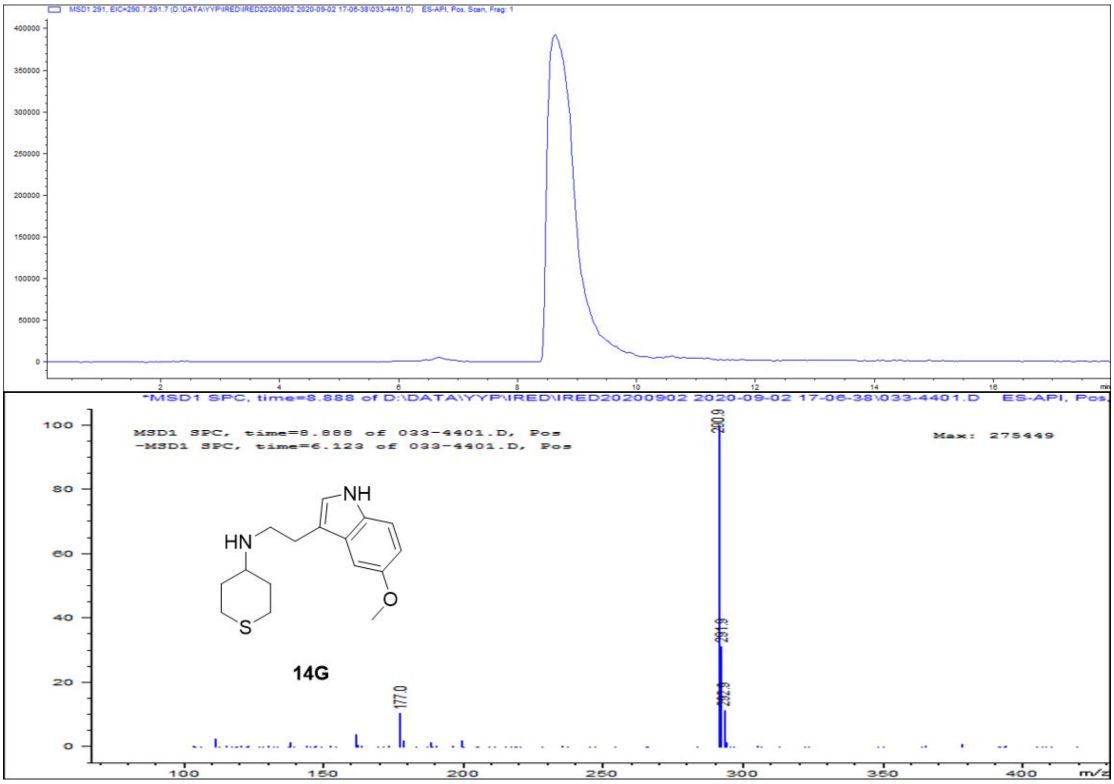

Figure 86. LCMS analysis: IR-G02-catalysed reductive amination of **14** with **G**, showing amine product **14G**.

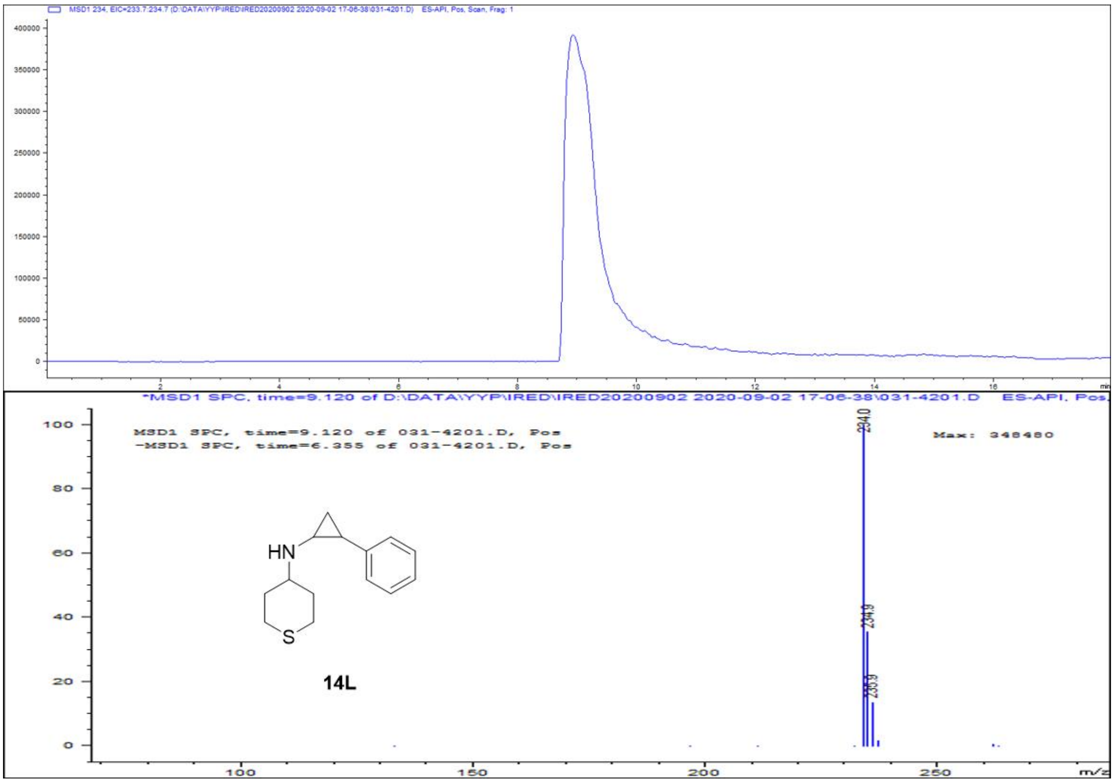

Figure 87. LCMS analysis: IR-G02-catalysed reductive amination of **14** with **L**, showing amine product **14L**.

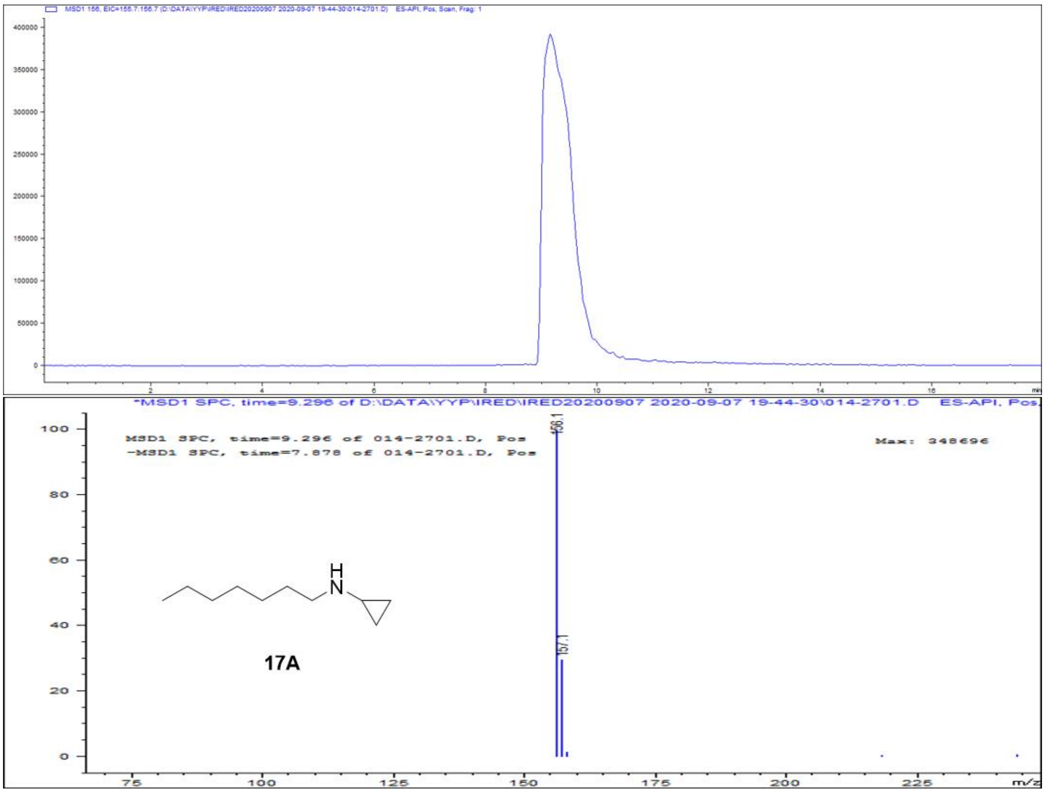

Figure 88. LCMS analysis: IR-G02-catalysed reductive amination of **17** with **A**, showing amine product **17A**.

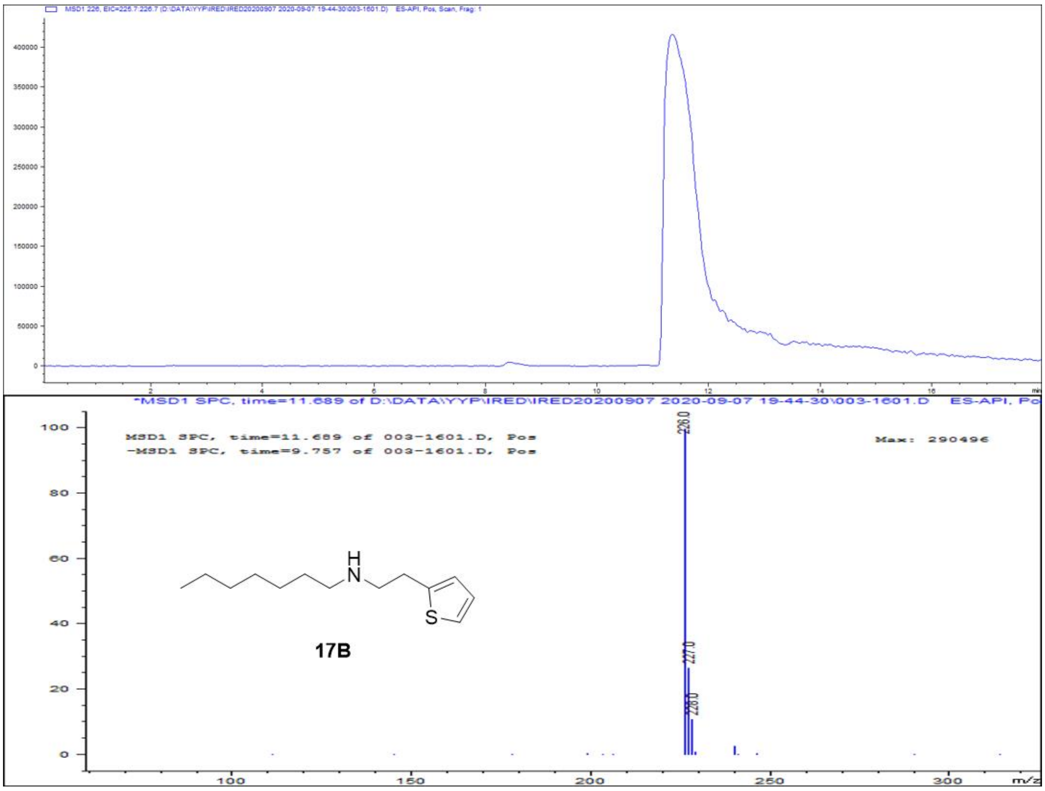

Figure 89. LCMS analysis: IR-G02-catalysed reductive amination of **17** with **B**, showing amine product **17B**.

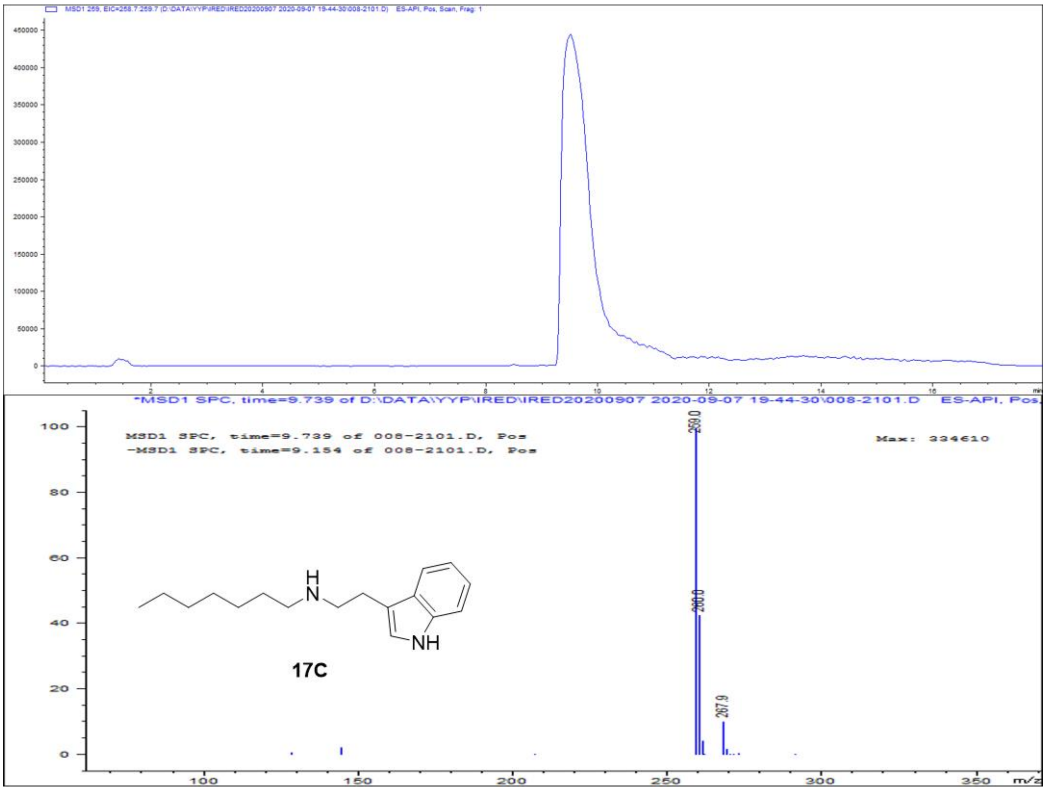

Figure 90. LCMS analysis: IR-G02-catalysed reductive amination of **17** with **C**, showing amine product **17C**.

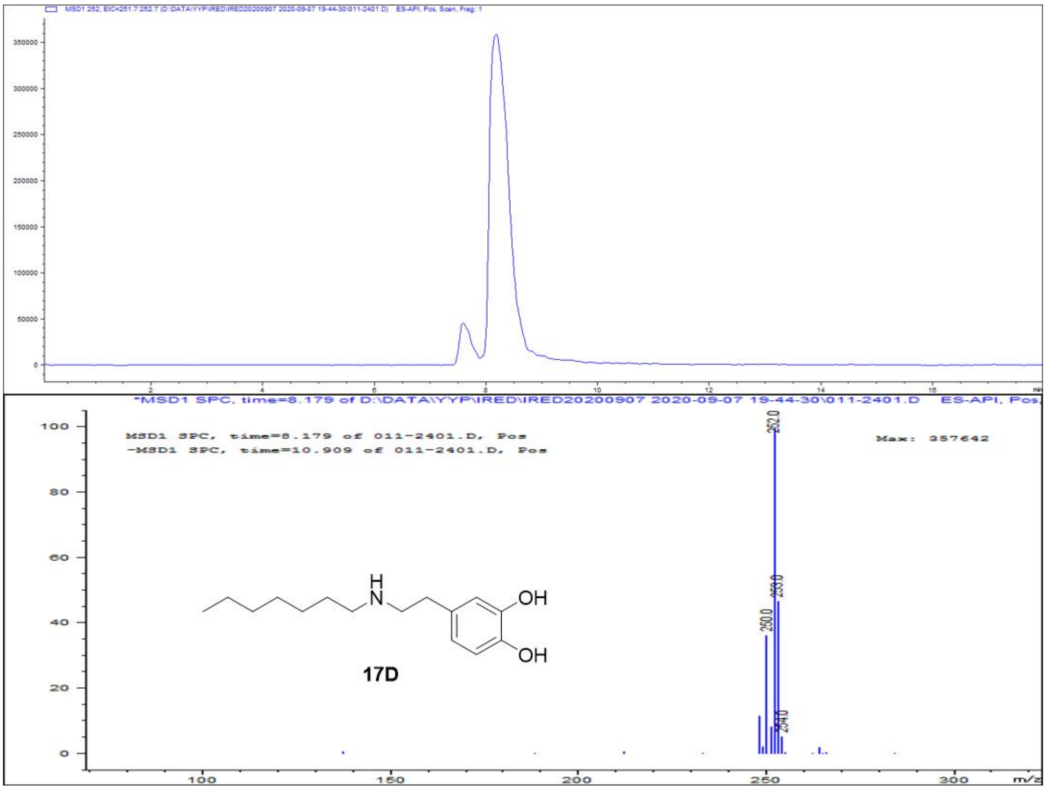

Figure 91. LCMS analysis: IR-G02-catalysed reductive amination of **17** with **D**, showing amine product **17D**.

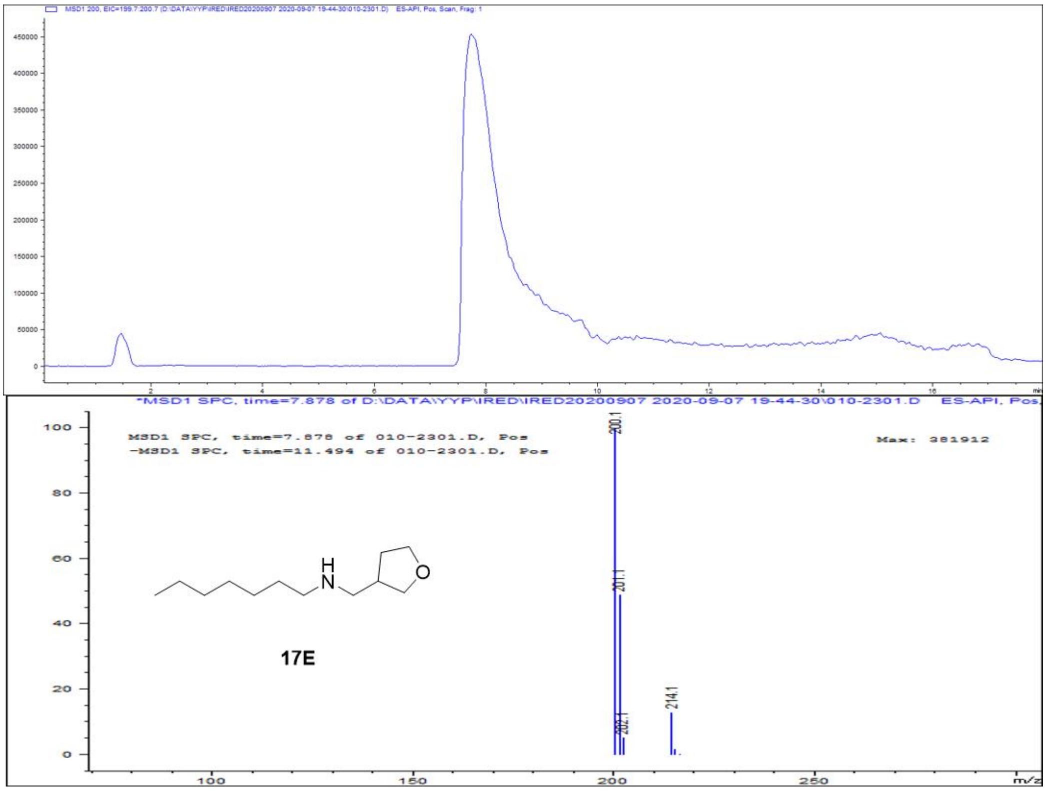

Figure 92. LCMS analysis: IR-G02-catalysed reductive amination of **17** with **E**, showing amine product **17E**.

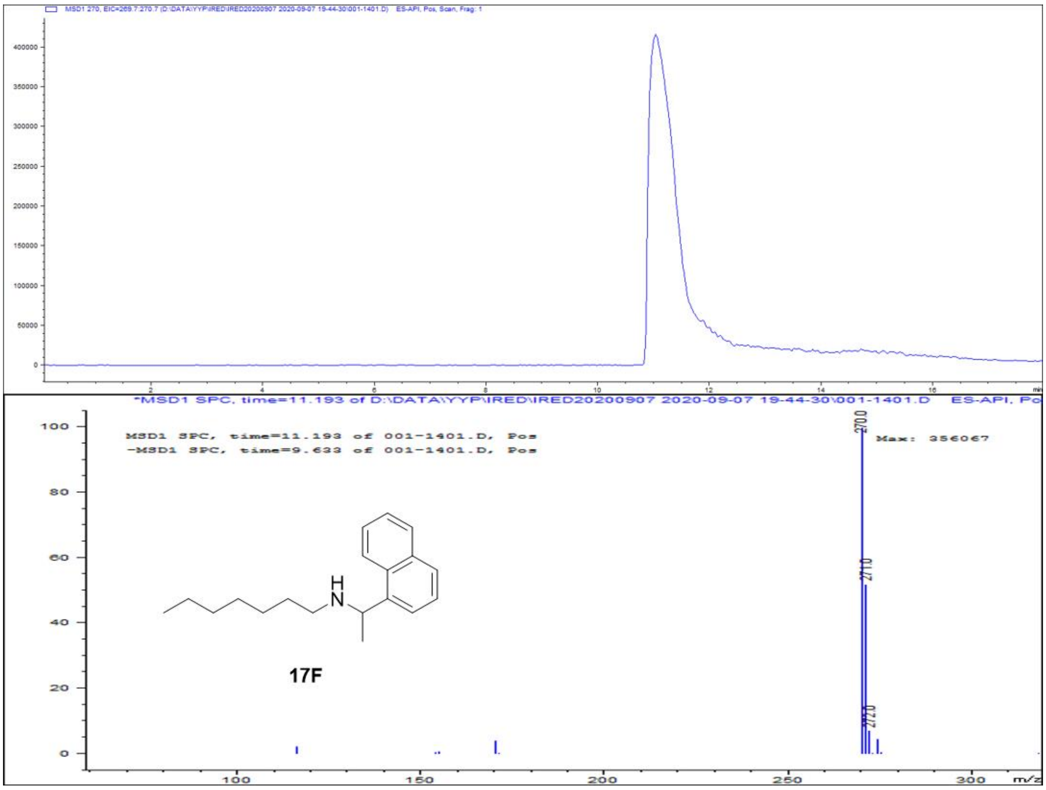

Figure 93. LCMS analysis: IR-G02-catalysed reductive amination of **17** with **F**, showing amine product **17F**.

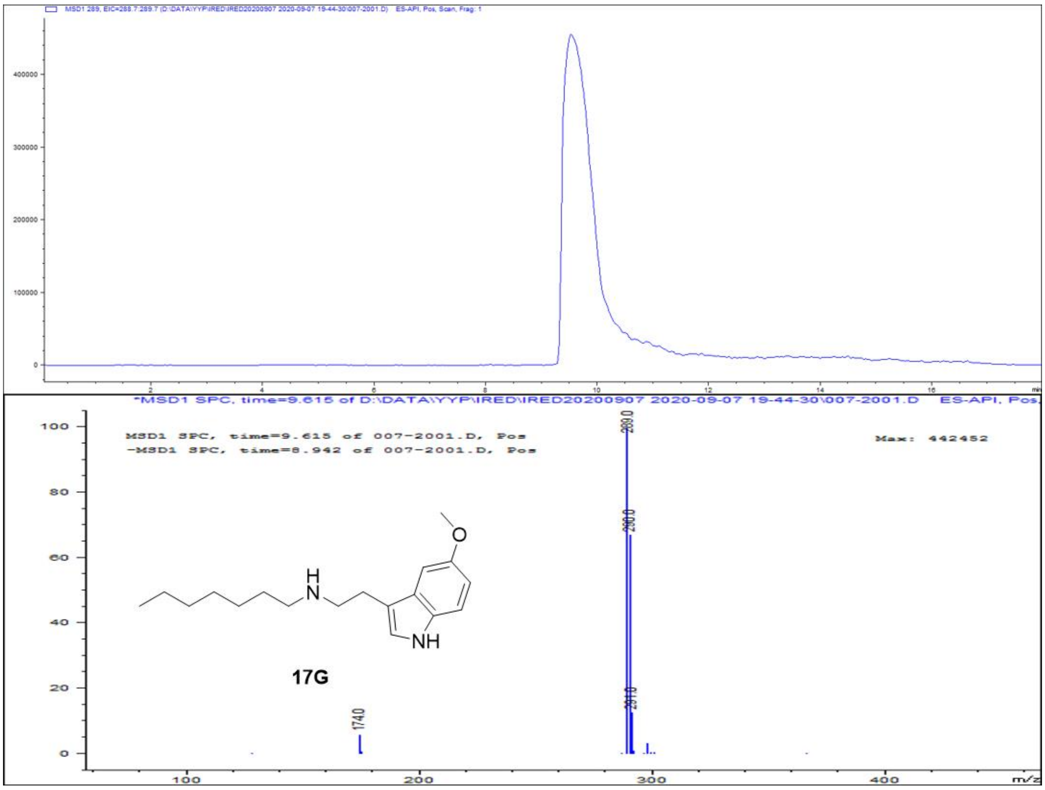

Figure 94. LCMS analysis: IR-G02-catalysed reductive amination of **17** with **G**, showing amine product **17G**.

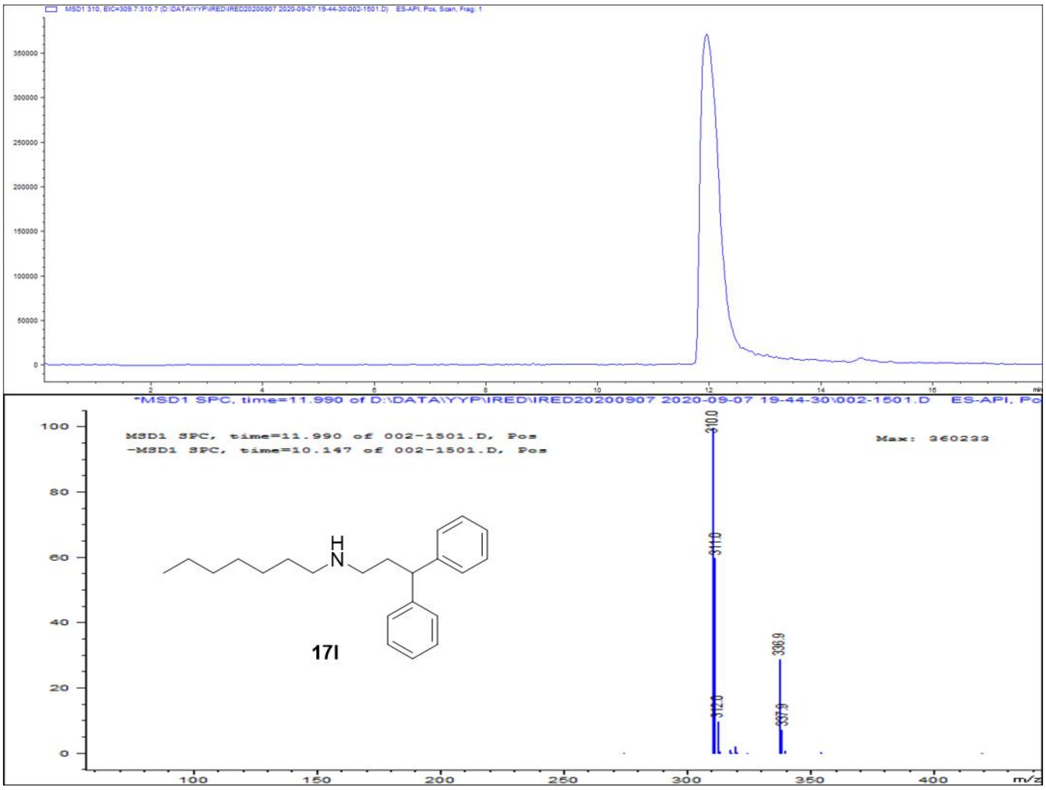

Figure 95. LCMS analysis: IR-G02-catalysed reductive amination of **17** with **I**, showing amine product **17I**.

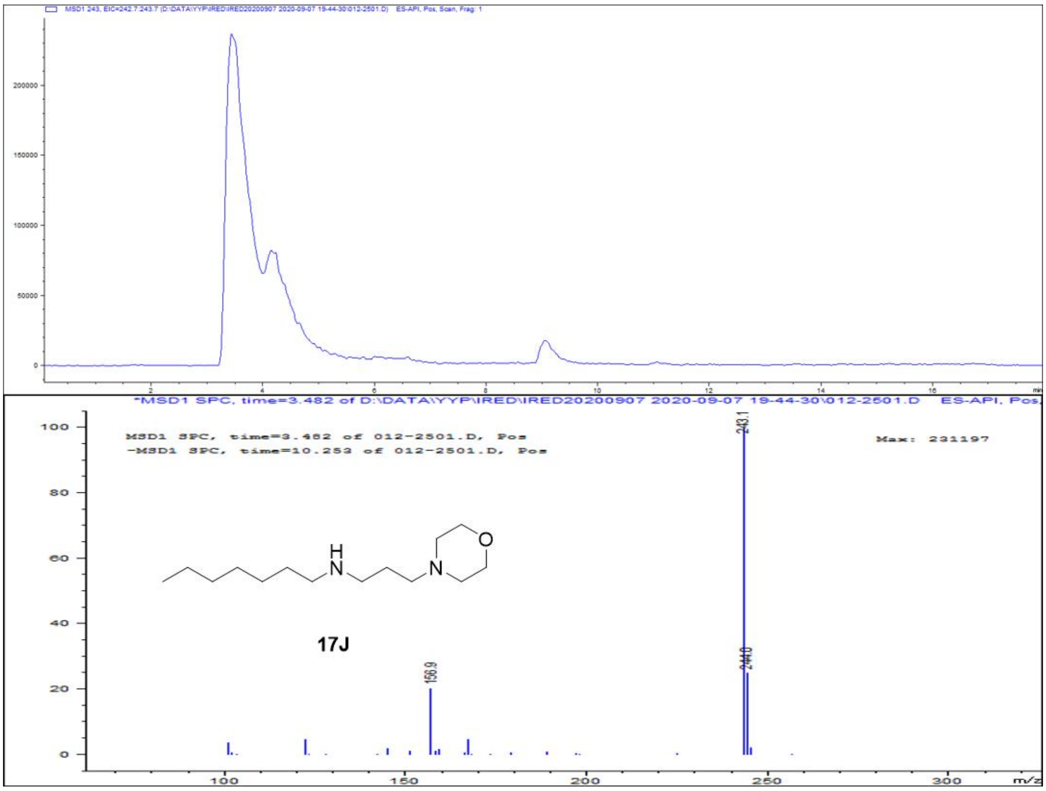

Figure 96. LCMS analysis: IR-G02-catalysed reductive amination of **17** with **J**, showing amine product **17J**.

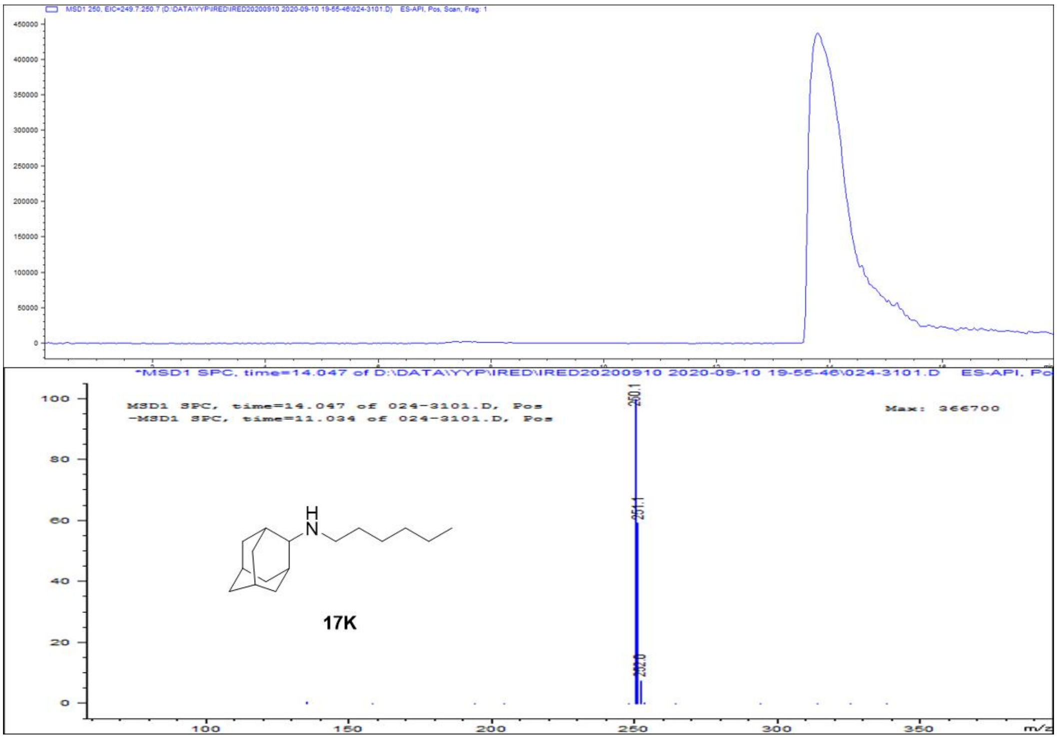

Figure 97. LCMS analysis: IR-G02-catalysed reductive amination of **17** with **K**, showing amine product **17K**.

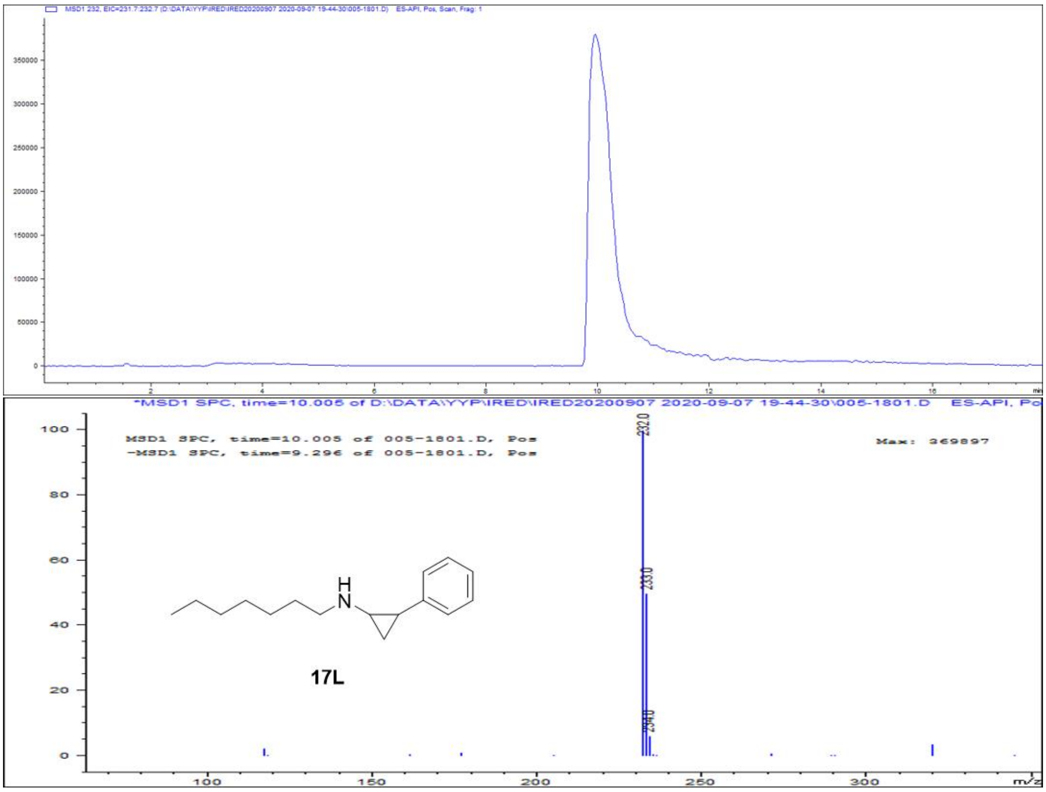

Figure 98. LCMS analysis: IR-G02-catalysed reductive amination of **17** with **L**, showing amine product **17L**.

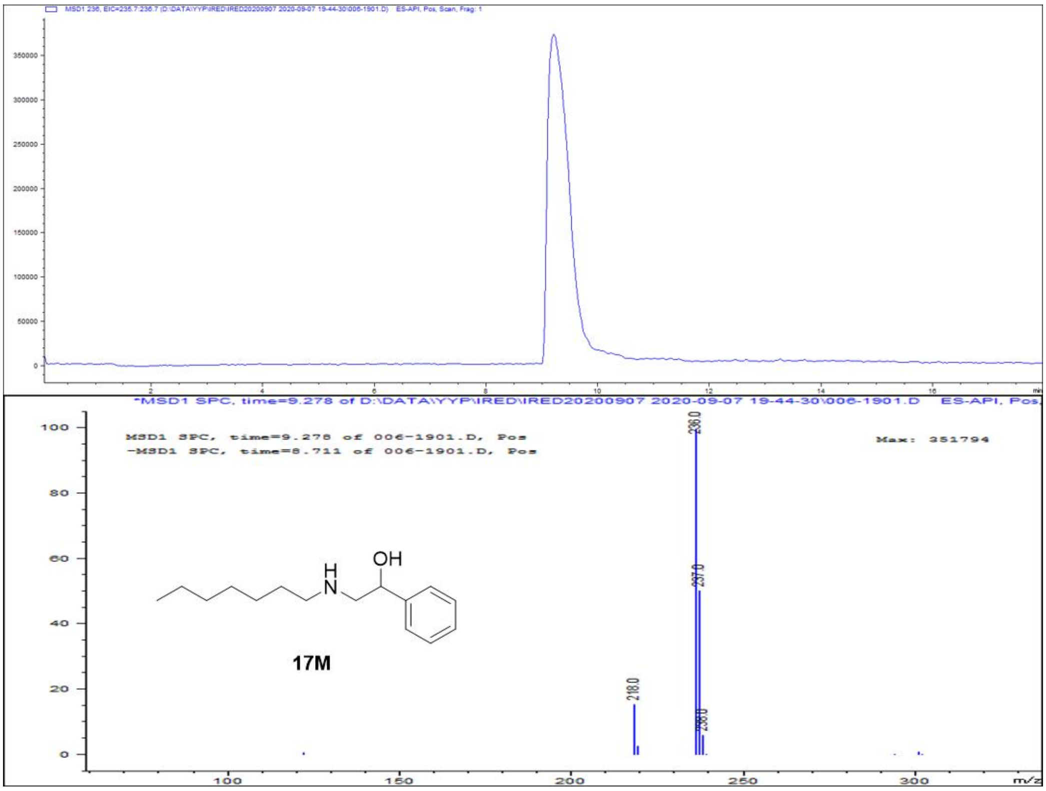

Figure 99. LCMS analysis: IR-G02-catalysed reductive amination of **17** with **M**, showing amine product **17M**.

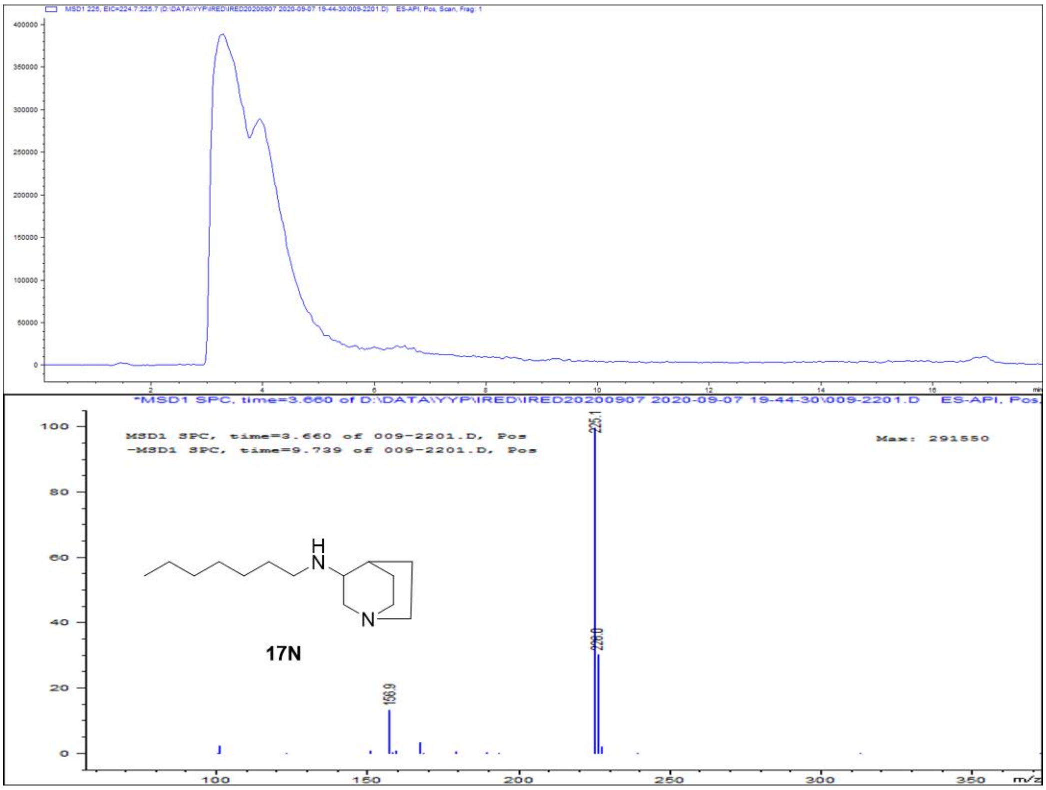

Figure 100. LCMS analysis: IR-G02-catalysed reductive amination of **17** with **N**, showing amine product **17N**.

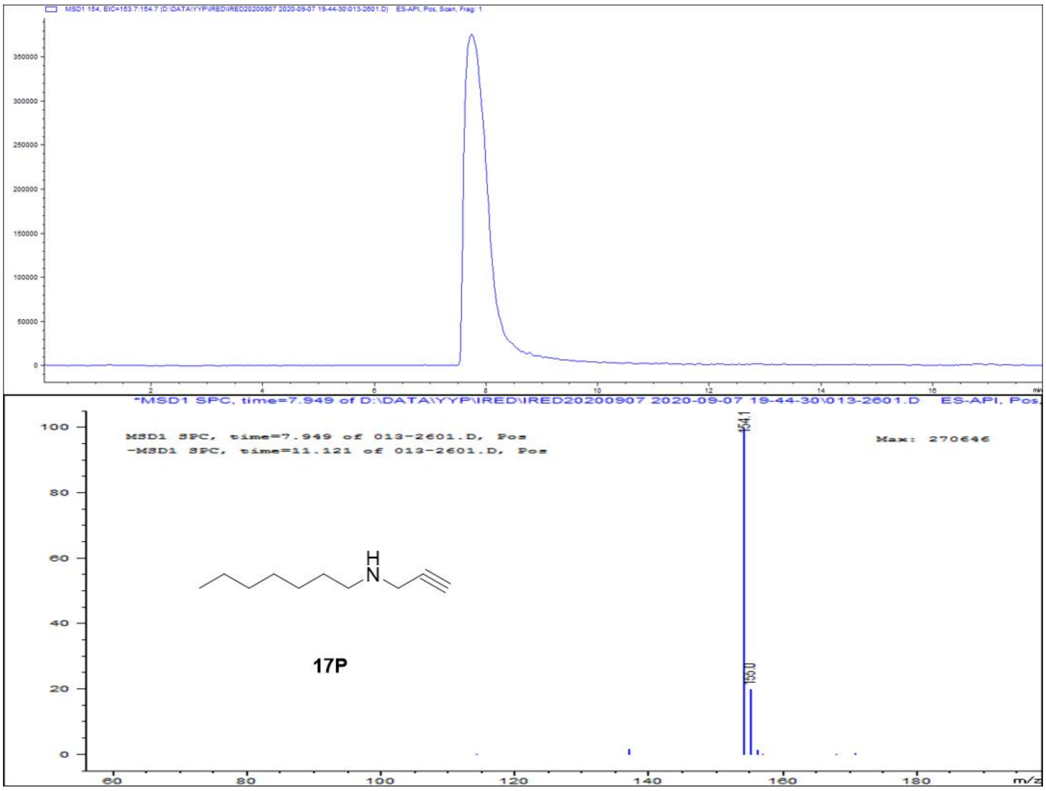

Figure 101. LCMS analysis: IR-G02-catalysed reductive amination of **17** with **P**, showing amine product **17P**.

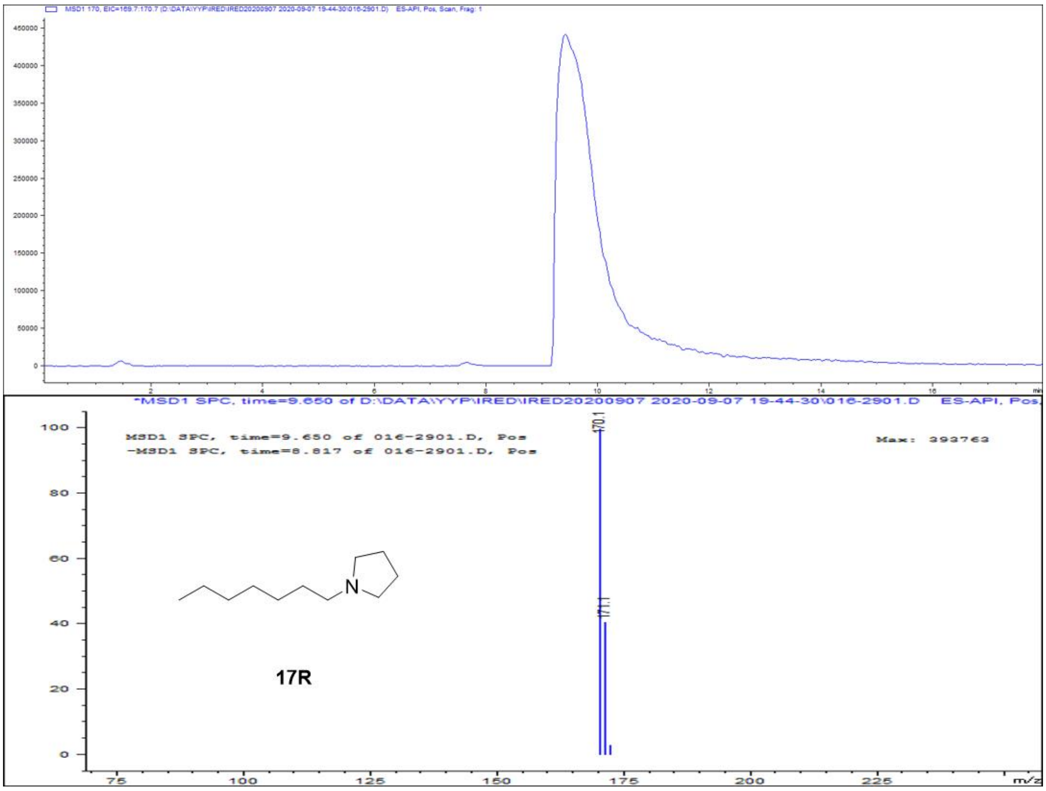

Figure 102. LCMS analysis: IR-G02-catalysed reductive amination of **17** with **R**, showing amine product **17R**.

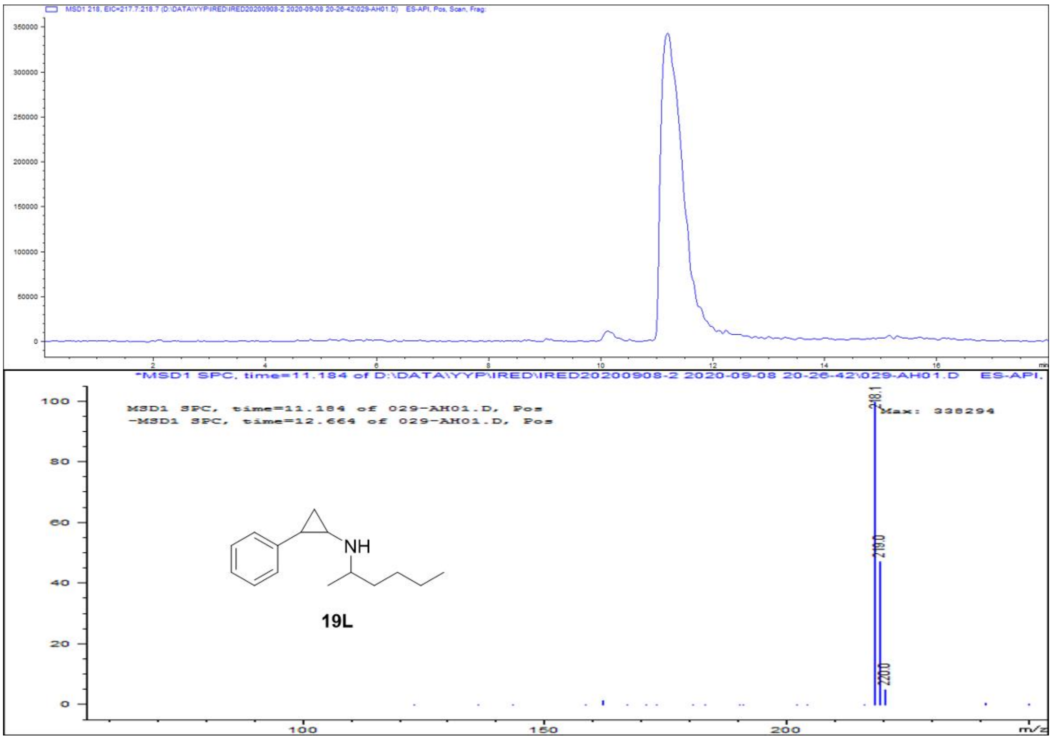

Figure 103. LCMS analysis: IR-G02-catalysed reductive amination of **19** with **L**, showing amine product **19L**.

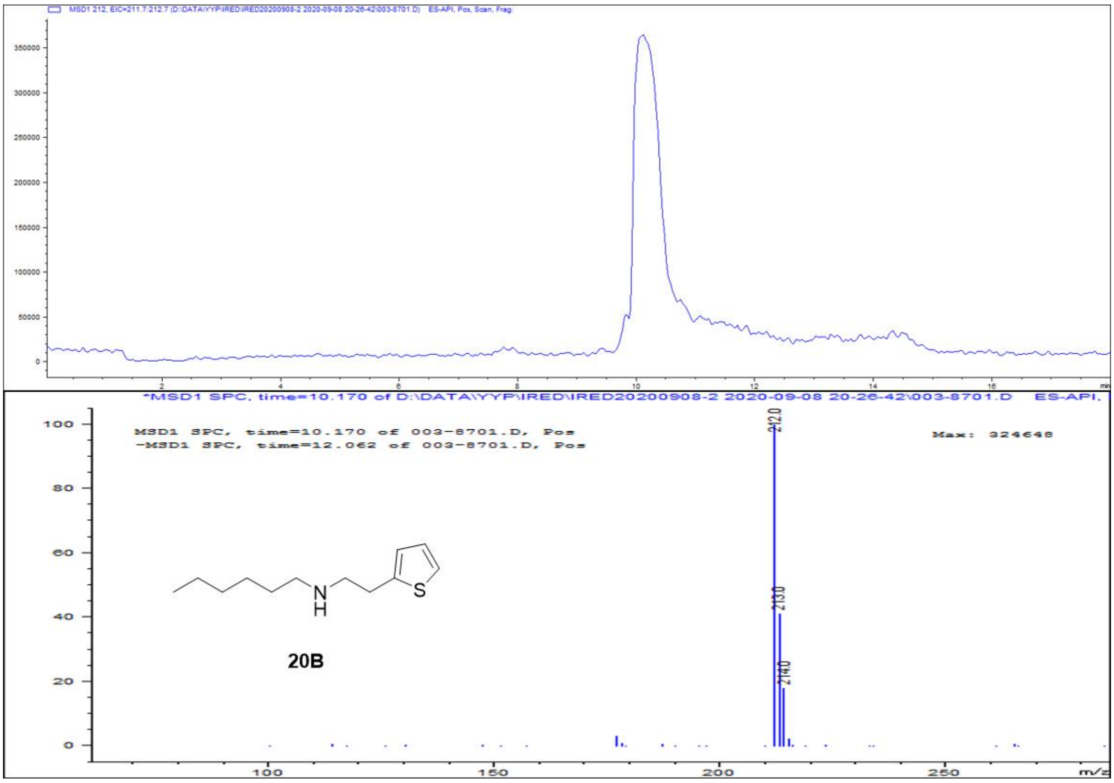

Figure 104. LCMS analysis: IR-G02-catalysed reductive amination of **20** with **B**, showing amine product **20B**.

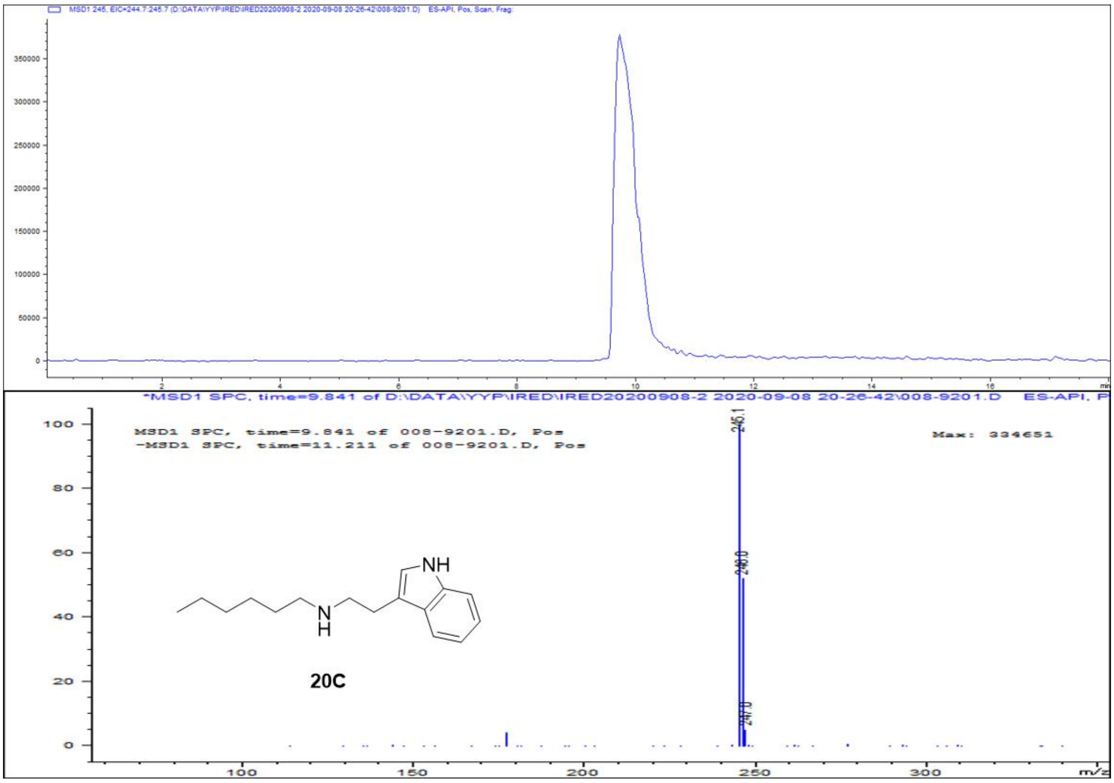

Figure 105. LCMS analysis: IR-G02-catalysed reductive amination of **20** with **C**, showing amine product **20C**.

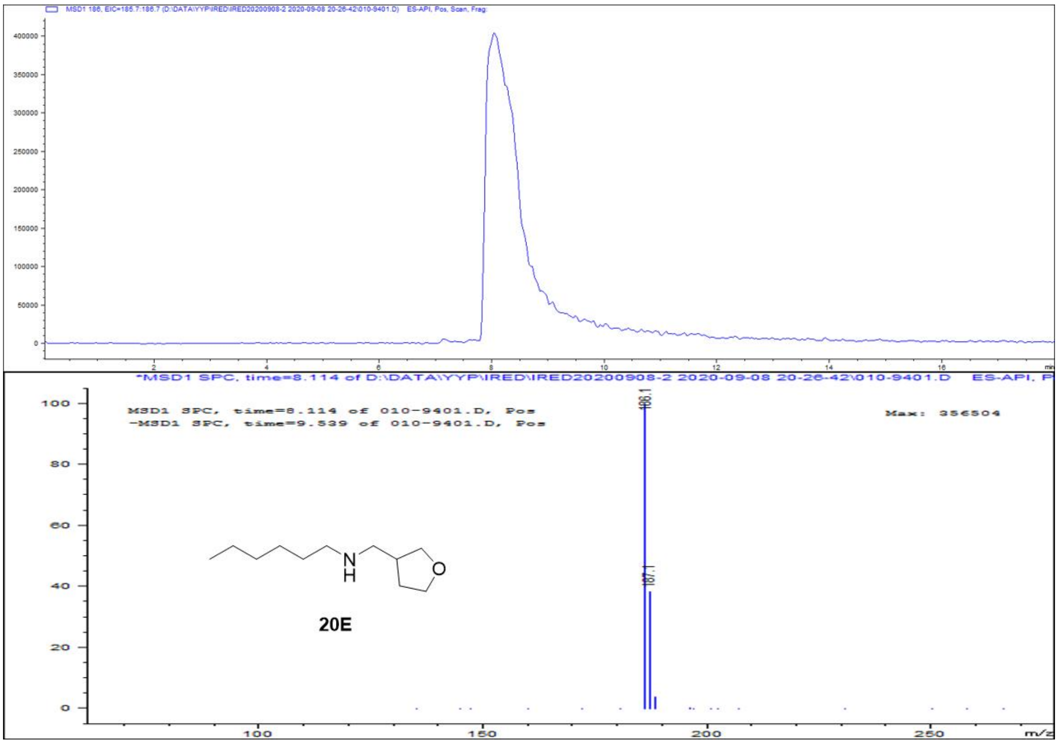

Figure 106. LCMS analysis: IR-G02-catalysed reductive amination of **20** with **E**, showing amine product **20E**.

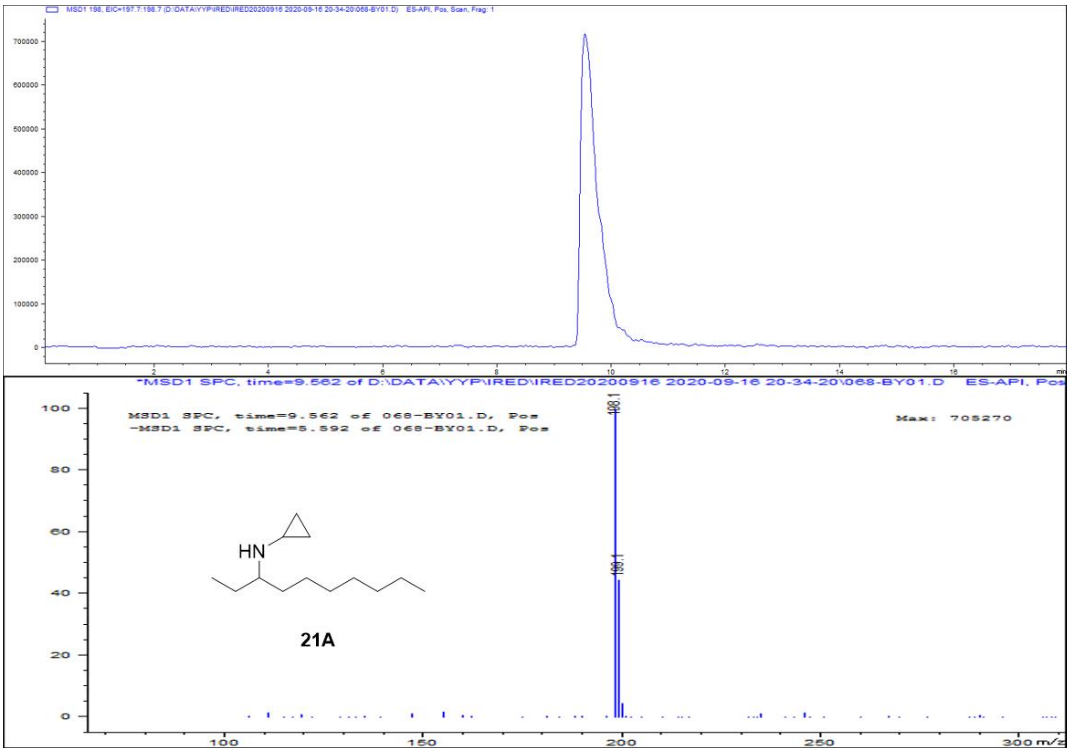

Figure 107. LCMS analysis: IR-G02-catalysed reductive amination of **21** with **A**, showing amine product **21A**.

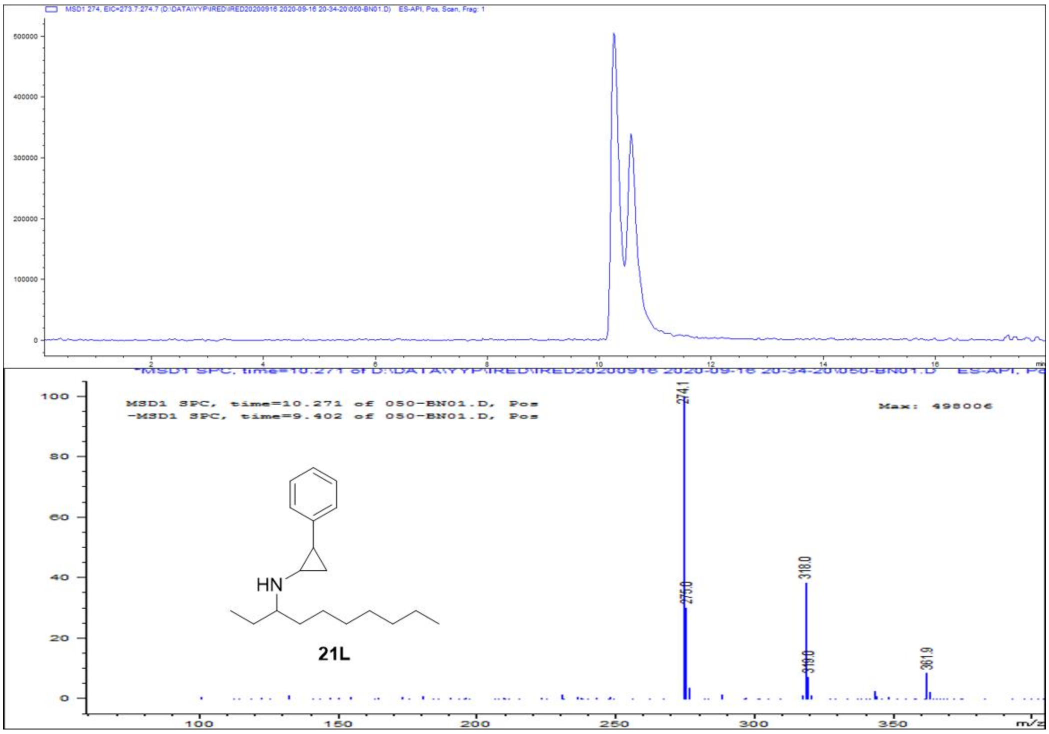

Figure 108. LCMS analysis: IR-G02-catalysed reductive amination of **21** with **L**, showing amine product **21L**.

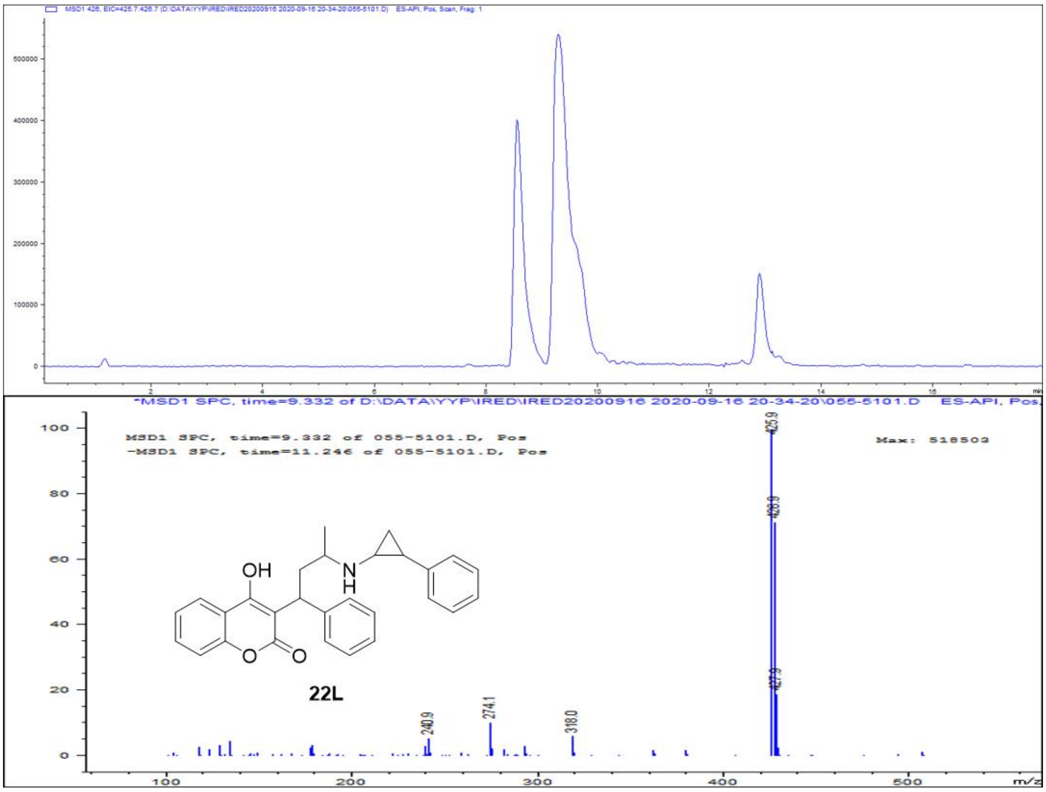

Figure 109. LCMS analysis: IR-G02-catalysed reductive amination of **22** with **L**, showing amine product **22L**.
