# Supplementary material for: Actinomycetes-derived imine reductases with a preference towards bulky amine substrates
Source: Commun Chem. 2022 Oct 8;5:123. doi: 10.1038/s42004-022-00743-y (PMC9814587; doi:10.1038/s42004-022-00743-y)
Supplement: Supplementary file 6 — Supplementary Data 4 [file 42004_2022_743_MOESM6_ESM.pdf]

The NMR spectra of all reductive amination products

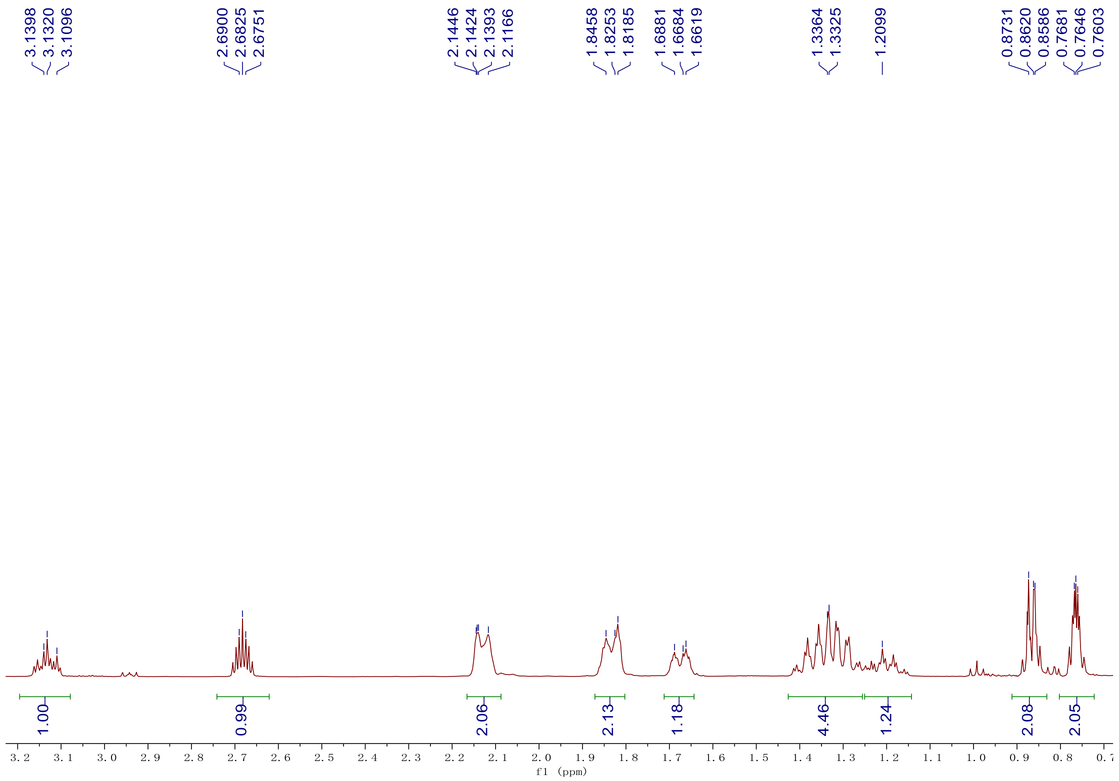

Figure 1. ^{1}H NMR (500 MHz, CD_3OD) spectrum of **1A**.

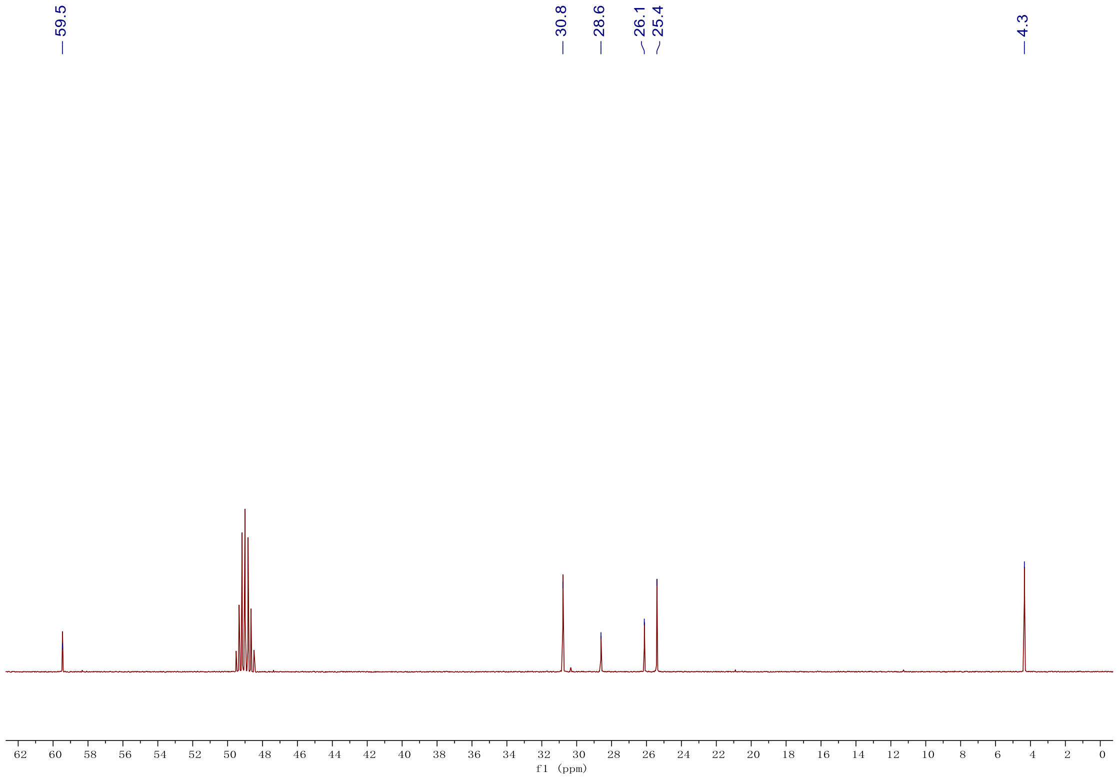

Figure 2. ^{13}C NMR (125 MHz, CD_3OD) spectrum of **1A**.

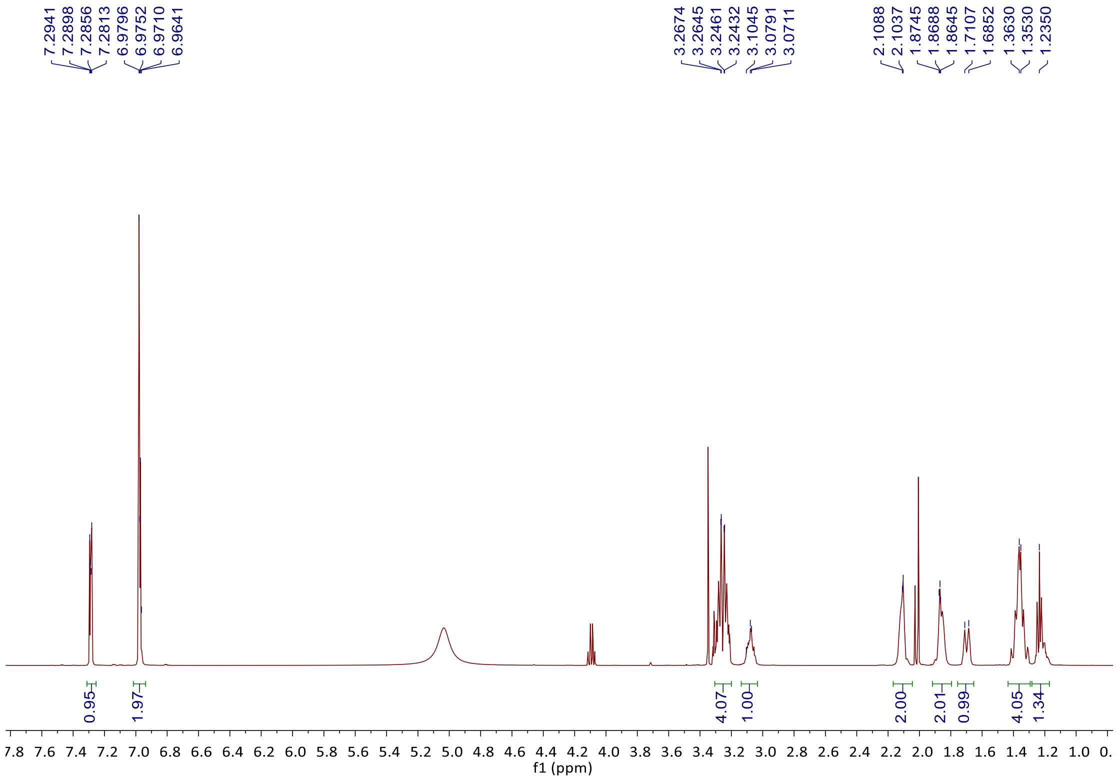

Figure 3. ^{1}H NMR (500 MHz, CD_3OD) spectrum of **1B**.

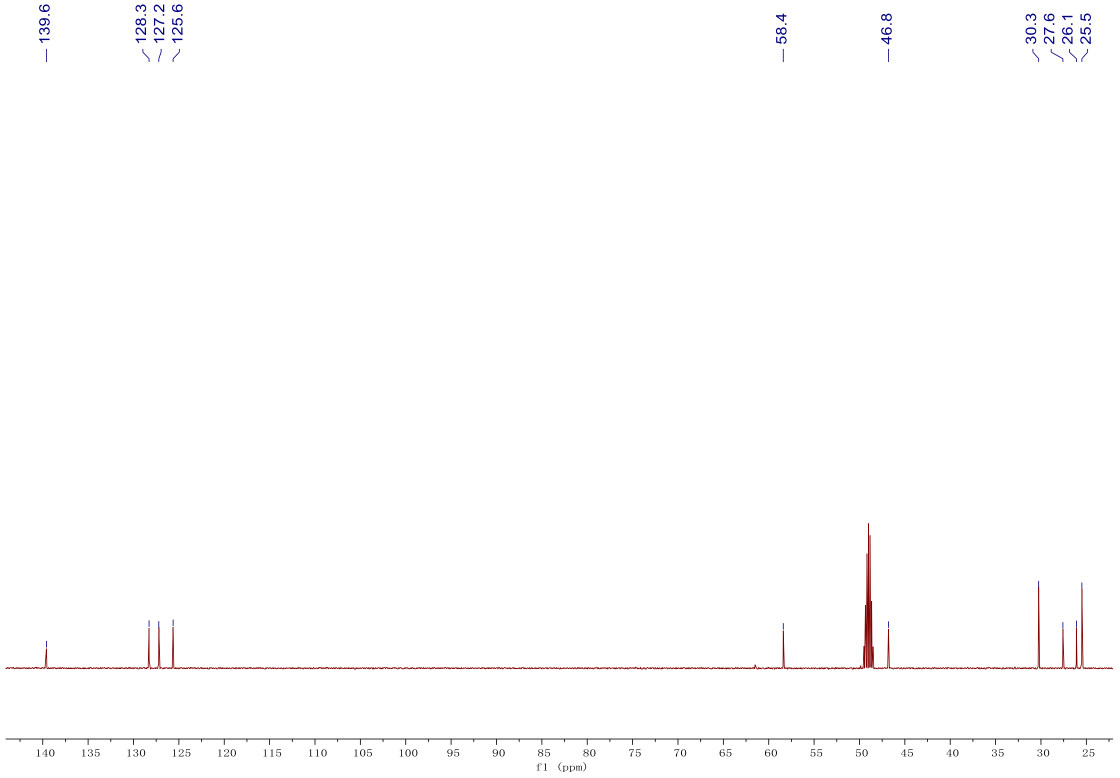

Figure 4. ^{13}C NMR (125 MHz, CD_3OD) spectrum of **1B**.

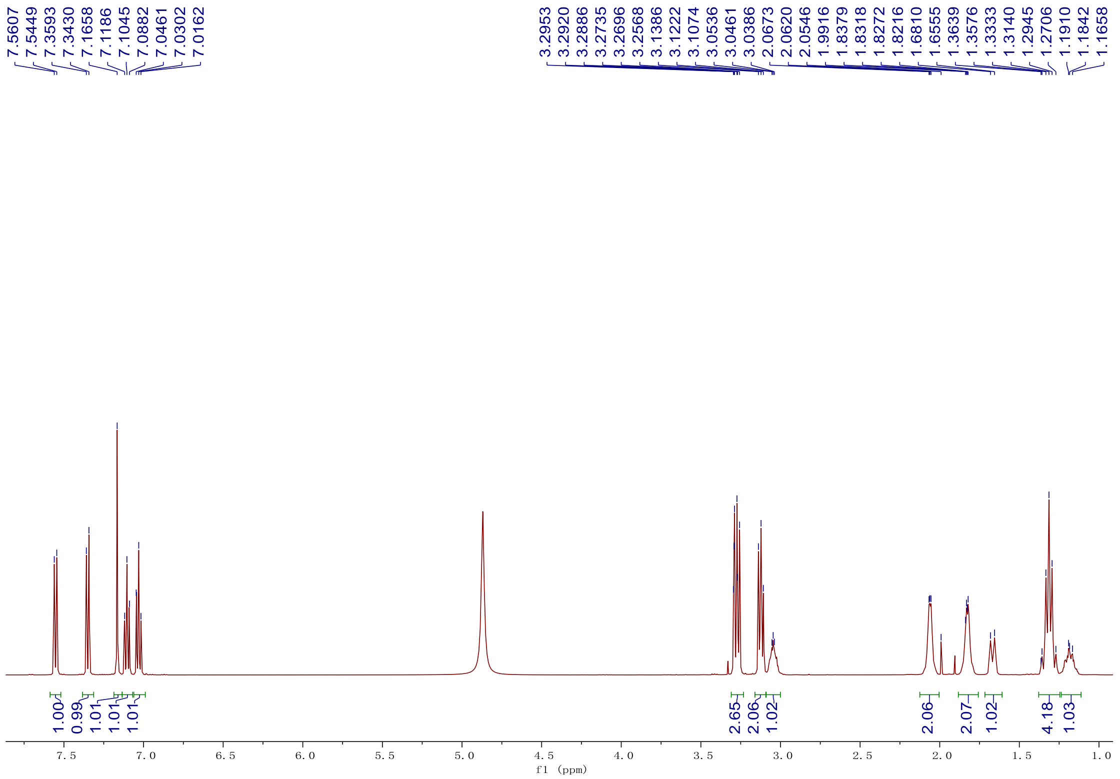

Figure 5. ^{1}H NMR (500 MHz, CD_3OD) spectrum of **1C**.

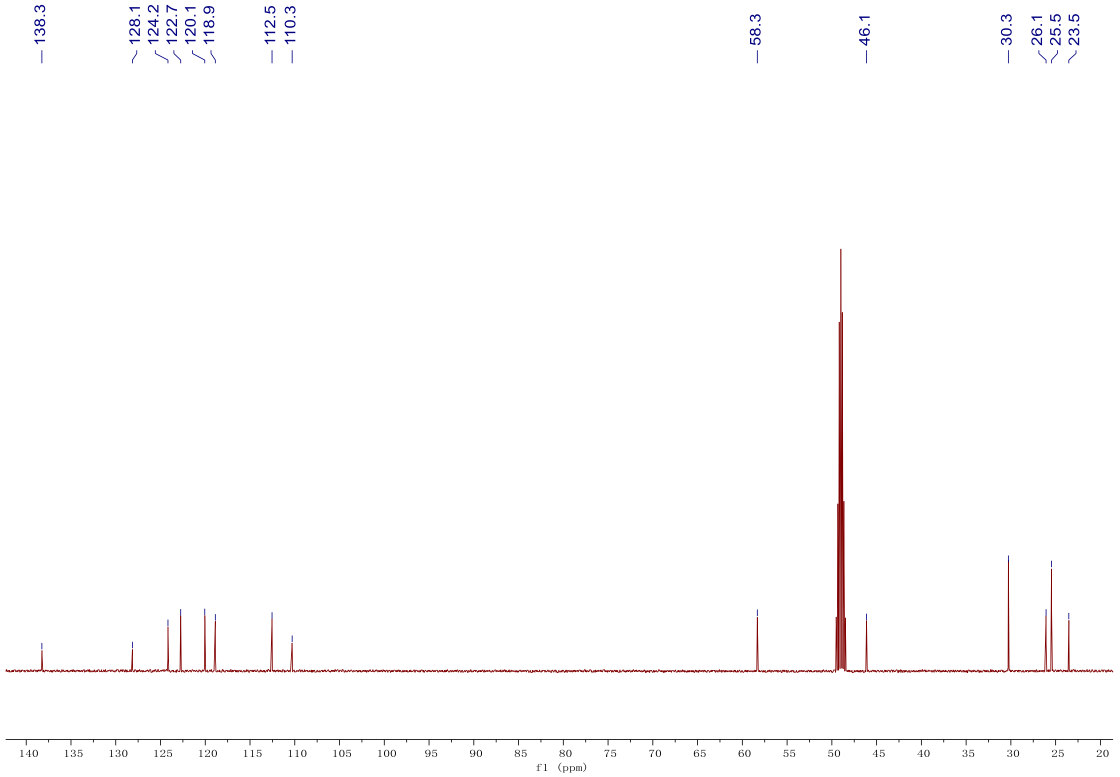

Figure 6. ^{13}C NMR (125 MHz, CD_3OD) spectrum of **1C**.

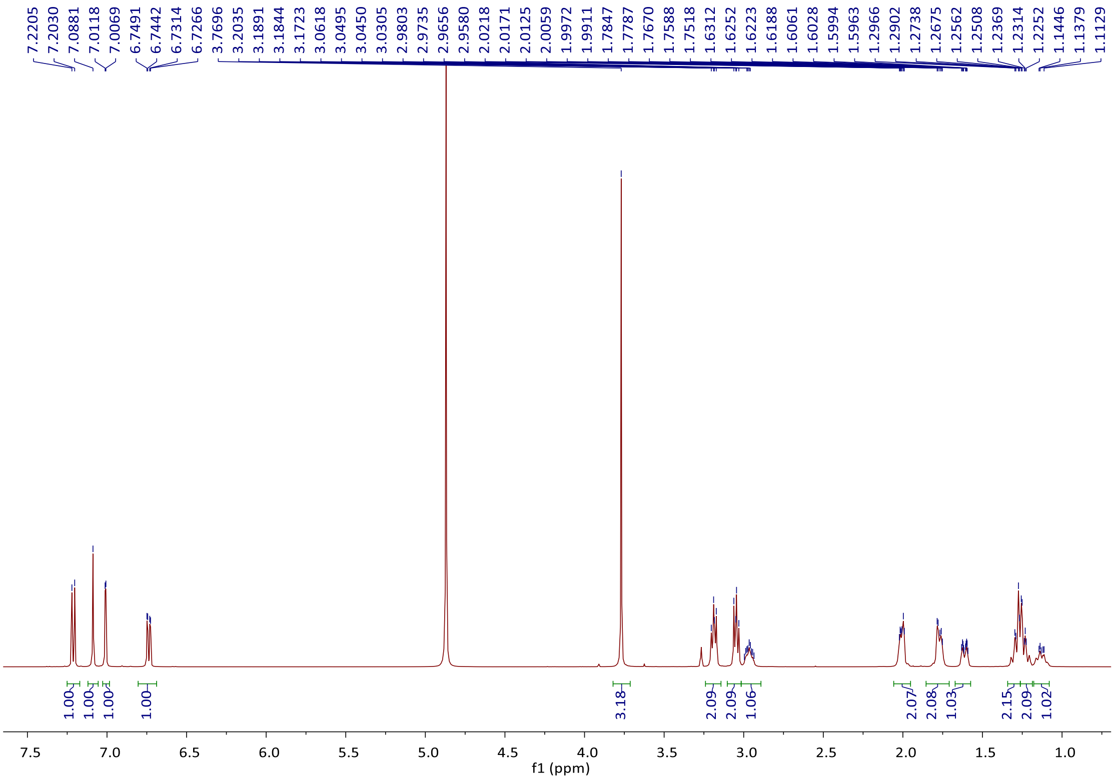

Figure 7. ^{1}H NMR (500 MHz, CD_3OD) spectrum of **1G**.

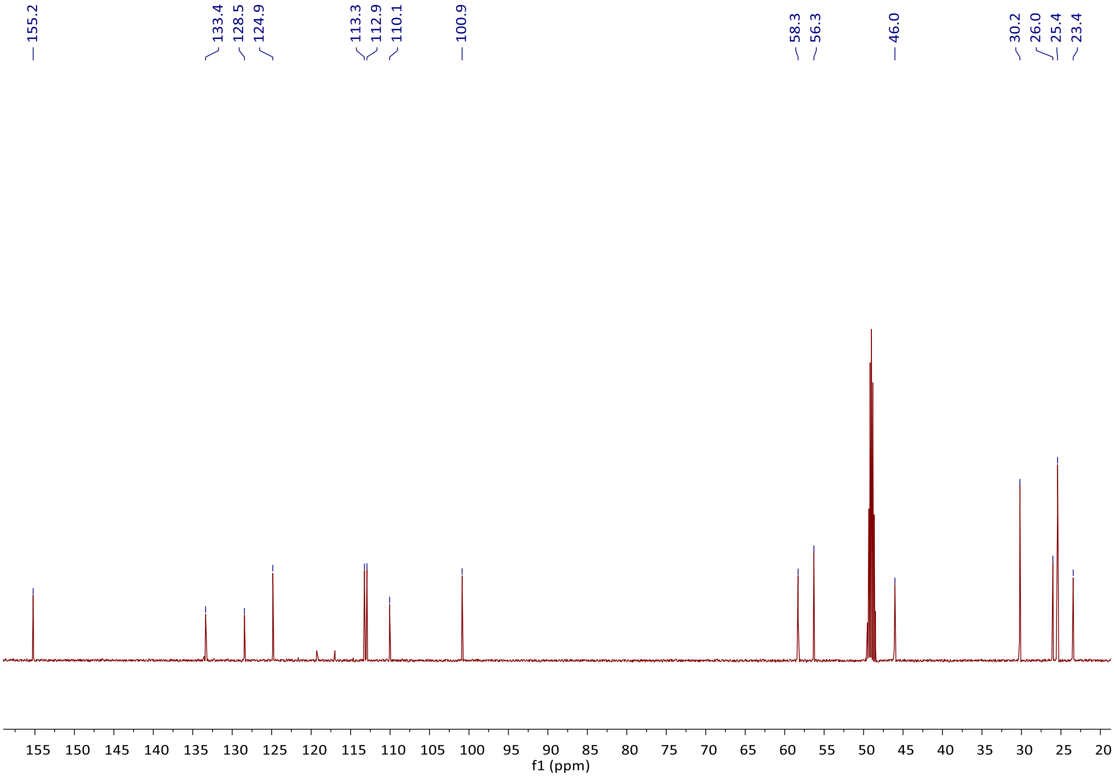

Figure 8. ^{13}C NMR (125 MHz, CD_3OD) spectrum of **1G**.

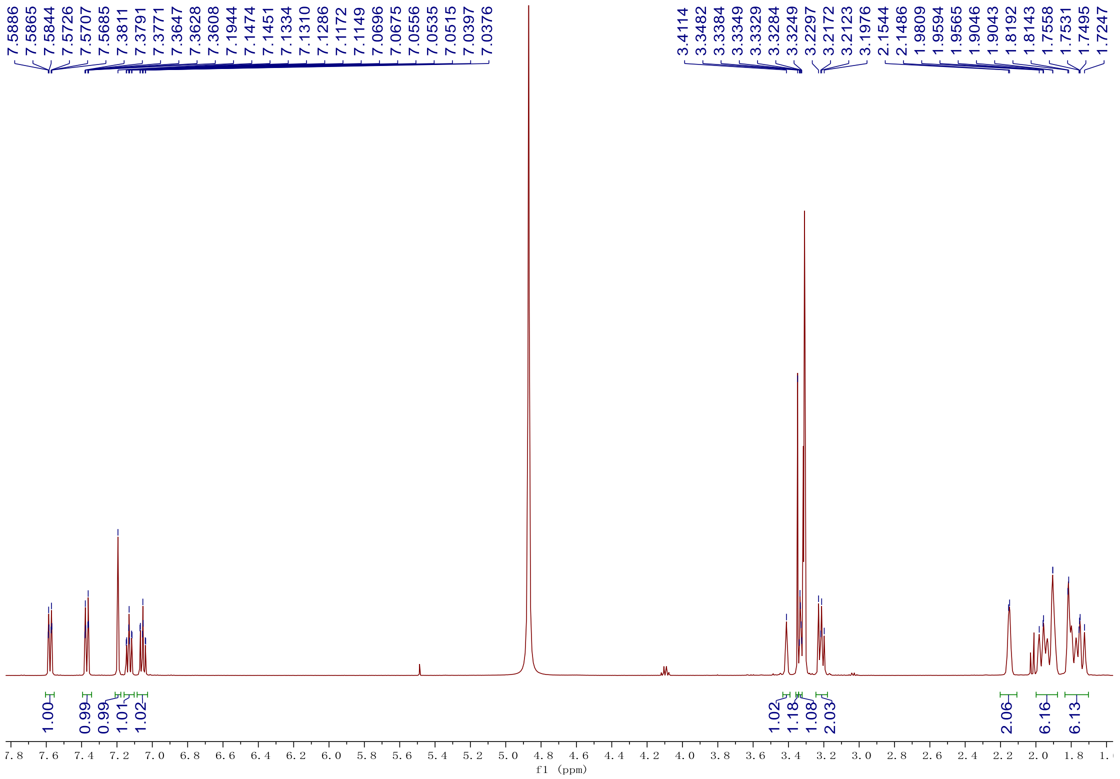

Figure 9. ^{1}H NMR (500 MHz, CD_3OD) spectrum of **2C**.

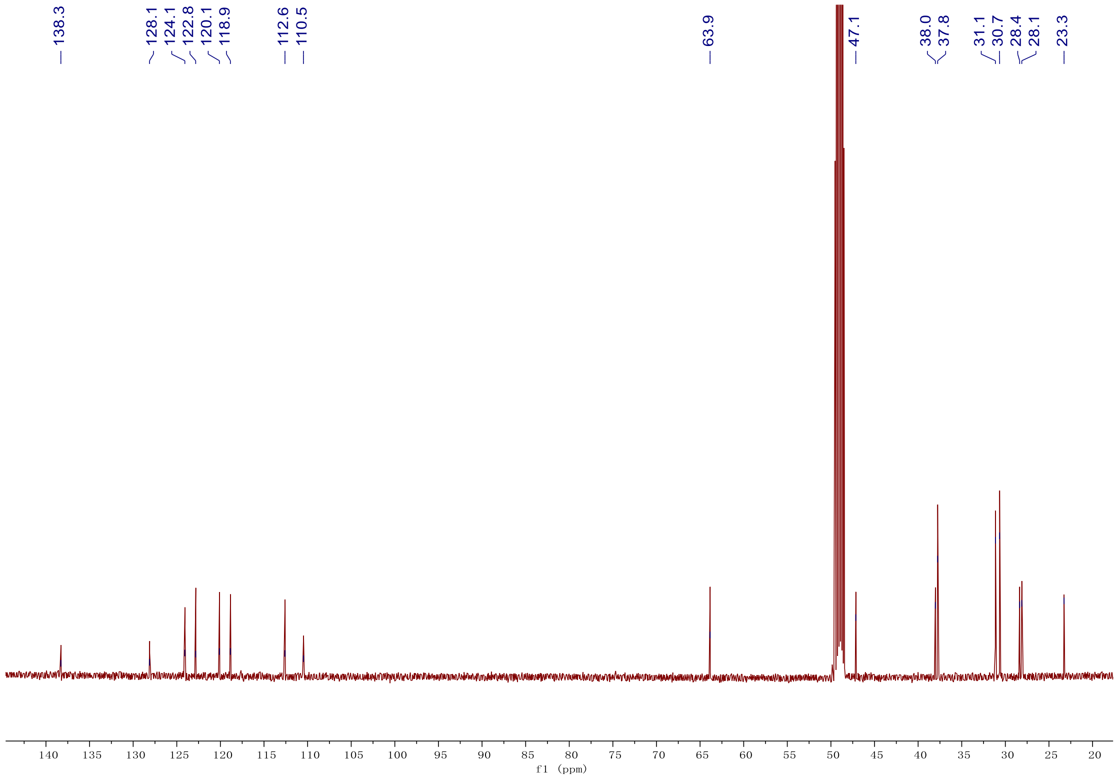

Figure 10. ^{13}C NMR (125 MHz, CD_3OD) spectrum of **2C**.

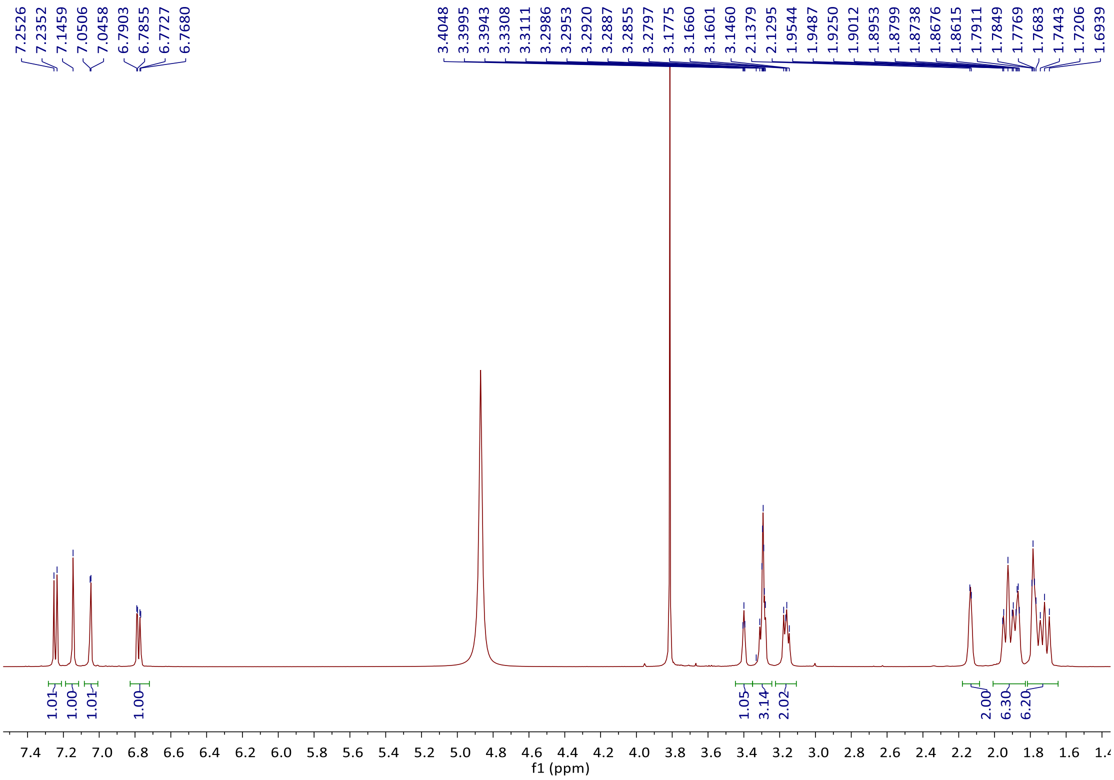

Figure 11. ^{1}H NMR (500 MHz, CD_3OD) spectrum of **2D**.

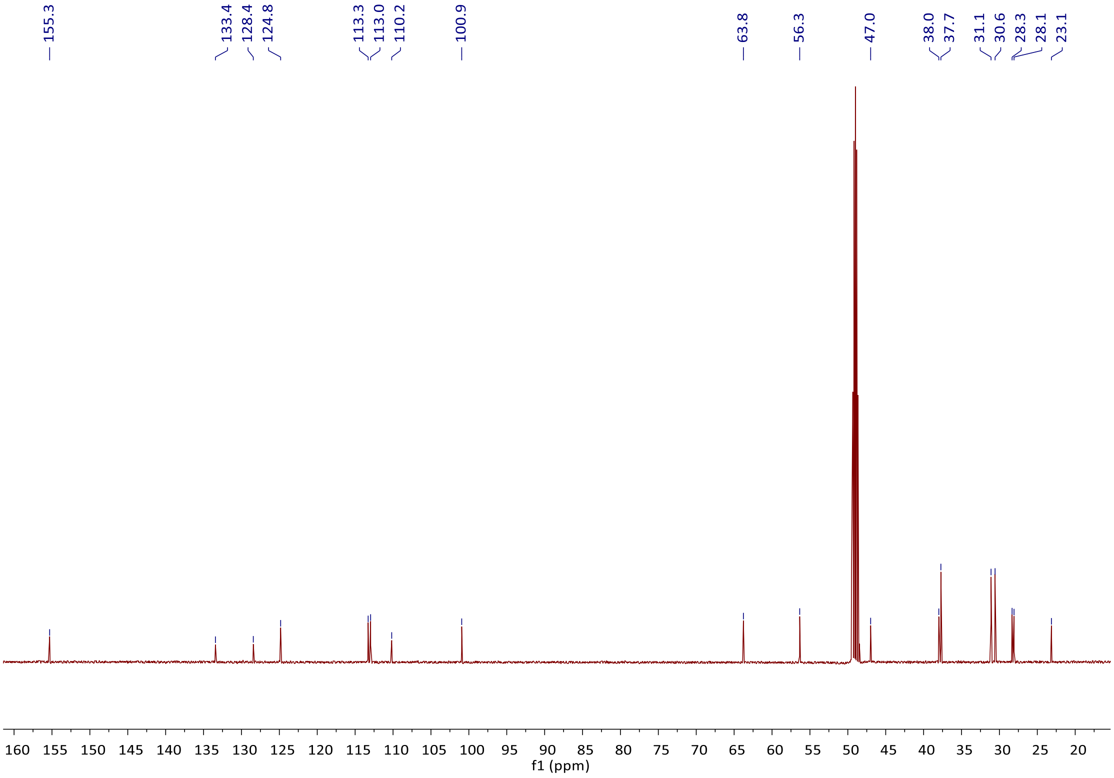

Figure 12. ^{13}C NMR (125 MHz, CD_3OD) spectrum of **2D**.

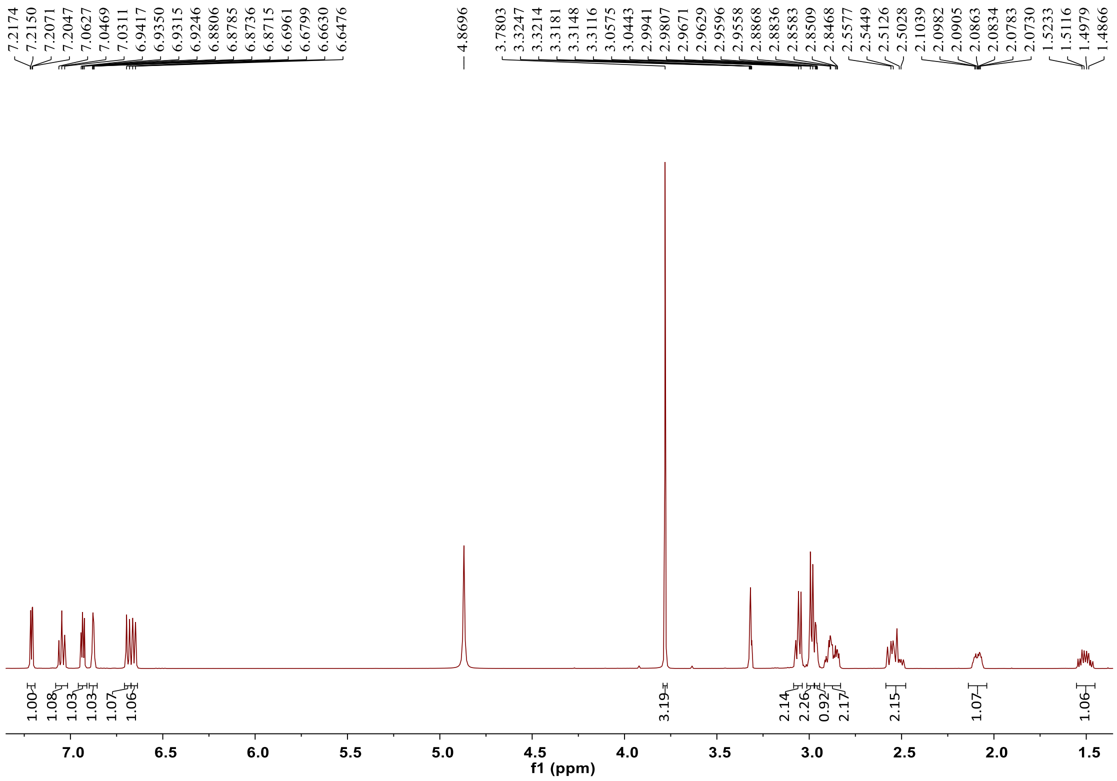

Figure 13. ^{1}H NMR (500 MHz, CD_3OD) spectrum of **3B**.

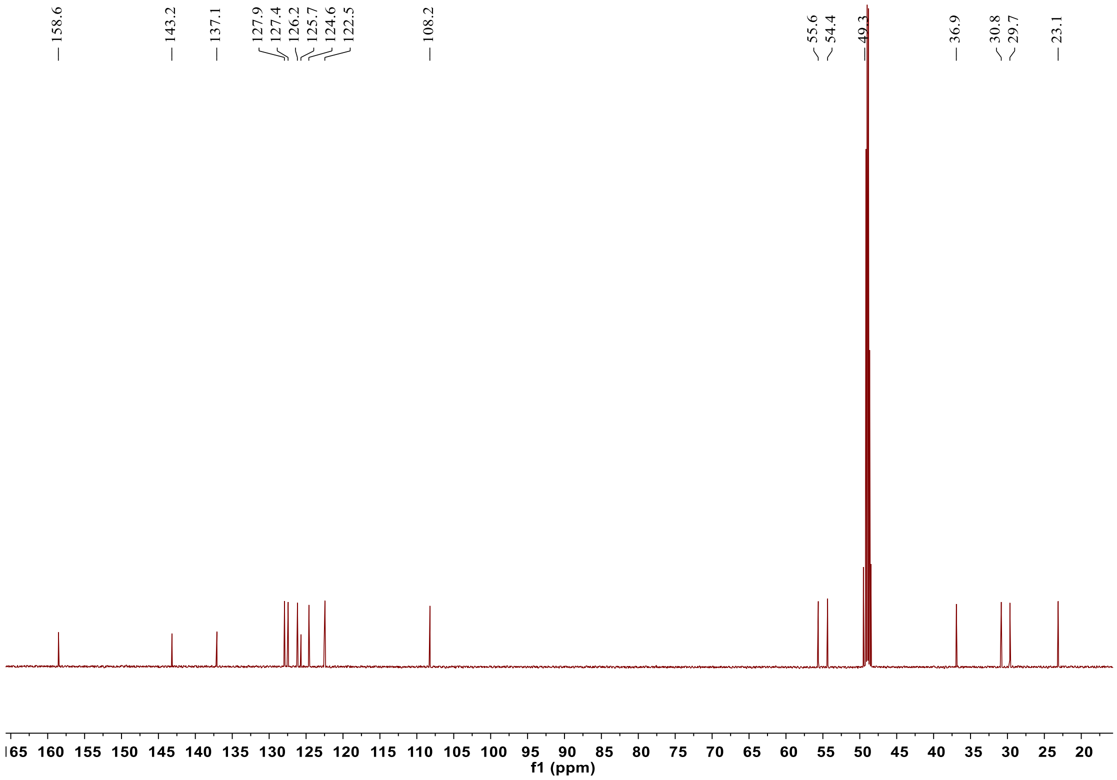

Figure 14. ^{13}C NMR (125 MHz, CD_3OD) spectrum of **3B**.

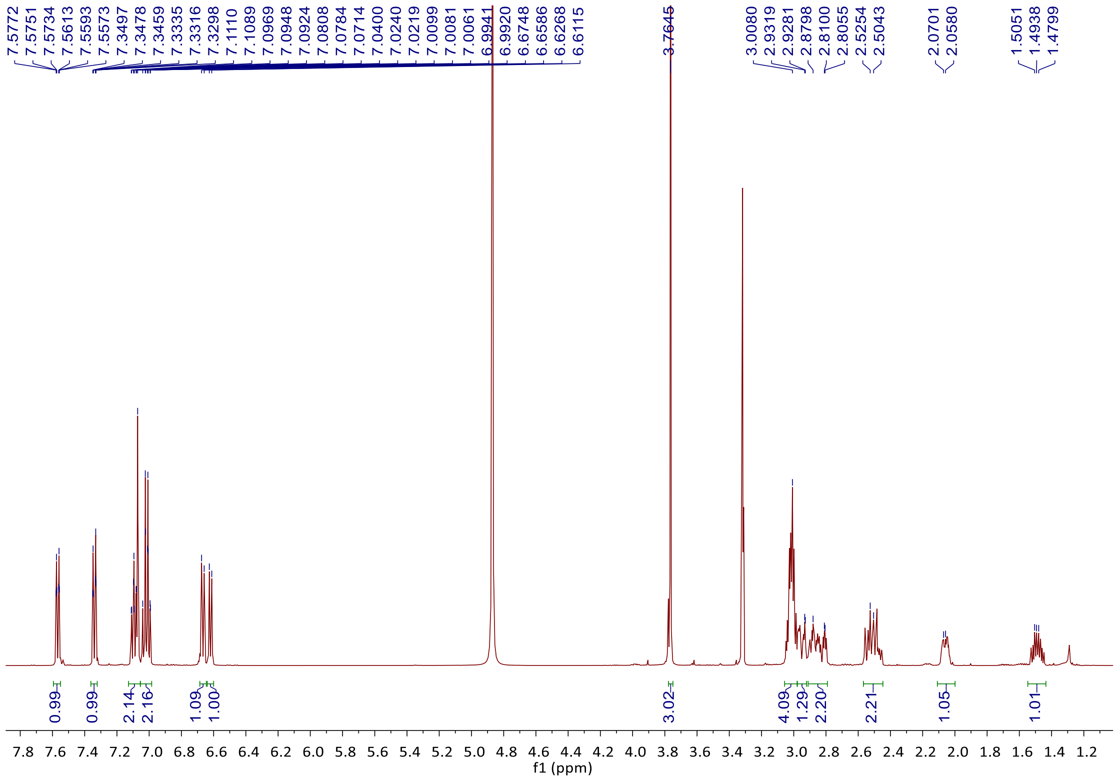

Figure 15. ^{1}H NMR (500 MHz, CD_3OD) spectrum of **3C**.

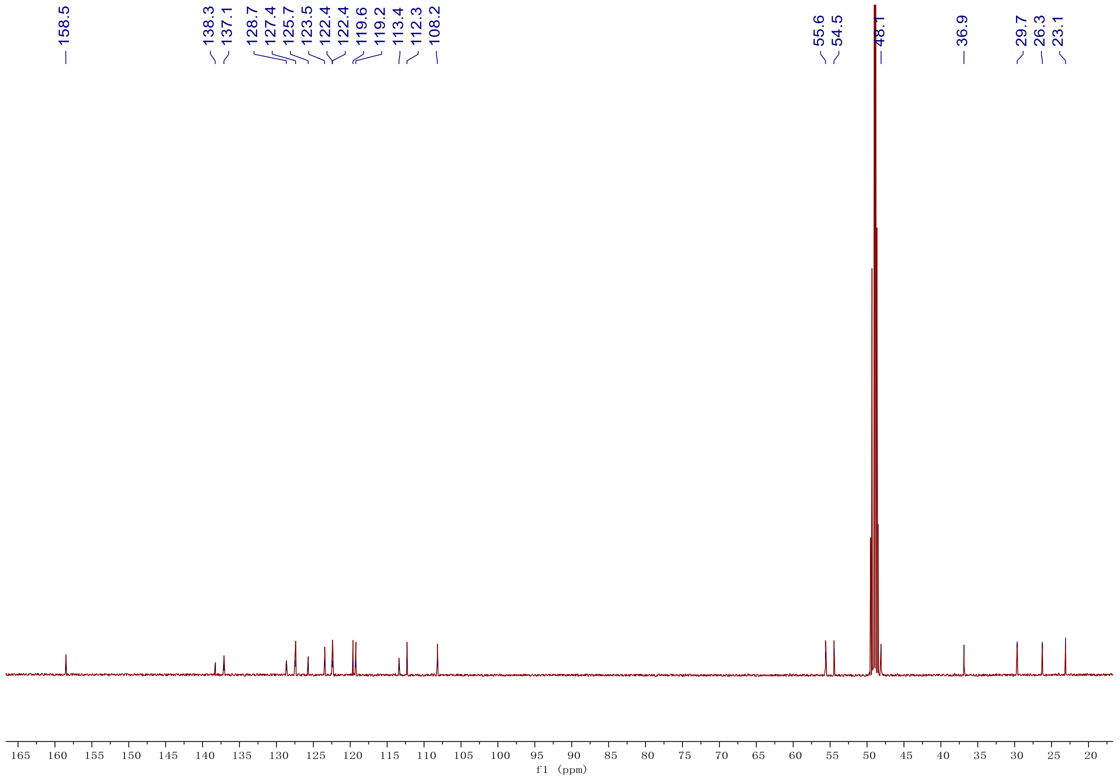

Figure 16. ^{13}C NMR (125 MHz, CD_3OD) spectrum of **3C**.

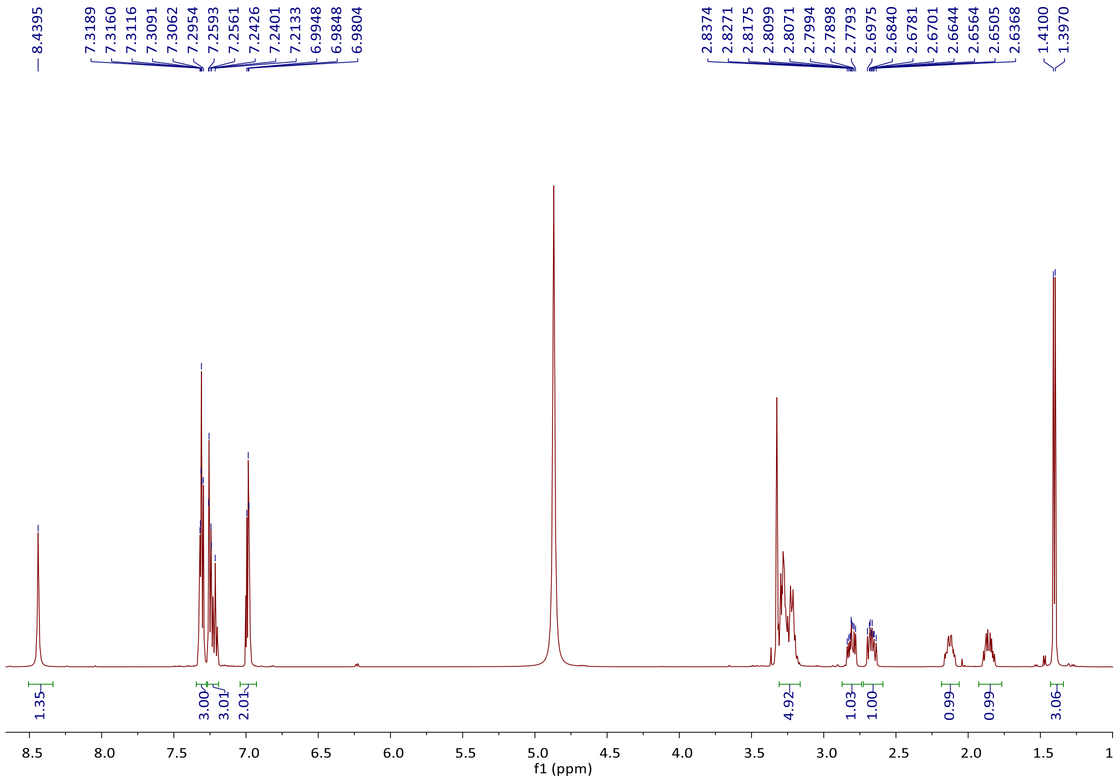

Figure 17. ^{1}H NMR (500 MHz, CD_3OD) spectrum of **4B**.

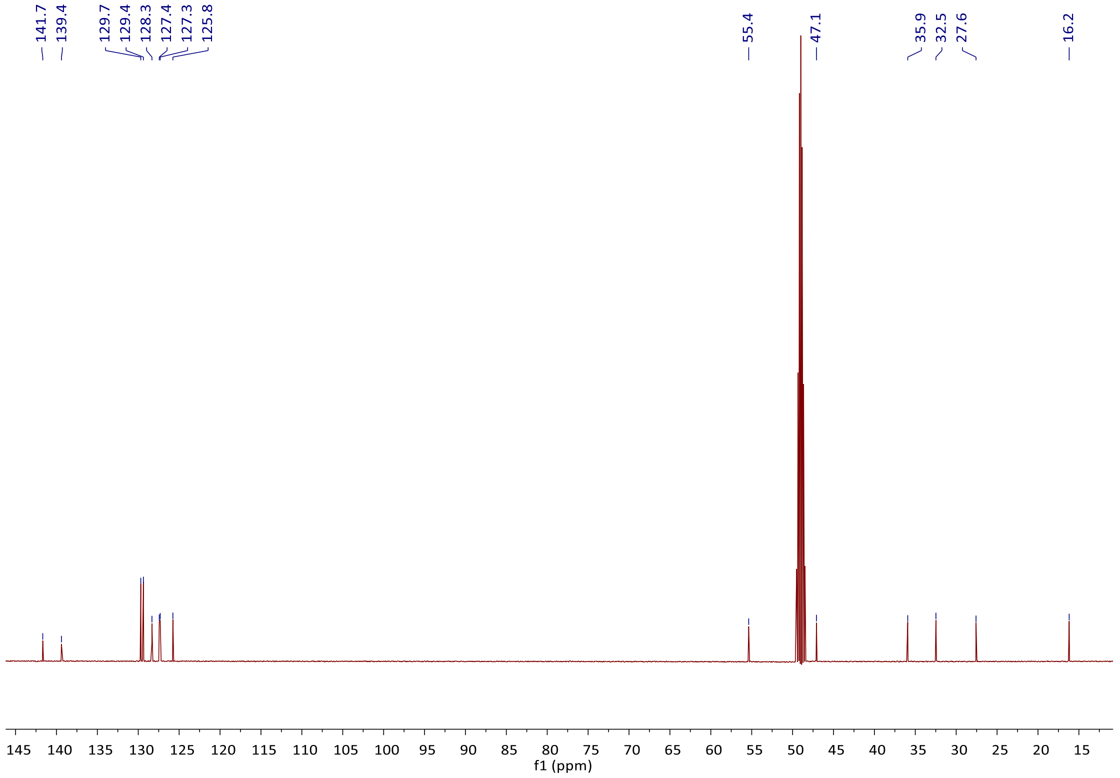

Figure 18. ^{13}C NMR (125 MHz, CD_3OD) spectrum of **4B**.

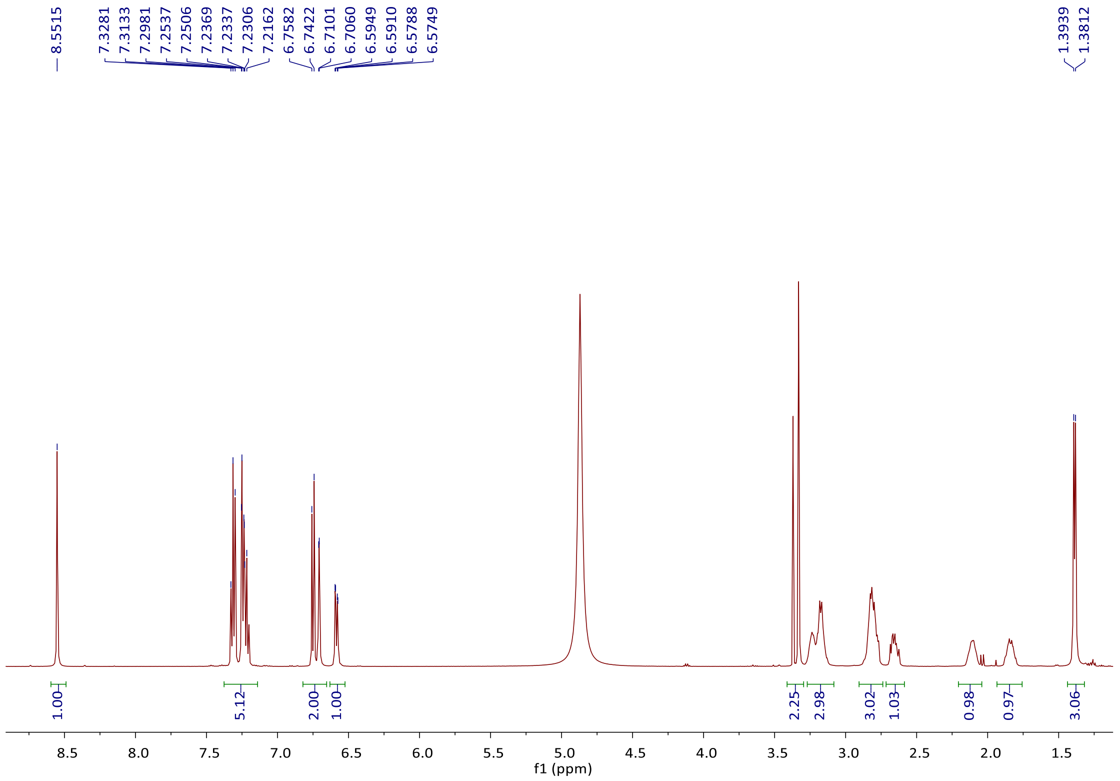

Figure 19. ^{1}H NMR (500 MHz, CD_3OD) spectrum of **4D**.

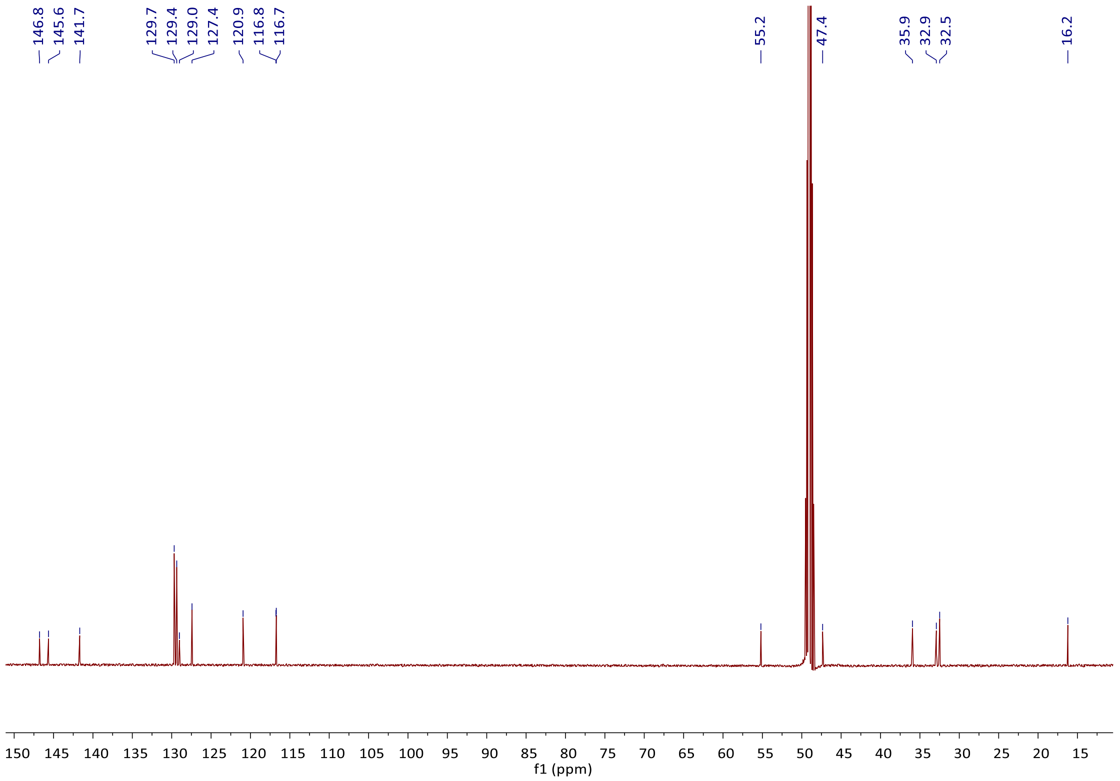

Figure 20. ^{13}C NMR (125 MHz, CD_3OD) spectrum of **4D**.

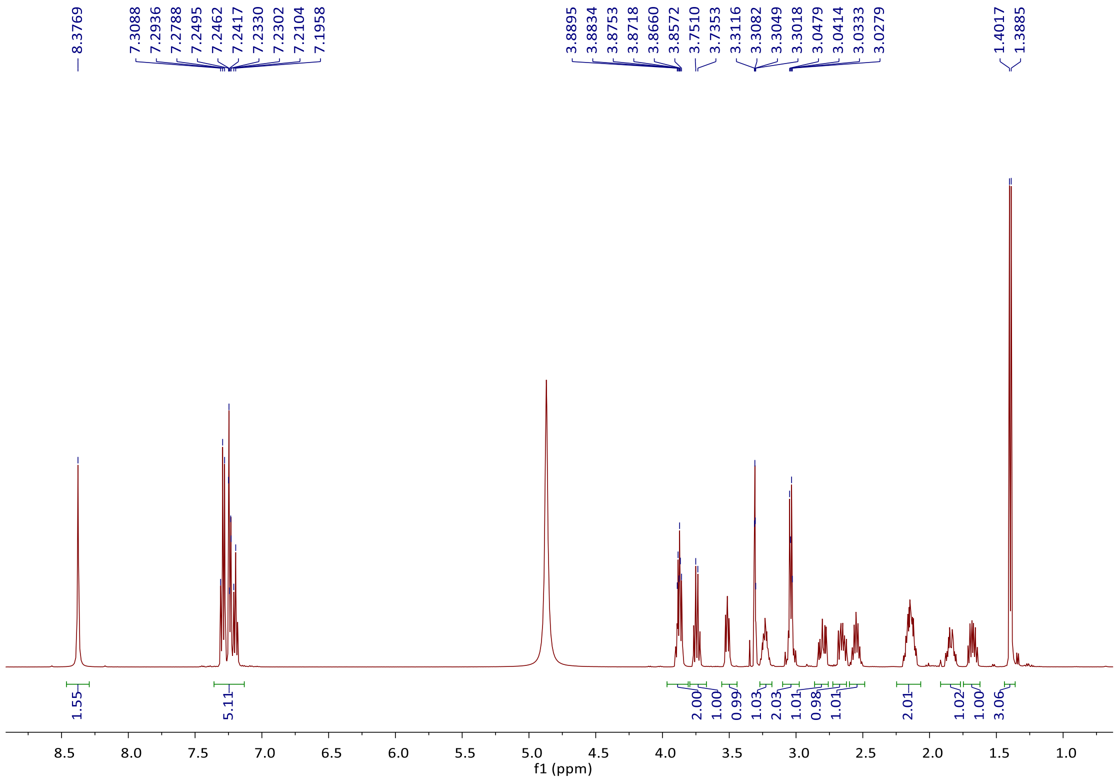

Figure 21. ^{1}H NMR (500 MHz, CD_3OD) spectrum of **4E**.

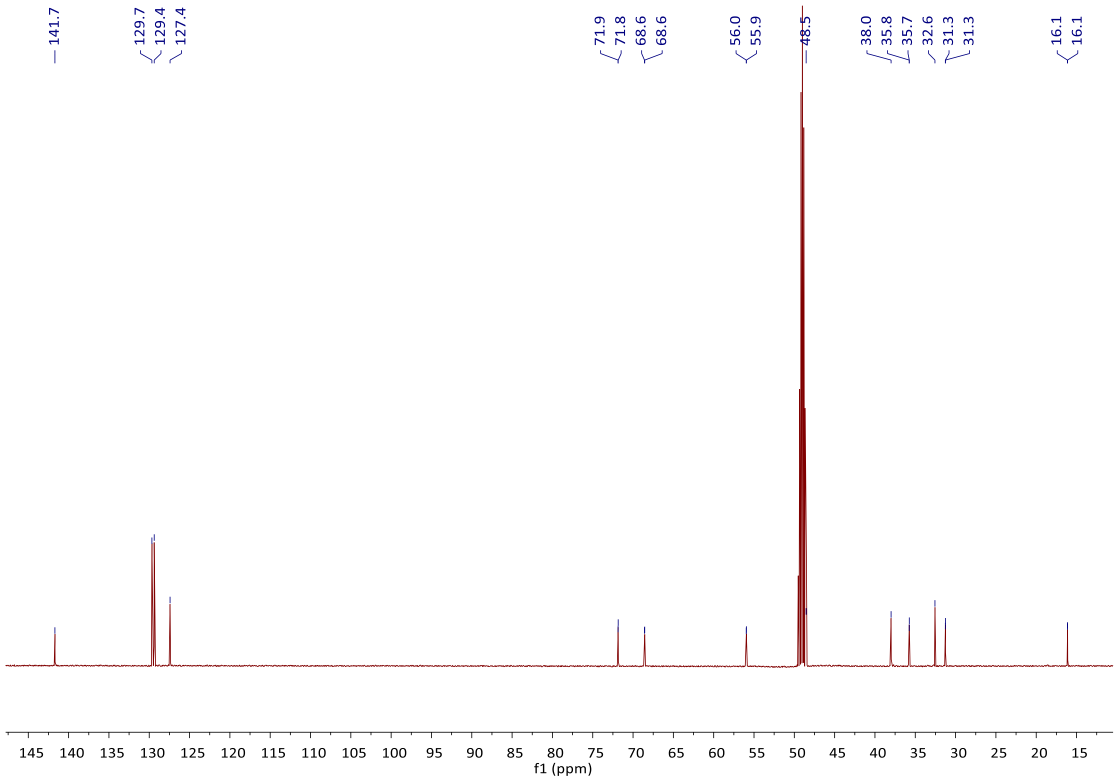

Figure 22. ^{13}C NMR (125 MHz, CD_3OD) spectrum of **4E**.

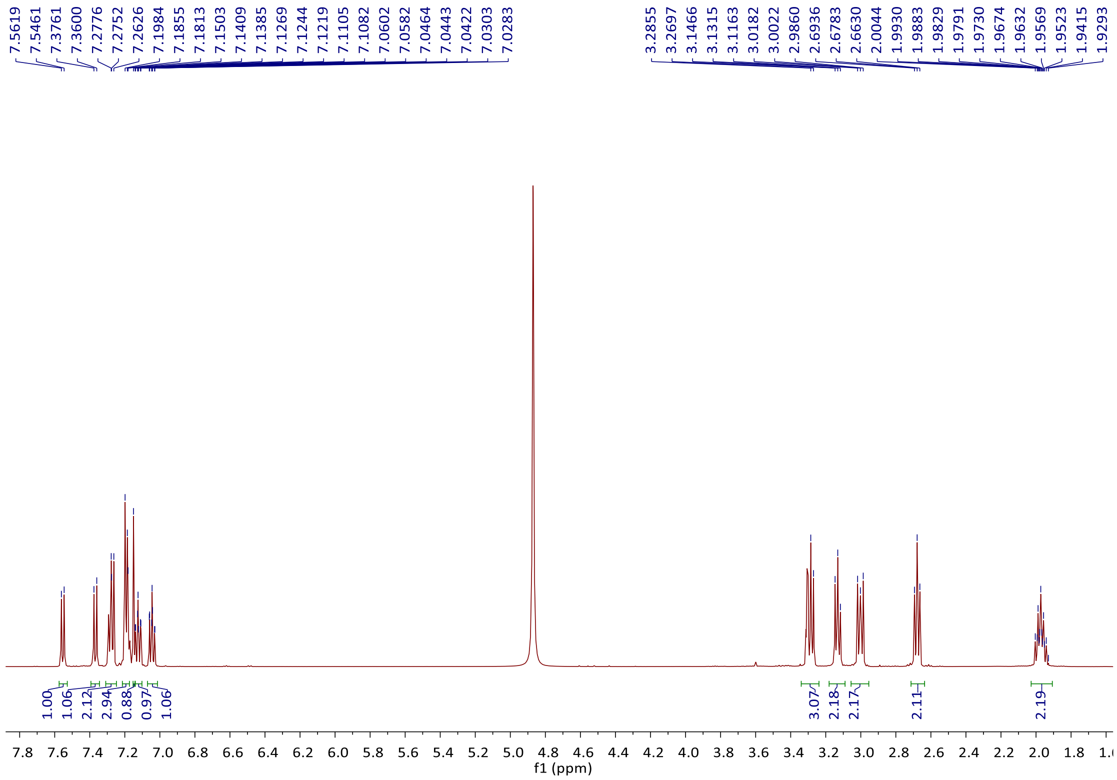

Figure 23. ^{1}H NMR (500 MHz, CD_3OD) spectrum of **5C**.

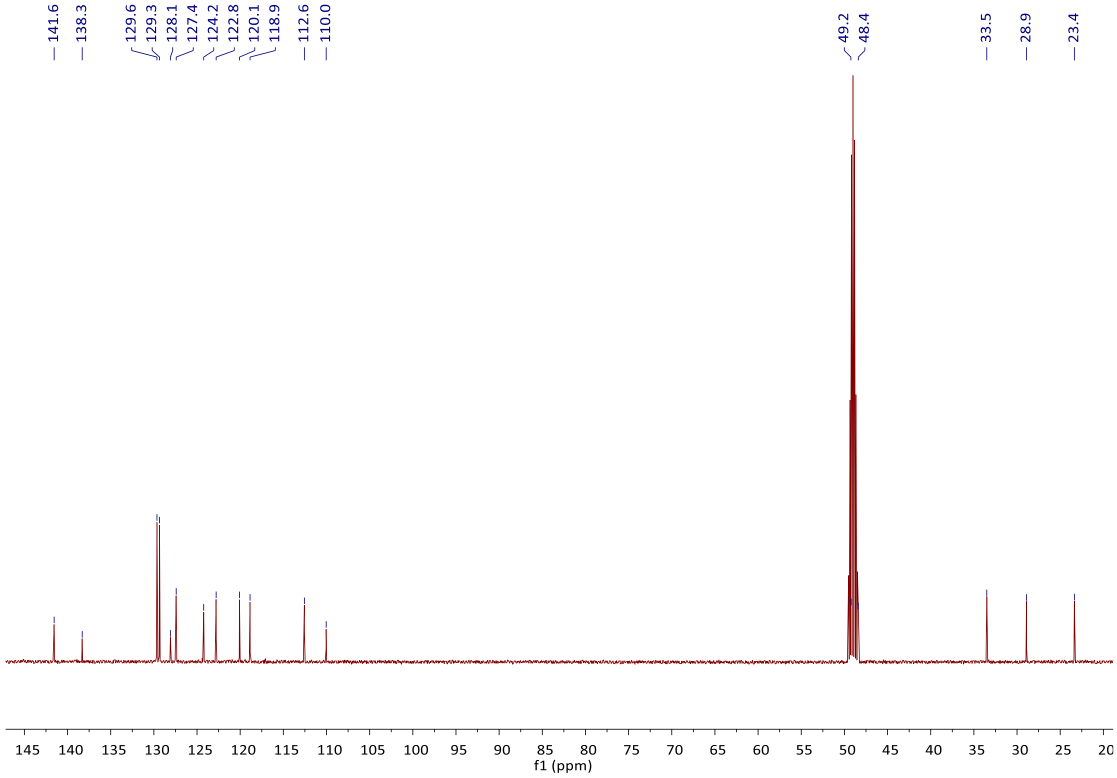

Figure 24. ^{13}C NMR (125 MHz, CD_3OD) spectrum of **5C**.

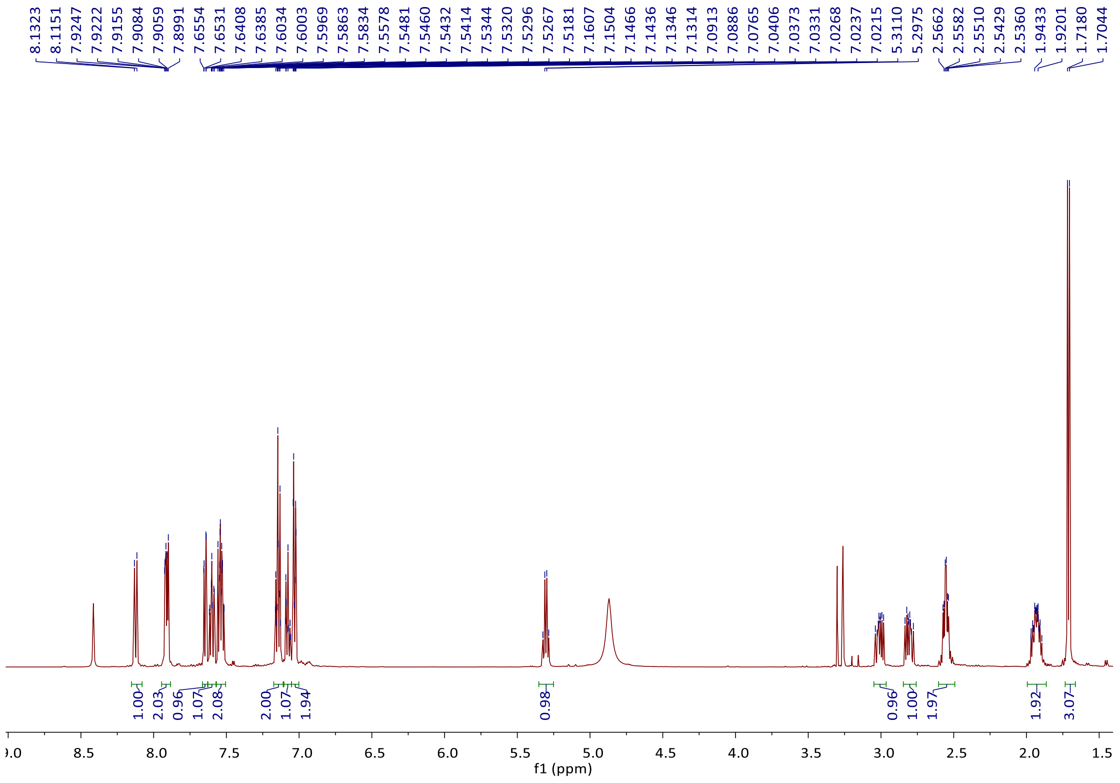

Figure 25. ^{1}H NMR (500 MHz, CD_3OD) spectrum of **5F**.

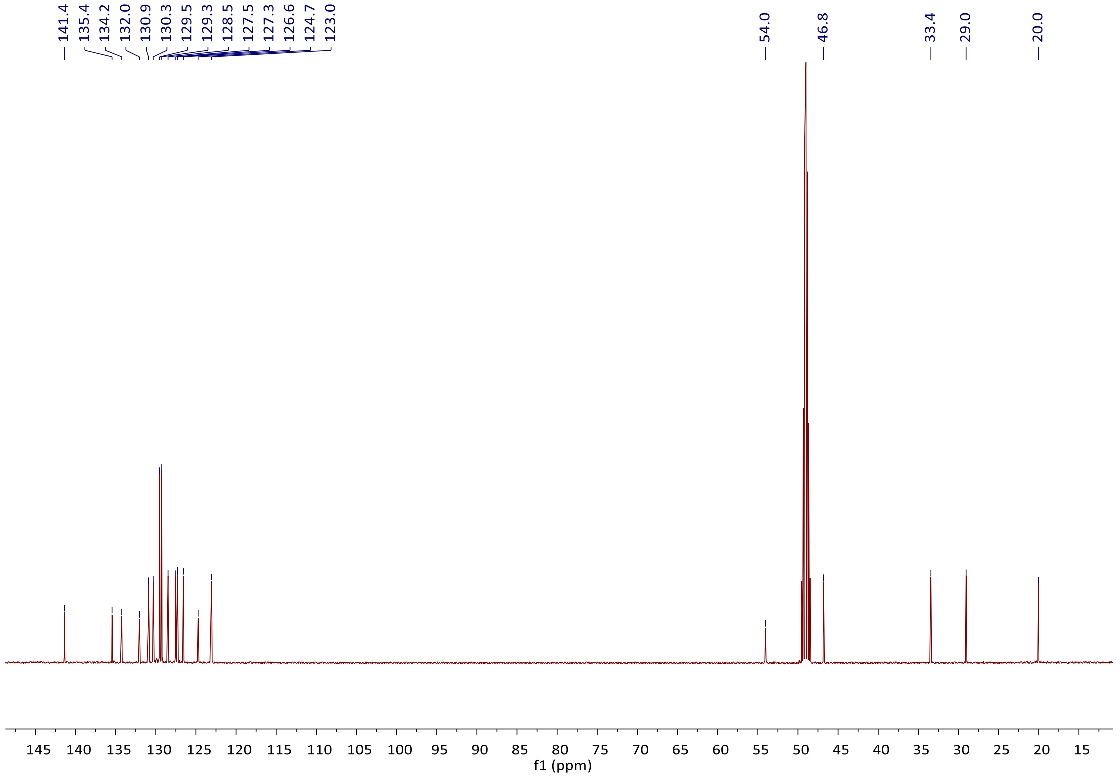

Figure 26. ^{13}C NMR (125 MHz, CD_3OD) spectrum of **5F**.

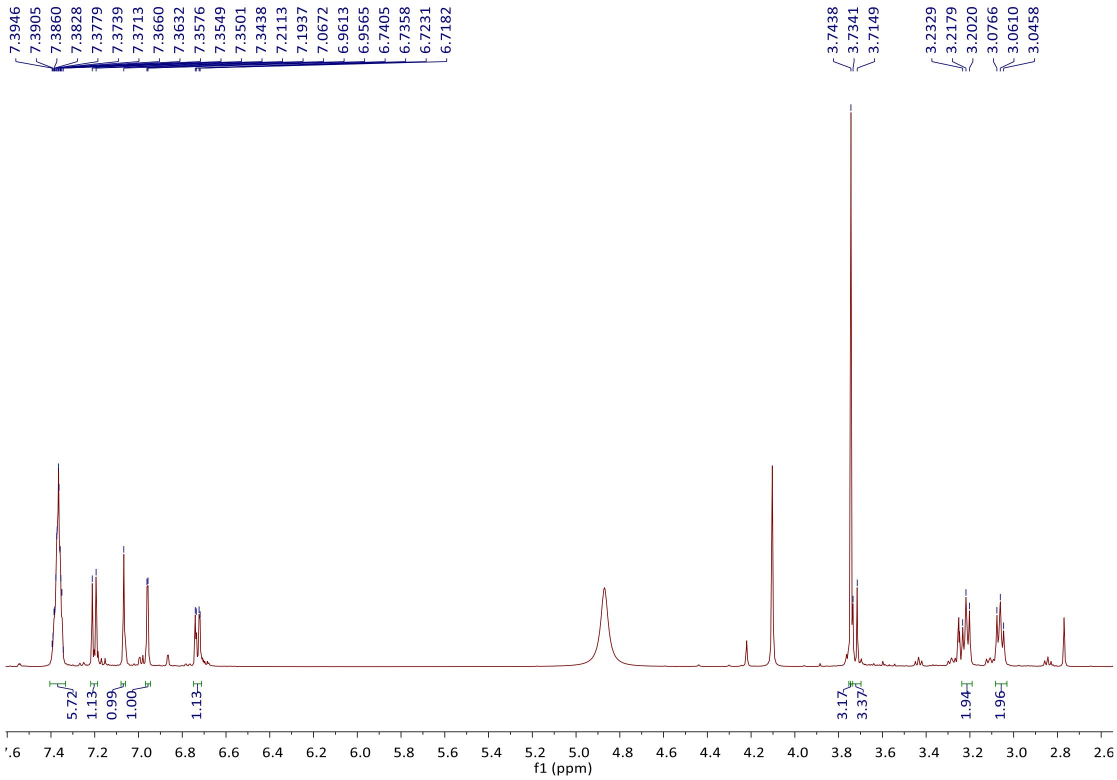

Figure 27. ^{1}H NMR (500 MHz, CD_3OD) spectrum of **6G**.

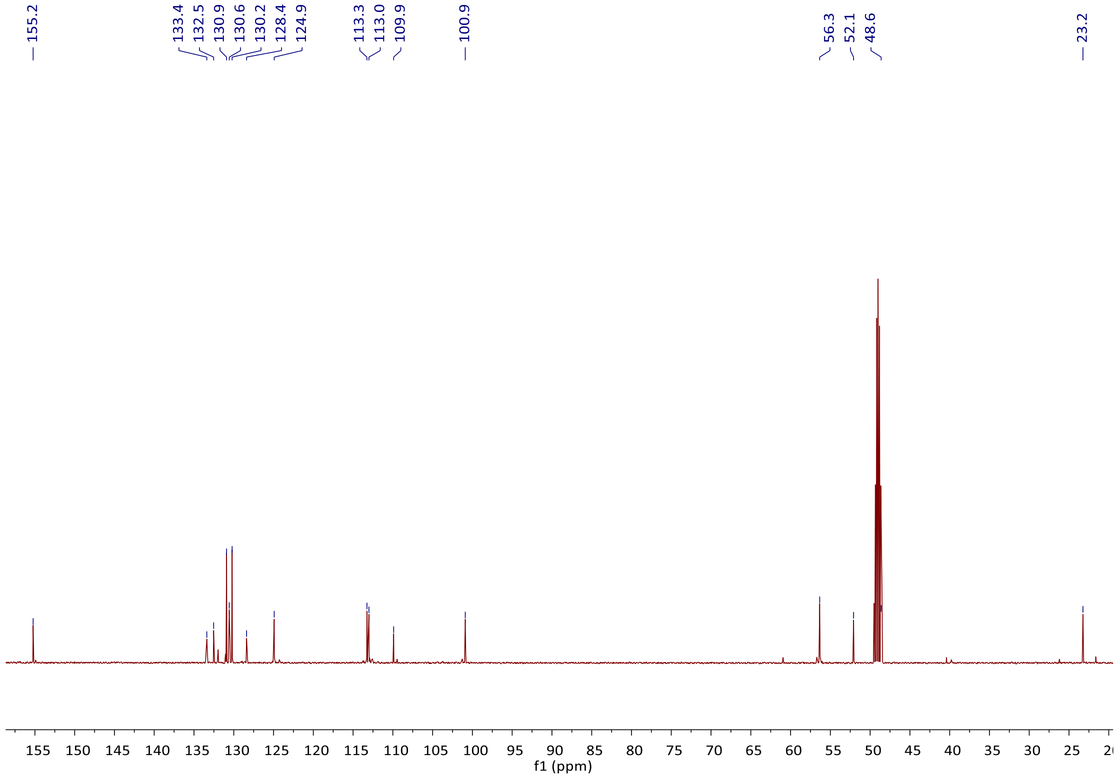

Figure 28. ^{13}C NMR (125 MHz, CD_3OD) spectrum of **6G**.

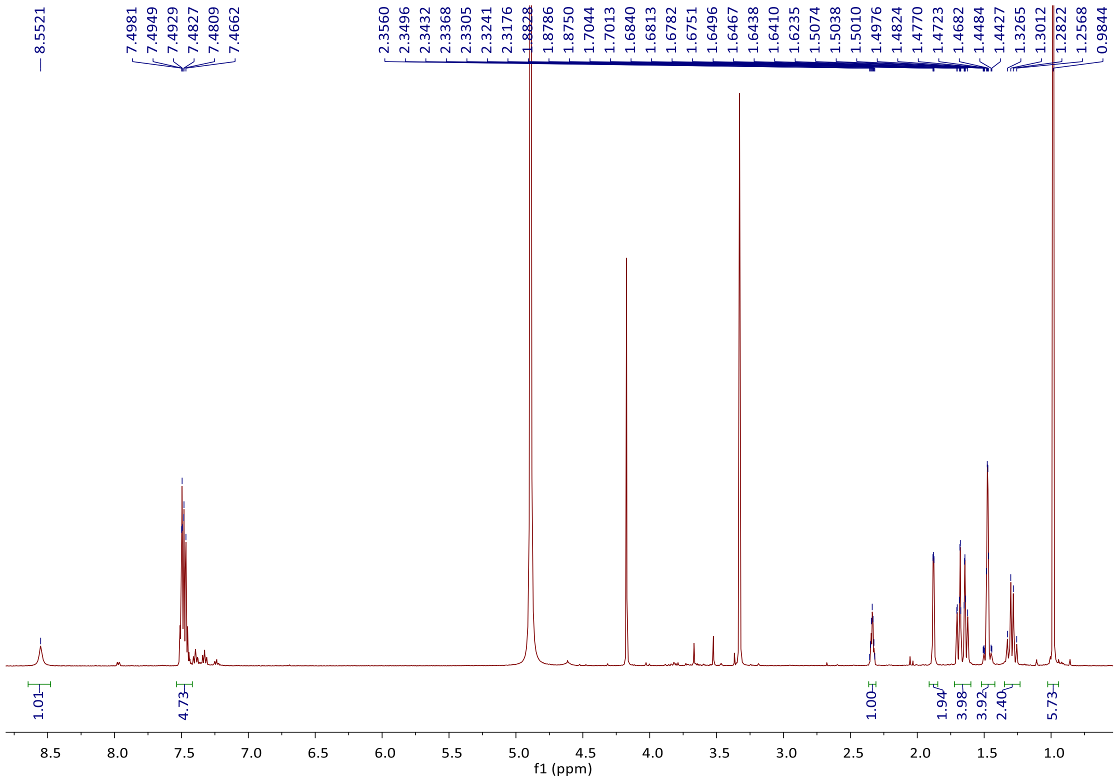

Figure 29. ^{1}H NMR (500 MHz, CD_3OD) spectrum of **6H**.

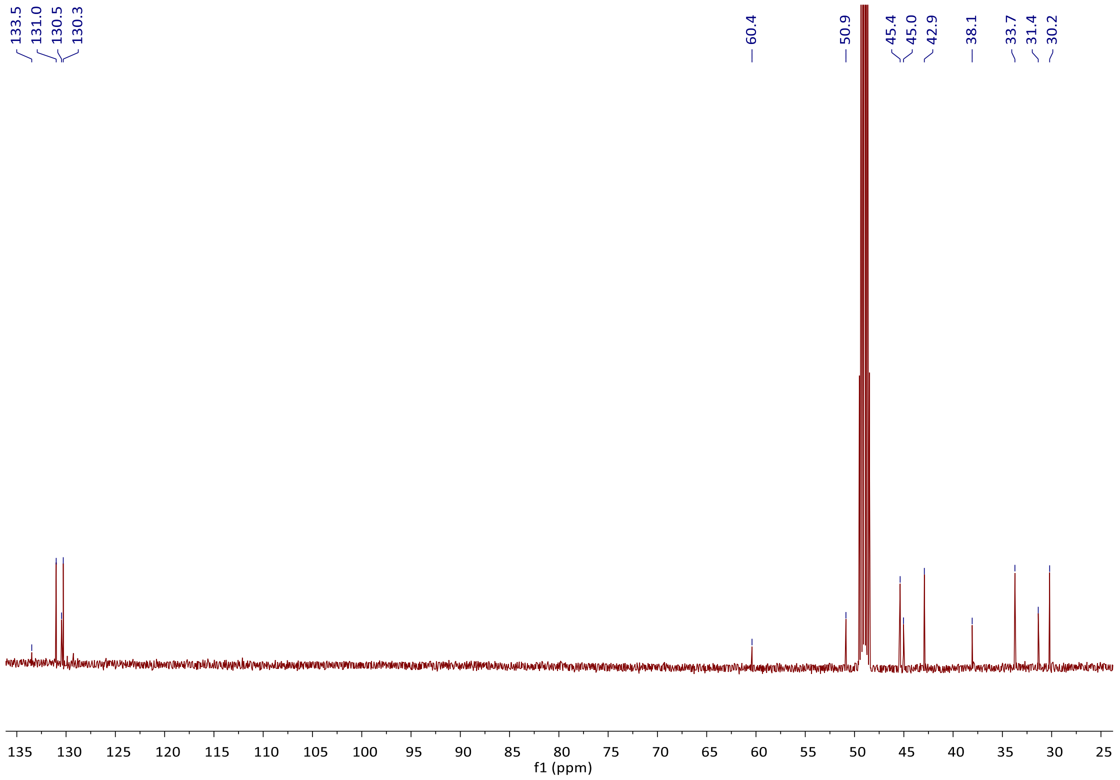

Figure 30. ^{13}C NMR (125 MHz, CD_3OD) spectrum of **6H**.

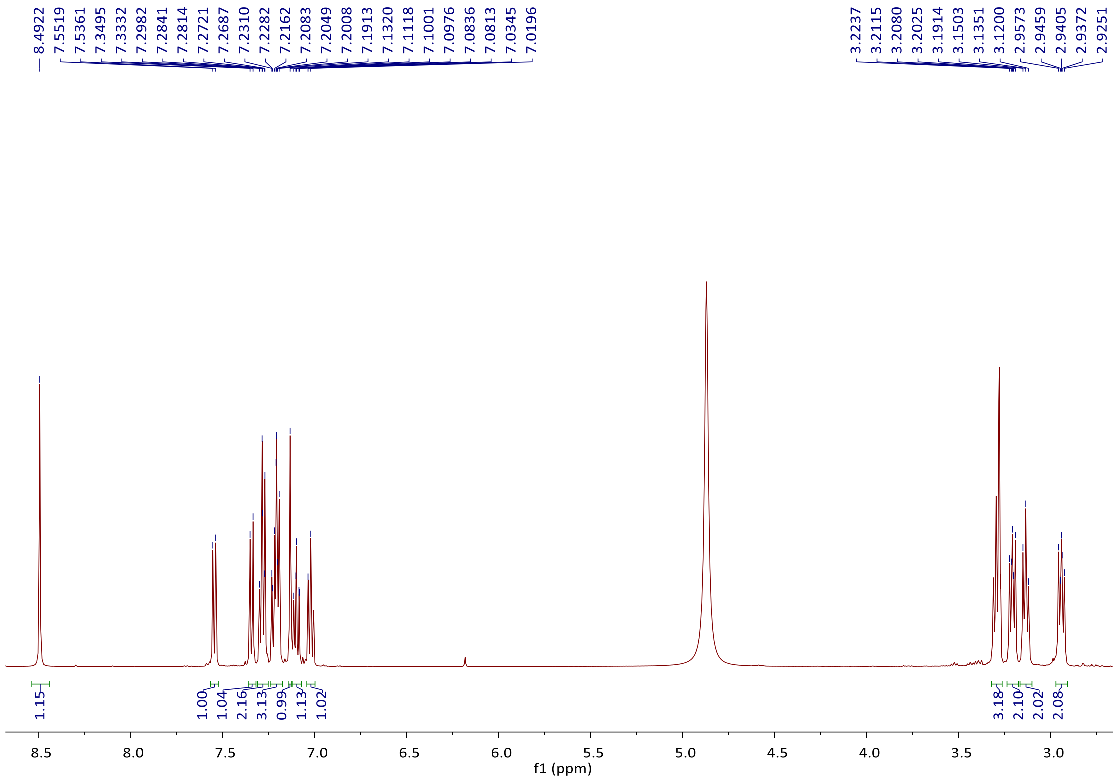

Figure 31. ^{1}H NMR (500 MHz, CD_3OD) spectrum of **7C**.

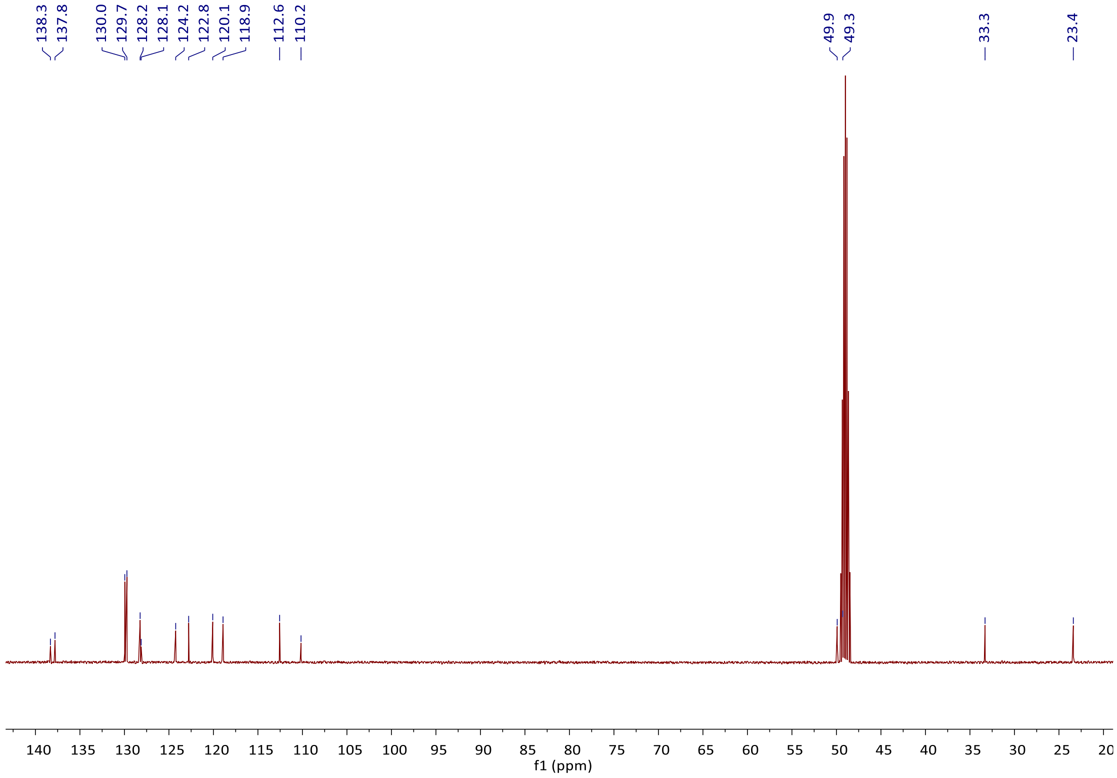

Figure 32. ^{13}C NMR (125 MHz, CD_3OD) spectrum of **7C**.

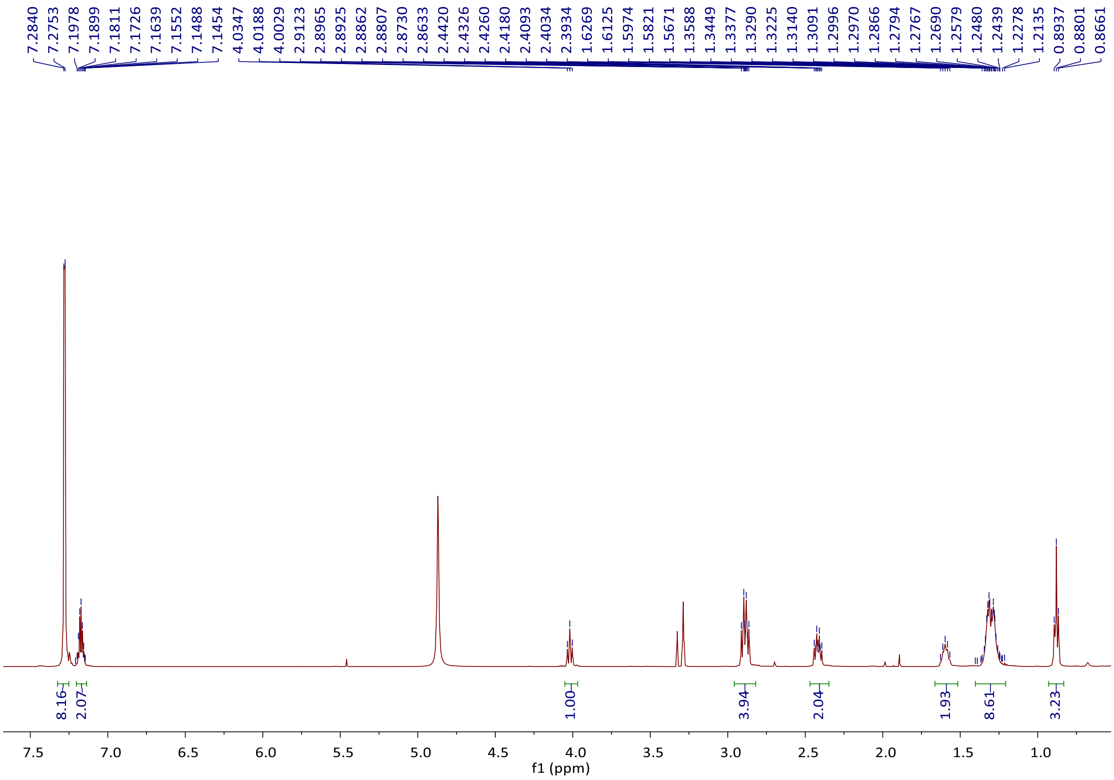

Figure 33. ^{1}H NMR (500 MHz, CD_3OD) spectrum of **8I**.

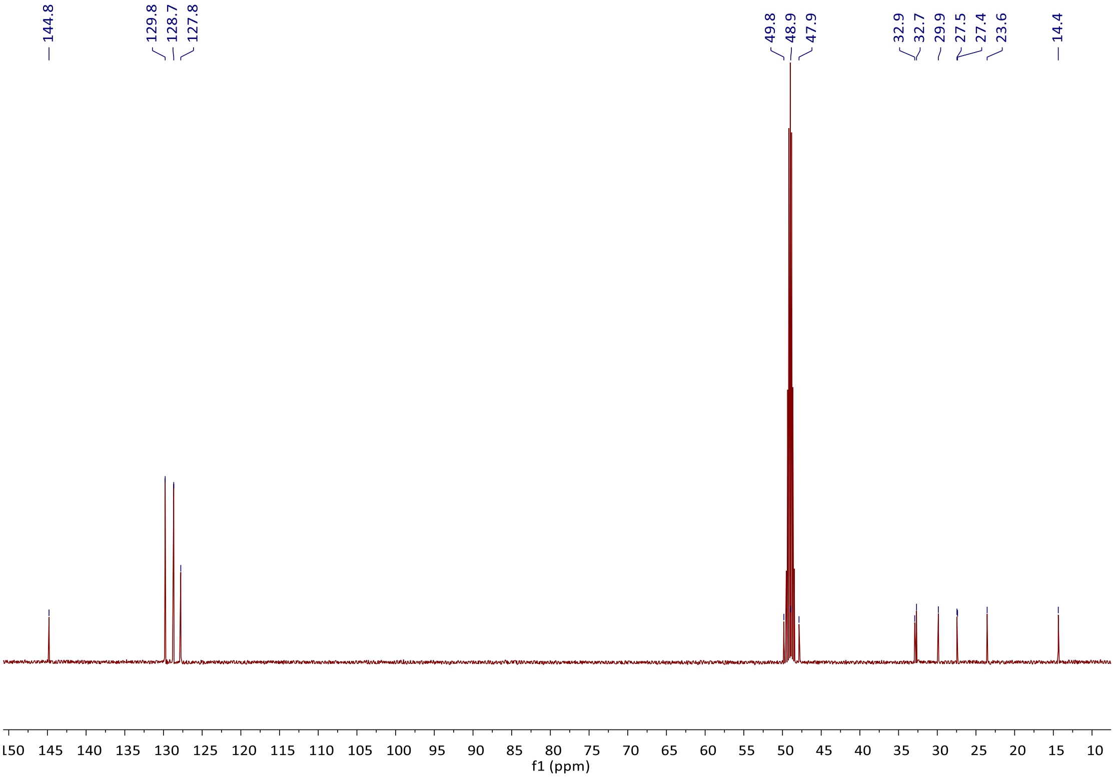

Figure 34. ^{13}C NMR (125 MHz, CD_3OD) spectrum of **8I**.

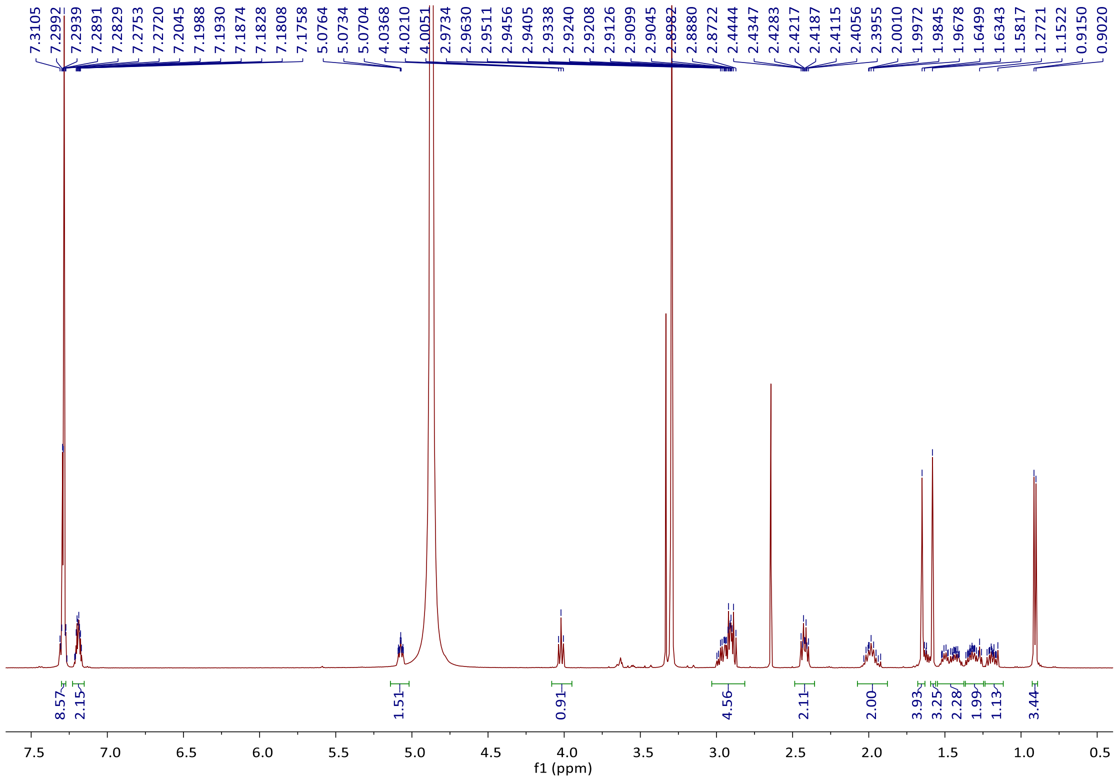

Figure 35. ^{1}H NMR (500 MHz, CD_3OD) spectrum of **9I**.

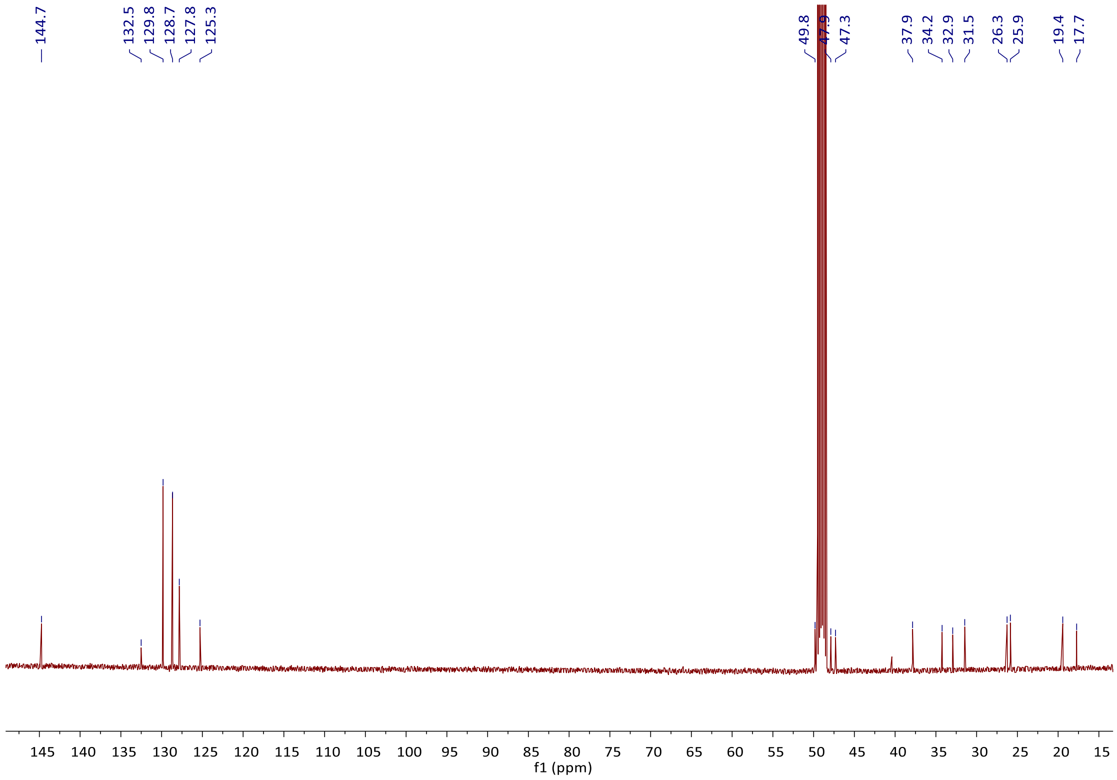

Figure 36. ^{13}C NMR (125 MHz, CD_3OD) spectrum of **9I**.
